# Intra-Host Co-Existing Strains of SARS-CoV-2 Reference Genome Uncovered by Exhaustive Computational Search

**DOI:** 10.3390/v15051065

**Published:** 2023-04-26

**Authors:** Xinhui Cai, Tian Lan, Pengyao Ping, Brian Oliver, Jinyan Li

**Affiliations:** 1 Data Science Institute and School of Computer Science, Faculty of Engineering and IT, University of Technology Sydney, Ultimo, NSW 2007, Australia; xinhui.cai@student.uts.edu.au (X.C.); tian.lan-1@student.uts.edu.au (T.L.); pengyao.ping@student.uts.edu.au (P.P.); 2 School of Life Sciences, Faculty of Science, University of Technology Sydney, Ultimo, NSW 2007, Australia; brian.oliver@uts.edu.au; 3 Shenzhen Institute of Advanced Technology, Chinese Academy of Sciences, 1068 Xueyuan Avenue, Shenzhen University Town, Shenzhen 518055, China

**Keywords:** within-host diversity, SARS-CoV-2, phylogeny, spike protein, error correction, de novo assembly

## Abstract

The COVID-19 pandemic caused by SARS-CoV-2 has had a severe impact on people worldwide. The reference genome of the virus has been widely used as a template for designing mRNA vaccines to combat the disease. In this study, we present a computational method aimed at identifying co-existing intra-host strains of the virus from RNA-sequencing data of short reads that were used to assemble the original reference genome. Our method consisted of five key steps: extraction of relevant reads, error correction for the reads, identification of within-host diversity, phylogenetic study, and protein binding affinity analysis. Our study revealed that multiple strains of SARS-CoV-2 can coexist in both the viral sample used to produce the reference sequence and a wastewater sample from California. Additionally, our workflow demonstrated its capability to identify within-host diversity in foot-and-mouth disease virus (FMDV). Through our research, we were able to shed light on the binding affinity and phylogenetic relationships of these strains with the published SARS-CoV-2 reference genome, SARS-CoV, variants of concern (VOC) of SARS-CoV-2, and some closely related coronaviruses. These insights have important implications for future research efforts aimed at identifying within-host diversity, understanding the evolution and spread of these viruses, as well as the development of effective treatments and vaccines against them.

## 1. Introduction

The COVID-19 pandemic has had an unprecedented and profound impact on the world, prompting scientists and researchers to conduct extensive studies on the genome sequences of SARS-CoV-2, the virus responsible for the outbreak. The objective of these studies is to gain a comprehensive understanding of the virus, including the mechanisms of infection and the development effective therapies and vaccines. The reference genome sequences, obtained by Zhou et al. [1] (GISAID Accession ID EPI_ISL_402124) and Wu et al. [2] (NCBI Accession ID NC045512.2), have provided the foundation for much of this research. In fact, the design of the widely used mRNA vaccines, BNT162b2 [3] and mRNA-1273 [4], is based on the reference genome sequences. They were assembled by MEGAHIT ([5]) from sequencing reads (SRR11092062) generated from a BALF sample collected from a patient early in the course of the pandemic.

MEGAHIT is a k-mer-based de novo genome assembly tool that utilizes succinct de Bruijn graphs for high efficiency. It takes an iterative strategy to build multiple succinct de Bruijn graphs from a small k-mer to a large k-mer. Throughout this process, MEGAHIT removes those edges of low frequency, which are considered potentially erroneous, removes tips that are small branches with an in-degree of 0, and merges bubbles. However, these steps may inadvertently discard important genomic information, such as within-host diversity. For example, the bubble-merging step can lead to the loss of information about alternate alleles or variants.

Tools for identifying within-host diversity can broadly be categorized into two types: those that focus on detecting base changes and those that attempt to assemble complete sequences. The former takes a reference sequence and sequencing reads as the input and identifies bases that differ from the reference at the same positions. GATK is an example of such tools [6]. However, tools of this type do not provide information on how the identified changes relate to different strains. This is because, at certain positions, different reads may exhibit different bases, causing conflict with each other, or grouping a set of changes that would produce a sequence without any reads to cover certain positions, which means this part of the sequence cannot be found in any reads. In contrast, assembly-based tools typically take sequencing read files as the input and produce contigs that they assemble from the reads. To achieve this, modified de Bruijn-graph-based algorithms are often used, such as Haploflow. However, de Bruijn-graph-based tools still have the issue of ignoring low-frequency co-existing strains due to the nature of the algorithm itself, although they usually attempt to avoid it.

This paper details an exhaustive search workflow designed to identify intra-host co-existing strains of SARS-CoV-2 from the SRR11092062 reference genome. The workflow comprises a read extraction step, error correction, and strain identification followed by phylogenetic analysis of the newly found strains to trace the evolutionary path of SARS-CoV-2 and its relationship with other coronaviruses. Additionally, the study includes protein binding analysis of the identified co-existing strains’ spike protein regions.

RNA virus populations tend to be highly genetically diverse, including multiple viral strains due to high mutation rates, natural selection, and recombinations [7]. As an RNA virus, SARS-CoV-2 is expected to mutate frequently [8]. Moreover, a recent study reported mutations of SARS-CoV-2 that evade antibody detection within a host’s body over time [9]. In addition to mutations, within-host diversity could also result from superinfection [10]. By identifying the strains present, this study can help determine which strain(s) caused the within-host diversity of the virus.

Our method successfully identified two intra-host co-existing strains of SARS-CoV-2 from the same viral sample that produced the reference sequence (GISAID ID EPI_ISL_402124). Interestingly, the nucleotide differences in both strains were evenly distributed throughout the entire reference genome. Co-existing Strain 1 exhibited twenty six nucleotide differences in the spike glycoprotein region, while Strain 2 showed ten nucleotide differences in comparison to the original reference sequence. Further analysis revealed that both strains were closely related to the reference strain of SARS-CoV-2 and equally distant from other major variants, including B.1.617 (Delta) and BA.1 (Omicron). However, the two co-existing strains exhibit different structural properties compared to the SARS-CoV-2 reference strain in the region that encodes binding to the receptor protein ACE2.

These findings provide valuable insights into the genetic diversity and evolution of SARS-CoV-2 within a host. It highlights the importance of understanding intra-host variation, which can have significant implications for disease transmission, diagnosis, and treatment.

In addition to identifying intra-host diversity in a SARS-CoV-2 reference genome sequence sample, we evaluated the effectiveness of our workflow in analyzing other RNA virus samples. Firstly, we compared the output of our workflow with the within-host diversity identification tool Haploflow [11] using a wastewater sample [12]. The results demonstrated that our workflow was able to identify within-host diversity with high accuracy and sensitivity. Furthermore, we applied our workflow to a foot-and-mouth disease virus sample [13], which revealed its potential for analyzing other RNA virus samples. These results confirmed the versatility and effectiveness of our workflow in identifying within-host diversity in various RNA viruses.

Overall, our study not only provides valuable insights into the genetic diversity of SARS-CoV-2, but also demonstrates the potential of our workflow in analyzing other RNA virus samples.

## 2. Materials and Methods

Our methodology has five main steps, which are necessary to investigate the existence of co-existing strains of virus. The five steps are: (1) extraction of non-human reads, (2) error correction of these reads, (3) identification of those reads containing mutations, (4) phylogenetic study on the genome sequences and spike proteins, and (5) binding affinity studies of the mutated spike proteins with the host receptors.

The nucleotide sequences and amino acid sequences used for phylogenetic analysis were downloaded from NCBI Genebank. The accession numbers are: MN996532.2 (RaTG13), EF203064.1 (HKU2), AT278488.2 (SARS-CoV), KC776174.1 (MERS-CoV), OM366054.1 (SARS-CoV-2 B.1.617.1), MZ724516.1 (SARS-CoV-2 B.1.617.2), MZ275302.1 (SARS-CoV-2 C.37), MW642248.1 (SARS-CoV-2 P.1), MZ068161.1 (SARS-CoV-2 B.1.351), OM616632.1 (SARS-CoV-2 B.1.1.7), OM766152.1 (SARS-CoV-2 BA.1), MN996528.1 (SARS-CoV-2 WIV04), MZ937000.1 (BANAL-20-52), MZ937003.2 (BANAL-20-236).

The sequencing reads were downloaded from SRA [14] with accession numbers from SRR11092056 to SRR11092064. The dataset SRR11092062 has the best quality and contains the most reads of SARS-CoV-2 and the highest average coverage for the reference genome sequence. It is the sample that was used to assemble the SARS-CoV-2 reference genome for GISAID, so we decided to use it as the primary dataset in this work. The other read files, SRR11092057, SRR11092058, SRR11092059, SRR11092060, SRR11092061, SRR11092063, and SRR11092064 were also sampled in late 2019 in Wuhan and provided to us by the team of Zhou et al. [1].

**Figure 1:**
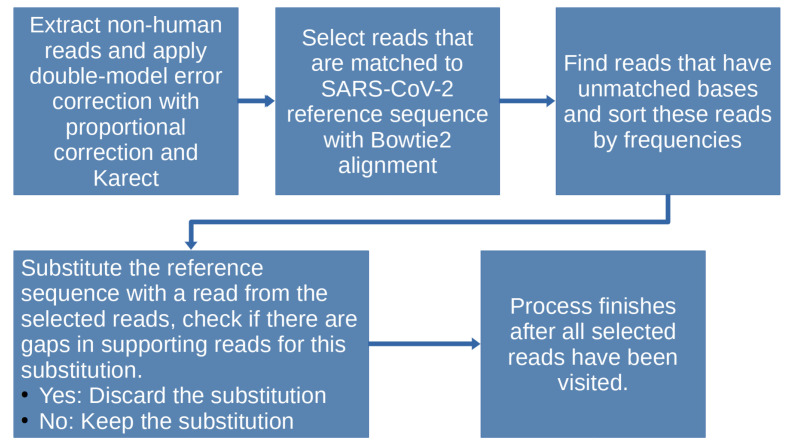
Strain identification process. The process begins with extracting non-human reads from the sample and applying double-model error correction to it. Aligning the corrected reads with the SARS-CoV-2 reference genome would allow the selection of reads that are related to the virus. From the related reads, select those with mismatches to the reference genome as candidate reads possibly belonging to co-existing strains. Then, proceed with the substitution process for identifying the co-existing strains from the reads that are compatible with each other.

A graphical representation of our method for co-existing strain identification is presented in Figure 1. The code and detailed instruction can be found in this Github repository: https://github.com/RyanCairepo/strain_identify/tree/master, accessed on 22 April 2023.

### 2.1. Non-Human Reads’ Extraction

To begin the workflow, the raw sequencing data underwent a filtering process to remove human-related reads. As the sample (SRR11092062) was taken from a COVID-19 patient’s BALF, it inevitably contained reads of human genes. While the authors of the reference genome sequence filtered out mammalian genes with an unpublished local database, for this work, reads of human genes were filtered out using Bowtie2-2.44-linux [15] with the –large-index and –local options. The GRCh38.p13 human DNA sequence [16] was used as the reference for the filtering process. Out of the 122,608,060 reads in the raw paired-end read set, 11,752,058 reads remained after filtering. This process reduced the total size of the files from 48.2 GB to 4.2 GB. These remaining reads were considered close to the input for MEGAHIT in the work of Zhou et al. [1] and, thus, were used for the next stage of the workflow.

### 2.2. Error Correction for the Non-Human Reads

To increase the reliability of reads used for identifying new strains, we implemented a double-model error-correction mechanism. The dataset used for this analysis was SRR11092062, a paired-end NGS RNA-sequencing reads dataset with a possible error rate of 0.5–2% [17]. As errors in reads could interfere with the identification of new strains, we combined two error-correction tools, each tackling a part of the potentially erroneous reads.

The first tool we used is called proportional error correction. It is a model based on miREC [18] called smallRNAproper (small RNA sequencing reads’ restoration) [19], which works with the whole reads instead of with k-mers. The idea behind this tool is to identify potentially erroneous bases by comparing their frequency. Reads with a frequency of one are more likely to have errors due to the nature of PCR amplification. On the other hand, reads with a frequency greater than one are more likely to be error-free. Therefore, the correction involves substituting the erroneous reads with their correct counterparts, using reads with a frequency greater than one to correct those with a frequency of one. The reads were 150 bases long, and the number of possible erroneous nucleotides was most likely less than 2 (the error rate of the Miseq sequencing platform could be above 1.5%, but less than 2% [20]). This step corrects a large part of the possible erroneous reads, but may still leave some of the errors untouched because not all reads with a frequency equal to one will have a correct counterpart. These reads are then handled by the second correction tool, Karect [21].

The second model of error correction involves separating reads with a frequency of one from the input files and applying Karect to generate reads with higher frequencies from singletons using multiple sequence alignment techniques. This approach is effective in dealing with errors in singleton reads that are not handled by proportional correction. However, it is important to note that Karect may also generate a significant number of new reads that do not actually exist in the original data, as demonstrated by the creation of 37,678 new sequences out of the 127,642 reported errors by Zhang et al. [18]. To address this issue, we retained only those reads with a frequency greater than one and an edit distance of one compared to their original state.

### 2.3. New Strain Identification

To further extract reads related only to SARS-CoV-2, the corrected reads were aligned against the reference strain sequence EPI_ISL_402124 using Bowtie2 (with mode set to “local”). Bowtie2 was chosen after evaluating alignment tools, which included Bowtie2, minimap2, and bwa-mem2, on the SARS-CoV-2 reference genome sequence and the raw reads of SRR11092062. While minimap2 is known for its speed with long reads, it is not optimal for short spliced reads, and bwa-mem2’s documentation lacks details, making it difficult to adjust the parameters for relevant results. Benchmarking by Musich et al. [22] showed that Bowtie2 is one of the best aligners for NGS reads, with complete functionality for adjusting mismatch punishment and changing the CIGAR string syntax. Since speed was not a concern in this scenario, Bowtie2 was deemed the most-suitable tool for the task.

After extracting SARS-CoV-2-related reads using Bowtie2 with an increased tolerance for the penalty of mismatched bases (–score-min G,10,4 - D 25 -R 3 -N 1 -L 20 -i S,1,0.50), we narrowed our focus to a subset of reads that exhibited minor variations from the reference sequence. Given the high similarity among different SARS-CoV-2 variants [23], we identified these reads as potential candidates for substitution. Distinguishing variations within reads entails recording their position and type. Each candidate read containing different bases is inserted into the reference genome individually and without conflicts, preserving prior changes. Substituted reads are typically non-overlapping and are more likely to belong to the same strain, as dictated by enzymatic fragmentation [24]. Following each substitution, an alignment was conducted to confirm its validity, i.e., whether the reads fully cover the updated sequence. To be considered, all reads in this subset must be perfectly matched and paired with the updated sequence. A matrix was constructed from these reads, with rows representing the bases and columns representing the positions in the reference sequence. The substitution was deemed valid and retained if the matrix had no empty columns; otherwise, it was reverted.

In addition to the best-coverage sample SRR11092062, Zhou et al. [1] made available four additional samples from SARS-CoV-2 patients in Wuhan along with their paper that published the reference sequence (SRR11092057–SRR11092064). These samples are characterized by lower numbers of SARS-CoV-2 reads and were generated with smaller numbers of reads relative to SRR11092062. Compared to SRR11092062, the remaining samples collectively had less than one-fifth of the reads aligned with SARS-CoV-2. Moreover, the reference genome sequence could not be supported by perfectly matched reads in any of them (many positions were not covered by any reads). Therefore, we verified read substitutions using these four samples, given the possibility that these patients may have been infected with or developed within-host diversity similar to the discovered strains of SARS-CoV-2.

The verification process began by separating SARS-CoV-2 reads via alignment against the original strain reference sequence. Using the original strain as a reference sequence ensured that reads from other strains of SARS-CoV-2 were not missed, as differences among strains typically do not appear as long, continuous streaks of nucleotide bases. After separating the SARS-CoV-2 reads, each substituted base was inspected for supporting reads that had the same base at the same position or a source read frequency greater than one. Once a supporting read was found, the inspection proceeded forward and backward to determine how many bases in the read matched the discovered strain before a mismatch occurred, thereby creating a segment that spanned one or multiple reads. If the end or head of the read was reached without encountering a mismatch, the program shifted to the read before or after the current read and started from the same position relative to the discovered strain sequence. To expand the segment, each read that the process traversed through must not conflict with the position of the current substituted base. If a substituted base has no support in other samples, has a read frequency of only one, and the segment is shorter than 50 bases, the substitution was reversed from the resulting sequence, and another read was processed with the same substitution procedure.

The process yielded several outputs, including FASTA files containing the complete nucleotide sequences of the two identified strains and their spike gene sequences. Additionally, lists of the differences for each strain on the original strain sequence were generated. The amino acid sequences of the discovered strains’ spike genes were also translated to identify non-synonymous changes, which resulted in alterations to the translated amino acid sequences.

### 2.4. Phylogenetic Study

Accurate identification of the phylogeny of co-existing strains to the reference strain, major later variants of SARS-CoV-2, and other coronaviruses is a critical task. To achieve this, we included a group of genome sequences from bat coronaviruses, SARS-CoV, MERS-CoV, and other major variants of SARS-CoV-2 in our phylogenetic analysis. We performed a multi-sequence alignment (MSA) on these sequences using MAFFT v7.543 [25] and constructed two phylogenetic trees using FastTree 2.1.11 [26] for the spike protein sequences and the whole-genome sequences. In addition, PHYLIP’s dnapars v3.698 [27] was used to confirm the phylogenetic tree constructed from the genome sequences. To run MSA for nucleotide sequences, we utilized the recommended –6merpair option, while for protein sequences, we employed the –amino, –maxiterate 50, and –localpair options, as recommended on the MAFFT website.

To build the phylogenetic tree, we carefully selected a group of viruses that have a potential relationship with the SARS-CoV-2 reference strain. The selected bat coronaviruses, RaTG13, HKU2, BANAL-20-236, and BANAL-20-52, were included because of the similarity of their spike genes to SARS-CoV or SARS-CoV-2. Additionally, we included SARS-CoV and MERS-CoV to provide a broader perspective. Notably, the HKU2 bat coronavirus’ spike protein is suspected to have a common evolutionary origin with bat-CoV HKU2, bat-SARS-CoV, and SARS-CoV [28]. BANAL-20-52 is particularly interesting because it has a spike protein that is 98.4% similar to the original SARS-CoV-2 strain spike protein.

Our goal was to identify whether the newly discovered strains were closer to some of the other coronaviruses or SARS-CoV than to the reference genome, providing potential clues for identifying an evolutionary pathway from SARS-CoV or other coronaviruses to SARS-CoV-2. Therefore, we included all the WHO variants of concern (B.1.1.7, B.1.617.2, P.1, B.1.351, and B.1.1.529/BA.1) and some other variants of interest (C.37 and B.1.617.1) in our analysis. This allowed us to examine whether some of the variants were closer to the discovered strains than to the reference genome. By doing so, our phylogenetic tree can provide insight into how close our newly discovered co-existing strains are to the SARS-CoV-2 major strains and other bat coronaviruses.

### 2.5. Protein Binding Affinity Investigation

To determine the 3D folding structures of the spike protein amino acid sequences for co-existing strains, we first translated their entire nucleotide sequences into amino acid sequences using exPasy [29]. Next, we utilized Alphafold2 with MMseqs2 [30,31] to predict the structures with the parameters’ multi-sequence alignment mode (MSA mode) set as mmseqs2_uniref_env and pair_mode set as unpaired_paired. We obtained the spike protein structure of the SARS-CoV-2 reference strain from the Protein Data Bank (PDB) [32] under the accession number 7CWL and the ACE2 structure under the accession number 6M1D for comparison. To find the most-suitable model, we used the average of the predicted local distance difference test (PlDDT) [33] on each site and selected Model 3 from the 5 protein structure models produced by Alphafold2 based on this metric (see Table 1, Table 2 and Table 3).

To examine the protein binding affinity, we assessed the docking energy scores using HDOCK1.1 [34] with default parameters. The tool provided predicted docking models with protein docking scores. We measured the scores between Model 3 and human ACE2 using this tool.

## 3. Results

We applied our workflow to two samples, namely SRR11092062 and SRR12596175. SRR11092062 was collected from a patient in Wuhan in 2019, and it was used to assemble the SARS-CoV-2 reference genome sequence. SRR12596175, on the other hand, was collected from a wastewater plant in California in 2020. Both samples appeared to contain two additional strains’ sequences, in addition to the contig sequence produced by MEGAHIT. We constructed phylogenetic trees using the discovered strains’ sequences from both samples. Furthermore, we predicted the spike protein binding affinity using the discovered strains from the SRR11092062 sample. Additionally, we also performed strain identification on an FMDV sample ERR034193 collected from an infected animal.

### 3.1. Results from the Wuhan Sample

#### 3.1.1. Error Correction

A total of 234,432 reads out of the 11,752,058 reads were corrected using the proportional correction method, while only 35 reads were corrected using Karect because we carefully avoided generating non-existent reads. Five of the corrected reads were found in the co-existing Strain 1, all of which had a frequency greater than one. This outcome served as evidence that our error correction procedure yielded improvements in the quality of reads for downstream analysis.

#### 3.1.2. Differences in Sequences

We identified two co-existing strains of the SARS-CoV-2 reference genome using our designed workflow on sample SRR11092062. The following analysis was based on the nucleotide sequences and spike protein amino acid sequences of these two strains.

Compared to the original strain of SARS-CoV-2, Co-existing Strain 1 exhibited nucleotide base differences across the entire region of its spike gene. Specifically, we observed 22 non-synonymous differences (i.e., differences that affect the translated protein amino acid sequence) and 4 synonymous differences in the spike gene region of Strain 1. Among the 179 observed differences, 115 were located inside the opening reading frame (ORF) region, 5 in the nucleocapsid (N) protein, 12 in the membrane (M) protein, 1 in the envelope (E) protein, and the remaining differences in non-structural (3 NS3) proteins.

In contrast, Co-existing Strain 2 exhibited fewer nucleotide base differences across the entire sequence, including 1 synonymous change and 9 non-synonymous changes in the spike region. About 67 of the 89 different bases were located in the ORF region, 2 in the N protein, and the rest in NS8 proteins. Compared to Co-existing Strain 1, Strain 2 had more differences in the N protein region, but fewer differences in the spike protein region. Additional details about the two co-existing strains’ nucleotide sequences, spike protein sequences, and the differences in the spike protein compared to the SARS-CoV-2 reference sequence (Tables S1–S7, File S1) are provided in the Supplementary Materials. We identified these base differences using the alignment tool EMBOSS Needle [35].

The amino acid mutations in the spike proteins of the discovered strains were distinct from those found in later-mutated strains of the virus (prior to Omicron), indicating that our newly discovered strains may be ancestors or siblings of the SARS-CoV-2 reference strain, rather than descendants. Notably, the discovered strain 2 contains the A27S mutation, which is also found in some Omicron variants (BA.2, BA.4, BA.5, BA2.12.1, BA2.75). However, other differences in the discovered Strain 2 do not match these Omicron variants.

**Figure 2:**
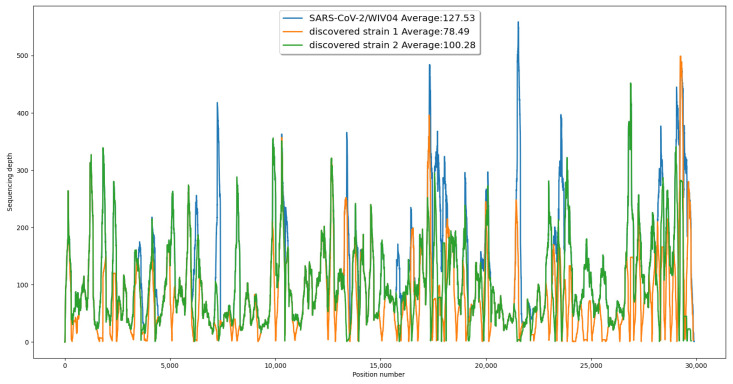
The coverage at each position of the sequences. The coverage of a base is calculated by how many reads cover it. The average sequence coverage for the reference genome was higher than both discovered strains. At some positions, the discovered strains’ sequences had a higher coverage than the reference genome sequence, which indicates identification of more dominant bases in the discovered strains.

To provide further evidence for the validity of our results, we generated a coverage chart comparing the original reference strain to our newly discovered co-existing strains. This chart demonstrated that all three sequences were fully supported by the reads, with a similar pattern of coverage at each position (see Figure 2).

We formed the sequences of the two identified strains from the reads that passed the verification step. For Strain 1, out of the 195 reads initially included, only 88 reads remained after verification. The 107 reads that were removed had a frequency of one and could not be confirmed by consecutive segments. Similarly, for Strain 2, only 24 out of the original 146 reads could be verified. The verification step allowed us to increase the reliability of the sequences of the discovered strains.

#### 3.1.3. Comparison with Other Tools

Three other quasi-species genome assembly tools, namely PEHaplow [36], Haploflow [11], and GATK4.2.5.0 HaplotypeCaller [6], were employed to analyze SRR11092062. While Haploflow had previously identified variant strains of SARS-CoV-2 in wastewater samples, it failed to produce reliable results for SRR11092062, even after more than 168 h of supercomputer processing time. Haploflow could not generate any contigs longer than 1500 bases, and it produced similar results on our size-reduced and error-corrected version of SRR11092062 (non-human reads set) in a much shorter time. HaplotypeCaller detected three mutations at positions 306, 565, and 17,825, which were also identified by our approaches. However, HaplotypeCaller proposed a substitution at position 306 that could not be supported by the reads, resulting in a newly formed contig at positions 310–324 with 0 coverage. PEHaplo returned errors when run with default arguments and the SRR11092062 reads, and setting up the environment for PEHaplo using conda [37], as proposed by the authors, took more than 48 h.

#### 3.1.4. Phylogenetic Relationship Analysis

**Figure 3:**
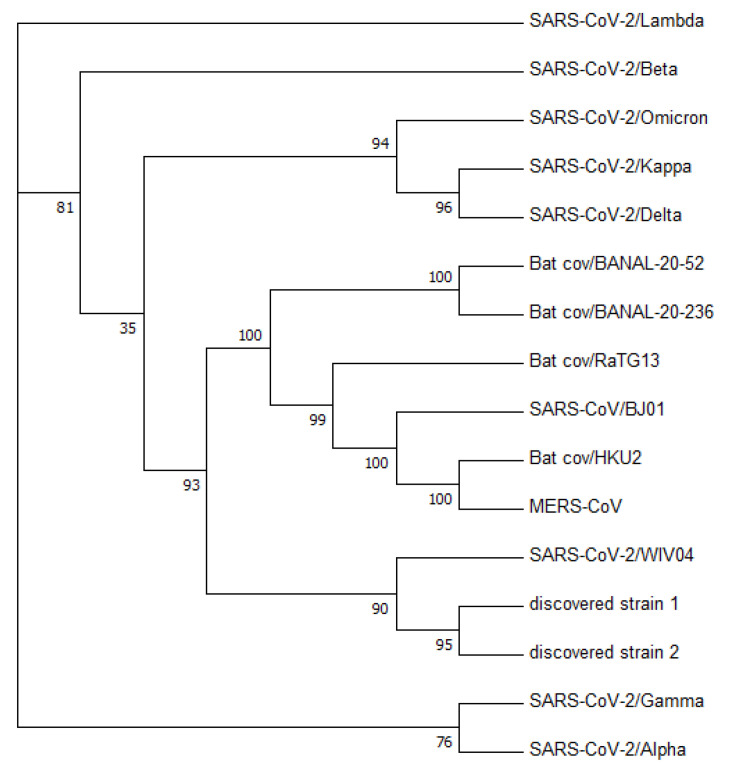
Phylogenetic tree constructed from nucleotide sequences using FastTree. The number denotes the confidence of the maximum-likelihood calculation by FastTree. It shows with a high confidence that the two discovered strains are closely related to the reference genome.

**Figure 4:**
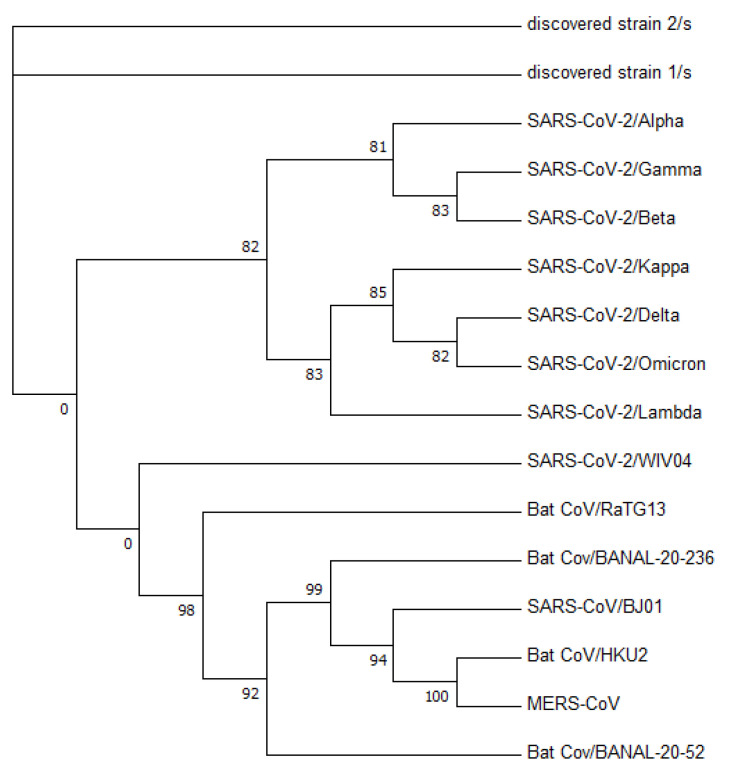
Phylogenetic tree constructed from the spike protein sequences. The two zero edges are persistent with both Fasttree [26] and iQTree2 [38].

Figure 3 displays a phylogenetic tree that was constructed using whole-genome sequences, while Figure 4 presents a phylogenetic tree based on the spike proteins of selected coronaviruses. Notably, the latter tree exhibits varying levels of similarity among the coronaviruses when compared to the former, which was constructed using whole-nucleotide sequences. Both trees indicate that the two newly discovered strains were more closely related to the original SARS-CoV-2 strain WIV04 than to any other coronaviruses.

**Figure 5:**
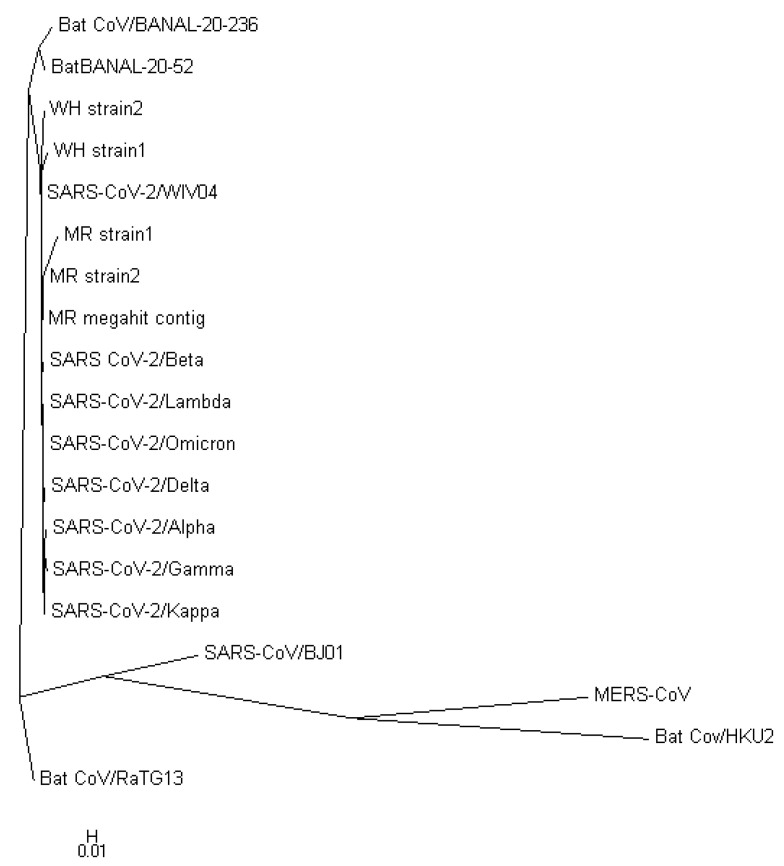
Phylogenetic tree constructed from the nucleotide sequences using PHYLIP’s dnapars, a parsimony-based software for phylogenetic tree construction. Dnapars does not provide a confidence value for the constructed tree, which is different from Fasttree.

To validate the accuracy of our constructed trees, we also utilized PHYLIP’s dnapars [27] to construct another phylogenetic tree using whole-nucleotide sequences. It is worth noting that PHYLIP’s dnapars utilizes parsimony methods, which differ from the maximum-likelihood algorithm utilized by FastTree. Nevertheless, the PHYLIP tree reaffirmed our previous findings, indicating that the sequences of the two newly discovered strains are more similar to the reference genome than to any other major variants Figure 5.

Tables S1 and S2 outline the variations observed in the nucleotide and spike protein sequences of the discovered Strain 1, the discovered Strain 2, and the original strain of SARS-CoV-2. These tables provide detailed information on segment lengths and read frequencies, which are crucial factors in determining the reliability of the substitutions. Segment size is determined by identifying the number of continuous matching bases between the substituted read and other reads that overlap with its positions in multiple samples. Additionally, read frequency was taken into account.

To be included in this analysis, our criteria were that either the frequency of the read was greater than one (indicating that the read appeared more than once in SRR11092062) or the segment size was larger than or equal to 50. Notably, both discovered strains exhibited a closer relationship to the reference genome sequence, as confirmed by their nucleotide and spike protein sequence similarities. Interestingly, the top-two highest similarities for both discovered strains were observed when compared to each other and to the reference genome sequence. Our analysis further revealed that both discovered strains displayed synonymous changes at positions 3625 and 22,114, while at position 10,451, they exhibited a non-synonymous change.

Table 4 and Table 5 present the results of the similarity analysis of spike protein sequences. The low-similarity rates observed between the chosen coronaviruses in both tables indicate that the spike protein amino acid sequences are more suitable for similarity analysis than the whole-nucleotide sequences, owing to their higher stability across different sequences of the same virus.

The comparison of the spike protein sequences among the chosen coronaviruses revealed that the original SARS-CoV-2 strain (WIV04) shares more bases with all other coronaviruses than the discovered Strain 1 and the discovered Strain 2. Specifically, the discovered Strain 1 has approximately 1% bases that differ from the original SARS-CoV-2 strain, while the discovered Strain 2 has only about 0.3% differing bases. In comparison, the percentage of differences observed in the whole-nucleotide sequences is even smaller.

Our analysis further showed that the phylogenetic tree constructed from the spike protein sequences places the discovered Strain 1 and the discovered Strain 2 closer to the SARS-CoV-2 viruses than to the other bat coronaviruses. Table 4 and Table 5 provide a summary of the similarity comparison results for spike protein sequences and the whole-nucleotide sequences, respectively.

#### 3.1.5. Protein Binding Affinity Study

We employed Alphafold2 [30,31] to predict the 3D structures of the spike proteins for the two co-existing strains and examined their docking status with the human cell surface protein ACE2. The top-10 models of the discovered Strain 1, the discovered Strain 2, and the original strain were selected based on their docking scores and ligand root-mean-squared deviation (RMSD), and their binding affinities are summarized in Table 1, Table 2 and Table 3. Our results indicated that the binding affinity of the spike protein of the co-existing Strain 1 with the human receptor ACE2 was about 4.84% lower than that of the original strain’s spike protein, while the co-existing strain 2’s spike protein exhibited a 9.78% decrease in binding affinity.

The complex structure formed by the spike protein of the co-existing Strain 1 and the human ACE2 receptor is comparatively less stable than that formed by the original strain’s spike protein. Moreover, the complex structure formed by the co-existing Strain 2’s spike protein shows increased instability in comparison with that of the co-existing Strain 1’s spike protein. Therefore, both discovered strains are likely to be less contagious than the original SARS-CoV-2 strain.

### 3.2. California Wastewater Sample Results

In mid-2020, Crits-Christoph et al. [12] collected sewage samples from the San Francisco Bay Area in California, USA, and identified multiple viral genotypes, including several SARS-CoV-2 sequences. These SARS-CoV-2 genomes were found to be more closely related to those observed in California than those in other regions of the U.S. The authors submitted 11 samples to the Sequence Read Archive (SRA). For this study, we utilized the SRR12596175 sample collected from Marin as it had a high abundance of SARS-CoV-2-related reads, and we were able to identify the corresponding haploflow-resulting sequences.

To avoid confusion with the discovered strains from SRR11092062, we refer to the SARS-CoV-2 strains discovered from the wastewater sample as the “MR discovered strains”.

To determine whether the submitted sequence from Crits-Christoph et al. [12] was supported by the reads from SRR12596175, an alignment was performed, which revealed 25 gaps in the sequence. This suggested that the submitted sequence may not be supported by the reads and could be due to RNA degradation or may not be from a SARS-CoV-2 strain that exists in the sample. As a result, we assembled a consensus sequence using MEGAHIT 1.2.9 with default options, which contained the longest contig produced by haploflow, with dozens of bases extended from the haploflow contig at the beginning and end of the sequence. The resulting sequence was supported by the reads according to the alignment results and is likely the dominant SARS-CoV-2 strain sequence in the sample.

#### 3.2.1. Differences in the Sequences of Discovered Strains

After obtaining the sequence of the possible dominant strain, we used a strain identification program to detect whether within-host diversity existed. The program identified two sequences, each with at least 10 reads with a frequency greater than one. We verified the substituted reads by comparing them to 10 additional samples submitted under PRJNA-661613 by Crits-Christoph et al. [12], which were collected within California and, therefore, may contain the same circulating SARS-CoV-2 strains. Since the protein information is not available for the assembled contig, we estimated the possible spike protein region by translating the entire sequence with Expasy [29]. We located the index of the spike region from the possible spike protein amino acid sequence, which was a consecutive region of 1000+ bases at the second half of the translated sequence. The translated spike protein sequence of the assembled contig was almost identical to the SARS-CoV-2 reference genome sequence’s spike protein, except for base 623. However, Expasy identified nine additional bases at the beginning that also belonged to the MR-assembled contig’s possible spike protein region.

After examining the sequences, we found that the MR discovered Strain 1 of SRR12596175 exhibited several insertion/deletion sites, including 2 deletions and 2 other non-synonymous changes, out of a total of 8 changes in the spike region. The total number of changes in the MR discovered Strain 1 was 63, which is significantly higher than the number of changes in the MR discovered Strain 2, where only 17 changes were identified with 2 in the possible spike regions. Details of the changes for both MR discovered strains are listed in Tables S3 and S4. However, it is important to note that the translated amino acid sequence of the MR discovered Strain 1 in the possible spike region was not a contiguous segment like the MR discovered Strain 2. Instead, it consisted of several fragmented pieces, with the longest fragment containing 422 amino acid bases. As a result, the inference regarding the synonymous status of this region is not very reliable.

The complete nucleotide sequences of the MEGAHIT assembled contig, MR discovered strain 1, MR discovered strain 2, and their corresponding spike proteins, as well as the differences of spike proteins (Figures S1–S4, File S1) can be found in the Supplementary File.

#### 3.2.2. Phylogenetic and Sequence Similarity Analysis

The nucleotide sequences of the two strains discovered in MR, as well as the possible dominant strain assembled by MEGAHIT from wastewater sample SRR12596175, showed higher similarity to the original SARS-CoV-2 strain than to any other variants of concern (VOCs) listed in Table 4 and Table 5. Although the possible spike protein sequence of the MR discovered Strain 1 showed a broken region, making its result unreliable, the MR discovered Strain 2’s possible spike protein sequence demonstrated a closer relationship to the original SARS-CoV-2 strain than other VOCs.

**Figure 6:**
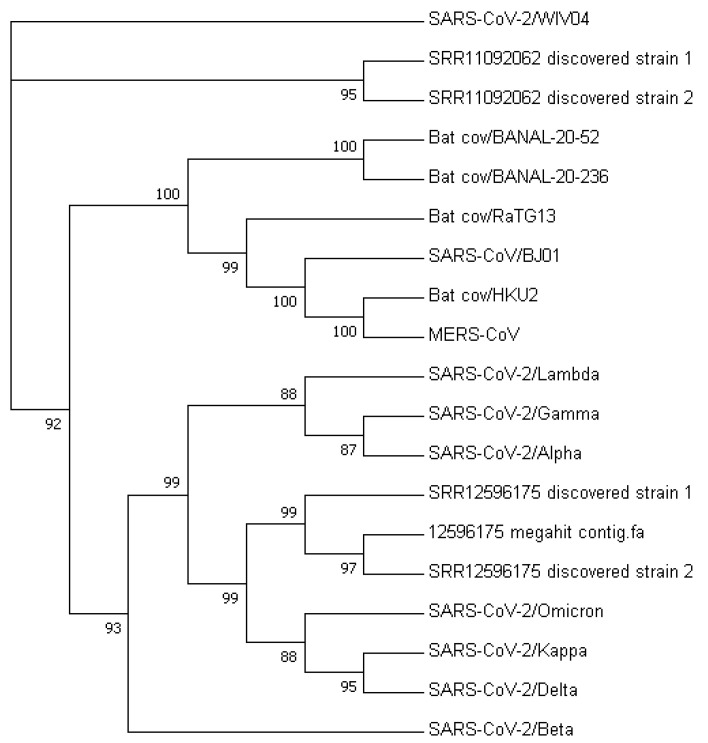
Phylogenetic tree constructed from nucleotide sequences of MR discovered strains.

**Figure 7:**
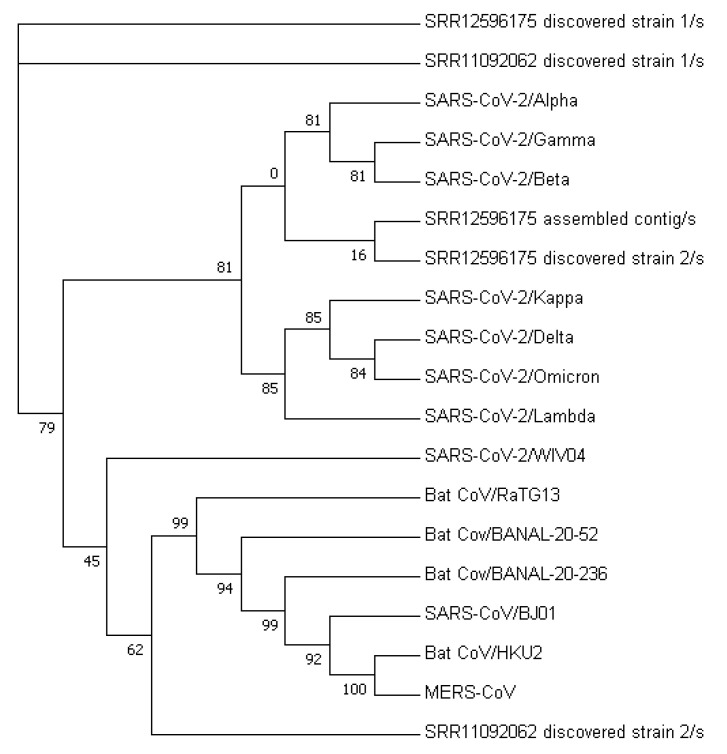
Phylogenetic tree constructed from the spike protein sequences of MR discovered strains.

The phylogenetic tree constructed from the nucleotide sequences of the MR discovered strains (see Figure 6) suggests that they belong to their own clade, which is closely related to the Omicron Variant B.A.1 compared to other VOCs. However, the tree constructed from spike protein sequences revealed that the MR discovered Strain 2 and the possible dominant strain have a closer relationship to the Alpha variant (see Figure 7).

### 3.3. Foot-and-Mouth Disease Virus Animal Samples

Foot-and-mouth disease is caused by the highly contagious foot-and-mouth disease virus (FMDV), a non-enveloped RNA virus belonging to the Picornaviridae family. The FMDV is known to devastate the economy due to its ability to transmit from animals to humans through direct or indirect contact [39]. The high mutation rate exhibited by the FMDV as an RNA virus makes it easier to identify its within-host diversity with next-generation sequencing (NGS) technologies [13].

In a study by Wright et al. [13], the FMDV was sequenced from animals using the Illumina Genome Analyzer. The researchers identified polymorphic sites from the FMDV reference genome sequence in the U.K. in 2007 [40] and the consensus sequences assembled from the samples. However, while the study provided information about the polymorphic sites, the authors did not show how those sites would form different strains of viruses, which is a crucial aspect of understanding the FMDV’s diversity.

We utilized MEGAHIT v1.2.9 with default options to generate consensus sequences from the sequencing read files provided by Wright et al. [13]. Our analysis revealed that 2 out of 6 read files produced identical sequences to the FMDV reference genome sequence from the UK in 2007, while the remaining files contained several mismatches. We selected the read file ERR034193 to assemble a consensus sequence as it included all five substitutions identified by the authors, which enabled us to demonstrate our workflow on an RNA virus other than SARS-CoV-2.

After applying our strain identification process to the read file, we successfully identified multiple co-existing strains, each with over 170 changed bases from the consensus sequence. These strains shared approximately 15–20 changed bases with one another. The authors proposed over 2000 high-frequency polymorphic sites, but not all of them could be assigned to a single or two strains due to some sites having multiple possible bases. Additionally, combining sites from different strains would result in a lack of actual reads to support the sequence.

The first identified strain contained four out of the five proposed substitutions that were consistent with the reference genome sequence. Other strains generally contained one or two of the five substitutions, sometimes with different bases from both the consensus sequence and the reference genome sequence. For example, at position 3138, Strain 2 showed an A instead of the T in the reference genome or the G in the consensus sequence.

The synonymous/non-synonymous ratio of changed bases in Strain 1 was 3.56. This ratio was generally about 3.3–3.6 in all the discovered strains, except for Strain 3, which had a ratio of 4.0. This may indicate positive selection occurring in the host environment for the FMDV. The synonymity information of the three strains can be found in Tables S5–S7 in the Supplementary. Here, we only report the results of the first three discovered strains, as they provide a representative sample of all the strains discovered.

## 4. Discussion

A computational workflow was designed for within-host diversity identification of co-existing strains of RNA viruses within a sample. This method addresses the limitations of commonly used de Bruijn-graph-based de novo genome assemblers, such as MEGAHIT, by identifying low-frequency co-existing strains that are often overlooked during the merging edge process of these tools. We applied this workflow to a high-coverage sample SRR11092062, from which MEGAHIT had assembled the reference genome sequence of SARS-CoV-2, and successfully detected two other co-existing strains of SARS-CoV-2 apart from the WIV04 assembled by MEGAHIT. Similarly, in the wastewater sample SRR12596175, the workflow discovered two co-existing SARS-CoV-2 strains’ sequences apart from a dominant strain sequence assembled by MEGAHIT. Other than SARS-CoV-2 samples, our strain identification process was also able to detect within-host diversity of the FMDV from an animal sample ERR034193.

The strain identification process is more sensitive to within-host diversity than normal de Bruijn-graph-based assembler, as it works on established genome sequences along with the sequencing reads, rather than assembling a sequence from scratch. The pre-processing step of non-human read extraction and error correction ensures the reliability of the reads used for strain identification. The identification process examines every position, allowing for a better chance to spot local variant sites than working on k-mer-scale, like the de Bruijn-graph-based assemblers. However, frequency is still used to determine the reliability of a read, with reads that show up multiple times having a relatively low probability of containing errors. When the frequency of a read is one, the read is verified by examining whether the mismatched part of it also shows up in other samples collected along with SRR11092062, with a consecutive region of more than 50 bases. This is because co-existing strains may have been circulating in Wuhan in late 2019, and other samples have inferior sequencing qualities, making it more difficult to find the entire read that matches perfectly the reference genome sequence and the identified strains. The produced sequence can be supported by a set of perfectly matched reads that have no gaps among them, which means these reads can be concatenated together to form the sequence. Therefore, after strain identification, multiple reliable co-existing strains’ sequences were produced.

The discovered strains from Wuhan showed a more remote relationship with all the coronaviruses in the table than the original SARS-CoV-2 strain, which may indicate that these two co-existing strains are produced by mutations from the original strain, but they were not widely circulated and may have diminished after the initial successful control of the pandemic outbreak in Wuhan, China. The prediction result suggests that the transmissibility of both newly discovered strains is weaker than the original strain. The weaker binding affinities of the two discovered strains with ACE2 also indicate that the two discovered strains could be replaced by the SARS-CoV-2 reference strain, just like Delta replaced the reference strain and Omicron replaced Delta.

In the future, this workflow could be applied to other RNA virus samples sequenced by NGS because within-host diversity is a common phenomenon in RNA virus infection. It often signals mutation and, sometimes, superinfection. Identifying co-existing strains and revealing the key differences in important protein regions and the binding activities will allow researchers to better infer the evolution status and circulation strains’ information. The identified co-existing strains of viruses could help in designing more effective and future-proof vaccines by detecting the potential dominant strains. This is because, if within-host diversity comes from mutation, then the mutated strain could have stronger transmission capability, resulting in a gradual replacement of the existing strain. Designing mRNA vaccines based on the mutated strain’s sequence will allow a much quicker process to protect the public against the future dominant strain. If it is a result of superinfection, a comparison of protein binding affinity among these strains may also provide insights into the contagiousness of difference strains.

## 5. Conclusions

In this study, our method outperformed existing methods to detect reliable SARS-CoV-2 strains in two samples (SRR12596175 and SRR11092062), demonstrating superior sensitivity compared to Haploflow and MEGAHIT. Our workflow was able to detect co-existing strains supported by reads without gaps, whereas Haploflow only assembled the dominant strain and MEGAHIT struggled with strain identification. We also demonstrated the generalizability of our workflow for RNA virus samples using the FMDV sample ERR034193.

Our workflow involved a multi-step process, including the extraction of relevant reads, error correction, strain identification, phylogenetic analysis, and protein binding analysis. We employed a novel double-model error-correction model that produced a reliable read set for further analysis. The identified strains’ sequences were supported by reads in the sample, and some of the variant sites also appeared in other samples collected at the same location around the same date. The protein binding affinity was determined by predicting the spike protein structure with AlphaFold2 and computing the docking scores. The final results included not only identified co-existing strains’ sequences, but also their phylogenetic relationship with the original strain and the protein binding affinity differences.

Our findings revealed that the discovered strains from SRR11092062 formed more unstable complex structures with human ACE2, indicating a lower ability to infect than the original SARS-CoV-2 strain. These strains also did not share any mutations with other major variants of concern. The contagiousness of the two discovered strains from Wuhan was lower than the original reference strain, suggesting the possibility of these two strains being the sibling strains and later being replaced.

Furthermore, our strain-identification algorithm performed better than Haploflow in detecting co-existing strains in a wastewater sample from California. We detected two possible co-existing strains of SARS-CoV-2 in the sample, while Haploflow only managed to reproduce the MEGAHIT-assembled contig sequence. This demonstrates our method’s ability to compensate de Bruijn-graph-based de novo assemblers in identifying co-existing strains from a sequencing read library.

Overall, our method presents a reliable and efficient workflow for identifying co-existing SARS-CoV-2 strains in sequencing read libraries. Our findings have implications for understanding the evolution and transmission of the virus and could aid in the development of targeted therapeutic interventions.

## Figures and Tables

**Table 1:** HDOCK results for top-10 models of binding energy scores and RMSD for WIV04 (7CWL) interaction with ACE2 (6M1D).

Rank	1	2	3	4	5	6	7	8	9	10
Docking Score	−320.46	−287.91	−283.90	−282.99	−280.66	−276.94	−274.08	−270.94	−266.21	−265.61
Ligand RMSD (Å)	380.77	369.92	339.95	327.94	317.26	400.29	368.67	289.60	403.08	383.07

**Table 2:** HDOCK results for top-10 models of binding energy scores and RMSD for discovered Strain 1 spike protein 3D structure interaction with ACE2 (6M1D).

Rank	1	2	3	4	5	6	7	8	9	10
Docking Score	−298.65	−278.85	−274.11	−264.91	−263.87	−262.92	−260.56	−258.40	−257.71	−256.86
Ligand RMSD (Å)	346.57	292.14	308.84	381.99	393.60	374.32	289.67	343.61	248.65	358.10

**Table 3:** HDOCK results for top-10 models of binding energy scores and RMSD for discovered Strain 2 spike protein 3D structure interaction with ACE2 (6M1D).

Rank	1	2	3	4	5	6	7	8	9	10
Docking Score	−298.22	−283.19	−272.89	−268.42	−267.25	−258.48	−249.65	−247.00	−243.73	−242.99
Ligand RMSD (Å)	356.14	308.39	353.95	391.48	377.23	369.87	350.24	381.94	327.39	297.72

**Table 4:** Similarity comparison on the nucleotide sequences for the original SARS-CoV-2 strain (WIV04), two discovered strains from SRR11092062, the wastewater assembled contig, the MR discovered strains from the wastewater sample, and other related coronaviruses. The similarity is calculated as the length of the shared sub-sequence divided by the total length of the nucleotide sequence. WH means the sample SRR11092062 from Wuhan (the original SARS-CoV-2 reference genome sample), and MR means the sample from Marin wastewater SRR12596175. MR_strain_1 and MR_strain_2 are short for MR_discovered_strain_1 and MR_discovered_strain_2.

Virus Name	SARS-CoV-2/WIV04	WH strain_1	WH strain_2	MR_contig	MR strain_1	MR strain_2
Bat_cov/RaTG13	0.961	0.954	0.957	0.959	0.943	0.959
Bat_cov/HKU2	0.514	0.512	0.514	0.513	0.51	0.512
SARS-CoV/BJ01	0.798	0.793	0.796	0.797	0.785	0.796
MERS-CoV	0.57	0.568	0.57	0.57	0.566	0.569
SARS-CoV-2/Kappa	0.996	0.989	0.993	0.995	0.977	0.994
SARS-CoV-2/Delta	0.996	0.988	0.992	0.994	0.977	0.994
SARS-CoV-2/Lambda	0.997	0.989	0.993	0.995	0.978	0.995
SARS-CoV-2/Gamma	0.985	0.978	0.982	0.984	0.966	0.983
SARS-CoV-2/Beta	0.997	0.991	0.994	0.996	0.979	0.996
SARS-CoV-2/Alpha	0.995	0.987	0.991	0.993	0.976	0.993
SARS-CoV-2/Omicron	0.998	0.99	0.994	0.996	0.979	0.996
Bat_cov/BANAL-20-52	0.967	0.961	0.964	0.966	0.949	0.965
Bat_cov/BANAL-20-236	0.959	0.952	0.956	0.957	0.941	0.957
SARS-CoV-2/WIV04	1.0	0.993	0.997	0.998	0.98	0.997
WH_discovered-strain_1	0.993	1.0	0.989	0.991	0.974	0.991
WH_discovered-strain_2	0.997	0.989	1.0	0.995	0.977	0.994
MR_megahit-contig.fa	0.998	0.991	0.995	1.0	0.982	0.999
MR_strain_1	0.98	0.974	0.977	0.982	1.0	0.981
MR_strain_2	0.997	0.991	0.994	0.999	0.981	1.0

**Table 5:** Similarity comparison on the (possible) spike protein sequences for the original SARS-CoV-2 strain (WIV04), the two discovered strains, the wastewater assembled contig, the MR discovered strains from the wastewater sample, and other related coronaviruses. The similarity is calculated as the length of the shared sub-sequence divided by the total length of the nucleotide sequence. WH means the sample SRR11092062 from Wuhan (the original SARS-CoV-2 reference genome sample), and MR means the sample from Marin wastewater SRR12596175. MR_strain_1 and MR_strain_2 are short for MR_discovered_strain_1 and MR_discovered_strain_2.

Virus Name	WIV04/s	WH strain_1	WH strain_2	MR_contig	MR strain_1	MR strain_2
Bat_CoV/RaTG13	0.974	0.963	0.971	0.966	0.477	0.965
Bat_CoV/HKU2	0.188	0.184	0.188	0.183	0.099	0.181
SARS-CoV/BJ01	0.764	0.754	0.763	0.756	0.331	0.756
MERS-CoV	0.327	0.323	0.327	0.326	0.199	0.326
SARS-CoV-2/Kappa	0.993	0.982	0.99	0.987	0.492	0.985
SARS-CoV-2/Delta	0.993	0.982	0.99	0.987	0.49	0.985
SARS-CoV-2/Lambda	0.989	0.978	0.986	0.983	0.483	0.981
SARS-CoV-2/Gamma	0.991	0.98	0.988	0.984	0.489	0.983
SARS-CoV-2/Beta	0.991	0.98	0.99	0.985	0.489	0.983
SARS-CoV-2/Alpha	0.992	0.981	0.989	0.986	0.491	0.984
SARS-CoV-2/Omicron	0.995	0.984	0.992	0.989	0.491	0.987
Bat_Cov/BANAL-20-52	0.984	0.973	0.981	0.976	0.486	0.975
Bat_Cov/BANAL-20-236	0.907	0.897	0.907	0.901	0.41	0.9
SARS-CoV-2/WIV04	1.0	0.989	0.997	0.992	0.495	0.991
WH_discovered-strain_1/s	0.989	1.0	0.986	0.981	0.494	0.98
WH_discovered-strain_2/s	0.997	0.986	1.0	0.989	0.493	0.987
MR_contig/s	0.992	0.981	0.989	1.0	0.505	0.998
MR_strain_1/s	0.498	0.498	0.496	0.505	1.0	0.503
MR_strain_2/s	0.991	0.98	0.988	0.998	0.503	1.0

## Data Availability

The nucleotide sequences and amino acid sequences used for phylogenetic analysis were downloaded from NCBI Genebank. The accession numbers are: MN996532.2 (RaTG13), EF203064.1 (HKU2), AT278488.2 (SARS-CoV), KC776174.1 (MERS-CoV), OM366054.1 (SARS-CoV-2 B.1.617.1), MZ724516.1 (SARS-CoV-2 B.1.617.2), MZ275302.1 (SARS-CoV-2 C.37), MW642248.1 (SARS-CoV-2 P.1), MZ068161.1 (SARS-CoV-2 B.1.351), OM616632.1 (SARS-CoV-2 B.1.1.7), OM766152.1 (SARS-CoV-2 BA.1), MN996528.1 (SARS-CoV-2 WIV04), MZ937000.1 (BANAL-20-52), MZ937003.2 (BANAL-20-236). The sequencing reads were downloaded from SRA [14] with accession numbers from SRR11092057 to SRR11092064 and SRR12596165-SRR12596175. The dataset SRR11092062 has the best quality and contains the most reads of SARS-CoV-2. It was chosen as the primary dataset in this work. SRR12596175 is the only sample we are able to identify with labeling from the wastewater sample paper.

## Supplementary Materials

The following are available at https://www.mdpi.com/article/10.3390/v15051065/s1. Supplementary data including tables and sequences are in the Supplementary Section. Table S1: Verified substituted bases from the discovered strain 1 from SRR11092062, a “-” sign means the base is deleted; Table S2: Verified substituted bases from discovered strain 2 of SRR11092062, a “-” sign means the base is deleted; Table S3: Verified substituted bases from the MR discovered strain 1, a “-” sign in original strain means the base is inserted in MR discovered strain 1, and the “-” sign in MR discovered strain 1 means the base is deleted; Table S4: Verified substituted bases from MR discovered train 2; Table S5: Substituted bases of strain 1 from FMDV sample. The consensus sequence doesn’t contain the first 350 bases in the reference genome sequence EU448639.1, so the positions are adjusted when determining the synonymity. The non-synonymous/synonymous ratio is 3.56. There are 178 changes in total. Table S6: Substituted bases of strain 2 from FMDV sample. The consensus sequence doesn’t contain the first 350 bases in the reference genome sequence EU448639.1, so the positions are adjusted when determining the synonymity. The non-synonymous/synonymous ratio is 3.68. There are 166 changes in total. Table S7: Substituted bases of strain 3 from FMDV sample. The consensus sequence doesn’t contain the first 350 bases in the reference genome sequence EU448639.1, so the positions are adjusted when determining the synonymity. The non-synonymous/synonymous ratio is 4.0. There are 170 changes in total; Figure S1: Difference of discovered strain 1 spike protein sequence and original strain spike protein sequence; Figure S2: Difference of discovered strain 2 spike protein sequence and original strain spike protein sequence; Figure S3: Difference of MR discovered strain 1 spike protein sequence and the MR assembled contig spike protein sequence; Figure S4: Difference of MR discovered strain 2 spike protein sequence and MR assembled contig spike protein sequence File S1: Sequences

## Sample Availability

See Data Availability.
