# Supplementary material for: Intra-Host Co-Existing Strains of SARS-CoV-2 Reference Genome Uncovered by Exhaustive Computational Search
Source: Viruses. 2023 Apr 26;15(5):1065. doi: 10.3390/v15051065 (PMC10224212; doi:10.3390/v15051065)
Supplement: Supplementary file 1 [file viruses-15-01065-s001.zip › viruses-2214872-supplementary.pdf]

1. Supplemental Material

1.1. Tables

Table S1. Verified substituted bases from the discovered strain 1 from SRR11092062, a "-" sign means the base is deleted.

Position	Bases in Reference Strain	Bases in WH Discovered Strain 1	Frequency	Segment Length	Change Status
348	T	C	1	156	non-synonymous
565	T	C	5	0	synonymous
1645	C	G	1	54	non-synonymous
1804	A	T	2	66	non-synonymous
2248	T	A	2	0	synonymous
2249	G	A	2	0	non-synonymous
2251	A	T	2	0	non-synonymous
2253	A	G	2	0	non-synonymous
2254	A	G	2	0	non-synonymous
2450	C	A	2	149	non-synonymous
2451	T	A	2	1	non-synonymous
2452	A	G	2	1	non-synonymous
2453	C	T	2	1	non-synonymous
2454	T	C	2	1	non-synonymous
2455	C	G	2	0	non-synonymous
2456	A	G	2	0	non-synonymous
2457	T	A	2	0	non-synonymous
2458	G	T	2	0	non-synonymous
2460	C	G	2	2	non-synonymous
2462	C	A	2	0	non-synonymous
2463	T	G	2	0	non-synonymous
3264	C	T	2	0	non-synonymous
3625	A	G	4	176	synonymous
3784	C	T	3	0	synonymous
3912	C	T	2	0	non-synonymous
4288	G	T	2	0	non-synonymous
5091	C	T	2	0	non-synonymous
6119	T	C	2	0	non-synonymous
6120	C	T	2	0	non-synonymous
6121	A	C	2	0	non-synonymous
6122	A	C	2	1	synonymous
6125	G	C	2	0	non-synonymous
6126	A	T	2	0	non-synonymous
6127	G	T	2	0	non-synonymous
6270	C	A	2	0	non-synonymous
6273	A	C	2	0	non-synonymous
6274	T	G	2	0	non-synonymous

Table S1. *Cont.*

Position	Bases in Reference Strain	Bases in WH Discovered Strain 1	Frequency	Segment Length	Change Status
6275	A	G	2	0	non-synonymous
6277	A	T	2	0	non-synonymous
7241	G	C	3	17	non-synonymous
7242	A	C	3	1	non-synonymous
7244	T	C	3	1	non-synonymous
7245	G	T	3	2	non-synonymous
7246	G	T	3	1	non-synonymous
7247	T	G	3	1	non-synonymous
7248	T	G	3	1	non-synonymous
7249	T	C	3	1	non-synonymous
7250	T	C	3	1	synonymous
7252	G	C	3	2	synonymous
7253	G	C	3	2	non-synonymous
7254	C	G	3	3	non-synonymous
7256	T	C	3	1	non-synonymous
7257	A	T	3	0	non-synonymous
7930	A	G	2	0	synonymous
7933	A	G	2	4	synonymous
7935	C	A	2	2	non-synonymous
7936	G	T	2	1	non-synonymous
7937	T	C	2	1	non-synonymous
7938	C	G	2	1	non-synonymous
8173	A	G	2	24	synonymous
8174	G	C	2	1	non-synonymous
8175	C	T	2	2	non-synonymous
8177	C	G	2	3	non-synonymous
8179	G	C	2	3	non-synonymous
8181	A	T	2	0	non-synonymous
8182	A	C	2	2	non-synonymous
8183	G	C	2	0	non-synonymous
8185	G	A	2	0	non-synonymous
8186	T	C	2	0	non-synonymous
9167	T	A	3	161	non-synonymous
10,009	T	A	2	0	synonymous
10,451	A	G	12	179	non-synonymous
10,933	T	-	2	0	non-synonymous
12506	A	-	2	0	non-synonymous
12,847	T	A	2	118	synonymous
13,529	T	C	2	0	non-synonymous
13,927	T	C	3	0	synonymous

Table S1. *Cont.*

Position	Bases in Reference Strain	Bases in WH Discovered Strain 1	Frequency	Segment Length	Change Status
15,060	T	C	2	0	non-synonymous
15,771	T	C	3	150	non-synonymous
15,957	G	A	2	137	non-synonymous
16,333	C	A	2	0	synonymous
16,335	T	G	2	0	non-synonymous
16,757	C	A	2	51	non-synonymous
16,759	C	G	2	2	non-synonymous
16,760	C	T	2	1	non-synonymous
16,761	A	C	2	1	non-synonymous
16,762	C	G	2	3	non-synonymous
16,763	T	G	2	1	non-synonymous
16,764	T	A	2	1	non-synonymous
16,765	A	T	2	1	non-synonymous
16,766	A	C	2	1	non-synonymous
16,767	C	G	2	0	non-synonymous
17,458	C	A	3	0	non-synonymous
17,459	C	G	3	1	non-synonymous
17,460	A	T	3	0	non-synonymous
17,650	T	G	2	175	synonymous
17,825	C	T	7	1	non-synonymous
18,022	A	G	3	0	synonymous
18,024	T	C	3	0	non-synonymous
18,025	G	T	3	0	non-synonymous
18,597	G	A	2	0	non-synonymous
19,050	T	A	2	9	non-synonymous
19,817	T	A	2	80	non-synonymous
19,819	C	G	2	0	non-synonymous
19,820	C	T	2	0	non-synonymous
19,821	A	C	2	1	non-synonymous
19,823	A	G	2	2	non-synonymous
19,824	G	A	2	0	non-synonymous
19,825	G	T	2	1	non-synonymous
19,826	T	C	2	0	non-synonymous
19,828	A	T	2	3	non-synonymous
19,830	A	G	2	0	non-synonymous
19,831	A	C	2	0	non-synonymous
19,832	T	C	2	3	non-synonymous
20,136	A	C	3	0	non-synonymous
21,587	C	T	2	0	non-synonymous
22,114	T	G	2	0	synonymous

Table S1. *Cont.*

Position	Bases in Reference Strain	Bases in WH Discovered Strain 1	Frequency	Segment Length	Change Status
22,274	T	C	2	0	non-synonymous
22,990	T	A	3	0	synonymous
23,364	G	A	2	0	non-synonymous
23,366	A	G	2	0	non-synonymous
23,367	C	T	2	0	non-synonymous
23,368	A	C	2	0	non-synonymous
23,369	A	G	2	0	non-synonymous
23,605	T	C	4	206	synonymous
23,859	C	G	1	56	non-synonymous
24,108	T	A	1	177	non-synonymous
24,259	T	A	1	155	synonymous
24,653	G	C	2	0	non-synonymous
24,654	A	T	2	1	non-synonymous
24,655	G	C	2	0	non-synonymous
24,656	T	C	2	0	non-synonymous
24,658	T	A	2	0	non-synonymous
24,659	G	C	2	0	non-synonymous
24,661	A	T	2	0	non-synonymous
24,804	T	A	2	0	non-synonymous
24,805	T	G	2	2	non-synonymous
24,807	G	C	2	2	non-synonymous
24,808	T	G	2	0	non-synonymous
24,809	C	G	2	0	non-synonymous
25,046	C	G	2	0	non-synonymous
25,518	T	C	2	3	synonymous
25,519	T	C	2	0	non-synonymous
25,522	G	C	2	3	non-synonymous
25,525	T	C	2	0	non-synonymous
25,526	G	T	2	1	non-synonymous
25,527	G	T	2	0	non-synonymous
25,669	C	A	2	1	non-synonymous
25,670	A	G	2	0	non-synonymous
25,671	C	T	2	0	non-synonymous
25,673	T	G	2	0	non-synonymous
25,674	T	G	2	0	non-synonymous
25,675	T	A	2	0	non-synonymous
25,677	G	C	2	2	non-synonymous
26,354	T	A	1	163	non-synonymous
26,836	G	T	2	40	non-synonymous
26,838	A	G	2	0	non-synonymous

Table S1. *Cont.*

Position	Bases in Reference Strain	Bases in WH Discovered Strain 1	Frequency	Segment Length	Change Status
26,839	C	G	2	1	non-synonymous
26,840	G	C	2	2	non-synonymous
26,842	G	T	2	2	non-synonymous
26,843	T	C	2	0	non-synonymous
26,844	T	C	2	1	non-synonymous
26,845	C	G	2	0	non-synonymous
26,846	C	A	2	1	non-synonymous
26,847	A	C	2	0	non-synonymous
26,849	G	T	2	5	non-synonymous
27,017	T	A	4	158	synonymous
27,372	A	G	3	0	synonymous
27,885	T	G	3	2	non-synonymous
28,293	A	G	3	151	non-synonymous
28,510	T	C	3	1	synonymous
28,777	A	C	2	0	synonymous
29,066	A	C	3	0	non-synonymous
29,456	C	T	3	0	non-synonymous
29,840	T	A	3	142	synonymous
29,843	G	C	3	3	synonymous
29,844	A	G	3	162	synonymous
29,845	T	G	3	162	synonymous
29,846	T	A	3	1	synonymous

Table S2. Verified substituted bases from discovered strain 2 of SRR11092062, a "-" sign means the base is deleted.

Position	Bases in Reference Strain	Bases in WH Discovered Strain 2	Frequency	Segment Length	Change Status
3625	A	C	3	153	synonymous
4291	A	T	2	0	synonymous
6160	T	A	2	0	synonymous
6161	G	A	2	0	non-synonymous
6162	A	G	2	0	non-synonymous
6163	T	A	2	11	non-synonymous
6164	G	C	2	11	non-synonymous
6165	T	C	2	11	non-synonymous
6167	G	C	2	11	non-synonymous
6169	G	T	2	11	non-synonymous
6171	C	G	2	1	non-synonymous
6172	T	C	2	11	non-synonymous

Table S2. *Cont.*

Position	Bases in Reference Strain	Bases in WH Discovered Strain 2	Frequency	Segment Length	Change Status
6173	A	C	2	2	non-synonymous
6175	T	C	2	3	non-synonymous
6176	G	C	2	3	non-synonymous
6177	A	G	2	3	non-synonymous
6178	T	A	2	9	non-synonymous
6179	T	C	2	4	non-synonymous
6180	A	T	2	3	non-synonymous
7305	T	A	2	159	non-synonymous
10,451	A	C	6	214	non-synonymous
13,392	G	T	2	56	non-synonymous
13,950	T	A	2	50	non-synonymous
13,951	A	G	2	0	non-synonymous
13,953	A	C	2	2	non-synonymous
13,954	T	G	2	1	non-synonymous
13,955	T	G	2	2	non-synonymous
13,957	C	T	2	0	non-synonymous
13,958	G	C	2	1	non-synonymous
13,959	C	G	2	1	non-synonymous
13,960	G	T	2	1	non-synonymous
13,961	T	A	2	0	non-synonymous
15,882	T	A	1	176	non-synonymous
16,469	C	A	2	129	non-synonymous
16,470	C	A	2	150	non-synonymous
16,471	A	G	2	150	non-synonymous
16,473	T	C	2	1	non-synonymous
16,474	A	G	2	1	non-synonymous
17,374	A	G	2	157	synonymous
17,709	A	G	4	233	non-synonymous
17,886	T	C	3	153	non-synonymous
18,108	A	G	2	1	non-synonymous
19,150	G	A	2	145	non-synonymous
19,151	C	A	2	1	non-synonymous
19,152	C	G	2	1	non-synonymous
19,153	A	T	2	2	non-synonymous
19,155	A	G	2	2	non-synonymous
19,156	C	G	2	2	non-synonymous
19,159	T	C	2	1	synonymous
19,160	C	G	2	2	non-synonymous
19,162	G	A	2	3	non-synonymous
19,163	A	G	2	3	non-synonymous

Table S2. *Cont.*

Position	Bases in Reference Strain	Bases in WH Discovered Strain 2	Frequency	Segment Length	Change Status
19,165	A	C	2	2	non-synonymous
19,167	A	T	2	1	non-synonymous
19,168	T	G	2	3	non-synonymous
19,171	A	G	2	4	synonymous
19,172	C	T	2	4	non-synonymous
19,173	A	T	2	1	non-synonymous
19,174	G	C	2	0	non-synonymous
19,175	A	T	2	1	non-synonymous
19,176	T	G	2	1	non-synonymous
19,177	G	T	2	0	non-synonymous
19,896	T	C	2	0	non-synonymous
21,486	T	A	2	0	non-synonymous
21,487	A	C	2	0	non-synonymous
21,488	A	T	2	3	non-synonymous
21,489	A	T	2	2	non-synonymous
21,638	C	A	2	143	non-synonymous
21,639	C	A	2	143	non-synonymous
21,640	T	G	2	143	non-synonymous
21,641	G	T	2	143	non-synonymous
21,643	A	G	2	143	non-synonymous
21,644	T	G	2	143	non-synonymous
22,114	T	C	2	160	synonymous
23,280	C	T	1	52	non-synonymous
23,281	T	C	1	12	non-synonymous
23,590	T	C	2	207	synonymous
28,240	T	C	2	1	non-synonymous
28,242	G	T	2	1	non-synonymous
28,243	T	G	2	1	non-synonymous
28,245	T	A	2	1	non-synonymous
28,246	T	G	2	2	non-synonymous
28,248	G	T	2	3	non-synonymous
28,249	A	C	2	1	non-synonymous
28,251	T	G	2	1	non-synonymous
28,253	C	T	2	2	non-synonymous
28,254	A	C	2	1	non-synonymous
29,177	C	T	2	0	non-synonymous
29,377	T	A	2	21	synonymous
29,542	A	G	2	139	synonymous
29,543	G	A	2	1	synonymous
29,544	A	C	2	2	synonymous

Table S2. *Cont.*

Position	Bases in Reference Strain	Bases in WH Discovered Strain 2	Frequency	Segment Length	Change Status
29,546	C	G	2	0	synonymous
29,547	A	C	2	1	synonymous
29,548	C	T	2	1	synonymous
29,549	A	T	2	0	synonymous
29,550	C	G	2	1	synonymous
29,551	A	G	2	1	synonymous
29,552	A	C	2	3	synonymous
29,553	G	C	2	0	synonymous
29,554	G	T	2	0	synonymous
29,556	A	C	2	0	synonymous
29,559	T	C	2	0	synonymous
29,560	G	T	2	0	synonymous
29,561	G	T	2	3	synonymous
29,724	C	A	2	50	synonymous
29,725	A	G	2	4	synonymous
29,727	T	C	2	2	synonymous
29,728	T	G	2	1	synonymous
29,729	T	G	2	0	synonymous
29,730	C	A	2	1	synonymous
29,731	A	T	2	2	synonymous
29,733	C	G	2	2	synonymous
29,734	G	T	2	1	synonymous
29,737	G	C	2	4	synonymous
29,739	C	A	2	2	synonymous
29,740	A	T	2	1	synonymous
29,741	C	G	2	1	synonymous
29,742	G	T	2	0	synonymous
29,745	G	T	2	2	synonymous

Table S**3.** Verified substituted bases from the MR discovered strain 1, a "-" sign in original strain means the base is inserted in MR discovered strain 1, and the "-" sign in MR discovered strain 1 means the base is deleted.

Position	Bases in Assembled Contig	Bases in MR Discovered Strain 1	Frequency	Segment Length	Change Type
289	G	T	4	0	unavailable
1413	T	C	17	129	non-synonymous
1659	A	C	2	0	non-synonymous
1766	T	G	20	77	synonymous
2938	C	G	1	58	non-synonymous
3270	A	G	27	117	non-synonymous

Table S3. *Cont.*

Position	Bases in Assembled Contig	Bases in MR Discovered Strain 1	Frequency	Segment Length	Change Type
5611	C	T	2	0	non-synonymous
6725	-	T	3	1	non-synonymous
7535	A	C	5	0	non-synonymous
7749	C	A	3	0	non-synonymous
8111	G	A	18	0	non-synonymous
8367	A	T	2	2	non-synonymous
9111	G	A	1	104	non-synonymous
10,347	C	T	25	0	non-synonymous
10,433	T	A	1	93	non-synonymous
10,925	A	T	4	0	synonymous
11,840	C	A	3	0	synonymous
11,944	T	C	20	103	non-synonymous
12,168	A	G	1	118	non-synonymous
13,328	-	A	2	4	non-synonymous
14,363	G	T	2	0	unavailable
14,833	C	T	16	0	unavailable
15,082	T	-	2	0	unavailable
15,795	T	G	2	51	unavailable
16,288	C	T	3	0	unavailable
16,592	G	T	3	6	unavailable
16,687	T	-	2	0	unavailable
17,542	T	C	4	0	unavailable
18,452	A	G	13	0	unavailable
18,803	C	A	1	88	unavailable
18,888	C	T	2	0	unavailable
19,867	T	A	1	88	unavailable
20,011	C	T	2	66	unavailable
20,145	C	G	2	0	unavailable
21,083	G	A	3	0	unavailable
21,332	C	T	23	0	unavailable
22,208	G	A	2	0	synonymous
22,946	G	A	2	0	synonymous
23,071	C	T	2	35	non-synonymous
23,259	A	-	2	0	non-synonymous
23,276	C	T	2	12	synonymous
24,597	A	T	2	0	non-synonymous
24,773	C	T	23	0	synonymous
25,220	A	-	3	0	non-synonymous
25,701	T	A	1	114	unavailable
25,736	C	T	3	3	unavailable

Table S3. *Cont.*

Position	Bases in Assembled Contig	Bases in MR Discovered Strain 1	Frequency	Segment Length	Change Type
25,935	G	T	20	0	unavailable
27,086	A	G	2	0	unavailable
27,379	T	A	1	106	unavailable
27,754	A	C	2	85	unavailable
27,755	C	T	2	1	unavailable
27,756	T	A	2	0	unavailable
27,898	G	T	3	7	unavailable
27,992	C	T	26	0	unavailable
28,115	T	C	14	140	unavailable
28,243	-	T	4	1	unavailable
28,500	C	T	19	0	unavailable
28,813	T	A	1	81	unavailable
28,897	C	T	37	0	unavailable
29,028	G	T	5	36	unavailable
29,080	-	A	5	57	unavailable
29,524	A	T	1	128	unavailable
29,609	T	C	3	9	unavailable

Table S4. Verified substituted bases from MR discovered train 2.

Position	Bases in Assembled Contig	Bases in MR Discovered Strain 2	Frequency	Segment Length	Change Type
1558	A	T	1	134	non-synonymous
5598	A	G	2	0	non-synonymous
7471	T	C	2	0	non-synonymous
10,724	T	A	1	163	non-synonymous
13,378	G	T	2	0	non-synonymous
15,099	T	A	3	0	unavailable
15,350	A	T	1	140	unavailable
15,855	A	T	1	133	unavailable
17,156	T	C	1	89	unavailable
17,483	G	A	1	138	unavailable
17,589	G	T	2	0	unavailable
18,476	A	G	2	0	unavailable
21,277	G	A	2	0	unavailable
21,622	T	A	1	105	non-synonymous
22,283	A	G	2	0	non-synonymous
27,778	G	A	2	10	unavailable
28,851	T	G	2	0	unavailable

Table S5. Substituted bases of strain 1 from FMDV sample. The consensus sequence doesn't contain the first 350 bases in the reference genome sequence EU448639.1, so the positions are adjusted when determining the synonymity. The non-synonymous/synonymous ratio is 3.56. There are 178 changes in total.

Position	Bases in Assembled Contig	Bases in FMDV Discovered Strain 1	Frequency	Change Type
64	A	C	2	unavailable
158	G	A	2	unavailable
233	T	C	3	unavailable
243	A	G	21	unavailable
299	C	A	2	unavailable
413	A	G	81	unavailable
426	T	G	2	unavailable
455	A	C	11	unavailable
538	T	G	10	unavailable
562	A	G	2	unavailable
593	A	G	2	unavailable
645	T	C	4	unavailable
767	T	A	20	non-synonymous
799	A	C	5	non-synonymous
853	T	C	13	non-synonymous
863	A	C	63	non-synonymous
942	C	T	5	synonymous
973	A	G	2	non-synonymous
1009	C	T	2	non-synonymous
1063	A	C	115	non-synonymous
1123	T	G	3	non-synonymous
1175	T	G	5	non-synonymous
1189	A	C	23	non-synonymous
1262	T	G	2	non-synonymous
1342	T	A	8	non-synonymous
1394	T	A	31	non-synonymous
1398	C	A	2	non-synonymous
1419	T	C	4	synonymous
1480	T	C	8	non-synonymous
1483	A	C	56	non-synonymous
1563	T	G	7	synonymous
1622	G	A	3	non-synonymous
1639	A	C	27	non-synonymous
1640	A	C	49	non-synonymous
1690	T	A	7	non-synonymous
1710	T	C	2	synonymous
1765	T	G	2	non-synonymous

Table S5. *Cont.*

Position	Bases in Assembled Contig	Bases in FMDV Discovered Strain 1	Frequency	Change Type
1820	A	C	49	non-synonymous
1901	A	G	53	non-synonymous
1912	A	C	33	non-synonymous
1984	A	G	27	non-synonymous
2034	A	G	2	synonymous
2081	A	C	44	non-synonymous
2135	T	G	130	non-synonymous
2142	T	C	26	synonymous
2157	T	C	4	synonymous
2220	T	G	3	synonymous
2282	T	G	116	non-synonymous
2312	T	C	2	non-synonymous
2330	A	C	15	non-synonymous
2405	G	A	453	non-synonymous
2417	A	G	528	non-synonymous
2468	T	C	2	non-synonymous
2486	C	T	2	non-synonymous
2539	T	A	4	non-synonymous
2605	A	G	81	non-synonymous
2689	T	G	9	non-synonymous
2714	T	C	49	non-synonymous
2720	A	C	96	non-synonymous
2788	A	G	808	non-synonymous
2856	T	G	102	synonymous
2872	A	G	109	non-synonymous
2932	A	C	303	non-synonymous
2993	T	C	3	non-synonymous
3046	A	G	87	non-synonymous
3103	T	C	4	non-synonymous
3120	T	C	2	synonymous
3199	A	C	19	non-synonymous
3266	A	C	128	non-synonymous
3320	T	G	2	non-synonymous
3338	A	G	5	non-synonymous
3400	A	G	163	non-synonymous
3475	G	A	2	non-synonymous
3524	T	C	119	non-synonymous
3564	A	G	92	synonymous
3644	A	C	32	non-synonymous

Table S5. *Cont.*

Position	Bases in Assembled Contig	Bases in FMDV Discovered Strain 1	Frequency	Change Type
3645	G	C	18	non-synonymous
3671	A	C	3	non-synonymous
3725	T	G	72	non-synonymous
3759	A	C	1302	non-synonymous
3848	T	A	524	non-synonymous
3886	A	C	23	non-synonymous
3947	T	C	8	non-synonymous
3963	A	C	11	synonymous
4037	C	T	10	non-synonymous
4056	A	C	58	synonymous
4106	T	G	2390	non-synonymous
4169	T	G	66	non-synonymous
4170	C	G	39	non-synonymous
4171	A	C	8	non-synonymous
4223	A	C	12	non-synonymous
4276	T	G	421	non-synonymous
4292	A	C	384	non-synonymous
4381	T	A	237	non-synonymous
4419	A	G	3	synonymous
4441	A	C	123	non-synonymous
4450	A	C	706	non-synonymous
4510	A	C	230	non-synonymous
4565	A	T	6	non-synonymous
4629	T	A	49	synonymous
4663	A	C	33	non-synonymous
4735	G	T	3226	non-synonymous
4788	C	T	2709	synonymous
4868	T	G	670	non-synonymous
4896	G	T	282	non-synonymous
4946	T	G	124	non-synonymous
4983	C	T	5	synonymous
5033	A	T	3	non-synonymous
5085	C	T	28	synonymous
5118	A	C	6	non-synonymous
5177	T	C	5	non-synonymous
5228	T	C	3	non-synonymous
5278	T	G	5	non-synonymous
5299	A	C	4	non-synonymous
5394	T	C	28	synonymous
5419	T	C	22	non-synonymous

Table S5. *Cont.*

Position	Bases in Assembled Contig	Bases in FMDV Discovered Strain 1	Frequency	Change Type
5469	C	T	2	synonymous
5478	A	C	64	synonymous
5544	T	C	4	synonymous
5552	A	G	3	non-synonymous
5621	T	G	12	non-synonymous
5624	A	G	2	non-synonymous
5625	G	A	6	synonymous
5679	T	G	8	synonymous
5720	C	A	664	non-synonymous
5780	T	G	8	non-synonymous
5832	C	A	3	non-synonymous
5846	A	C	6	non-synonymous
5851	A	G	44	non-synonymous
5860	A	C	308	non-synonymous
5922	A	T	4	synonymous
5923	G	T	4	non-synonymous
5945	A	C	36	non-synonymous
5996	G	A	8	non-synonymous
6010	A	C	24	non-synonymous
6066	T	G	16	synonymous
6112	A	C	169	non-synonymous
6165	A	G	553	synonymous
6236	T	G	8	non-synonymous
6270	T	C	2	synonymous
6338	T	G	9	non-synonymous
6396	T	G	43	synonymous
6399	G	T	51	synonymous
6400	T	A	78	non-synonymous
6459	T	G	130	synonymous
6479	T	A	24	non-synonymous
6543	T	G	22	non-synonymous
6561	A	C	70	synonymous
6624	T	G	14	synonymous
6629	A	G	10	non-synonymous
6680	T	C	50	non-synonymous
6683	A	G	4	non-synonymous
6740	T	G	226	non-synonymous
6760	A	C	2	non-synonymous
6793	A	C	71	non-synonymous
6852	T	C	3	synonymous

Table S5. *Cont.*

Position	Bases in Assembled Contig	Bases in FMDV Discovered Strain 1	Frequency	Change Type
6857	A	G	21	non-synonymous
6930	C	A	4	non-synonymous
6953	C	T	4	non-synonymous
7004	T	C	5839	non-synonymous
7091	T	G	136	non-synonymous
7148	T	G	17	non-synonymous
7197	A	G	324	synonymous
7254	C	A	5	non-synonymous
7262	A	C	56	non-synonymous
7344	T	G	49	non-synonymous
7350	T	C	125	synonymous
7401	C	A	3	synonymous
7405	A	C	16	non-synonymous
7461	C	A	2	synonymous
7478	A	C	187	non-synonymous
7531	A	C	30	non-synonymous
7612	T	C	110	non-synonymous
7671	A	G	3	synonymous
7708	G	T	2	non-synonymous
7777	T	G	52	unavailable
7828	-	A	33	unavailable
7829	-	A	12	unavailable

Table S6. Substituted bases of strain 2 from FMDV sample. The consensus sequence doesn't contain the first 350 bases in the reference genome sequence EU448639.1, so the positions are adjusted when determining the synonymity. The non-synonymous/synonymous ratio is 3.68. There are 166 changes in total.

Position	Bases in Assembled Contig	Bases in FMDV Discovered Strain 2	Frequency	Change Type
93	A	C	2	unavailable
165	A	G	2	unavailable
188	G	A	2	unavailable
225	A	G	2	unavailable
258	A	C	20	unavailable
323	A	G	2	unavailable
394	T	G	203	unavailable
413	A	G	25	unavailable
473	T	C	10	unavailable
495	G	A	3	unavailable

Table S6. *Cont.*

Position	Bases in Assembled Contig	Bases in FMDV Discovered Strain 2	Frequency	Change Type
548	A	G	2	unavailable
607	A	G	2	unavailable
768	C	A	4	synonymous
804	A	G	37	synonymous
853	T	C	24	non-synonymous
910	T	C	2	non-synonymous
936	G	A	2	non-synonymous
974	T	C	2	non-synonymous
1026	T	C	2	synonymous
1063	A	C	4	non-synonymous
1075	A	C	34	non-synonymous
1131	T	C	2	synonymous
1201	T	G	13	non-synonymous
1229	A	T	2	non-synonymous
1347	T	C	5	synonymous
1402	T	A	18	non-synonymous
1440	G	A	6	synonymous
1495	T	C	9	non-synonymous
1498	A	C	17	non-synonymous
1575	T	C	2	synonymous
1603	A	C	3	non-synonymous
1654	T	C	10	non-synonymous
1693	G	A	2	non-synonymous
1795	A	C	9	non-synonymous
1865	T	C	10	non-synonymous
1867	A	C	8	non-synonymous
1919	T	G	130	non-synonymous
1942	T	G	2	non-synonymous
1985	A	C	2	non-synonymous
2067	C	T	55	synonymous
2142	T	C	35	synonymous
2163	T	G	2	synonymous
2214	C	T	2	synonymous
2274	T	G	49	synonymous
2338	C	G	10	non-synonymous
2339	G	C	4	non-synonymous
2404	C	T	298	non-synonymous
2417	A	G	237	non-synonymous
2456	T	G	5	non-synonymous
2492	A	G	2	non-synonymous

Table S6. *Cont.*

Position	Bases in Assembled Contig	Bases in FMDV Discovered Strain 2	Frequency	Change Type
2522	C	T	3	non-synonymous
2558	T	C	2	non-synonymous
2577	A	G	3	synonymous
2662	T	C	13	non-synonymous
2706	T	C	2	synonymous
2746	A	C	73	non-synonymous
2799	T	A	2	non-synonymous
2841	A	T	15	synonymous
2892	T	G	17	synonymous
2930	T	C	54	non-synonymous
2932	A	C	54	non-synonymous
3023	T	G	3	non-synonymous
3063	G	A	3	synonymous
3107	C	T	2	non-synonymous
3157	A	C	8	non-synonymous
3235	A	C	26	non-synonymous
3294	T	G	5	synonymous
3296	A	G	2	non-synonymous
3297	G	A	2	non-synonymous
3362	T	C	3	non-synonymous
3409	A	G	6	non-synonymous
3482	A	C	4	non-synonymous
3557	T	C	125	non-synonymous
3566	A	G	5	non-synonymous
3617	T	C	18	non-synonymous
3668	A	G	3	non-synonymous
3680	A	G	3	non-synonymous
3744	T	G	92	synonymous
3768	C	A	384	non-synonymous
3867	T	G	357	synonymous
3895	G	T	4	non-synonymous
3968	T	C	9	non-synonymous
3972	G	T	6	synonymous
4057	C	A	7	non-synonymous
4106	T	G	853	non-synonymous
4111	C	A	853	non-synonymous
4171	A	C	8	non-synonymous
4172	C	T	2	non-synonymous
4177	A	G	5	non-synonymous
4234	T	A	16	non-synonymous

Table S6. *Cont.*

Position	Bases in Assembled Contig	Bases in FMDV Discovered Strain 2	Frequency	Change Type
4250	A	G	101	non-synonymous
4310	T	C	21	non-synonymous
4364	T	G	138	non-synonymous
4381	T	A	12	non-synonymous
4419	A	C	53	synonymous
4478	A	C	185	non-synonymous
4549	G	A	18	non-synonymous
4594	T	C	10	non-synonymous
4649	T	A	7	non-synonymous
4684	A	C	38	non-synonymous
4735	G	T	140	non-synonymous
4762	T	G	140	non-synonymous
4807	A	C	7	non-synonymous
4868	T	G	250	non-synonymous
4871	A	G	250	non-synonymous
4899	C	A	545	non-synonymous
4958	T	G	22	non-synonymous
4984	C	T	3	non-synonymous
5066	C	T	3	non-synonymous
5115	C	T	7	synonymous
5172	A	G	7	synonymous
5258	T	G	4	non-synonymous
5325	G	A	4	synonymous
5335	A	G	2	non-synonymous
5394	T	G	12	synonymous
5423	A	G	10	non-synonymous
5475	A	C	42	synonymous
5528	A	G	7	non-synonymous
5591	T	C	15	non-synonymous
5604	A	C	7	synonymous
5676	T	G	3	non-synonymous
5685	A	C	82	synonymous
5744	A	G	24	non-synonymous
5801	T	C	4	non-synonymous
5843	T	G	7	non-synonymous
5851	A	G	90	non-synonymous
5907	A	G	5	synonymous
5947	C	T	8	non-synonymous
6026	T	G	12	non-synonymous
6099	T	C	326	synonymous

Table S6. *Cont.*

Position	Bases in Assembled Contig	Bases in FMDV Discovered Strain 2	Frequency	Change Type
6173	T	G	117	non-synonymous
6185	A	C	16	non-synonymous
6243	C	A	8	non-synonymous
6252	C	T	2	synonymous
6298	G	A	3	non-synonymous
6328	A	T	9	non-synonymous
6398	T	G	22	non-synonymous
6408	C	G	30	synonymous
6409	G	A	72	non-synonymous
6459	T	C	53	synonymous
6500	A	G	24	non-synonymous
6559	T	C	16	non-synonymous
6569	A	C	54	non-synonymous
6668	T	C	60	non-synonymous
6752	T	C	229	non-synonymous
6793	A	G	35	non-synonymous
6844	A	G	26	non-synonymous
6896	T	C	10	non-synonymous
6960	T	G	88	synonymous
6994	A	G	10	non-synonymous
7031	A	G	10	non-synonymous
7129	T	G	91	non-synonymous
7197	A	G	6	synonymous
7248	C	A	4	non-synonymous
7253	A	C	29	non-synonymous
7350	T	C	1116	synonymous
7399	A	C	60	non-synonymous
7445	A	C	59	non-synonymous
7499	C	A	3	non-synonymous
7512	A	C	23	non-synonymous
7607	T	C	131	non-synonymous
7652	A	G	2	non-synonymous
7695	T	C	2	synonymous
7726	T	C	4	unavailable
7784	T	C	200	unavailable
7790	G	T	180	unavailable

Table S7. Substituted bases of strain 3 from FMDV sample. The consensus sequence doesn't contain the first 350 bases in the reference genome sequence EU448639.1, so the positions are adjusted when determining the synonymity. The non-synonymous/synonymous ratio is 4.0. There are 170 changes in total.

Position	Bases in Assembled Contig	Bases in FMDV Discovered Strain 3	Frequency	Change Type
246	A	C	15	unavailable
376	A	G	2	unavailable
436	T	C	15	unavailable
451	A	C	19	unavailable
521	T	G	5	unavailable
588	C	T	2	unavailable
649	A	G	2	unavailable
727	A	G	2	non-synonymous
785	T	C	4	non-synonymous
792	A	C	8	synonymous
844	A	C	23	non-synonymous
918	A	G	2	synonymous
961	A	G	4	non-synonymous
1030	C	T	2	non-synonymous
1061	A	C	16	non-synonymous
1113	T	G	2	synonymous
1149	T	C	2	synonymous
1204	T	G	4	non-synonymous
1253	A	G	4	non-synonymous
1319	G	T	2	non-synonymous
1372	T	A	18	non-synonymous
1409	T	C	2	non-synonymous
1461	T	G	5	non-synonymous
1489	A	C	12	non-synonymous
1543	G	A	2	non-synonymous
1606	A	C	3	non-synonymous
1648	A	C	9	non-synonymous
1778	A	C	5	non-synonymous
1830	A	C	4	non-synonymous
1878	T	C	138	synonymous
1942	T	G	34	non-synonymous
2016	T	C	2	synonymous
2051	C	T	2	non-synonymous
2071	G	T	14	non-synonymous
2132	T	G	4	non-synonymous
2220	T	C	4	synonymous
2279	T	C	15	non-synonymous

Table S7. *Cont.*

Position	Bases in Assembled Contig	Bases in FMDV Discovered Strain 3	Frequency	Change Type
2331	C	-	58	non-synonymous
2398	T	G	9	non-synonymous
2456	T	G	11	non-synonymous
2503	A	C	2	non-synonymous
2552	T	C	4	non-synonymous
2581	A	C	3	non-synonymous
2643	A	C	10	synonymous
2706	T	C	2	synonymous
2714	T	C	5	non-synonymous
2720	A	C	13	non-synonymous
2725	T	C	42	non-synonymous
2850	T	G	26	non-synonymous
2856	T	G	3	synonymous
2867	A	G	12	non-synonymous
2924	A	T	8	non-synonymous
2932	A	C	8	non-synonymous
2976	G	A	2	synonymous
3029	C	A	3	non-synonymous
3061	C	T	2	non-synonymous
3122	A	G	2	non-synonymous
3155	T	C	2	non-synonymous
3202	A	C	3	non-synonymous
3256	A	C	12	non-synonymous
3319	G	A	2	non-synonymous
3350	T	C	2	non-synonymous
3401	T	C	4	non-synonymous
3433	C	A	3	synonymous
3509	A	G	110	non-synonymous
3559	G	A	8	non-synonymous
3560	C	T	2	non-synonymous
3573	G	T	2	non-synonymous
3626	T	A	10	non-synonymous
3627	G	T	2	synonymous
3678	T	C	3	synonymous
3716	C	T	2	non-synonymous
3766	T	A	10	non-synonymous
3767	A	C	37	non-synonymous
3823	T	G	275	non-synonymous
3836	T	G	13	non-synonymous
3839	T	C	2	non-synonymous

Table S7. *Cont.*

Position	Bases in Assembled Contig	Bases in FMDV Discovered Strain 3	Frequency	Change Type
3889	T	C	13	non-synonymous
3936	G	A	4	synonymous
3990	A	G	85	synonymous
4059	T	G	7	synonymous
4106	T	G	51	non-synonymous
4111	C	A	51	non-synonymous
4112	T	A	51	non-synonymous
4114	A	T	12	non-synonymous
4187	T	G	11	non-synonymous
4258	G	C	575	non-synonymous
4287	C	G	375	non-synonymous
4373	G	C	53	non-synonymous
4381	T	A	6	non-synonymous
4389	G	T	3	non-synonymous
4441	A	C	428	non-synonymous
4494	C	A	3	non-synonymous
4510	A	G	83	non-synonymous
4571	C	T	3	non-synonymous
4614	A	G	7	synonymous
4660	A	C	16	non-synonymous
4726	T	G	274	non-synonymous
4785	C	A	5	non-synonymous
4797	A	C	8	synonymous
4868	T	G	32	non-synonymous
4869	T	G	32	non-synonymous
4908	A	C	139	non-synonymous
4976	T	G	12	non-synonymous
5015	C	T	2	non-synonymous
5076	C	A	3	non-synonymous
5085	C	T	8	synonymous
5137	T	C	6	non-synonymous
5181	G	T	3	non-synonymous
5265	T	C	4	synonymous
5316	T	C	3	synonymous
5362	A	C	10	non-synonymous
5412	C	A	3	non-synonymous
5417	A	C	2	non-synonymous
5463	A	C	39	synonymous
5520	A	C	4	synonymous
5584	G	A	4	non-synonymous

Table S7. *Cont.*

Position	Bases in Assembled Contig	Bases in FMDV Discovered Strain 3	Frequency	Change Type
5612	A	G	4	non-synonymous
5668	A	G	4	non-synonymous
5689	A	C	38	non-synonymous
5745	C	G	22	non-synonymous
5808	T	C	2	synonymous
5810	A	C	23	non-synonymous
5860	A	C	76	non-synonymous
5904	A	G	2	synonymous
5924	A	G	7	non-synonymous
6009	C	T	57	synonymous
6064	G	C	3	non-synonymous
6081	C	G	4	non-synonymous
6082	A	G	4	non-synonymous
6145	A	C	100	non-synonymous
6196	A	C	5	non-synonymous
6252	C	T	13	synonymous
6325	G	A	3	non-synonymous
6344	A	G	2	non-synonymous
6396	T	C	8	synonymous
6404	A	C	49	non-synonymous
6467	T	C	14	non-synonymous
6541	T	C	6	non-synonymous
6554	A	C	48	non-synonymous
6604	A	C	2	non-synonymous
6654	T	A	5	non-synonymous
6657	A	C	26	synonymous
6707	T	C	14	non-synonymous
6758	T	C	87	non-synonymous
6830	T	A	6	non-synonymous
6831	A	G	13	synonymous
6884	A	G	16	non-synonymous
6956	T	G	14	non-synonymous
6998	A	C	5	non-synonymous
7056	C	A	3	non-synonymous
7085	A	G	8	non-synonymous
7136	T	C	27	non-synonymous
7163	C	T	5	non-synonymous
7227	C	A	4	synonymous
7262	A	C	31	non-synonymous
7306	A	C	9	non-synonymous

Table S7. *Cont.*

Position	Bases in Assembled Contig	Bases in FMDV Discovered Strain 3	Frequency	Change Type
7370	T	C	15	non-synonymous
7379	A	C	16	non-synonymous
7432	T	C	3	non-synonymous
7462	A	C	27	non-synonymous
7512	A	G	6	synonymous
7563	T	G	91	synonymous
7627	T	A	54	non-synonymous
7648	T	C	2	non-synonymous
7685	T	C	2	non-synonymous
7733	G	C	2	unavailable
7734	A	G	2	unavailable
7783	C	T	267	unavailable
7784	T	C	267	unavailable

1.2. Sequences

1.3. SRR11092062 Discovered Strain 1 Spike Protein Sequence

1 MFVFLVLLSLVSSQCVNLTT RTQLPPAYTNSFTRGVYYPD KVFRSSVLHSTQDLFLPFFS NVTWFHAIHVSGTNGTKRFD NPVLPFNDGVYFASTEKSNI 100

101 IRGWIFGTTLDSKTQSLLIV NNATNVVIKVCEFQFCNDPF LGVYYHKNNKSWMESEFRVY SSANNCTFEYVSQPFLMDLE GKQGNFKNLREFVFKNIDGY 200

201 FKIYSKHTPINLVRDLPQGF SALEPLVDLPIGINITRLQT LLALHRSYLTPGDSSSGWTA GAAAYYVGYLQPRTFLLKYN ENGTITDAVDCALDPLSETK 300

301 CTLKSFTVEKGIYQTSNFRV QPTESIVRFPNITNLCPFGE VFNATRFASVYAWNRKRISN CVADYSVLYNSASFSTFKCY GVSPTKLNDLCFTNVYADSF 400

401 VIRGDEVRQIAPGQTGKIAD YNYKLPDDFTGCVIAWNSNN LDSKVGGNYNYLYRLFRKSN LKPFERDISTEIYQAGSTPC NGVEGFNCYFPLQSYGFQPT 500

501 NGVGYQPYRVVVLSFELLHA PATVCGPKKSTNLVKNKCVN FNFNGLTGTGVLTESNKKFL PFQQFGRDIADTTDAVRDPQ TLEILDITPCSFGGVSVITP 600

601 EVDTSNQVAVLYQDVNCTEV PVAIHADQLTPTWRVYSTGS NVFQTRAGCLIGAEHVNNSY ECDIPIGAGICASYQTQTNS PRRARSVASQSIIAYTMSLG 700

701 AENSVAYSNNSIAIPTNFTI SVTTEILPVSMTKTSVDCTM YICGDSTECSNLLLQYGSFC TQLNRGLTGIAVEQDKNTQE VFAQVKQIYKTPPIKDFGGF 800

801 NFSQILPDPSKPSKRSFIED LLFNKVTLADAGFIKQYGDC LGDIAARDHICAQKFNGLTV LPPLLTDEMIAQYTSALLAG TITSGWTFGAGAALQIPFAM 900

901 QMAYRFNGIGVTQNVLYENQ KLIANQFNSAIGKIQDSLSS TASALGKLQDVVNQNAQALN TLVKQLSSNFGAISSVLNDI LSRLDKVEAEVQIDRLITGR 1000

1001 LQSLQTYVTQQLIRAAEIRA SANLAATKMSLRLLGQSKRV DFCGKGYHLMSFPQSAPHGV VFLHVTYVPAQEKNFTTAPA KSDDGKAHFPREGVFVSNGT 1100

1101 HWFVTQRNFYEPQIITTDNT FVSGNCDVVIGIVNNTVYDP LQPELDSFKEELDKYFKNHT SADVDLGDISGINASVVNIQ KEIDRLNEVAKNLNESLIDL 1200

1201 QELGKYEQYIKWPWYIWLGF IAGLIAIVMVTIMLCCMTSC CSCLKGCCSCGSCCKFDEDD SEPVLKGVKLHYT SEPVLKGVKLHYT 1273

1.4. Difference of SRR11092062 Discovered Strain 1 Spike Protein Sequence and SARS-CoV-2 Spike Protein Sequence

The format of EMBOSS Needle is that each three lines followed an empty line, the first in the three lines is 50 amino acid bases of SARS-CoV-2 original strain spike protein, the second line is the matching status, and the third line is 50 amino acid bases of discovered strain 1. | means the two bases at the position in the first protein sequence and the second protein sequence are the same, other signs mean the two bases are different. The figure is located at 1 and 1.

1.5. SRR11092062 Discovered Strain 2 Spike Protein Sequence

1 MFVFLVLLPLVSSQCVNLTT RTQLPKSDTNSFTRGVYYPD KVFRSSVLHSTQDLFLPFFS NVTWFHAIHVSGTNGTKRFD NPVLPFNDGVYFASTEKSNI 100

101 IRGWIFGTTLDSKTQSLLIV NNATNVVIKVCEFQFCNDPF LGVYYHKNNKSWMESEFRVY SSANNCTFEYVSQPFLMDLE GKQGNFKNLREFVFKNIDGY 200

201 FKIYSKHTPINLVRDLPQGF SALEPLVDLPIGINITRFQT LLALHRSYLTPGDSSSGWTA GAAAYYVGYLQPRTFLLKYN ENGTITDAVDCALDPLSETK 300

301 CTLKSFTVEKGIYQTSNFRV QPTESIVRFPNITNLCPFGE VFNATRFASVYAWNRKRISN CVADYSVLYNSASFSTFKCY GVSPTKLNDLCFTNVYADSF 400

401 VIRGDEVRQIAPGQTGKIAD YNYKLPDDFTGCVIAWNSNN LDSKVGGNYNYLYRLFRKSN LKPFERDISTEIYQAGSTPC NGVEGFNCYFPLQSYGFQPT 500

501 NGVGYQPYRVVVLSFELLHA PATVCGPKKSTNLVKNKCVN FNFNGLTGTGVLTESNKKFL PFQQFGRDIADTIDAVRDPQ TLEILDITPCSFGGVSVITP 600

601 GTNTSNQVAVLYQDVNCTEV PVAIHADQLTPTWRVYSTGS NVFQTRAGCLIGAEHVNNSY ECDIPIGAGICASYQTQTNS PRRARSVASQSIIAYTMSLG 700

701 AENSVAYSNNSIAIPTNFTI SVTTEILPVSMTKTSVDCTM YICGDSTECSNLLLQYGSFC TQLNRALTGIAVEQDKNTQE VFAQVKQIYKTPPIKDFGGF 800

801 NFSQILPDPSKPSKRSFIED LLFNKVTLADAGFIKQYGDC LGDIAARDLICAQKFNGLTV LPPLLTDEMIAQYTSALLAG TITSGWTFGAGAALQIPFAM 900

901 QMAYRFNGIGVTQNVLYENQ KLIANQFNSAIGKIQDSLSS TASALGKLQDVVNQNAQALN TLVKQLSSNFGAISSVLNDI LSRLDKVEAEVQIDRLITGR 1000

1001 LQSLQTYVTQQLIRAAEIRA SANLAATKMSECVLGQSKRV DFCGKGYHLMSFPQSAPHGV VFLHVTYVPAQEKNFTTAPA ICHDGKAHFPREGVFVSNGT 1100

1101 HWFVTQRNFYEPQIITTDNT FVSGNCDVVIGIVNNTVYDP LQPELDSFKEELDKYFKNHT SPDVDLGDISGINASVVNIQ KEIDRLNEVAKNLNESLIDL 1200

1201 QELGKYEQYIKWPWYIWLGF IAGLIAIVMVTIMLCCMTSC CSCLKGCCSCGSCCKFDEDD SEPVLKGVKLHYT 1273

1.6. Difference of Discovered Strain 2 Spike Protein Sequence and SARS-CoV-2 Spike Protein Sequence

The format of EMBOSS Needle is that each three lines followed an empty line, the first in the three lines is 50 amino acid bases of SARS-CoV-2 original strain spike protein, the second line is the matching status, and the third line is 50 amino acid bases of discovered strain 2. | means the two bases at the position in the first protein sequence and the second protein sequence are the same, other signs mean the two bases are different.The figure is located at 2 and 2.

1.7. SRR11092062 Discovered Strain 1 Nucleotide Sequence

1 ATTAAAGGTTTATACCTTCC CAGGTAACAAACCAACCAAC TTTCGATCTCTTGTAGATCT GTTCTCTAAACGAACTTTAA AATCTGTGTGGCTGTCACTC 100
101 GGCTGCATGCTTAGTGCACT CACGCAGTATAATTAATAAC TAATTACTGTCGTTGACAGG ACACGAGTAACTCGTCTATC TTCTGCAGGCTGCTTACGGT 200
201 TTCGTCCGTGTTGCAGCCGA TCATCAGCACATCTAGGTTT CGTCCGGGTGTGACCGAAAG GTAAGATGGAGAGCCTTGTC CCTGGTTTCAACGAGAAAAC 300
301 ACACGTCCAACTCAGTTTGC CTGTTTTACAGGTTCGCGAC GTGCTCGCACGTGGCTTTGG AGACTCCGTGGAGGAGGTCT TATCAGAGGCACGTCAACAT 400
401 CTTAAAGATGGCACTTGTGG CTTAGTAGAAGTTGAAAAAG GCGTTTTGCCTCAACTTGAA CAGCCCTATGTGTTCATCAA ACGTTCGGATGCTCGAACTG 500
501 CACCTCATGGTCATGTTATG GTTGAGCTGGTAGCAGAACT CGAAGGCATTCAGTACGGTC GTAGCGGTGAGACACTTGGT GTCCTTGTCCCTCATGTGGG 600
601 CGAAATACCAGTGGCTTACC GCAAGGTTCTTCTTCGTAAG AACGGTAATAAAGGAGCTGG TGGCCATAGTTACGGCGCCG ATCTAAAGTCATTTGACTTA 700
701 GGCGACGAGCTTGGCACTGA TCCTTATGAAGATTTTCAAG AAAACTGGAACACTAAACAT AGCAGTGGTGTTACCCGTGA ACTCATGCGTGAGCTTAACG 800
801 GAGGGGCATACACTCGCTAT GTCGATAACAACTTCTGTGG CCCTGATGGCTACCCTCTTG AGTGCATTAAAGACCTTCTA GCACGTGCTGGTAAAGCTTC 900
901 ATGCACTTTGTCCGAACAAC TGGACTTTATTGACACTAAG AGGGGTGTATACTGCTGCCG TGAACATGAGCATGAAATTG CTTGGTACACGGAACGTTCT 1000
1001 GAAAAGAGCTATGAATTGCA GACACCTTTTGAAATTAAAT TGGCAAAGAAATTTGACACC TTCAATGGGGAATGTCCAAA TTTTGTATTTCCCTTAAATT 1100
1101 CCATAATCAAGACTATTCAA CCAAGGGTTGAAAAGAAAAA GCTTGATGGCTTTATGGGTA GAATTCGATCTGTCTATCCA GTTGCGTCACCAAATGAATG 1200
1201 CAACCAAATGTGCCTTTCAA CTCTCATGAAGTGTGATCAT TGTGGTGAAACTTCATGGCA GACGGGCGATTTTGTTAAAG CCACTTGCGAATTTTGTGGC 1300
1301 ACTGAGAATTTGACTAAAGA AGGTGCCACTACTTGTGGTT ACTTACCCCAAAATGCTGTT GTTAAAATTTATTGTCCAGC ATGTCACAATTCAGAAGTAG 1400
1401 GACCTGAGCATAGTCTTGCC GAATACCATAATGAATCTGG CTTGAAAACCATTCTTCGTA AGGGTGGTCGCACTATTGCC TTTGGAGGCTGTGTGTTCTC 1500
1501 TTATGTTGGTTGCCATAACA AGTGTGCCTATTGGGTTCCA CGTGCTAGCGCTAACATAGG TTGTAACCATACAGGTGTTG TTGGAGAAGGTTCCGAAGGT 1600
1601 CTTAATGACAACCTTCTTGA AATACTCCAAAAAGAGAAAG TCAAGATCAATATTGTTGGT GACTTTAAACTTAATGAAGA GATCGCCATTATTTTGGCAT 1700
1701 CTTTTTCTGCTTCCACAAGT GCTTTTGTGGAAACTGTGAA AGGTTTGGATTATAAAGCAT TCAAACAAATTGTTGAATCC TGTGGTAATTTTAAAGTTAC 1800
1801 AAATGGAAAAGCTAAAAAAG GTGCCTGGAATATTGGTGAA CAGAAATCAATACTGAGTCC TCTTTATGCATTTGCATCAG AGGCTGCTCGTGTTGTACGA 1900
1901 TCAATTTTCTCCCGCACTCT TGAAACTGCTCAAAATTCTG TGCGTGTTTTACAGAAGGCC GCTATAACAATACTAGATGG AATTTCACAGTATTCACTGA 2000
2001 GACTCATTGATGCTATGATG TTCACATCTGATTTGGCTAC TAACAATCTAGTTGTAATGG CCTACATTACAGGTGGTGTT GTTCAGTTGACTTCGCAGTG 2100
2101 GCTAACTAACATCTTTGGCA CTGTTTATGAAAAACTCAAA CCCGTCCTTGATTGGCTTGA AGAGAAGTTTAAGGAAGGTG TAGAGTTTCTTAGAGACGGT 2200
2201 TGGGAAATTGTTAAATTTAT CTCAACCTGTGCTTGTGAAA TTGTCGGAAGTCGGATTGTC ACCTGTGCAAAGGAAATTAA GGAGAGTGTTCAGACATTCT 2300
2301 TTAAGCTTGTAAATAAATTT TTGGCTTTGTGTGCTGACTC TATCATTATTGGTGGAGCTA AACTTAAAGCCTTGAATTTA GGTGAAACATTTGTCACGCA 2400
2401 CTCAAAGGGATTGTACAGAA AGTGTGTTAAATCCAGAGAA GAAACTGGCAAGTCGGATCG TAGAAAAGCCCCAAAAGAAA TTATCTTCTTAGAGGGAGAA 2500
2501 ACACTTCCCACAGAAGTGTT AACAGAGGAAGTTGTCTTGA AAACTGGTGATTTACAACCA TTAGAACAACCTACTAGTGA AGCTGTTGAAGCTCCATTGG 2600
2601 TTGGTACACCAGTTTGTATT AACGGGCTTATGTTGCTCGA AATCAAAGACACAGAAAAGT ACTGTGCCCTTGCACCTAAT ATGATGGTAACAAACAATAC 2700
2701 CTTCACACTCAAAGGCGGTG CACCAACAAAGGTTACTTTT GGTGATGACACTGTGATAGA AGTGCAAGGTTACAAGAGTG TGAATATCACTTTTGAACTT 2800
2801 GATGAAAGGATTGATAAAGT ACTTAATGAGAAGTGCTCTG CCTATACAGTTGAACTCGGT ACAGAAGTAAATGAGTTCGC CTGTGTTGTGGCAGATGCTG 2900
2901 TCATAAAAACTTTGCAACCA GTATCTGAATTACTTACACC ACTGGGCATTGATTTAGATG AGTGGAGTATGGCTACATAC TACTTATTTGATGAGTCTGG 3000
3001 TGAGTTTAAATTGGCTTCAC ATATGTATTGTTCTTTCTAC CCTCCAGATGAGGATGAAGA AGAAGGTGATTGTGAAGAAG AAGAGTTTGAGCCATCAACT 3100
3101 CAATATGAGTATGGTACTGA AGATGATTACCAAGGTAAAC CTTTGGAATTTGGTGCCACT TCTGCTGCTCTTCAACCTGA AGAAGAGCAAGAAGAAGATT 3200
3201 GGTTAGATGATGATAGTCAA CAAACTGTTGGTCAACAAGA CGGCAGTGAGGACAATCAGA CAATTACTATTCAAACAATT GTTGAGGTTCAACCTCAATT 3300
3301 AGAGATGGAACTTACACCAG TTGTTCAGACTATTGAAGTG AATAGTTTTAGTGGTTATTT AAAACTTACTGACAATGTAT ACATTAAAAATGCAGACATT 3400
3401 GTGGAAGAAGCTAAAAAGGT AAAACCAACAGTGGTTGTTA ATGCAGCCAATGTTTACCTT AAACATGGAGGAGGTGTTGC AGGAGCCTTAAATAAGGCTA 3500
3501 CTAACAATGCCATGCAAGTT GAATCTGATGATTACATAGC TACTAATGGACCACTTAAAG TGGGTGGTAGTTGTGTTTTA AGCGGACACAATCTTGCTAA 3600
3601 ACACTGTCTTCATGTTGTCG GCCCGAATGTTAACAAAGGT GAAGACATTCAACTTCTTAA GAGTGCTTATGAAAATTTTA ATCAGCACGAAGTTCTACTT 3700
3701 GCACCATTATTATCAGCTGG TATTTTTGGTGCTGACCCTA TACATTCTTTAAGAGTTTGT GTAGATACTGTTCGCACAAA TGTTTACTTAGCTGTCTTTG 3800
3801 ATAAAAATCTCTATGACAAA CTTGTTTCAAGCTTTTTGGA AATGAAGAGTGAAAAGCAAG TTGAACAAAAGATCGCTGAG ATTCCTAAAGAGGAAGTTAA 3900
3901 GCCATTTATAATTGAAAGTA AACCTTCAGTTGAACAGAGA AAACAAGATGATAAGAAAAT CAAAGCTTGTGTTGAAGAAG TTACAACAACTCTGGAAGAA 4000
4001 ACTAAGTTCCTCACAGAAAA CTTGTTACTTTATATTGACA TTAATGGCAATCTTCATCCA GATTCTGCCACTCTTGTTAG TGACATTGACATCACTTTCT 4100
4101 TAAAGAAAGATGCTCCATAT ATAGTGGGTGATGTTGTTCA AGAGGGTGTTTTAACTGCTG TGGTTATACCTACTAAAAAG GCTGGTGGCACTACTGAAAT 4200
4201 GCTAGCGAAAGCTTTGAGAA AAGTGCCAACAGACAATTAT ATAACCACTTACCCGGGTCA GGGTTTAAATGGTTACACTG TAGAGGATGCAAAGACAGTG 4300
4301 CTTAAAAAGTGTAAAAGTGC CTTTTACATTCTACCATCTA TTATCTCTAATGAGAAGCAA GAAATTCTTGGAACTGTTTC TTGGAATTTGCGAGAAATGC 4400
4401 TTGCACATGCAGAAGAAACA CGCAAATTAATGCCTGTCTG TGTGGAAACTAAAGCCATAG TTTCAACTATACAGCGTAAA TATAAGGGTATTAAAATACA 4500
4501 AGAGGGTGTGGTTGATTATG GTGCTAGATTTTACTTTTAC ACCAGTAAAACAACTGTAGC GTCACTTATCAACACACTTA ACGATCTAAATGAAACTCTT 4600
4601 GTTACAATGCCACTTGGCTA TGTAACACATGGCTTAAATT TGGAAGAAGCTGCTCGGTAT ATGAGATCTCTCAAAGTGCC AGCTACAGTTTCTGTTTCTT 4700
4701 CACCTGATGCTGTTACAGCG TATAATGGTTATCTTACTTC TTCTTCTAAAACACCTGAAG AACATTTTATTGAAACCATC TCACTTGCTGGTTCCTATAA 4800
4801 AGATTGGTCCTATTCTGGAC AATCTACACAACTAGGTATA GAATTTCTTAAGAGAGGTGA TAAAAGTGTATATTACACTA GTAATCCTACCACATTCCAC 4900
4901 CTAGATGGTGAAGTTATCAC CTTTGACAATCTTAAGACAC TTCTTTCTTTGAGAGAAGTG AGGACTATTAAGGTGTTTAC AACAGTAGACAACATTAACC 5000
5001 TCCACACGCAAGTTGTGGAC ATGTCAATGACATATGGACA ACAGTTTGGTCCAACTTATT TGGATGGAGCTGATGTTACT AAAATAAAACTTCATAATTC 5100
5101 ACATGAAGGTAAAACATTTT ATGTTTTACCTAATGATGAC ACTCTACGTGTTGAGGCTTT TGAGTACTACCACACAACTG ATCCTAGTTTTCTGGGTAGG 5200
5201 TACATGTCAGCATTAAATCA CACTAAAAAGTGGAAATACC CACAAGTTAATGGTTTAACT TCTATTAAATGGGCAGATAA CAACTGTTATCTTGCCACTG 5300
5301 CATTGTTAACACTCCAACAA ATAGAGTTGAAGTTTAATCC ACCTGCTCTACAAGATGCTT ATTACAGAGCAAGGGCTGGT GAAGCTGCTAACTTTTGTGC 5400
5401 ACTTATCTTAGCCTACTGTA ATAAGACAGTAGGTGAGTTA GGTGATGTTAGAGAAACAAT GAGTTACTTGTTTCAACATG CCAATTTAGATTCTTGCAAA 5500
5501 AGAGTCTTGAACGTGGTGTG TAAAACTTGTGGACAACAGC AGACAACCCTTAAGGGTGTA GAAGCTGTTATGTACATGGG CACACTTTCTTATGAACAAT 5600
5601 TTAAGAAAGGTGTTCAGATA CCTTGTACGTGTGGTAAACA AGCTACAAAATATCTAGTAC AACAGGAGTCACCTTTTGTT ATGATGTCAGCACCACCTGC 5700
5701 TCAGTATGAACTTAAGCATG GTACATTTACTTGTGCTAGT GAGTACACTGGTAATTACCA GTGTGGTCACTATAAACATA TAACTTCTAAAGAAACTTTG 5800
5801 TATTGCATAGACGGTGCTTT ACTTACAAAGTCCTCAGAAT ACAAAGGTCCTATTACGGAT GTTTTCTACAAAGAAAACAG TTACACAACAACCATAAAAC 5900
5901 CAGTTACTTATAAATTGGAT GGTGTTGTTTGTACAGAAAT TGACCCTAAGTTGGACAATT ATTATAAGAAAGACAATTCT TATTTCACAGAGCAACCAAT 6000
6001 TGATCTTGTACCAAACCAAC CATATCCAAACGCAAGCTTC GATAATTTTAAGTTTGTATG TGATAATATCAAATTTGCTG ATGATTTAAACCAGTTAACT 6100
6101 GGTTATAAGAAACCTGCTCT CCGACTTCTTAAAGTTACAT TTTTCCCTGACTTAAATGGT GATGTGGTGGCTATTGATTA TAAACACTACACACCCTCTT 6200
6201 TTAAGAAAGGAGCTAAATTG TTACATAAACCTATTGTTTG GCATGTTAACAATGCAACTA ATAAAGCCAAGTCGGATCCA AATACCTGGTGTATACGTTG 6300
6301 TCTTTGGAGCACAAAACCAG TTGAAACATCAAATTCGTTT GATGTACTGAAGTCAGAGGA CGCGCAGGGAATGGATAATC TTGCCTGCGAAGATCTAAAA 6400
6401 CCAGTCTCTGAAGAAGTAGT GGAAAATCCTACCATACAGA AAGACGTTCTTGAGTGTAAT GTGAAAACTACCGAAGTTGT AGGAGACATTATACTTAAAC 6500
6501 CAGCAAATAATAGTTTAAAA ATTACAGAAGAGGTTGGCCA CACAGATCTAATGGCTGCTT ATGTAGACAATTCTAGTCTT ACTATTAAGAAACCTAATGA 6600
6601 ATTATCTAGAGTATTAGGTT TGAAAACCCTTGCTACTCAT GGTTTAGCTGCTGTTAATAG TGTCCCTTGGGATACTATAG CTAATTATGCTAAGCCTTTT 6700
6701 CTTAACAAAGTTGTTAGTAC AACTACTAACATAGTTACAC GGTGTTTAAACCGTGTTTGT ACTAATTATATGCCTTATTT CTTTACTTTATTGCTACAAT 6800
6801 TGTGTACTTTTACTAGAAGT ACAAATTCTAGAATTAAAGC ATCTATGCCGACTACTATAG CAAAGAATACTGTTAAGAGT GTCGGTAAATTTTGTCTAGA 6900
6901 GGCTTCATTTAATTATTTGA AGTCACCTAATTTTTCTAAA CTGATAAATATTATAATTTG GTTTTTACTATTAAGTGTTT GCCTAGGTTCTTTAATCTAC 7000
7001 TCAACCGCTGCTTTAGGTGT TTTAATGTCTAATTTAGGCA TGCCTTCTTACTGTACTGGT TACAGAGAAGGCTATTTGAA CTCTACTAATGTCACTATTG 7100
7101 CAACCTACTGTACTGGTTCT ATACCTTGTAGTGTTTGTCT TAGTGGTTTAGATTCTTTAG ACACCTATCCTTCTTTAGAA ACTATACAAATTACCATTTC 7200
7201 ATCTTTTAAATGGGATTTAA CTGCTTTTGGCTTAGTTGCA CCGCTTGGCCTCCGACTTAT TCTTTTCACTAGGTTTTTCT ATGTACTTGGATTGGCTGCA 7300
7301 ATCATGCAATTGTTTTTCAG CTATTTTGCAGTACATTTTA TTAGTAATTCTTGGCTTATG TGGTTAATAATTAATCTTGT ACAAATGGCCCCGATTTCAG 7400
7401 CTATGGTTAGAATGTACATC TTCTTTGCATCATTTTATTA TGTATGGAAAAGTTATGTGC ATGTTGTAGACGGTTGTAAT TCATCAACTTGTATGATGTG 7500
7501 TTACAAACGTAATAGAGCAA CAAGAGTCGAATGTACAACT ATTGTTAATGGTGTTAGAAG GTCCTTTTATGTCTATGCTA ATGGAGGTAAAGGCTTTTGC 7600
7601 AAACTACACAATTGGAATTG TGTTAATTGTGATACATTCT GTGCTGGTAGTACATTTATT AGTGATGAAGTTGCGAGAGA CTTGTCACTACAGTTTAAAA 7700
7701 GACCAATAAATCCTACTGAC CAGTCTTCTTACATCGTTGA TAGTGTTACAGTGAAGAATG GTTCCATCCATCTTTACTTT GATAAAGCTGGTCAAAAGAC 7800
7801 TTATGAAAGACATTCTCTCT CTCATTTTGTTAACTTAGAC AACCTGAGAGCTAATAACAC TAAAGGTTCATTGCCTATTA ATGTTATAGTTTTTGATGGT 7900
7901 AAATCAAAATGTGAAGAATC ATCTGCAAAGTCGGATCGTG TTTACTACAGTCAGCTTATG TGTCAACCTATACTGTTACT AGATCAGGCATTAGTGTCTG 8000
8001 ATGTTGGTGATAGTGCGGAA GTTGCAGTTAAAATGTTTGA TGCTTACGTTAATACGTTTT CATCAACTTTTAACGTACCA ATGGAAAAACTCAAAACACT 8100
8101 AGTTGCAACTGCAGAAGCTG AACTTGCAAAGAATGTGTCC TTAGACAATGTCTTATCTAC TTTTATTTCAGCGCTTGGCC TCCGACTTGTTGATTCAGAT 8200
8201 GTAGAAACTAAAGATGTTGT TGAATGTCTTAAATTGTCAC ATCAATCTGACATAGAAGTT ACTGGCGATAGTTGTAATAA CTATATGCTCACCTATAACA 8300

8301 AAGTTGAAAACATGACACCC CGTGACCTTGGTGCTTGTAT TGACTGTAGTGCGCGTCATA TTAATGCGCAGGTAGCAAAA AGTCACAACATTGCTTTGAT 8400
8401 ATGGAACGTTAAAGATTTCA TGTCATTGTCTGAACAACTA CGAAAACAAATACGTAGTGC TGCTAAAAAGAATAACTTAC CTTTTAAGTTGACATGTGCA 8500
8501 ACTACTAGACAAGTTGTTAA TGTTGTAACAACAAAGATAG CACTTAAGGGTGGTAAAATT GTTAATAATTGGTTGAAGCA GTTAATTAAAGTTACACTTG 8600
8601 TGTTCCTTTTTGTTGCTGCT ATTTTCTATTTAATAACACC TGTTCATGTCATGTCTAAAC ATACTGACTTTTCAAGTGAA ATCATAGGATACAAGGCTAT 8700
8701 TGATGGTGGTGTCACTCGTG ACATAGCATCTACAGATACT TGTTTTGCTAACAAACATGC TGATTTTGACACATGGTTTA GCCAGCGTGGTGGTAGTTAT 8800
8801 ACTAATGACAAAGCTTGCCC ATTGATTGCTGCAGTCATAA CAAGAGAAGTGGGTTTTGTC GTGCCTGGTTTGCCTGGCAC GATATTACGCACAACTAATG 8900
8901 GTGACTTTTTGCATTTCTTA CCTAGAGTTTTTAGTGCAGT TGGTAACATCTGTTACACAC CATCAAAACTTATAGAGTAC ACTGACTTTGCAACATCAGC 9000
9001 TTGTGTTTTGGCTGCTGAAT GTACAATTTTTAAAGATGCT TCTGGTAAGCCAGTACCATA TTGTTATGATACCAATGTAC TAGAAGGTTCTGTTGCTTAT 9100
9101 GAAAGTTTACGCCCTGACAC ACGTTATGTGCTCATGGATG GCTCTATTATTCAATTTCCT AACACCAACCTTGAAGGTTC TGTTAGAGTGGTAACAACTT 9200
9201 TTGATTCTGAGTACTGTAGG CACGGCACTTGTGAAAGATC AGAAGCTGGTGTTTGTGTAT CTACTAGTGGTAGATGGGTA CTTAACAATGATTATTACAG 9300
9301 ATCTTTACCAGGAGTTTTCT GTGGTGTAGATGCTGTAAAT TTACTTACTAATATGTTTAC ACCACTAATTCAACCTATTG GTGCTTTGGACATATCAGCA 9400
9401 TCTATAGTAGCTGGTGGTAT TGTAGCTATCGTAGTAACAT GCCTTGCCTACTATTTTATG AGGTTTAGAAGAGCTTTTGG TGAATACAGTCATGTAGTTG 9500
9501 CCTTTAATACTTTACTATTC CTTATGTCATTCACTGTACT CTGTTTAACACCAGTTTACT CATTCTTACCTGGTGTTTAT TCTGTTATTTACTTGTACTT 9600
9601 GACATTTTATCTTACTAATG ATGTTTCTTTTTTAGCACAT ATTCAGTGGATGGTTATGTT CACACCTTTAGTACCTTTCT GGATAACAATTGCTTATATC 9700
9701 ATTTGTATTTCCACAAAGCA TTTCTATTGGTTCTTTAGTA ATTACCTAAAGAGACGTGTA GTCTTTAATGGTGTTTCCTT TAGTACTTTTGAAGAAGCTG 9800
9801 CGCTGTGCACCTTTTTGTTA AATAAAGAAATGTATCTAAA GTTGCGTAGTGATGTGCTAT TACCTCTTACGCAATATAAT AGATACTTAGCTCTTTATAA 9900
9901 TAAGTACAAGTATTTTAGTG GAGCAATGGATACAACTAGC TACAGAGAAGCTGCTTGTTG TCATCTCGCAAAGGCTCTCA ATGACTTCAGTAACTCAGGT 10000
10001 TCTGATGTACTTTACCAACC ACCACAAACCTCTATCACCT CAGCTGTTTTGCAGAGTGGT TTTAGAAAAATGGCATTCCC ATCTGGTAAAGTTGAGGGTT 10100
10101 GTATGGTACAAGTAACTTGT GGTACAACTACACTTAACGG TCTTTGGCTTGATGACGTAG TTTACTGTCCAAGACATGTG ATCTGCACCTCTGAAGACAT 10200
10201 GCTTAACCCTAATTATGAAG ATTTACTCATTCGTAAGTCT AATCATAATTTCTTGGTACA GGCTGGTAATGTTCAACTCA GGGTTATTGGACATTCTATG 10300
10301 CAAAATTGTGTACTTAAGCT TAAGGTTGATACAGCCAATC CTAAGACACCTAAGTATAAG TTTGTTCGCATTCAACCAGG ACAGACTTTTTCAGTGTTAG 10400
10401 CTTGTTACAATGGTTCACCA TCTGGTGTTTACCAATGTGC TATGAGGCCCGATTTCACTA TTAAGGGTTCATTCCTTAAT GGTTCATGTGGTAGTGTTGG 10500
10501 TTTTAACATAGATTATGACT GTGTCTCTTTTTGTTACATG CACCATATGGAATTACCAAC TGGAGTTCATGCTGGCACAG ACTTAGAAGGTAACTTTTAT 10600
10601 GGACCTTTTGTTGACAGGCA AACAGCACAAGCAGCTGGTA CGGACACAACTATTACAGTT AATGTTTTAGCTTGGTTGTA CGCTGCTGTTATAAATGGAG 10700
10701 ACAGGTGGTTTCTCAATCGA TTTACCACAACTCTTAATGA CTTTAACCTTGTGGCTATGA AGTACAATTATGAACCTCTA ACACAAGACCATGTTGACAT 10800
10801 ACTAGGACCTCTTTCTGCTC AAACTGGAATTGCCGTTTTA GATATGTGTGCTTCATTAAA AGAATTACTGCAAAATGGTA TGAATGGACGTACCATATTG 10900
10901 GGTAGTGCTTTATTAGAAGA TGAATTTACACCTTTGATGT TGTTAGACAATGCTCAGGTG TTACTTTCCAAAGTGCAGTG AAAAGAACAATCAAGGGTAC 11000
11001 ACACCACTGGTTGTTACTCA CAATTTTGACTTCACTTTTA GTTTTAGTCCAGAGTACTCA ATGGTCTTTGTTCTTTTTTT TGTATGAAAATGCCTTTTTA 11100
11101 CCTTTTGCTATGGGTATTAT TGCTATGTCTGCTTTTGCAA TGATGTTTGTCAAACATAAG CATGCATTTCTCTGTTTGTT TTTGTTACCTTCTCTTGCCA 11200
11201 CTGTAGCTTATTTTAATATG GTCTATATGCCTGCTAGTTG GGTGATGCGTATTATGACAT GGTTGGATATGGTTGATACT AGTTTGTCTGGTTTTAAGCT 11300
11301 AAAAGACTGTGTTATGTATG CATCAGCTGTAGTGTTACTA ATCCTTATGACAGCAAGAAC TGTGTATGATGATGGTGCTA GGAGAGTGTGGACACTTATG 11400
11401 AATGTCTTGACACTCGTTTA TAAAGTTTATTATGGTAATG CTTTAGATCAAGCCATTTCC ATGTGGGCTCTTATAATCTC TGTTACTTCTAACTACTCAG 11500
11501 GTGTAGTTACAACTGTCATG TTTTTGGCCAGAGGTATTGT TTTTATGTGTGTTGAGTATT GCCCTATTTTCTTCATAACT GGTAATACACTTCAGTGTAT 11600
11601 AATGCTAGTTTATTGTTTCT TAGGCTATTTTTGTACTTGT TACTTTGGCCTCTTTTGTTT ACTCAACCGCTACTTTAGAC TGACTCTTGGTGTTTATGAT 11700
11701 TACTTAGTTTCTACACAGGA GTTTAGATATATGAATTCAC AGGGACTACTCCCACCCAAG AATAGCATAGATGCCTTCAA ACTCAACATTAAATTGTTGG 11800
11801 GTGTTGGTGGCAAACCTTGT ATCAAAGTAGCCACTGTACA GTCTAAAATGTCAGATGTAA AGTGCACATCAGTAGTCTTA CTCTCAGTTTTGCAACAACT 11900
11901 CAGAGTAGAATCATCATCTA AATTGTGGGCTCAATGTGTC CAGTTACACAATGACATTCT CTTAGCTAAAGATACTACTG AAGCCTTTGAAAAAATGGTT 12000
12001 TCACTACTTTCTGTTTTGCT TTCCATGCAGGGTGCTGTAG ACATAAACAAGCTTTGTGAA GAAATGCTGGACAACAGGGC AACCTTACAAGCTATAGCCT 12100
12101 CAGAGTTTAGTTCCCTTCCA TCATATGCAGCTTTTGCTAC TGCTCAAGAAGCTTATGAGC AGGCTGTTGCTAATGGTGAT TCTGAAGTTGTTCTTAAAAA 12200
12201 GTTGAAGAAGTCTTTGAATG TGGCTAAATCTGAATTTGAC CGTGATGCAGCCATGCAACG TAAGTTGGAAAAGATGGCTG ATCAAGCTATGACCCAAATG 12300
12301 TATAAACAGGCTAGATCTGA GGACAAGAGGGCAAAAGTTA CTAGTGCTATGCAGACAATG CTTTTCACTATGCTTAGAAA GTTGGATAATGATGCACTCA 12400
12401 ACAACATTATCAACAATGCA AGAGATGGTTGTGTTCCCTT GAACATAATACCTCTTACAA CAGCAGCCAAACTAATGGTT GTCATACCAGACTATAACAC 12500
12501 ATATAAAATACGTGTGATGG TACAACATTTACTTATGCAT CAGCATTGTGGGAAATCCAA CAGGTTGTAGATGCAGATAG TAAAATTGTTCAACTTAGTG 12600
12601 AAATTAGTATGGACAATTCA CCTAATTTAGCATGGCCTCT TATTGTAACAGCTTTAAGGG CCAATTCTGCTGTCAAATTA CAGAATAATGAGCTTAGTCC 12700
12701 TGTTGCACTACGACAGATGT CTTGTGCTGCCGGTACTACA CAAACTGCTTGCACTGATGA CAATGCGTTAGCTTACTACA ACACAACAAAGGGAGGTAGG 12800
12801 TTTGTACTTGCACTGTTATC CGATTTACAGGATTTGAAAT GGGCAAGATTCCCTAAGAGT GATGGAACTGGTACTATCTA TACAGAACTGGAACCACCTT 12900
12901 GTAGGTTTGTTACAGACACA CCTAAAGGTCCTAAAGTGAA GTATTTATACTTTATTAAAG GATTAAACAACCTAAATAGA GGTATGGTACTTGGTAGTTT 13000
13001 AGCTGCCACAGTACGTCTAC AAGCTGGTAATGCAACAGAA GTGCCTGCCAATTCAACTGT ATTATCTTTCTGTGCTTTTG CTGTAGATGCTGCTAAAGCT 13100
13101 TACAAAGATTATCTAGCTAG TGGGGGACAACCAATCACTA ATTGTGTTAAGATGTTGTGT ACACACACTGGTACTGGTCA GGCAATAACAGTTACACCGG 13200
13201 AAGCCAATATGGATCAAGAA TCCTTTGGTGGTGCATCGTG TTGTCTGTACTGCCGTTGCC ACATAGATCATCCAAATCCT AAAGGATTTTGTGACTTAAA 13300
13301 AGGTAAGTATGTACAAATAC CTACAACTTGTGCTAATGAC CCTGTGGGTTTTACACTTAA AAACACAGTCTGTACCGTCT GCGGTATGTGGAAAGGTTAT 13400
13401 GGCTGTAGTTGTGATCAACT CCGCGAACCCATGCTTCAGT CAGCTGATGCACAATCGTTT TTAAACGGGTTTGCGGTGTA AGTGCAGCCCGTCTTACACC 13500
13501 GTGCGGCACAGGCACTAGTA CTGATGCCGTATACAGGGCT TTTGACATCTACAATGATAA AGTAGCTGGTTTTGCTAAAT TCCTAAAAACTAATTGTTGT 13600
13601 CGCTTCCAAGAAAAGGACGA AGATGACAATTTAATTGATT CTTACTTTGTAGTTAAGAGA CACACTTTCTCTAACTACCA ACATGAAGAAACAATTTATA 13700
13701 ATTTACTTAAGGATTGTCCA GCTGTTGCTAAACATGACTT CTTTAAGTTTAGAATAGACG GTGACATGGTACCACATATA TCACGTCAACGTCTTACTAA 13800
13801 ATACACAATGGCAGACCTCG TCTATGCTTTAAGGCATTTT GATGAAGGTAATTGTGACAC ATTAAAAGAAATACTTGTCA CATACAATTGTTGTGATGAT 13900
13901 GATTATTTCAATAAAAAGGA CTGGCATGATTTTGTAGAAA ACCCAGATATATTACGCGTA TACGCCAACTTAGGTGAACG TGTACGCCAAGCTTTGTTAA 14000
14001 AAACAGTACAATTCTGTGAT GCCATGCGAAATGCTGGTAT TGTTGGTGTACTGACATTAG ATAATCAAGATCTCAATGGT AACTGGTATGATTTCGGTGA 14100
14101 TTTCATACAAACCACGCCAG GTAGTGGAGTTCCTGTTGTA GATTCTTATTATTCATTGTT AATGCCTATATTAACCTTGA CCAGGGCTTTAACTGCAGAG 14200
14201 TCACATGTTGACACTGACTT AACAAAGCCTTACATTAAGT GGGATTTGTTAAAATATGAC TTCACGGAAGAGAGGTTAAA ACTCTTTGACCGTTATTTTA 14300
14301 AATATTGGGATCAGACATAC CACCCAAATTGTGTTAACTG TTTGGATGACAGATGCATTC TGCATTGTGCAAACTTTAAT GTTTTATTCTCTACAGTGTT 14400
14401 CCCACCTACAAGTTTTGGAC CACTAGTGAGAAAAATATTT GTTGATGGTGTTCCATTTGT AGTTTCAACTGGATACCACT TCAGAGAGCTAGGTGTTGTA 14500
14501 CATAATCAGGATGTAAACTT ACATAGCTCTAGACTTAGTT TTAAGGAATTACTTGTGTAT GCTGCTGACCCTGCTATGCA CGCTGCTTCTGGTAATCTAT 14600
14601 TACTAGATAAACGCACTACG TGCTTTTCAGTAGCTGCACT TACTAACAATGTTGCTTTTC AAACTGTCAAACCCGGTAAT TTTAACAAAGACTTCTATGA 14700
14701 CTTTGCTGTGTCTAAGGGTT TCTTTAAGGAAGGAAGTTCT GTTGAATTAAAACACTTCTT CTTTGCTCAGGATGGTAATG CTGCTATCAGCGATTATGAC 14800
14801 TACTATCGTTATAATCTACC AACAATGTGTGATATCAGAC AACTACTATTTGTAGTTGAA GTTGTTGATAAGTACTTTGA TTGTTACGATGGTGGCTGTA 14900
14901 TTAATGCTAACCAAGTCATC GTCAACAACCTAGACAAATC AGCTGGTTTTCCATTTAATA AATGGGGTAAGGCTAGACTT TATTATGATTCAATGAGTTA 15000
15001 TGAGGATCAAGATGCACTTT TCGCATATACAAAACGTAAT GTCATCCCTACTATAACCCA AATGAATCTTAAGTATGCCA TTAGTGCAAAGAATAGAGCT 15100
15101 CGCACCGTAGCTGGTGTCTC TATCTGTAGTACTATGACCA ATAGACAGTTTCATCAAAAA TTATTGAAATCAATAGCCGC CACTAGAGGAGCTACTGTAG 15200
15201 TAATTGGAACAAGCAAATTC TATGGTGGTTGGCACAACAT GTTAAAAACTGTTTATAGTG ATGTAGAAAACCCTCACCTT ATGGGTTGGGATTATCCTAA 15300
15301 ATGTGATAGAGCCATGCCTA ACATGCTTAGAATTATGGCC TCACTTGTTCTTGCTCGCAA ACATACAACGTGTTGTAGCT TGTCACACCGTTTCTATAGA 15400
15401 TTAGCTAATGAGTGTGCTCA AGTATTGAGTGAAATGGTCA TGTGTGGCGGTTCACTATAT GTTAAACCAGGTGGAACCTC ATCAGGAGATGCCACAACTG 15500
15501 CTTATGCTAATAGTGTTTTT AACATTTGTCAAGCTGTCAC GGCCAATGTTAATGCACTTT TATCTACTGATGGTAACAAA ATTGCCGATAAGTATGTCCG 15600
15601 CAATTTACAACACAGACTTT ATGAGTGTCTCTATAGAAAT AGAGATGTTGACACAGACTT TGTGAATGAGTTTTACGCAT ATTTGCGTAAACATTTCTCA 15700
15701 ATGATGATACTCTCTGACGA TGCTGTTGTGTGTTTCAATA GCACTTATGCATCTCAAGGT CTAGTGGCCAGCATAAAGAA CTTTAAGTCAGTTCTTTATT 15800
15801 ATCAAAACAATGTTTTTATG TCTGAAGCAAAATGTTGGAC TGAGACTGACCTTACTAAAG GACCTCATGAATTTTGCTCT CAACATACAATGCTAGTTAA 15900
15901 ACAGGGTGATGATTATGTGT ACCTTCCTTACCCAGATCCA TCAAGAATCCTAGGAGCCGG CTGTTTTGTAGATGATATCG TAAAAACAGATGGTACACTT 16000
16001 ATGATTGAACGGTTCGTGTC TTTAGCTATAGATGCTTACC CACTTACTAAACATCCTAAT CAGGAGTATGCTGATGTCTT TCATTTGTACTTACAATACA 16100
16101 TAAGAAAGCTACATGATGAG TTAACAGGACACATGTTAGA CATGTATTCTGTTATGCTTA CTAATGATAACACTTCAAGG TATTGGGAACCTGAGTTTTA 16200
16201 TGAGGCTATGTACACACCGC ATACAGTCTTACAGGCTGTT GGGGCTTGTGTTCTTTGCAA TTCACAGACTTCATTAAGAT GTGGTGCTTGCATACGTAGA 16300
16301 CCATTCTTATGTTGTAAATG CTGTTACGACAAGGTCATAT CAACATCACATAAATTAGTC TTGTCTGTTAATCCGTATGT TTGCAATGCTCCAGGTTGTG 16400
16401 ATGTCACAGATGTGACTCAA CTTTACTTAGGAGGTATGAG CTATTATTGTAAATCACATA AACCACCCATTAGTTTTCCA TTGTGTGCTAATGGACAAGT 16500
16501 TTTTGGTTTATATAAAAATA CATGTGTTGGTAGCGATAAT GTTACTGACTTTAATGCAAT TGCAACATGTGACTGGACAA ATGCTGGTGATTACATTTTA 16600
16601 GCTAACACCTGTACTGAAAG ACTCAAGCTTTTTGCAGCAG AAACGCTCAAAGCTACTGAG GAGACATTTAAACTGTCTTA TGGTATTGCTACTGTACGTG 16700
16701 AAGTGCTGTCTGACAGAGAA TTACATCTTTCATGGGAAGT TGGTAAACCTAGACAAGTCG GATCGCGAAATTATGTCTTT ACTGGTTATCGTGTAACTAA 16800
16801 AAACAGTAAAGTACAAATAG GAGAGTACACCTTTGAAAAA GGTGACTATGGTGATGCTGT TGTTTACCGAGGTACAACAA CTTACAAATTAAATGTTGGT 16900
16901 GATTATTTTGTGCTGACATC ACATACAGTAATGCCATTAA GTGCACCTACACTAGTGCCA CAAGAGCACTATGTTAGAAT TACTGGCTTATACCCAACAC 17000
17001 TCAATATCTCAGATGAGTTT TCTAGCAATGTTGCAAATTA TCAAAAGGTTGGTATGCAAA AGTATTCTACACTCCAGGGA CCACCTGGTACTGGTAAGAG 17100
17101 TCATTTTGCTATTGGCCTAG CTCTCTACTACCCTTCTGCT CGCATAGTGTATACAGCTTG CTCTCATGCCGCTGTTGATG CACTATGTGAGAAGGCATTA 17200
17201 AAATATTTGCCTATAGATAA ATGTAGTAGAATTATACCTG CACGTGCTCGTGTAGAGTGT TTTGATAAATTCAAAGTGAA TTCAACATTAGAACAGTATG 17300
17301 TCTTTTGTACTGTAAATGCA TTGCCTGAGACGACAGCAGA TATAGTTGTCTTTGATGAAA TTTCAATGGCCACAAATTAT GATTTGAGTGTTGTCAATGC 17400
17401 CAGATTACGTGCTAAGCACT ATGTGTACATTGGCGACCCT GCTCAATTACCTGCAAGTCG CACATTGCTAACTAAGGGCA CACTAGAACCAGAATATTTC 17500
17501 AATTCAGTGTGTAGACTTAT GAAAACTATAGGTCCAGACA TGTTCCTCGGAACTTGTCGG CGTTGTCCTGCTGAAATTGT TGACACTGTGAGTGCTTTGG 17600
17601 TTTATGATAATAAGCTTAAA GCACATAAAGACAAATCAGC TCAATGCGTTAAAATGTTTT ATAAGGGTGTTATCACGCAT GATGTTTCATCTGCAATTAA 17700
17701 CAGGCCACAAATAGGCGTGG TAAGAGAATTCCTTACACGT AACCCTGCTTGGAGAAAAGC TGTCTTTATTTCACCTTATA ATTCACAGAATGCTGTAGCC 17800
17801 TCAAAGATTTTGGGACTACC AATTCAAACTGTTGATTCAT CACAGGGCTCAGAATATGAC TATGTCATATTCACTCAAAC CACTGAAACAGCTCACTCTT 17900
17901 GTAATGTAAACAGATTTAAT GTTGCTATTACCAGAGCAAA AGTAGGCATACTTTGCATAA TGTCTGATAGAGACCTTTAT GACAAGTTGCAATTTACAAG 18000
18001 TCTTGAAATTCCACGTAGGG ACTTGGCAACTTTACAAGCT GAAAATGTAACAGGACTCTT TAAAGATTGTAGTAAGGTAA TCACTGGGTTACATCCTACA 18100
18101 CAGGCACCTACACACCTCAG TGTTGACACTAAATTCAAAA CTGAAGGTTTATGTGTTGAC ATACCTGGCATACCTAAGGA CATGACCTATAGAAGACTCA 18200
18201 TCTCTATGATGGGTTTTAAA ATGAATTATCAAGTTAATGG TTACCCTAACATGTTTATCA CCCGCGAAGAAGCTATAAGA CATGTACGTGCATGGATTGG 18300
18301 CTTCGATGTCGAGGGGTGTC ATGCTACTAGAGAAGCTGTT GGTACCAATTTACCTTTACA GCTAGGTTTTTCTACAGGTG TTAACCTAGTTGCTGTACCT 18400
18401 ACAGGTTATGTTGATACACC TAATAATACAGATTTTTCCA GAGTTAGTGCTAAACCACCG CCTGGAGATCAATTTAAACA CCTCATACCACTTATGTACA 18500
18501 AAGGACTTCCTTGGAATGTA GTGCGTATAAAGATTGTACA AATGTTAAGTGACACACTTA AAAATCTCTCTGACAGAGTC GTATTTGTCTTATGAGCACA 18600
18601 TGGCTTTGAGTTGACATCTA TGAAGTATTTTGTGAAAATA GGACCTGAGCGCACCTGTTG TCTATGTGATAGACGTGCCA CATGCTTTTCCACTGCTTCA 18700
18701 GACACTTATGCCTGTTGGCA TCATTCTATTGGATTTGATT ACGTCTATAATCCGTTTATG ATTGATGTTCAACAATGGGG TTTTACAGGTAACCTACAAA 18800
18801 GCAACCATGATCTGTATTGT CAAGTCCATGGTAATGCACA TGTAGCTAGTTGTGATGCAA TCATGACTAGGTGTCTAGCT GTCCACGAGTGCTTTGTTAA 18900
18901 GCGTGTTGACTGGACTATTG AATATCCTATAATTGGTGAT GAACTGAAGATTAATGCGGC TTGTAGAAAGGTTCAACACA TGGTTGTTAAAGCTGCATTA 19000
19001 TTAGCAGACAAATTCCCAGT TCTTCACGACATTGGTAACC CTAAAGCAATTAAGTGTGTA CCTCAAGCTGATGTAGAATG GAAGTTCTATGATGCACAGC 19100

19101 CTTGTAGTGACAAAGCTTAT AAAATAGAAGAATTATTCTA TTCTTATGCCACACATTCTG ACAAATTCACAGATGGTGTA TGCCTATTTTGGAATTGCAA 19200
19201 TGTCGATAGATATCCTGCTA ATTCCATTGTTTGTAGATTT GACACTAGAGTGCTATCTAA CCTTAACTTGCCTGGTTGTG ATGGTGGCAGTTTGTATGTA 19300
19301 AATAAACATGCATTCCACAC ACCAGCTTTTGATAAAAGTG CTTTTGTTAATTTAAAACAA TTACCATTTTTCTATTACTC TGACAGTCCATGTGAGTCTC 19400
19401 ATGGAAAACAAGTAGTGTCA GATATAGATTATGTACCACT AAAGTCTGCTACGTGTATAA CACGTTGCAATTTAGGTGGT GCTGTCTGTAGACATCATGC 19500
19501 TAATGAGTACAGATTGTATC TCGATGCTTATAACATGATG ATCTCAGCTGGCTTTAGCTT GTGGGTTTACAAACAATTTG ATACTTATAACCTCTGGAAC 19600
19601 ACTTTTACAAGACTTCAGAG TTTAGAAAATGTGGCTTTTA ATGTTGTAAATAAGGGACAC TTTGATGGACAACAGGGTGA AGTACCAGTTTCTATCATTA 19700
19701 ATAACACTGTTTACACAAAA GTTGATGGTGTTGATGTAGA ATTGTTTGAAAATAAAACAA CATTACCTGTTAATGTAGCA TTTGAGCTTTGGGCTAAGCG 19800
19801 CAACATTAAACCAGAAGTCG GATCGTAGCCACTCAATAAT TTGGGTGTGGACATTGCTGC TAATACTGTGATCTGGGACT ACAAAAGAGATGCTCCAGCA 19900
19901 CATATATCTACTATTGGTGT TTGTTCTATGACTGACATAG CCAAGAAACCAACTGAAACG ATTTGTGCACCACTCACTGT CTTTTTTGATGGTAGAGTTG 20000
20001 ATGGTCAAGTAGACTTATTT AGAAATGCCCGTAATGGTGT TCTTATTACAGAAGGTAGTG TTAAAGGTTTACAACCATCT GTAGGTCCCAAACAAGCTAG 20100
20101 TCTTAATGGAGTCACATTAA TTGGAGAAGCCGTCAAAACA CAGTTCAATTATTATAAGAA AGTTGATGGTGTTGTCCAAC AATTACCTGAAACTTACTTT 20200
20201 ACTCAGAGTAGAAATTTACA AGAATTTAAACCCAGGAGTC AAATGGAAATTGATTTCTTA GAATTAGCTATGGATGAATT CATTGAACGGTATAAATTAG 20300
20301 AAGGCTATGCCTTCGAACAT ATCGTTTATGGAGATTTTAG TCATAGTCAGTTAGGTGGTT TACATCTACTGATTGGACTA GCTAAACGTTTTAAGGAATC 20400
20401 ACCTTTTGAATTAGAAGATT TTATTCCTATGGACAGTACA GTTAAAAACTATTTCATAAC AGATGCGCAAACAGGTTCAT CTAAGTGTGTGTGTTCTGTT 20500
20501 ATTGATTTATTACTTGATGA TTTTGTTGAAATAATAAAAT CCCAAGATTTATCTGTAGTT TCTAAGGTTGTCAAAGTGAC TATTGACTATACAGAAATTT 20600
20601 CATTTATGCTTTGGTGTAAA GATGGCCATGTAGAAACATT TTACCCAAAATTACAATCTA GTCAAGCGTGGCAACCGGGT GTTGCTATGCCTAATCTTTA 20700
20701 CAAAATGCAAAGAATGCTAT TAGAAAAGTGTGACCTTCAA AATTATGGTGATAGTGCAAC ATTACCTAAAGGCATAATGA TGAATGTCGCAAAATATACT 20800
20801 CAACTGTGTCAATATTTAAA CACATTAACATTAGCTGTAC CCTATAATATGAGAGTTATA CATTTTGGTGCTGGTTCTGA TAAAGGAGTTGCACCAGGTA 20900
20901 CAGCTGTTTTAAGACAGTGG TTGCCTACGGGTACGCTGCT TGTCGATTCAGATCTTAATG ACTTTGTCTCTGATGCAGAT TCAACTTTGATTGGTGATTG 21000
21001 TGCAACTGTACATACAGCTA ATAAATGGGATCTCATTATT AGTGATATGTACGACCCTAA GACTAAAAATGTTACAAAAG AAAATGACTCTAAAGAGGGT 21100
21101 TTTTTCACTTACATTTGTGG GTTTATACAACAAAAGCTAG CTCTTGGAGGTTCCGTGGCT ATAAAGATAACAGAACATTC TTGGAATGCTGATCTTTATA 21200
21201 AGCTCATGGGACACTTCGCA TGGTGGACAGCCTTTGTTAC TAATGTGAATGCGTCATCAT CTGAAGCATTTTTAATTGGA TGTAATTATCTTGGCAAACC 21300
21301 ACGCGAACAAATAGATGGTT ATGTCATGCATGCAAATTAC ATATTTTGGAGGAATACAAA TCCAATTCAGTTGTCTTCCT ATTCTTTATTTGACATGAGT 21400
21401 AAATTTCCCCTTAAATTAAG GGGTACTGCTGTTATGTCTT TAAAAGAAGGTCAAATCAAT GATATGATTTTATCTCTTCT TAGTAAAGGTAGACTTATAA 21500
21501 TTAGAGAAAACAACAGAGTT GTTATTTCTAGTGATGTTCT TGTTAACAACTAAACGAACA ATGTTTGTTTTTCTTGTTTT ATTGTCACTAGTCTCTAGTC 21600
21601 AGTGTGTTAATCTTACAACC AGAACTCAATTACCCCCTGC ATACACTAATTCTTTCACAC GTGGTGTTTATTACCCTGAC AAAGTTTTCAGATCCTCAGT 21700
21701 TTTACATTCAACTCAGGACT TGTTCTTACCTTTCTTTTCC AATGTTACTTGGTTCCATGC TATACATGTCTCTGGGACCA ATGGTACTAAGAGGTTTGAT 21800
21801 AACCCTGTCCTACCATTTAA TGATGGTGTTTATTTTGCTT CCACTGAGAAGTCTAACATA ATAAGAGGCTGGATTTTTGG TACTACTTTAGATTCGAAGA 21900
21901 CCCAGTCCCTACTTATTGTT AATAACGCTACTAATGTTGT TATTAAAGTCTGTGAATTTC AATTTTGTAATGATCCATTT TTGGGTGTTTATTACCACAA 22000
22001 AAACAACAAAAGTTGGATGG AAAGTGAGTTCAGAGTTTAT TCTAGTGCGAATAATTGCAC TTTTGAATATGTCTCTCAGC CTTTTCTTATGGACCTTGAA 22100
22101 GGAAAACAGGGGAATTTCAA AAATCTTAGGGAATTTGTGT TTAAGAATATTGATGGTTAT TTTAAAATATATTCTAAGCA CACGCCTATTAATTTAGTGC 22200
22201 GTGATCTCCCTCAGGGTTTT TCGGCTTTAGAACCATTGGT AGATTTGCCAATAGGTATTA ACATCACTAGGCTTCAAACT TTACTTGCTTTACATAGAAG 22300
22301 TTATTTGACTCCTGGTGATT CTTCTTCAGGTTGGACAGCT GGTGCTGCAGCTTATTATGT GGGTTATCTTCAACCTAGGA CTTTTCTATTAAAATATAAT 22400
22401 GAAAATGGAACCATTACAGA TGCTGTAGACTGTGCACTTG ACCCTCTCTCAGAAACAAAG TGTACGTTGAAATCCTTCAC TGTAGAAAAAGGAATCTATC 22500
22501 AAACTTCTAACTTTAGAGTC CAACCAACAGAATCTATTGT TAGATTTCCTAATATTACAA ACTTGTGCCCTTTTGGTGAA GTTTTTAACGCCACCAGATT 22600
22601 TGCATCTGTTTATGCTTGGA ACAGGAAGAGAATCAGCAAC TGTGTTGCTGATTATTCTGT CCTATATAATTCCGCATCAT TTTCCACTTTTAAGTGTTAT 22700
22701 GGAGTGTCTCCTACTAAATT AAATGATCTCTGCTTTACTA ATGTCTATGCAGATTCATTT GTAATTAGAGGTGATGAAGT CAGACAAATCGCTCCAGGGC 22800
22801 AAACTGGAAAGATTGCTGAT TATAATTATAAATTACCAGA TGATTTTACAGGCTGCGTTA TAGCTTGGAATTCTAACAAT CTTGATTCTAAGGTTGGTGG 22900
22901 TAATTATAATTACCTGTATA GATTGTTTAGGAAGTCTAAT CTCAAACCTTTTGAGAGAGA TATTTCAACTGAAATCTATC AGGCCGGAAGCACACCTTGT 23000
23001 AATGGTGTTGAAGGTTTTAA TTGTTACTTTCCTTTACAAT CATATGGTTTCCAACCCACT AATGGTGTTGGTTACCAACC ATACAGAGTAGTAGTACTTT 23100
23101 CTTTTGAACTTCTACATGCA CCAGCAACTGTTTGTGGACC TAAAAAGTCTACTAATTTGG TTAAAAACAAATGTGTCAAT TTCAACTTCAATGGTTTAAC 23200
23201 AGGCACAGGTGTTCTTACTG AGTCTAACAAAAAGTTTCTG CCTTTCCAACAATTTGGCAG AGACATTGCTGACACTACTG ATGCTGTCCGTGATCCACAG 23300
23301 ACACTTGAGATTCTTGACAT TACACCATGTTCTTTTGGTG GTGTCAGTGTTATAACACCA GAAGTCGATACTTCTAACCA GGTTGCTGTTCTTTATCAGG 23400
23401 ATGTTAACTGCACAGAAGTC CCTGTTGCTATTCATGCAGA TCAACTTACTCCTACTTGGC GTGTTTATTCTACAGGTTCT AATGTTTTTCAAACACGTGC 23500
23501 AGGCTGTTTAATAGGGGCTG AACATGTCAACAACTCATAT GAGTGTGACATACCCATTGG TGCAGGTATATGCGCTAGTT ATCAGACTCAGACTAATTCT 23600
23601 CCCCGGCGGGCACGTAGTGT AGCTAGTCAATCCATCATTG CCTACACTATGTCACTTGGT GCAGAAAATTCAGTTGCTTA CTCTAATAACTCTATTGCCA 23700
23701 TACCCACAAATTTTACTATT AGTGTTACCACAGAAATTCT ACCAGTGTCTATGACCAAGA CATCAGTAGATTGTACAATG TACATTTGTGGTGATTCAAC 23800
23801 TGAATGCAGCAATCTTTTGT TGCAATATGGCAGTTTTTGT ACACAATTAAACCGTGGTTT AACTGGAATAGCTGTTGAAC AAGACAAAAACACCCAAGAA 23900
23901 GTTTTTGCACAAGTCAAACA AATTTACAAAACACCACCAA TTAAAGATTTTGGTGGTTTT AATTTTTCACAAATATTACC AGATCCATCAAAACCAAGCA 24000
24001 AGAGGTCATTTATTGAAGAT CTACTTTTCAACAAAGTGAC ACTTGCAGATGCTGGCTTCA TCAAACAATATGGTGATTGC CTTGGTGATATTGCTGCTAG 24100
24101 AGACCACATTTGTGCACAAA AGTTTAACGGCCTTACTGTT TTGCCACCTTTGCTCACAGA TGAAATGATTGCTCAATACA CTTCTGCACTGTTAGCGGGT 24200
24201 ACAATCACTTCTGGTTGGAC CTTTGGTGCAGGTGCTGCAT TACAAATACCATTTGCAATG CAAATGGCTTATAGGTTTAA TGGTATTGGAGTTACACAGA 24300
24301 ATGTTCTCTATGAGAACCAA AAATTGATTGCCAACCAATT TAATAGTGCTATTGGCAAAA TTCAAGACTCACTTTCTTCC ACAGCAAGTGCACTTGGAAA 24400
24401 ACTTCAAGATGTGGTCAACC AAAATGCACAAGCTTTAAAC ACGCTTGTTAAACAACTTAG CTCCAATTTTGGTGCAATTT CAAGTGTTTTAAATGATATC 24500
24501 CTTTCACGTCTTGACAAAGT TGAGGCTGAAGTGCAAATTG ATAGGTTGATCACAGGCAGA CTTCAAAGTTTGCAGACATA TGTGACTCAACAATTAATTA 24600
24601 GAGCTGCAGAAATCAGAGCT TCTGCTAATCTTGCTGCTAC TAAAATGTCACTCCGACTTC TTGGACAATCAAAAAGAGTT GATTTTTGTGGAAAGGGCTA 24700
24701 TCATCTTATGTCCTTCCCTC AGTCAGCACCTCATGGTGTA GTCTTCTTGCATGTGACTTA TGTCCCTGCACAAGAAAAGA ACTTCACAACTGCTCCTGCC 24800
24801 AAGTCGGATGATGGAAAAGC ACACTTTCCTCGTGAAGGTG TCTTTGTTTCAAATGGCACA CACTGGTTTGTAACACAAAG GAATTTTTATGAACCACAAA 24900
24901 TCATTACTACAGACAACACA TTTGTGTCTGGTAACTGTGA TGTTGTAATAGGAATTGTCA ACAACACAGTTTATGATCCT TTGCAACCTGAATTAGACTC 25000
25001 ATTCAAGGAGGAGTTAGATA AATATTTTAAGAATCATACA TCAGCAGATGTTGATTTAGG TGACATCTCTGGCATTAATG CTTCAGTTGTAAACATTCAA 25100
25101 AAAGAAATTGACCGCCTCAA TGAGGTTGCCAAGAATTTAA ATGAATCTCTCATCGATCTC CAAGAACTTGGAAAGTATGA GCAGTATATAAAATGGCCAT 25200
25201 GGTACATTTGGCTAGGTTTT ATAGCTGGCTTGATTGCCAT AGTAATGGTGACAATTATGC TTTGCTGTATGACCAGTTGC TGTAGTTGTCTCAAGGGCTG 25300
25301 TTGTTCTTGTGGATCCTGCT GCAAATTTGATGAAGACGAC TCTGAGCCAGTGCTCAAAGG AGTCAAATTACATTACACAT AAACGAACTTATGGATTTGT 25400
25401 TTATGAGAATCTTCACAATT GGAACTGTAACTTTGAAGCA AGGTGAAATCAAGGATGCTA CTCCTTCAGATTTTGTTCGC GCTACTGCAACGATACCGAT 25500
25501 ACAAGCCTCACTCCCCCTCC GACTTCTTATTGTTGGCGTT GCACTTCTTGCTGTTTTTCA GAGCGCTTCCAAAATCATAA CCCTCAAAAAGAGATGGCAA 25600
25601 CTAGCACTCTCCAAGGGTGT TCACTTTGTTTGCAACTTGC TGTTGTTGTTTGTAACAGTT TACTCAAGTCGGATCCTCGT TGCTGCTGGCCTTGAAGCCC 25700
25701 CTTTTCTCTATCTTTATGCT TTAGTCTACTTCTTGCAGAG TATAAACTTTGTAAGAATAA TAATGAGGCTTTGGCTTTGC TGGAAATGCCGTTCCAAAAA 25800
25801 CCCATTACTTTATGATGCCA ACTATTTTCTTTGCTGGCAT ACTAATTGTTACGACTATTG TATACCTTACAATAGTGTAA CTTCTTCAATTGTCATTACT 25900
25901 TCAGGTGATGGCACAACAAG TCCTATTTCTGAACATGACT ACCAGATTGGTGGTTATACT GAAAAATGGGAATCTGGAGT AAAAGACTGTGTTGTATTAC 26000
26001 ACAGTTACTTCACTTCAGAC TATTACCAGCTGTACTCAAC TCAATTGAGTACAGACACTG GTGTTGAACATGTTACCTTC TTCATCTACAATAAAATTGT 26100
26101 TGATGAGCCTGAAGAACATG TCCAAATTCACACAATCGAC GGTTCATCCGGAGTTGTTAA TCCAGTAATGGAACCAATTT ATGATGAACCGACGACGACT 26200
26201 ACTAGCGTGCCTTTGTAAGC ACAAGCTGATGAGTACGAAC TTATGTACTCATTCGTTTCG GAAGAGACAGGTACGTTAAT AGTTAATAGCGTACTTCTTT 26300
26301 TTCTTGCTTTCGTGGTATTC TTGCTAGTTACACTAGCCAT CCTTACTGCGCATCGATTGT GTGCGTACTGCTGCAATATT GTTAACGTGAGTCTTGTAAA 26400
26401 ACCTTCTTTTTACGTTTACT CTCGTGTTAAAAATCTGAAT TCTTCTAGAGTTCCTGATCT TCTGGTCTAAACGAACTAAA TATTATATTAGTTTTTCTGT 26500
26501 TTGGAACTTTAATTTTAGCC ATGGCAGATTCCAACGGTAC TATTACCGTTGAAGAGCTTA AAAAGCTCCTTGAACAATGG AACCTAGTAATAGGTTTCCT 26600
26601 ATTCCTTACATGGATTTGTC TTCTACAATTTGCCTATGCC AACAGGAATAGGTTTTTGTA TATAATTAAGTTAATTTTCC TCTGGCTGTTATGGCCAGTA 26700
26701 ACTTTAGCTTGTTTTGTGCT TGCTGCTGTTTACAGAATAA ATTGGATCACCGGTGGAATT GCTATCGCAATGGCTTGTCT TGTAGGCTTGATGTGGCTCA 26800
26801 GCTACTTCATTGCTTCTTTC AGACTGTTTGCGCTTGGCCT CCGACTTTGGTCATTCAATC CAGAAACTAACATTCTTCTC AACGTGCCACTCCATGGCAC 26900
26901 TATTCTGACCAGACCGCTTC TAGAAAGTGAACTCGTAATC GGAGCTGTGATCCTTCGTGG ACATCTTCGTATTGCTGGAC ACCATCTAGGACGCTGTGAC 27000
27001 ATCAAGGACCTGCCAAAAGA AATCACTGTTGCTACATCAC GAACGCTTTCTTATTACAAA TTGGGAGCTTCGCAGCGTGT AGCAGGTGACTCAGGTTTTG 27100
27101 CTGCATACAGTCGCTACAGG ATTGGCAACTATAAATTAAA CACAGACCATTCCAGTAGCA GTGACAATATTGCTTTGCTT GTACAGTAAGTGACAACAGA 27200
27201 TGTTTCATCTCGTTGACTTT CAGGTTACTATAGCAGAGAT ATTACTAATTATTATGAGGA CTTTTAAAGTTTCCATTTGG AATCTTGATTACATCATAAA 27300
27301 CCTCATAATTAAAAATTTAT CTAAGTCACTAACTGAGAAT AAATATTCTCAATTAGATGA AGAGCAACCGATGGAGATTG ATTAAACGAACATGAAAATT 27400
27401 ATTCTTTTCTTGGCACTGAT AACACTCGCTACTTGTGAGC TTTATCACTACCAAGAGTGT GTTAGAGGTACAACAGTACT TTTAAAAGAACCTTGCTCTT 27500
27501 CTGGAACATACGAGGGCAAT TCACCATTTCATCCTCTAGC TGATAACAAATTTGCACTGA CTTGCTTTAGCACTCAATTT GCTTTTGCTTGTCCTGACGG 27600
27601 CGTAAAACACGTCTATCAGT TACGTGCCAGATCAGTTTCA CCTAAACTGTTCATCAGACA AGAGGAAGTTCAAGAACTTT ACTCTCCAATTTTTCTTATT 27700
27701 GTTGCGGCAATAGTGTTTAT AACACTTTGCTTCACACTCA AAAGAAAGACAGAATGATTG AACTTTCATTAATTGACTTC TATTTGTGCTTTTTAGCCTT 27800
27801 TCTGCTATTCCTTGTTTTAA TTATGCTTATTATCTTTTGG TTCTCACTTGAACTGCAAGA TCATAATGAAACTTGTCACG CCGAAACGAACATGAAATTT 27900
27901 CTTGTTTTCTTAGGAATCAT CACAACTGTAGCTGCATTTC ACCAAGAATGTAGTTTACAG TCATGTACTCAACATCAACC ATATGTAGTTGATGACCCGT 28000
28001 GTCCTATTCACTTCTATTCT AAATGGTATATTAGAGTAGG AGCTAGAAAATCAGCACCTT TAATTGAATTGTGCGTGGAT GAGGCTGGTTCTAAATCACC 28100
28101 CATTCAGTACATCGATATCG GTAATTATACAGTTTCCTGT TTACCTTTTACAATTAATTG CCAGGAACCTAAATTGGGTA GTCTTGTAGTGCGTTGTTCG 28200
28201 TTCTATGAAGACTTTTTAGA GTATCATGACGTTCGTGTTG TTTTAGATTTCATCTAAACG AACAAACTAAAATGTCTGAT AATGGACCCCGAAATCAGCG 28300
28301 AAATGCACCCCGCATTACGT TTGGTGGACCCTCAGATTCA ACTGGCAGTAACCAGAATGG AGAACGCAGTGGGGCGCGAT CAAAACAACGTCGGCCCCAA 28400
28401 GGTTTACCCAATAATACTGC GTCTTGGTTCACCGCTCTCA CTCAACATGGCAAGGAAGAC CTTAAATTCCCTCGAGGACA AGGCGTTCCAATTAACACCA 28500
28501 ATAGCAGCCCAGATGACCAA ATTGGCTACTACCGAAGAGC TACCAGACGAATTCGTGGTG GTGACGGTAAAATGAAAGAT CTCAGTCCAAGATGGTATTT 28600
28601 CTACTACCTAGGAACTGGGC CAGAAGCTGGACTTCCCTAT GGTGCTAACAAAGACGGCAT CATATGGGTTGCAACTGAGG GAGCCTTGAATACACCAAAA 28700
28701 GATCACATTGGCACCCGCAA TCCTGCTAACAATGCTGCAA TCGTGCTACAACTTCCTCAA GGAACAACATTGCCCAAAGG CTTCTACGCAGAAGGGAGCA 28800
28801 GAGGCGGCAGTCAAGCCTCT TCTCGTTCCTCATCACGTAG TCGCAACAGTTCAAGAAATT CAACTCCAGGCAGCAGTAGG GGAACTTCTCCTGCTAGAAT 28900
28901 GGCTGGCAATGGCGGTGATG CTGCTCTTGCTTTGCTGCTG CTTGACAGATTGAACCAGCT TGAGAGCAAAATGTCTGGTA AAGGCCAACAACAACAAGGC 29000
29001 CAAACTGTCACTAAGAAATC TGCTGCTGAGGCTTCTAAGA AGCCTCGGCAAAAACGTACT GCCCCTAAAGCATACAATGT AACACAAGCTTTCGGCAGAC 29100
29101 GTGGTCCAGAACAAACCCAA GGAAATTTTGGGGACCAGGA ACTAATCAGACAAGGAACTG ATTACAAACATTGGCCGCAA ATTGCACAATTTGCCCCCAG 29200
29201 CGCTTCAGCGTTCTTCGGAA TGTCGCGCATTGGCATGGAA GTCACACCTTCGGGAACGTG GTTGACCTACACAGGTGCCA TCAAATTGGATGACAAAGAT 29300
29301 CCAAATTTCAAAGATCAAGT CATTTTGCTGAATAAGCATA TTGACGCATACAAAACATTC CCACCAACAGAGCCTAAAAA GGACAAAAAGAAGAAGGCTG 29400
29401 ATGAAACTCAAGCCTTACCG CAGAGACAGAAGAAACAGCA AACTGTGACTCTTTTTCCTG CTGCAGATTTGGATGATTTC TCCAAACAATTGCAACAATC 29500
29501 CATGAGCAGTGCTGACTCAA CTCAGGCCTAAACTCATGCA GACCACACAAGGCAGATGGG CTATATAAACGTTTTCGCTT TTCCGTTTACGATATATAGT 29600
29601 CTACTCTTGTGCAGAATGAA TTCTCGTAACTACATAGCAC AAGTAGATGTAGTTAACTTT AATCTCACATAGCAATCTTT AATCAGTGTGTAACATTAGG 29700
29701 GAGGACTTGAAAGAGCCACC ACATTTTCACCGAGGCCACG CGGAGTACGATCGAGTGTAC AGTGAACAATGCTAGGGAGA GCTGCCTATATGGAAGAGCC 29800

29801 CTAATGTGTAAAATTAATTT TAGTAGTGCTATCCCCAAGT CGGATTAATAGCTTCTTAGG AGAATGACAAAAAAAAAAAA AAAAAAAAA 29889

1.8. SRR11092062 Discovered Strain 2 Nucleotide Sequence

1 ATTAAAGGTTTATACCTTCC CAGGTAACAAACCAACCAAC TTTCGATCTCTTGTAGATCT GTTCTCTAAACGAACTTTAA AATCTGTGTGGCTGTCACTC 100

101 GGCTGCATGCTTAGTGCACT CACGCAGTATAATTAATAAC TAATTACTGTCGTTGACAGG ACACGAGTAACTCGTCTATC TTCTGCAGGCTGCTTACGGT 200

201 TTCGTCCGTGTTGCAGCCGA TCATCAGCACATCTAGGTTT CGTCCGGGTGTGACCGAAAG GTAAGATGGAGAGCCTTGTC CCTGGTTTCAACGAGAAAAC 300

301 ACACGTCCAACTCAGTTTGC CTGTTTTACAGGTTCGCGAC GTGCTCGTACGTGGCTTTGG AGACTCCGTGGAGGAGGTCT TATCAGAGGCACGTCAACAT 400

401 CTTAAAGATGGCACTTGTGG CTTAGTAGAAGTTGAAAAAG GCGTTTTGCCTCAACTTGAA CAGCCCTATGTGTTCATCAA ACGTTCGGATGCTCGAACTG 500

501 CACCTCATGGTCATGTTATG GTTGAGCTGGTAGCAGAACT CGAAGGCATTCAGTACGGTC GTAGTGGTGAGACACTTGGT GTCCTTGTCCCTCATGTGGG 600

601 CGAAATACCAGTGGCTTACC GCAAGGTTCTTCTTCGTAAG AACGGTAATAAAGGAGCTGG TGGCCATAGTTACGGCGCCG ATCTAAAGTCATTTGACTTA 700

701 GGCGACGAGCTTGGCACTGA TCCTTATGAAGATTTTCAAG AAAACTGGAACACTAAACAT AGCAGTGGTGTTACCCGTGA ACTCATGCGTGAGCTTAACG 800

801 GAGGGGCATACACTCGCTAT GTCGATAACAACTTCTGTGG CCCTGATGGCTACCCTCTTG AGTGCATTAAAGACCTTCTA GCACGTGCTGGTAAAGCTTC 900

901 ATGCACTTTGTCCGAACAAC TGGACTTTATTGACACTAAG AGGGGTGTATACTGCTGCCG TGAACATGAGCATGAAATTG CTTGGTACACGGAACGTTCT 1000

1001 GAAAAGAGCTATGAATTGCA GACACCTTTTGAAATTAAAT TGGCAAAGAAATTTGACACC TTCAATGGGGAATGTCCAAA TTTTGTATTTCCCTTAAATT 1100

1101 CCATAATCAAGACTATTCAA CCAAGGGTTGAAAAGAAAAA GCTTGATGGCTTTATGGGTA GAATTCGATCTGTCTATCCA GTTGCGTCACCAAATGAATG 1200

1201 CAACCAAATGTGCCTTTCAA CTCTCATGAAGTGTGATCAT TGTGGTGAAACTTCATGGCA GACGGGCGATTTTGTTAAAG CCACTTGCGAATTTTGTGGC 1300

1301 ACTGAGAATTTGACTAAAGA AGGTGCCACTACTTGTGGTT ACTTACCCCAAAATGCTGTT GTTAAAATTTATTGTCCAGC ATGTCACAATTCAGAAGTAG 1400

1401 GACCTGAGCATAGTCTTGCC GAATACCATAATGAATCTGG CTTGAAAACCATTCTTCGTA AGGGTGGTCGCACTATTGCC TTTGGAGGCTGTGTGTTCTC 1500

1501 TTATGTTGGTTGCCATAACA AGTGTGCCTATTGGGTTCCA CGTGCTAGCGCTAACATAGG TTGTAACCATACAGGTGTTG TTGGAGAAGGTTCCGAAGGT 1600

1601 CTTAATGACAACCTTCTTGA AATACTCCAAAAAGAGAAAG TCAACATCAATATTGTTGGT GACTTTAAACTTAATGAAGA GATCGCCATTATTTTGGCAT 1700

1701 CTTTTTCTGCTTCCACAAGT GCTTTTGTGGAAACTGTGAA AGGTTTGGATTATAAAGCAT TCAAACAAATTGTTGAATCC TGTGGTAATTTTAAAGTTAC 1800

1801 AAAAGGAAAAGCTAAAAAAG GTGCCTGGAATATTGGTGAA CAGAAATCAATACTGAGTCC TCTTTATGCATTTGCATCAG AGGCTGCTCGTGTTGTACGA 1900

1901 TCAATTTTCTCCCGCACTCT TGAAACTGCTCAAAATTCTG TGCGTGTTTTACAGAAGGCC GCTATAACAATACTAGATGG AATTTCACAGTATTCACTGA 2000

2001 GACTCATTGATGCTATGATG TTCACATCTGATTTGGCTAC TAACAATCTAGTTGTAATGG CCTACATTACAGGTGGTGTT GTTCAGTTGACTTCGCAGTG 2100

2101 GCTAACTAACATCTTTGGCA CTGTTTATGAAAAACTCAAA CCCGTCCTTGATTGGCTTGA AGAGAAGTTTAAGGAAGGTG TAGAGTTTCTTAGAGACGGT 2200

2201 TGGGAAATTGTTAAATTTAT CTCAACCTGTGCTTGTGAAA TTGTCGGTGGACAAATTGTC ACCTGTGCAAAGGAAATTAA GGAGAGTGTTCAGACATTCT 2300

2301 TTAAGCTTGTAAATAAATTT TTGGCTTTGTGTGCTGACTC TATCATTATTGGTGGAGCTA AACTTAAAGCCTTGAATTTA GGTGAAACATTTGTCACGCA 2400

2401 CTCAAAGGGATTGTACAGAA AGTGTGTTAAATCCAGAGAA GAAACTGGCCTACTCATGCC TCTAAAAGCCCCAAAAGAAA TTATCTTCTTAGAGGGAGAA 2500

2501 ACACTTCCCACAGAAGTGTT AACAGAGGAAGTTGTCTTGA AAACTGGTGATTTACAACCA TTAGAACAACCTACTAGTGA AGCTGTTGAAGCTCCATTGG 2600

2601 TTGGTACACCAGTTTGTATT AACGGGCTTATGTTGCTCGA AATCAAAGACACAGAAAAGT ACTGTGCCCTTGCACCTAAT ATGATGGTAACAAACAATAC 2700

2701 CTTCACACTCAAAGGCGGTG CACCAACAAAGGTTACTTTT GGTGATGACACTGTGATAGA AGTGCAAGGTTACAAGAGTG TGAATATCACTTTTGAACTT 2800

2801 GATGAAAGGATTGATAAAGT ACTTAATGAGAAGTGCTCTG CCTATACAGTTGAACTCGGT ACAGAAGTAAATGAGTTCGC CTGTGTTGTGGCAGATGCTG 2900

2901 TCATAAAAACTTTGCAACCA GTATCTGAATTACTTACACC ACTGGGCATTGATTTAGATG AGTGGAGTATGGCTACATAC TACTTATTTGATGAGTCTGG 3000

3001 TGAGTTTAAATTGGCTTCAC ATATGTATTGTTCTTTCTAC CCTCCAGATGAGGATGAAGA AGAAGGTGATTGTGAAGAAG AAGAGTTTGAGCCATCAACT 3100

3101 CAATATGAGTATGGTACTGA AGATGATTACCAAGGTAAAC CTTTGGAATTTGGTGCCACT TCTGCTGCTCTTCAACCTGA AGAAGAGCAAGAAGAAGATT 3200

3201 GGTTAGATGATGATAGTCAA CAAACTGTTGGTCAACAAGA CGGCAGTGAGGACAATCAGA CAACTACTATTCAAACAATT GTTGAGGTTCAACCTCAATT 3300

3301 AGAGATGGAACTTACACCAG TTGTTCAGACTATTGAAGTG AATAGTTTTAGTGGTTATTT AAAACTTACTGACAATGTAT ACATTAAAAATGCAGACATT 3400

3401 GTGGAAGAAGCTAAAAAGGT AAAACCAACAGTGGTTGTTA ATGCAGCCAATGTTTACCTT AAACATGGAGGAGGTGTTGC AGGAGCCTTAAATAAGGCTA 3500

3501 CTAACAATGCCATGCAAGTT GAATCTGATGATTACATAGC TACTAATGGACCACTTAAAG TGGGTGGTAGTTGTGTTTTA AGCGGACACAATCTTGCTAA 3600

3601 ACACTGTCTTCATGTTGTCG GCCCCAATGTTAACAAAGGT GAAGACATTCAACTTCTTAA GAGTGCTTATGAAAATTTTA ATCAGCACGAAGTTCTACTT 3700

3701 GCACCATTATTATCAGCTGG TATTTTTGGTGCTGACCCTA TACATTCTTTAAGAGTTTGT GTAGATACTGTTCGCACAAA TGTCTACTTAGCTGTCTTTG 3800

3801 ATAAAAATCTCTATGACAAA CTTGTTTCAAGCTTTTTGGA AATGAAGAGTGAAAAGCAAG TTGAACAAAAGATCGCTGAG ATTCCTAAAGAGGAAGTTAA 3900

3901 GCCATTTATAACTGAAAGTA AACCTTCAGTTGAACAGAGA AAACAAGATGATAAGAAAAT CAAAGCTTGTGTTGAAGAAG TTACAACAACTCTGGAAGAA 4000

4001 ACTAAGTTCCTCACAGAAAA CTTGTTACTTTATATTGACA TTAATGGCAATCTTCATCCA GATTCTGCCACTCTTGTTAG TGACATTGACATCACTTTCT 4100

4101 TAAAGAAAGATGCTCCATAT ATAGTGGGTGATGTTGTTCA AGAGGGTGTTTTAACTGCTG TGGTTATACCTACTAAAAAG GCTGGTGGCACTACTGAAAT 4200

4201 GCTAGCGAAAGCTTTGAGAA AAGTGCCAACAGACAATTAT ATAACCACTTACCCGGGTCA GGGTTTAAATGGTTACACTG TAGAGGAGGCTAAGACAGTG 4300

4301 CTTAAAAAGTGTAAAAGTGC CTTTTACATTCTACCATCTA TTATCTCTAATGAGAAGCAA GAAATTCTTGGAACTGTTTC TTGGAATTTGCGAGAAATGC 4400

4401 TTGCACATGCAGAAGAAACA CGCAAATTAATGCCTGTCTG TGTGGAAACTAAAGCCATAG TTTCAACTATACAGCGTAAA TATAAGGGTATTAAAATACA 4500

4501 AGAGGGTGTGGTTGATTATG GTGCTAGATTTTACTTTTAC ACCAGTAAAACAACTGTAGC GTCACTTATCAACACACTTA ACGATCTAAATGAAACTCTT 4600

4601 GTTACAATGCCACTTGGCTA TGTAACACATGGCTTAAATT TGGAAGAAGCTGCTCGGTAT ATGAGATCTCTCAAAGTGCC AGCTACAGTTTCTGTTTCTT 4700

4701 CACCTGATGCTGTTACAGCG TATAATGGTTATCTTACTTC TTCTTCTAAAACACCTGAAG AACATTTTATTGAAACCATC TCACTTGCTGGTTCCTATAA 4800

4801 AGATTGGTCCTATTCTGGAC AATCTACACAACTAGGTATA GAATTTCTTAAGAGAGGTGA TAAAAGTGTATATTACACTA GTAATCCTACCACATTCCAC 4900

4901 CTAGATGGTGAAGTTATCAC CTTTGACAATCTTAAGACAC TTCTTTCTTTGAGAGAAGTG AGGACTATTAAGGTGTTTAC AACAGTAGACAACATTAACC 5000

5001 TCCACACGCAAGTTGTGGAC ATGTCAATGACATATGGACA ACAGTTTGGTCCAACTTATT TGGATGGAGCTGATGTTACT AAAATAAAACCTCATAATTC 5100

5101 ACATGAAGGTAAAACATTTT ATGTTTTACCTAATGATGAC ACTCTACGTGTTGAGGCTTT TGAGTACTACCACACAACTG ATCCTAGTTTTCTGGGTAGG 5200

5201 TACATGTCAGCATTAAATCA CACTAAAAAGTGGAAATACC CACAAGTTAATGGTTTAACT TCTATTAAATGGGCAGATAA CAACTGTTATCTTGCCACTG 5300

5301 CATTGTTAACACTCCAACAA ATAGAGTTGAAGTTTAATCC ACCTGCTCTACAAGATGCTT ATTACAGAGCAAGGGCTGGT GAAGCTGCTAACTTTTGTGC 5400

5401 ACTTATCTTAGCCTACTGTA ATAAGACAGTAGGTGAGTTA GGTGATGTTAGAGAAACAAT GAGTTACTTGTTTCAACATG CCAATTTAGATTCTTGCAAA 5500

5501 AGAGTCTTGAACGTGGTGTG TAAAACTTGTGGACAACAGC AGACAACCCTTAAGGGTGTA GAAGCTGTTATGTACATGGG CACACTTTCTTATGAACAAT 5600

5601 TTAAGAAAGGTGTTCAGATA CCTTGTACGTGTGGTAAACA AGCTACAAAATATCTAGTAC AACAGGAGTCACCTTTTGTT ATGATGTCAGCACCACCTGC 5700

5701 TCAGTATGAACTTAAGCATG GTACATTTACTTGTGCTAGT GAGTACACTGGTAATTACCA GTGTGGTCACTATAAACATA TAACTTCTAAAGAAACTTTG 5800

5801 TATTGCATAGACGGTGCTTT ACTTACAAAGTCCTCAGAAT ACAAAGGTCCTATTACGGAT GTTTTCTACAAAGAAAACAG TTACACAACAACCATAAAAC 5900

5901 CAGTTACTTATAAATTGGAT GGTGTTGTTTGTACAGAAAT TGACCCTAAGTTGGACAATT ATTATAAGAAAGACAATTCT TATTTCACAGAGCAACCAAT 6000

6001 TGATCTTGTACCAAACCAAC CATATCCAAACGCAAGCTTC GATAATTTTAAGTTTGTATG TGATAATATCAAATTTGCTG ATGATTTAAACCAGTTAACT 6100

6101 GGTTATAAGAAACCTGCTTC AAGAGAGCTTAAAGTTACAT TTTTCCCTGACTTAAATGGA AGACCGCTTGGCCTCCGACT TAAACACTACACACCCTCTT 6200

6201 TTAAGAAAGGAGCTAAATTG TTACATAAACCTATTGTTTG GCATGTTAACAATGCAACTA ATAAAGCCACGTATAAACCA AATACCTGGTGTATACGTTG 6300

6301 TCTTTGGAGCACAAAACCAG TTGAAACATCAAATTCGTTT GATGTACTGAAGTCAGAGGA CGCGCAGGGAATGGATAATC TTGCCTGCGAAGATCTAAAA 6400

6401 CCAGTCTCTGAAGAAGTAGT GGAAAATCCTACCATACAGA AAGACGTTCTTGAGTGTAAT GTGAAAACTACCGAAGTTGT AGGAGACATTATACTTAAAC 6500

6501 CAGCAAATAATAGTTTAAAA ATTACAGAAGAGGTTGGCCA CACAGATCTAATGGCTGCTT ATGTAGACAATTCTAGTCTT ACTATTAAGAAACCTAATGA 6600

6601 ATTATCTAGAGTATTAGGTT TGAAAACCCTTGCTACTCAT GGTTTAGCTGCTGTTAATAG TGTCCCTTGGGATACTATAG CTAATTATGCTAAGCCTTTT 6700

6701 CTTAACAAAGTTGTTAGTAC AACTACTAACATAGTTACAC GGTGTTTAAACCGTGTTTGT ACTAATTATATGCCTTATTT CTTTACTTTATTGCTACAAT 6800

6801 TGTGTACTTTTACTAGAAGT ACAAATTCTAGAATTAAAGC ATCTATGCCGACTACTATAG CAAAGAATACTGTTAAGAGT GTCGGTAAATTTTGTCTAGA 6900

6901 GGCTTCATTTAATTATTTGA AGTCACCTAATTTTTCTAAA CTGATAAATATTATAATTTG GTTTTTACTATTAAGTGTTT GCCTAGGTTCTTTAATCTAC 7000

7001 TCAACCGCTGCTTTAGGTGT TTTAATGTCTAATTTAGGCA TGCCTTCTTACTGTACTGGT TACAGAGAAGGCTATTTGAA CTCTACTAATGTCACTATTG 7100

7101 CAACCTACTGTACTGGTTCT ATACCTTGTAGTGTTTGTCT TAGTGGTTTAGATTCTTTAG ACACCTATCCTTCTTTAGAA ACTATACAAATTACCATTTC 7200

7201 ATCTTTTAAATGGGATTTAA CTGCTTTTGGCTTAGTTGCA GAGTGGTTTTTGGCATATAT TCTTTTCACTAGGTTTTTCT ATGTACTTGGATTGGCTGCA 7300

7301 ATCAAGCAATTGTTTTTCAG CTATTTTGCAGTACATTTTA TTAGTAATTCTTGGCTTATG TGGTTAATAATTAATCTTGT ACAAATGGCCCCGATTTCAG 7400

7401 CTATGGTTAGAATGTACATC TTCTTTGCATCATTTTATTA TGTATGGAAAAGTTATGTGC ATGTTGTAGACGGTTGTAAT TCATCAACTTGTATGATGTG 7500

7501 TTACAAACGTAATAGAGCAA CAAGAGTCGAATGTACAACT ATTGTTAATGGTGTTAGAAG GTCCTTTTATGTCTATGCTA ATGGAGGTAAAGGCTTTTGC 7600

7601 AAACTACACAATTGGAATTG TGTTAATTGTGATACATTCT GTGCTGGTAGTACATTTATT AGTGATGAAGTTGCGAGAGA CTTGTCACTACAGTTTAAAA 7700

7701 GACCAATAAATCCTACTGAC CAGTCTTCTTACATCGTTGA TAGTGTTACAGTGAAGAATG GTTCCATCCATCTTTACTTT GATAAAGCTGGTCAAAAGAC 7800

7801 TTATGAAAGACATTCTCTCT CTCATTTTGTTAACTTAGAC AACCTGAGAGCTAATAACAC TAAAGGTTCATTGCCTATTA ATGTTATAGTTTTTGATGGT 7900

7901 AAATCAAAATGTGAAGAATC ATCTGCAAAATCAGCGTCTG TTTACTACAGTCAGCTTATG TGTCAACCTATACTGTTACT AGATCAGGCATTAGTGTCTG 8000

8001 ATGTTGGTGATAGTGCGGAA GTTGCAGTTAAAATGTTTGA TGCTTACGTTAATACGTTTT CATCAACTTTTAACGTACCA ATGGAAAAACTCAAAACACT 8100

8101 AGTTGCAACTGCAGAAGCTG AACTTGCAAAGAATGTGTCC TTAGACAATGTCTTATCTAC TTTTATTTCAGCAGCTCGGC AAGGGTTTGTTGATTCAGAT 8200

8201 GTAGAAACTAAAGATGTTGT TGAATGTCTTAAATTGTCAC ATCAATCTGACATAGAAGTT ACTGGCGATAGTTGTAATAA CTATATGCTCACCTATAACA 8300

8301 AAGTTGAAAACATGACACCC CGTGACCTTGGTGCTTGTAT TGACTGTAGTGCGCGTCATA TTAATGCGCAGGTAGCAAAA AGTCACAACATTGCTTTGAT 8400

8401 ATGGAACGTTAAAGATTTCA TGTCATTGTCTGAACAACTA CGAAAACAAATACGTAGTGC TGCTAAAAAGAATAACTTAC CTTTTAAGTTGACATGTGCA 8500

8501 ACTACTAGACAAGTTGTTAA TGTTGTAACAACAAAGATAG CACTTAAGGGTGGTAAAATT GTTAATAATTGGTTGAAGCA GTTAATTAAAGTTACACTTG 8600

8601 TGTTCCTTTTTGTTGCTGCT ATTTTCTATTTAATAACACC TGTTCATGTCATGTCTAAAC ATACTGACTTTTCAAGTGAA ATCATAGGATACAAGGCTAT 8700

8701 TGATGGTGGTGTCACTCGTG ACATAGCATCTACAGATACT TGTTTTGCTAACAAACATGC TGATTTTGACACATGGTTTA GCCAGCGTGGTGGTAGTTAT 8800

8801 ACTAATGACAAAGCTTGCCC ATTGATTGCTGCAGTCATAA CAAGAGAAGTGGGTTTTGTC GTGCCTGGTTTGCCTGGCAC GATATTACGCACAACTAATG 8900

8901 GTGACTTTTTGCATTTCTTA CCTAGAGTTTTTAGTGCAGT TGGTAACATCTGTTACACAC CATCAAAACTTATAGAGTAC ACTGACTTTGCAACATCAGC 9000

9001 TTGTGTTTTGGCTGCTGAAT GTACAATTTTTAAAGATGCT TCTGGTAAGCCAGTACCATA TTGTTATGATACCAATGTAC TAGAAGGTTCTGTTGCTTAT 9100

9101 GAAAGTTTACGCCCTGACAC ACGTTATGTGCTCATGGATG GCTCTATTATTCAATTTCCT AACACCTACCTTGAAGGTTC TGTTAGAGTGGTAACAACTT 9200

9201 TTGATTCTGAGTACTGTAGG CACGGCACTTGTGAAAGATC AGAAGCTGGTGTTTGTGTAT CTACTAGTGGTAGATGGGTA CTTAACAATGATTATTACAG 9300

9301 ATCTTTACCAGGAGTTTTCT GTGGTGTAGATGCTGTAAAT TTACTTACTAATATGTTTAC ACCACTAATTCAACCTATTG GTGCTTTGGACATATCAGCA 9400

9401 TCTATAGTAGCTGGTGGTAT TGTAGCTATCGTAGTAACAT GCCTTGCCTACTATTTTATG AGGTTTAGAAGAGCTTTTGG TGAATACAGTCATGTAGTTG 9500

9501 CCTTTAATACTTTACTATTC CTTATGTCATTCACTGTACT CTGTTTAACACCAGTTTACT CATTCTTACCTGGTGTTTAT TCTGTTATTTACTTGTACTT 9600

9601 GACATTTTATCTTACTAATG ATGTTTCTTTTTTAGCACAT ATTCAGTGGATGGTTATGTT CACACCTTTAGTACCTTTCT GGATAACAATTGCTTATATC 9700

9701 ATTTGTATTTCCACAAAGCA TTTCTATTGGTTCTTTAGTA ATTACCTAAAGAGACGTGTA GTCTTTAATGGTGTTTCCTT TAGTACTTTTGAAGAAGCTG 9800

9801 CGCTGTGCACCTTTTTGTTA AATAAAGAAATGTATCTAAA GTTGCGTAGTGATGTGCTAT TACCTCTTACGCAATATAAT AGATACTTAGCTCTTTATAA 9900

9901 TAAGTACAAGTATTTTAGTG GAGCAATGGATACAACTAGC TACAGAGAAGCTGCTTGTTG TCATCTCGCAAAGGCTCTCA ATGACTTCAGTAACTCAGGT 10000

10001 TCTGATGTTCTTTACCAACC ACCACAAACCTCTATCACCT CAGCTGTTTTGCAGAGTGGT TTTAGAAAAATGGCATTCCC ATCTGGTAAAGTTGAGGGTT 10100

10101 GTATGGTACAAGTAACTTGT GGTACAACTACACTTAACGG TCTTTGGCTTGATGACGTAG TTTACTGTCCAAGACATGTG ATCTGCACCTCTGAAGACAT 10200

10201 GCTTAACCCTAATTATGAAG ATTTACTCATTCGTAAGTCT AATCATAATTTCTTGGTACA GGCTGGTAATGTTCAACTCA GGGTTATTGGACATTCTATG 10300

10301 CAAAATTGTGTACTTAAGCT TAAGGTTGATACAGCCAATC CTAAGACACCTAAGTATAAG TTTGTTCGCATTCAACCAGG ACAGACTTTTTCAGTGTTAG 10400

10401 CTTGTTACAATGGTTCACCA TCTGGTGTTTACCAATGTGC TATGAGGCCCCATTTCACTA TTAAGGGTTCATTCCTTAAT GGTTCATGTGGTAGTGTTGG 10500

10501 TTTTAACATAGATTATGACT GTGTCTCTTTTTGTTACATG CACCATATGGAATTACCAAC TGGAGTTCATGCTGGCACAG ACTTAGAAGGTAACTTTTAT 10600

10601 GGACCTTTTGTTGACAGGCA AACAGCACAAGCAGCTGGTA CGGACACAACTATTACAGTT AATGTTTTAGCTTGGTTGTA CGCTGCTGTTATAAATGGAG 10700

10701 ACAGGTGGTTTCTCAATCGA TTTACCACAACTCTTAATGA CTTTAACCTTGTGGCTATGA AGTACAATTATGAACCTCTA ACACAAGACCATGTTGACAT 10800

10801 ACTAGGACCTCTTTCTGCTC AAACTGGAATTGCCGTTTTA GATATGTGTGCTTCATTAAA AGAATTACTGCAAAATGGTA TGAATGGACGTACCATATTG 10900

10901 GGTAGTGCTTTATTAGAAGA TGAATTTACACCTTTTGATG TTGTTAGACAATGCTCAGGT GTTACTTTCCAAAGTGCAGT GAAAAGAACAATCAAGGGTA 11000

11001 CACACCACTGGTTGTTACTC ACAATTTTGACTTCACTTTT AGTTTTAGTCCAGAGTACTC AATGGTCTTTGTTCTTTTTT TTGTATGAAAATGCCTTTTT 11100

11101 ACCTTTTGCTATGGGTATTA TTGCTATGTCTGCTTTTGCA ATGATGTTTGTCAAACATAA GCATGCATTTCTCTGTTTGT TTTTGTTACCTTCTCTTGCC 11200

11201 ACTGTAGCTTATTTTAATAT GGTCTATATGCCTGCTAGTT GGGTGATGCGTATTATGACA TGGTTGGATATGGTTGATAC TAGTTTGTCTGGTTTTAAGC 11300

11301 TAAAAGACTGTGTTATGTAT GCATCAGCTGTAGTGTTACT AATCCTTATGACAGCAAGAA CTGTGTATGATGATGGTGCT AGGAGAGTGTGGACACTTAT 11400

11401 GAATGTCTTGACACTCGTTT ATAAAGTTTATTATGGTAAT GCTTTAGATCAAGCCATTTC CATGTGGGCTCTTATAATCT CTGTTACTTCTAACTACTCA 11500

11501 GGTGTAGTTACAACTGTCAT GTTTTTGGCCAGAGGTATTG TTTTTATGTGTGTTGAGTAT TGCCCTATTTTCTTCATAAC TGGTAATACACTTCAGTGTA 11600

11601 TAATGCTAGTTTATTGTTTC TTAGGCTATTTTTGTACTTG TTACTTTGGCCTCTTTTGTT TACTCAACCGCTACTTTAGA CTGACTCTTGGTGTTTATGA 11700

11701 TTACTTAGTTTCTACACAGG AGTTTAGATATATGAATTCA CAGGGACTACTCCCACCCAA GAATAGCATAGATGCCTTCA AACTCAACATTAAATTGTTG 11800

11801 GGTGTTGGTGGCAAACCTTG TATCAAAGTAGCCACTGTAC AGTCTAAAATGTCAGATGTA AAGTGCACATCAGTAGTCTT ACTCTCAGTTTTGCAACAAC 11900

11901 TCAGAGTAGAATCATCATCT AAATTGTGGGCTCAATGTGT CCAGTTACACAATGACATTC TCTTAGCTAAAGATACTACT GAAGCCTTTGAAAAAATGGT 12000

12001 TTCACTACTTTCTGTTTTGC TTTCCATGCAGGGTGCTGTA GACATAAACAAGCTTTGTGA AGAAATGCTGGACAACAGGG CAACCTTACAAGCTATAGCC 12100

12101 TCAGAGTTTAGTTCCCTTCC ATCATATGCAGCTTTTGCTA CTGCTCAAGAAGCTTATGAG CAGGCTGTTGCTAATGGTGA TTCTGAAGTTGTTCTTAAAA 12200

12201 AGTTGAAGAAGTCTTTGAAT GTGGCTAAATCTGAATTTGA CCGTGATGCAGCCATGCAAC GTAAGTTGGAAAAGATGGCT GATCAAGCTATGACCCAAAT 12300

12301 GTATAAACAGGCTAGATCTG AGGACAAGAGGGCAAAAGTT ACTAGTGCTATGCAGACAAT GCTTTTCACTATGCTTAGAA AGTTGGATAATGATGCACTC 12400

12401 AACAACATTATCAACAATGC AAGAGATGGTTGTGTTCCCT TGAACATAATACCTCTTACA ACAGCAGCCAAACTAATGGT TGTCATACCAGACTATAACA 12500

12501 CATATAAAAATACGTGTGAT GGTACAACATTTACTTATGC ATCAGCATTGTGGGAAATCC AACAGGTTGTAGATGCAGAT AGTAAAATTGTTCAACTTAG 12600

12601 TGAAATTAGTATGGACAATT CACCTAATTTAGCATGGCCT CTTATTGTAACAGCTTTAAG GGCCAATTCTGCTGTCAAAT TACAGAATAATGAGCTTAGT 12700

12701 CCTGTTGCACTACGACAGAT GTCTTGTGCTGCCGGTACTA CACAAACTGCTTGCACTGAT GACAATGCGTTAGCTTACTA CAACACAACAAAGGGAGGTA 12800

12801 GGTTTGTACTTGCACTGTTA TCCGATTTACAGGATTTGAA ATGGGCTAGATTCCCTAAGA GTGATGGAACTGGTACTATC TATACAGAACTGGAACCACC 12900

12901 TTGTAGGTTTGTTACAGACA CACCTAAAGGTCCTAAAGTG AAGTATTTATACTTTATTAA AGGATTAAACAACCTAAATA GAGGTATGGTACTTGGTAGT 13000

13001 TTAGCTGCCACAGTACGTCT ACAAGCTGGTAATGCAACAG AAGTGCCTGCCAATTCAACT GTATTATCTTTCTGTGCTTT TGCTGTAGATGCTGCTAAAG 13100

13101 CTTACAAAGATTATCTAGCT AGTGGGGGACAACCAATCAC TAATTGTGTTAAGATGTTGT GTACACACACTGGTACTGGT CAGGCAATAACAGTTACACC 13200

13201 GGAAGCCAATATGGATCAAG AATCCTTTGGTGGTGCATCG TGTTGTCTGTACTGCCGTTG CCACATAGATCATCCAAATC CTAAAGGATTTTGTGACTTA 13300

13301 AAAGGTAAGTATGTACAAAT ACCTACAACTTGTGCTAATG ACCCTGTGGGTTTTACACTT AAAAACACAGTCTGTACCGT CTGCGGTATGTTGAAAGGTT 13400

13401 ATGGCTGTAGTTGTGATCAA CTCCGCGAACCCATGCTTCA GTCAGCTGATGCACAATCGT TTTTAAACGGGTTTGCGGTG TAAGTGCAGCCCGTCTTACA 13500

13501 CCGTGCGGCACAGGCACTAG TACTGATGTCGTATACAGGG CTTTTGACATCTACAATGAT AAAGTAGCTGGTTTTGCTAA ATTCCTAAAAACTAATTGTT 13600

13601 GTCGCTTCCAAGAAAAGGAC GAAGATGACAATTTAATTGA TTCTTACTTTGTAGTTAAGA GACACACTTTCTCTAACTAC CAACATGAAGAAACAATTTA 13700

13701 TAATTTACTTAAGGATTGTC CAGCTGTTGCTAAACATGAC TTCTTTAAGTTTAGAATAGA CGGTGACATGGTACCACATA TATCACGTCAACGTCTTACT 13800

13801 AAATACACAATGGCAGACCT CGTCTATGCTTTAAGGCATT TTGATGAAGGTAATTGTGAC ACATTAAAAGAAATACTTGT CACATACAATTGTTGTGATG 13900

13901 ATGATTATTTCAATAAAAAG GACTGGTATGATTTTGTAGA AAACCCAGAAGTCGGATCGT AATACGCCAACTTAGGTGAA CGTGTACGCCAAGCTTTGTT 14000

14001 AAAAACAGTACAATTCTGTG ATGCCATGCGAAATGCTGGT ATTGTTGGTGTACTGACATT AGATAATCAAGATCTCAATG GTAACTGGTATGATTTCGGT 14100

14101 GATTTCATACAAACCACGCC AGGTAGTGGAGTTCCTGTTG TAGATTCTTATTATTCATTG TTAATGCCTATATTAACCTT GACCAGGGCTTTAACTGCAG 14200

14201 AGTCACATGTTGACACTGAC TTAACAAAGCCTTACATTAA GTGGGATTTGTTAAAATATG ACTTCACGGAAGAGAGGTTA AAACTCTTTGACCGTTATTT 14300

14301 TAAATATTGGGATCAGACAT ACCACCCAAATTGTGTTAAC TGTTTGGATGACAGATGCAT TCTGCATTGTGCAAACTTTA ATGTTTTATTCTCTACAGTG 14400

14401 TTCCCACCTACAAGTTTTGG ACCACTAGTGAGAAAAATAT TTGTTGATGGTGTTCCATTT GTAGTTTCAACTGGATACCA CTTCAGAGAGCTAGGTGTTG 14500

14501 TACATAATCAGGATGTAAAC TTACATAGCTCTAGACTTAG TTTTAAGGAATTACTTGTGT ATGCTGCTGACCCTGCTATG CACGCTGCTTCTGGTAATCT 14600

14601 ATTACTAGATAAACGCACTA CGTGCTTTTCAGTAGCTGCA CTTACTAACAATGTTGCTTT TCAAACTGTCAAACCCGGTA ATTTTAACAAAGACTTCTAT 14700

14701 GACTTTGCTGTGTCTAAGGG TTTCTTTAAGGAAGGAAGTT CTGTTGAATTAAAACACTTC TTCTTTGCTCAGGATGGTAA TGCTGCTATCAGCGATTATG 14800

14801 ACTACTATCGTTATAATCTA CCAACAATGTGTGATATCAG ACAACTACTATTTGTAGTTG AAGTTGTTGATAAGTACTTT GATTGTTACGATGGTGGCTG 14900

14901 TATTAATGCTAACCAAGTCA TCGTCAACAACCTAGACAAA TCAGCTGGTTTTCCATTTAA TAAATGGGGTAAGGCTAGAC TTTATTATGATTCAATGAGT 15000

15001 TATGAGGATCAAGATGCACT TTTCGCATATACAAAACGTA ATGTCATCCCTACTATAACT CAAATGAATCTTAAGTATGC CATTAGTGCAAAGAATAGAG 15100

15101 CTCGCACCGTAGCTGGTGTC TCTATCTGTAGTACTATGAC CAATAGACAGTTTCATCAAA AATTATTGAAATCAATAGCC GCCACTAGAGGAGCTACTGT 15200

15201 AGTAATTGGAACAAGCAAAT TCTATGGTGGTTGGCACAAC ATGTTAAAAACTGTTTATAG TGATGTAGAAAACCCTCACC TTATGGGTTGGGATTATCCT 15300

15301 AAATGTGATAGAGCCATGCC TAACATGCTTAGAATTATGG CCTCACTTGTTCTTGCTCGC AAACATACAACGTGTTGTAG CTTGTCACACCGTTTCTATA 15400

15401 GATTAGCTAATGAGTGTGCT CAAGTATTGAGTGAAATGGT CATGTGTGGCGGTTCACTAT ATGTTAAACCAGGTGGAACC TCATCAGGAGATGCCACAAC 15500

15501 TGCTTATGCTAATAGTGTTT TTAACATTTGTCAAGCTGTC ACGGCCAATGTTAATGCACT TTTATCTACTGATGGTAACA AAATTGCCGATAAGTATGTC 15600

15601 CGCAATTTACAACACAGACT TTATGAGTGTCTCTATAGAA ATAGAGATGTTGACACAGAC TTTGTGAATGAGTTTTACGC ATATTTGCGTAAACATTTCT 15700

15701 CAATGATGATACTCTCTGAC GATGCTGTTGTGTGTTTCAA TAGCACTTATGCATCTCAAG GTCTAGTGGCTAGCATAAAG AACTTTAAGTCAGTTCTTTA 15800

15801 TTATCAAAACAATGTTTTTA TGTCTGAAGCAAAATGTTGG ACTGAGACTGACCTTACTAA AGGACCTCATGAATTTTGCT CACAACATACAATGCTAGTT 15900

15901 AAACAGGGTGATGATTATGT GTACCTTCCTTACCCAGATC CATCAAGAATCCTAGGGGCC GGCTGTTTTGTAGATGATAT CGTAAAAACAGATGGTACAC 16000

16001 TTATGATTGAACGGTTCGTG TCTTTAGCTATAGATGCTTA CCCACTTACTAAACATCCTA ATCAGGAGTATGCTGATGTC TTTCATTTGTACTTACAATA 16100

16101 CATAAGAAAGCTACATGATG AGTTAACAGGACACATGTTA GACATGTATTCTGTTATGCT TACTAATGATAACACTTCAA GGTATTGGGAACCTGAGTTT 16200

16201 TATGAGGCTATGTACACACC GCATACAGTCTTACAGGCTG TTGGGGCTTGTGTTCTTTGC AATTCACAGACTTCATTAAG ATGTGGTGCTTGCATACGTA 16300

16301 GACCATTCTTATGTTGTAAA TGCTGTTACGACCATGTCAT ATCAACATCACATAAATTAG TCTTGTCTGTTAATCCGTAT GTTTGCAATGCTCCAGGTTG 16400

16401 TGATGTCACAGATGTGACTC AACTTTACTTAGGAGGTATG AGCTATTATTGTAAATCACA TAAACCACAAGTCGGTTTTC CATTGTGTGCTAATGGACAA 16500

16501 GTTTTTGGTTTATATAAAAA TACATGTGTTGGTAGCGATA ATGTTACTGACTTTAATGCA ATTGCAACATGTGACTGGAC AAATGCTGGTGATTACATTT 16600

16601 TAGCTAACACCTGTACTGAA AGACTCAAGCTTTTTGCAGC AGAAACGCTCAAAGCTACTG AGGAGACATTTAAACTGTCT TATGGTATTGCTACTGTACG 16700

16701 TGAAGTGCTGTCTGACAGAG AATTACATCTTTCATGGGAA GTTGGTAAACCTAGACCACC ACTTAACCGAAATTATGTCT TTACTGGTTATCGTGTAACT 16800

16801 AAAAACAGTAAAGTACAAAT AGGAGAGTACACCTTTGAAA AAGGTGACTATGGTGATGCT GTTGTTTACCGAGGTACAAC AACTTACAAATTAAATGTTG 16900

16901 GTGATTATTTTGTGCTGACA TCACATACAGTAATGCCATT AAGTGCACCTACACTAGTGC CACAAGAGCACTATGTTAGA ATTACTGGCTTATACCCAAC 17000

17001 ACTCAATATCTCAGATGAGT TTTCTAGCAATGTTGCAAAT TATCAAAAGGTTGGTATGCA AAAGTATTCTACACTCCAGG GACCACCTGGTACTGGTAAG 17100

17101 AGTCATTTTGCTATTGGCCT AGCTCTCTACTACCCTTCTG CTCGCATAGTGTATACAGCT TGCTCTCATGCCGCTGTTGA TGCACTATGTGAGAAGGCAT 17200

17201 TAAAATATTTGCCTATAGAT AAATGTAGTAGAATTATACC TGCACGTGCTCGTGTAGAGT GTTTTGATAAATTCAAAGTG AATTCAACATTAGAACAGTA 17300

17301 TGTCTTTTGTACTGTAAATG CATTGCCTGAGACGACAGCA GATATAGTTGTCTTTGATGA AATTTCAATGGCCGCAAATT ATGATTTGAGTGTTGTCAAT 17400

17401 GCCAGATTACGTGCTAAGCA CTATGTGTACATTGGCGACC CTGCTCAATTACCTGCACCA CGCACATTGCTAACTAAGGG CACACTAGAACCAGAATATT 17500

17501 TCAATTCAGTGTGTAGACTT ATGAAAACTATAGGTCCAGA CATGTTCCTCGGAACTTGTC GGCGTTGTCCTGCTGAAATT GTTGACACTGTGAGTGCTTT 17600

17601 GGTTTATGATAATAAGCTTA AAGCACATAAAGACAAATCA GCTCAATGCTTTAAAATGTT TTATAAGGGTGTTATCACGC ATGATGTTTCATCTGCAATT 17700

17701 AACAGGCCGCAAATAGGCGT GGTAAGAGAATTCCTTACAC GTAACCCTGCTTGGAGAAAA GCTGTCTTTATTTCACCTTA TAATTCACAGAATGCTGTAG 17800

17801 CCTCAAAGATTTTGGGACTA CCAACTCAAACTGTTGATTC ATCACAGGGCTCAGAATATG ACTATGTCATATTCACTCAA ACCACCGAAACAGCTCACTC 17900

17901 TTGTAATGTAAACAGATTTA ATGTTGCTATTACCAGAGCA AAAGTAGGCATACTTTGCAT AATGTCTGATAGAGACCTTT ATGACAAGTTGCAATTTACA 18000

18001 AGTCTTGAAATTCCACGTAG GAATGTGGCAACTTTACAAG CTGAAAATGTAACAGGACTC TTTAAAGATTGTAGTAAGGT AATCACTGGGTTACATCCTA 18100

18101 CACAGGCGCCTACACACCTC AGTGTTGACACTAAATTCAA AACTGAAGGTTTATGTGTTG ACATACCTGGCATACCTAAG GACATGACCTATAGAAGACT 18200

18201 CATCTCTATGATGGGTTTTA AAATGAATTATCAAGTTAAT GGTTACCCTAACATGTTTAT CACCCGCGAAGAAGCTATAA GACATGTACGTGCATGGATT 18300

18301 GGCTTCGATGTCGAGGGGTG TCATGCTACTAGAGAAGCTG TTGGTACCAATTTACCTTTA CAGCTAGGTTTTTCTACAGG TGTTAACCTAGTTGCTGTAC 18400

18401 CTACAGGTTATGTTGATACA CCTAATAATACAGATTTTTC CAGAGTTAGTGCTAAACCAC CGCCTGGAGATCAATTTAAA CACCTCATACCACTTATGTA 18500

18501 CAAAGGACTTCCTTGGAATG TAGTGCGTATAAAGATTGTA CAAATGTTAAGTGACACACT TAAAAATCTCTCTGACAGAG TCGTATTTGTCTTATGGGCA 18600

18601 CATGGCTTTGAGTTGACATC TATGAAGTATTTTGTGAAAA TAGGACCTGAGCGCACCTGT TGTCTATGTGATAGACGTGC CACATGCTTTTCCACTGCTT 18700

18701 CAGACACTTATGCCTGTTGG CATCATTCTATTGGATTTGA TTACGTCTATAATCCGTTTA TGATTGATGTTCAACAATGG GGTTTTACAGGTAACCTACA 18800

18801 AAGCAACCATGATCTGTATT GTCAAGTCCATGGTAATGCA CATGTAGCTAGTTGTGATGC AATCATGACTAGGTGTCTAG CTGTCCACGAGTGCTTTGTT 18900

18901 AAGCGTGTTGACTGGACTAT TGAATATCCTATAATTGGTG ATGAACTGAAGATTAATGCG GCTTGTAGAAAGGTTCAACA CATGGTTGTTAAAGCTGCAT 19000

19001 TATTAGCAGACAAATTCCCA GTTCTTCACGACATTGGTAA CCCTAAAGCTATTAAGTGTG TACCTCAAGCTGATGTAGAA TGGAAGTTCTATGATGCACA 19100

19101 GCCTTGTAGTGACAAAGCTT ATAAAATAGAAGAATTATTC TATTCTTATAAGTCGGATCG TAGCCATGTCGTTCTGTGTG TATGCCTATTTTGGAATTGC 19200

19201 AATGTCGATAGATATCCTGC TAATTCCATTGTTTGTAGAT TTGACACTAGAGTGCTATCT AACCTTAACTTGCCTGGTTG TGATGGTGGCAGTTTGTATG 19300

19301 TAAATAAACATGCATTCCAC ACACCAGCTTTTGATAAAAG TGCTTTTGTTAATTTAAAAC AATTACCATTTTTCTATTAC TCTGACAGTCCATGTGAGTC 19400

19401 TCATGGAAAACAAGTAGTGT CAGATATAGATTATGTACCA CTAAAGTCTGCTACGTGTAT AACACGTTGCAATTTAGGTG GTGCTGTCTGTAGACATCAT 19500

19501 GCTAATGAGTACAGATTGTA TCTCGATGCTTATAACATGA TGATCTCAGCTGGCTTTAGC TTGTGGGTTTACAAACAATT TGATACTTATAACCTCTGGA 19600

19601 ACACTTTTACAAGACTTCAG AGTTTAGAAAATGTGGCTTT TAATGTTGTAAATAAGGGAC ACTTTGATGGACAACAGGGT GAAGTACCAGTTTCTATCAT 19700

19701 TAATAACACTGTTTACACAA AAGTTGATGGTGTTGATGTA GAATTGTTTGAAAATAAAAC AACATTACCTGTTAATGTAG CATTTGAGCTTTGGGCTAAG 19800

19801 CGCAACATTAAACCAGTACC AGAGGTGAAAATACTCAATA ATTTGGGTGTGGACATTGCT GCTAATACTGTGATCTGGGA CTACAAAAGAGATGCCCCAG 19900

19901 CACATATATCTACTATTGGT GTTTGTTCTATGACTGACAT AGCCAAGAAACCAACTGAAA CGATTTGTGCACCACTCACT GTCTTTTTTGATGGTAGAGT 20000

20001 TGATGGTCAAGTAGACTTAT TTAGAAATGCCCGTAATGGT GTTCTTATTACAGAAGGTAG TGTTAAAGGTTTACAACCAT CTGTAGGTCCCAAACAAGCT 20100

20101 AGTCTTAATGGAGTCACATT AATTGGAGAAGCCGTAAAAA CACAGTTCAATTATTATAAG AAAGTTGATGGTGTTGTCCA ACAATTACCTGAAACTTACT 20200

20201 TTACTCAGAGTAGAAATTTA CAAGAATTTAAACCCAGGAG TCAAATGGAAATTGATTTCT TAGAATTAGCTATGGATGAA TTCATTGAACGGTATAAATT 20300

20301 AGAAGGCTATGCCTTCGAAC ATATCGTTTATGGAGATTTT AGTCATAGTCAGTTAGGTGG TTTACATCTACTGATTGGAC TAGCTAAACGTTTTAAGGAA 20400

20401 TCACCTTTTGAATTAGAAGA TTTTATTCCTATGGACAGTA CAGTTAAAAACTATTTCATA ACAGATGCGCAAACAGGTTC ATCTAAGTGTGTGTGTTCTG 20500

20501 TTATTGATTTATTACTTGAT GATTTTGTTGAAATAATAAA ATCCCAAGATTTATCTGTAG TTTCTAAGGTTGTCAAAGTG ACTATTGACTATACAGAAAT 20600

20601 TTCATTTATGCTTTGGTGTA AAGATGGCCATGTAGAAACA TTTTACCCAAAATTACAATC TAGTCAAGCGTGGCAACCGG GTGTTGCTATGCCTAATCTT 20700

20701 TACAAAATGCAAAGAATGCT ATTAGAAAAGTGTGACCTTC AAAATTATGGTGATAGTGCA ACATTACCTAAAGGCATAAT GATGAATGTCGCAAAATATA 20800

20801 CTCAACTGTGTCAATATTTA AACACATTAACATTAGCTGT ACCCTATAATATGAGAGTTA TACATTTTGGTGCTGGTTCT GATAAAGGAGTTGCACCAGG 20900

20901 TACAGCTGTTTTAAGACAGT GGTTGCCTACGGGTACGCTG CTTGTCGATTCAGATCTTAA TGACTTTGTCTCTGATGCAG ATTCAACTTTGATTGGTGAT 21000

21001 TGTGCAACTGTACATACAGC TAATAAATGGGATCTCATTA TTAGTGATATGTACGACCCT AAGACTAAAAATGTTACAAA AGAAAATGACTCTAAAGAGG 21100

21101 GTTTTTTCACTTACATTTGT GGGTTTATACAACAAAAGCT AGCTCTTGGAGGTTCCGTGG CTATAAAGATAACAGAACAT TCTTGGAATGCTGATCTTTA 21200

21201 TAAGCTCATGGGACACTTCG CATGGTGGACAGCCTTTGTT ACTAATGTGAATGCGTCATC ATCTGAAGCATTTTTAATTG GATGTAATTATCTTGGCAAA 21300

21301 CCACGCGAACAAATAGATGG TTATGTCATGCATGCAAATT ACATATTTTGGAGGAATACA AATCCAATTCAGTTGTCTTC CTATTCTTTATTTGACATGA 21400

21401 GTAAATTTCCCCTTAAATTA AGGGGTACTGCTGTTATGTC TTTAAAAGAAGGTCAAATCA ATGATATGATTTTATCTCTT CTTAGACTTGGTAGACTTAT 21500

21501 AATTAGAGAAAACAACAGAG TTGTTATTTCTAGTGATGTT CTTGTTAACAACTAAACGAA CAATGTTTGTTTTTCTTGTT TTATTGCCACTAGTCTCTAG 21600

21601 TCAGTGTGTTAATCTTACAA CCAGAACTCAATTACCCAAG TCGGACACTAATTCTTTCAC ACGTGGTGTTTATTACCCTG ACAAAGTTTTCAGATCCTCA 21700

21701 GTTTTACATTCAACTCAGGA CTTGTTCTTACCTTTCTTTT CCAATGTTACTTGGTTCCAT GCTATACATGTCTCTGGGAC CAATGGTACTAAGAGGTTTG 21800

21801 ATAACCCTGTCCTACCATTT AATGATGGTGTTTATTTTGC TTCCACTGAGAAGTCTAACA TAATAAGAGGCTGGATTTTT GGTACTACTTTAGATTCGAA 21900

21901 GACCCAGTCCCTACTTATTG TTAATAACGCTACTAATGTT GTTATTAAAGTCTGTGAATT TCAATTTTGTAATGATCCAT TTTTGGGTGTTTATTACCAC 22000

22001 AAAAACAACAAAAGTTGGAT GGAAAGTGAGTTCAGAGTTT ATTCTAGTGCGAATAATTGC ACTTTTGAATATGTCTCTCA GCCTTTTCTTATGGACCTTG 22100

22101 AAGGAAAACAGGGCAATTTC AAAAATCTTAGGGAATTTGT GTTTAAGAATATTGATGGTT ATTTTAAAATATATTCTAAG CACACGCCTATTAATTTAGT 22200

22201 GCGTGATCTCCCTCAGGGTT TTTCGGCTTTAGAACCATTG GTAGATTTGCCAATAGGTAT TAACATCACTAGGTTTCAAA CTTTACTTGCTTTACATAGA 22300

22301 AGTTATTTGACTCCTGGTGA TTCTTCTTCAGGTTGGACAG CTGGTGCTGCAGCTTATTAT GTGGGTTATCTTCAACCTAG GACTTTTCTATTAAAATATA 22400

22401 ATGAAAATGGAACCATTACA GATGCTGTAGACTGTGCACT TGACCCTCTCTCAGAAACAA AGTGTACGTTGAAATCCTTC ACTGTAGAAAAAGGAATCTA 22500

22501 TCAAACTTCTAACTTTAGAG TCCAACCAACAGAATCTATT GTTAGATTTCCTAATATTAC AAACTTGTGCCCTTTTGGTG AAGTTTTTAACGCCACCAGA 22600

22601 TTTGCATCTGTTTATGCTTG GAACAGGAAGAGAATCAGCA ACTGTGTTGCTGATTATTCT GTCCTATATAATTCCGCATC ATTTTCCACTTTTAAGTGTT 22700

22701 ATGGAGTGTCTCCTACTAAA TTAAATGATCTCTGCTTTAC TAATGTCTATGCAGATTCAT TTGTAATTAGAGGTGATGAA GTCAGACAAATCGCTCCAGG 22800

22801 GCAAACTGGAAAGATTGCTG ATTATAATTATAAATTACCA GATGATTTTACAGGCTGCGT TATAGCTTGGAATTCTAACA ATCTTGATTCTAAGGTTGGT 22900

22901 GGTAATTATAATTACCTGTA TAGATTGTTTAGGAAGTCTA ATCTCAAACCTTTTGAGAGA GATATTTCAACTGAAATCTA TCAGGCCGGTAGCACACCTT 23000

23001 GTAATGGTGTTGAAGGTTTT AATTGTTACTTTCCTTTACA ATCATATGGTTTCCAACCCA CTAATGGTGTTGGTTACCAA CCATACAGAGTAGTAGTACT 23100

23101 TTCTTTTGAACTTCTACATG CACCAGCAACTGTTTGTGGA CCTAAAAAGTCTACTAATTT GGTTAAAAACAAATGTGTCA ATTTCAACTTCAATGGTTTA 23200

23201 ACAGGCACAGGTGTTCTTAC TGAGTCTAACAAAAAGTTTC TGCCTTTCCAACAATTTGGC AGAGACATTGCTGACACTAT CGATGCTGTCCGTGATCCAC 23300

23301 AGACACTTGAGATTCTTGAC ATTACACCATGTTCTTTTGG TGGTGTCAGTGTTATAACAC CAGGAACAAATACTTCTAAC CAGGTTGCTGTTCTTTATCA 23400

23401 GGATGTTAACTGCACAGAAG TCCCTGTTGCTATTCATGCA GATCAACTTACTCCTACTTG GCGTGTTTATTCTACAGGTT CTAATGTTTTTCAAACACGT 23500

23501 GCAGGCTGTTTAATAGGGGC TGAACATGTCAACAACTCAT ATGAGTGTGACATACCCATT GGTGCAGGTATATGCGCTAG TTATCAGACCCAGACTAATT 23600

23601 CTCCTCGGCGGGCACGTAGT GTAGCTAGTCAATCCATCAT TGCCTACACTATGTCACTTG GTGCAGAAAATTCAGTTGCT TACTCTAATAACTCTATTGC 23700

23701 CATACCCACAAATTTTACTA TTAGTGTTACCACAGAAATT CTACCAGTGTCTATGACCAA GACATCAGTAGATTGTACAA TGTACATTTGTGGTGATTCA 23800

23801 ACTGAATGCAGCAATCTTTT GTTGCAATATGGCAGTTTTT GTACACAATTAAACCGTGCT TTAACTGGAATAGCTGTTGA ACAAGACAAAAACACCCAAG 23900

23901 AAGTTTTTGCACAAGTCAAA CAAATTTACAAAACACCACC AATTAAAGATTTTGGTGGTT TTAATTTTTCACAAATATTA CCAGATCCATCAAAACCAAG 24000

24001 CAAGAGGTCATTTATTGAAG ATCTACTTTTCAACAAAGTG ACACTTGCAGATGCTGGCTT CATCAAACAATATGGTGATT GCCTTGGTGATATTGCTGCT 24100

24101 AGAGACCTCATTTGTGCACA AAAGTTTAACGGCCTTACTG TTTTGCCACCTTTGCTCACA GATGAAATGATTGCTCAATA CACTTCTGCACTGTTAGCGG 24200

24201 GTACAATCACTTCTGGTTGG ACCTTTGGTGCAGGTGCTGC ATTACAAATACCATTTGCTA TGCAAATGGCTTATAGGTTT AATGGTATTGGAGTTACACA 24300

24301 GAATGTTCTCTATGAGAACC AAAAATTGATTGCCAACCAA TTTAATAGTGCTATTGGCAA AATTCAAGACTCACTTTCTT CCACAGCAAGTGCACTTGGA 24400

24401 AAACTTCAAGATGTGGTCAA CCAAAATGCACAAGCTTTAA ACACGCTTGTTAAACAACTT AGCTCCAATTTTGGTGCAAT TTCAAGTGTTTTAAATGATA 24500

24501 TCCTTTCACGTCTTGACAAA GTTGAGGCTGAAGTGCAAAT TGATAGGTTGATCACAGGCA GACTTCAAAGTTTGCAGACA TATGTGACTCAACAATTAAT 24600

24601 TAGAGCTGCAGAAATCAGAG CTTCTGCTAATCTTGCTGCT ACTAAAATGTCAGAGTGTGT ACTTGGACAATCAAAAAGAG TTGATTTTTGTGGAAAGGGC 24700

24701 TATCATCTTATGTCCTTCCC TCAGTCAGCACCTCATGGTG TAGTCTTCTTGCATGTGACT TATGTCCCTGCACAAGAAAA GAACTTCACAACTGCTCCTG 24800

24801 CCATTTGTCATGATGGAAAA GCACACTTTCCTCGTGAAGG TGTCTTTGTTTCAAATGGCA CACACTGGTTTGTAACACAA AGGAATTTTTATGAACCACA 24900

24901 AATCATTACTACAGACAACA CATTTGTGTCTGGTAACTGT GATGTTGTAATAGGAATTGT CAACAACACAGTTTATGATC CTTTGCAACCTGAATTAGAC 25000

25001 TCATTCAAGGAGGAGTTAGA TAAATATTTTAAGAATCATA CATCACCAGATGTTGATTTA GGTGACATCTCTGGCATTAA TGCTTCAGTTGTAAACATTC 25100

25101 AAAAAGAAATTGACCGCCTC AATGAGGTTGCCAAGAATTT AAATGAATCTCTCATCGATC TCCAAGAACTTGGAAAGTAT GAGCAGTATATAAAATGGCC 25200

25201 ATGGTACATTTGGCTAGGTT TTATAGCTGGCTTGATTGCC ATAGTAATGGTGACAATTAT GCTTTGCTGTATGACCAGTT GCTGTAGTTGTCTCAAGGGC 25300

25301 TGTTGTTCTTGTGGATCCTG CTGCAAATTTGATGAAGACG ACTCTGAGCCAGTGCTCAAA GGAGTCAAATTACATTACAC ATAAACGAACTTATGGATTT 25400

25401 GTTTATGAGAATCTTCACAA TTGGAACTGTAACTTTGAAG CAAGGTGAAATCAAGGATGC TACTCCTTCAGATTTTGTTC GCGCTACTGCAACGATACCG 25500

25501 ATACAAGCCTCACTCCCTTT CGGATGGCTTATTGTTGGCG TTGCACTTCTTGCTGTTTTT CAGAGCGCTTCCAAAATCAT AACCCTCAAAAAGAGATGGC 25600

25601 AACTAGCACTCTCCAAGGGT GTTCACTTTGTTTGCAACTT GCTGTTGTTGTTTGTAACAG TTTACTCACACCTTTTGCTC GTTGCTGCTGGCCTTGAAGC 25700

25701 CCCTTTTCTCTATCTTTATG CTTTAGTCTACTTCTTGCAG AGTATAAACTTTGTAAGAAT AATAATGAGGCTTTGGCTTT GCTGGAAATGCCGTTCCAAA 25800

25801 AACCCATTACTTTATGATGC CAACTATTTTCTTTGCTGGC ATACTAATTGTTACGACTAT TGTATACCTTACAATAGTGT AACTTCTTCAATTGTCATTA 25900

25901 CTTCAGGTGATGGCACAACA AGTCCTATTTCTGAACATGA CTACCAGATTGGTGGTTATA CTGAAAAATGGGAATCTGGA GTAAAAGACTGTGTTGTATT 26000

26001 ACACAGTTACTTCACTTCAG ACTATTACCAGCTGTACTCA ACTCAATTGAGTACAGACAC TGGTGTTGAACATGTTACCT TCTTCATCTACAATAAAATT 26100

26101 GTTGATGAGCCTGAAGAACA TGTCCAAATTCACACAATCG ACGGTTCATCCGGAGTTGTT AATCCAGTAATGGAACCAAT TTATGATGAACCGACGACGA 26200

26201 CTACTAGCGTGCCTTTGTAA GCACAAGCTGATGAGTACGA ACTTATGTACTCATTCGTTT CGGAAGAGACAGGTACGTTA ATAGTTAATAGCGTACTTCT 26300

26301 TTTTCTTGCTTTCGTGGTAT TCTTGCTAGTTACACTAGCC ATCCTTACTGCGCTTCGATT GTGTGCGTACTGCTGCAATA TTGTTAACGTGAGTCTTGTA 26400

26401 AAACCTTCTTTTTACGTTTA CTCTCGTGTTAAAAATCTGA ATTCTTCTAGAGTTCCTGAT CTTCTGGTCTAAACGAACTA AATATTATATTAGTTTTTCT 26500

26501 GTTTGGAACTTTAATTTTAG CCATGGCAGATTCCAACGGT ACTATTACCGTTGAAGAGCT TAAAAAGCTCCTTGAACAAT GGAACCTAGTAATAGGTTTC 26600

26601 CTATTCCTTACATGGATTTG TCTTCTACAATTTGCCTATG CCAACAGGAATAGGTTTTTG TATATAATTAAGTTAATTTT CCTCTGGCTGTTATGGCCAG 26700

26701 TAACTTTAGCTTGTTTTGTG CTTGCTGCTGTTTACAGAAT AAATTGGATCACCGGTGGAA TTGCTATCGCAATGGCTTGT CTTGTAGGCTTGATGTGGCT 26800

26801 CAGCTACTTCATTGCTTCTT TCAGACTGTTTGCGCGTACG CGTTCCATGTGGTCATTCAA TCCAGAAACTAACATTCTTC TCAACGTGCCACTCCATGGC 26900

26901 ACTATTCTGACCAGACCGCT TCTAGAAAGTGAACTCGTAA TCGGAGCTGTGATCCTTCGT GGACATCTTCGTATTGCTGG ACACCATCTAGGACGCTGTG 27000

27001 ACATCAAGGACCTGCCTAAA GAAATCACTGTTGCTACATC ACGAACGCTTTCTTATTACA AATTGGGAGCTTCGCAGCGT GTAGCAGGTGACTCAGGTTT 27100

27101 TGCTGCATACAGTCGCTACA GGATTGGCAACTATAAATTA AACACAGACCATTCCAGTAG CAGTGACAATATTGCTTTGC TTGTACAGTAAGTGACAACA 27200

27201 GATGTTTCATCTCGTTGACT TTCAGGTTACTATAGCAGAG ATATTACTAATTATTATGAG GACTTTTAAAGTTTCCATTT GGAATCTTGATTACATCATA 27300

27301 AACCTCATAATTAAAAATTT ATCTAAGTCACTAACTGAGA ATAAATATTCTCAATTAGAT GAAGAGCAACCAATGGAGAT TGATTAAACGAACATGAAAA 27400

27401 TTATTCTTTTCTTGGCACTG ATAACACTCGCTACTTGTGA GCTTTATCACTACCAAGAGT GTGTTAGAGGTACAACAGTA CTTTTAAAAGAACCTTGCTC 27500

27501 TTCTGGAACATACGAGGGCA ATTCACCATTTCATCCTCTA GCTGATAACAAATTTGCACT GACTTGCTTTAGCACTCAAT TTGCTTTTGCTTGTCCTGAC 27600

27601 GGCGTAAAACACGTCTATCA GTTACGTGCCAGATCAGTTT CACCTAAACTGTTCATCAGA CAAGAGGAAGTTCAAGAACT TTACTCTCCAATTTTTCTTA 27700

27701 TTGTTGCGGCAATAGTGTTT ATAACACTTTGCTTCACACT CAAAAGAAAGACAGAATGAT TGAACTTTCATTAATTGACT TCTATTTGTGCTTTTTAGCC 27800

27801 TTTCTGCTATTCCTTGTTTT AATTATGCTTATTATCTTTT GGTTCTCACTTGAACTGCAA GATCATAATGAAACTTGTCA CGCCTAAACGAACATGAAAT 27900

27901 TTCTTGTTTTCTTAGGAATC ATCACAACTGTAGCTGCATT TCACCAAGAATGTAGTTTAC AGTCATGTACTCAACATCAA CCATATGTAGTTGATGACCC 28000

28001 GTGTCCTATTCACTTCTATT CTAAATGGTATATTAGAGTA GGAGCTAGAAAATCAGCACC TTTAATTGAATTGTGCGTGG ATGAGGCTGGTTCTAAATCA 28100

28101 CCCATTCAGTACATCGATAT CGGTAATTATACAGTTTCCT GTTTACCTTTTACAATTAAT TGCCAGGAACCTAAATTGGG TAGTCTTGTAGTGCGTTGTT 28200

28201 CGTTCTATGAAGACTTTTTA GAGTATCATGACGTTCGTGC TTGTAGATCTGTTCTCTAAA CGAACAAACTAAAATGTCTG ATAATGGACCCCAAAATCAG 28300

28301 CGAAATGCACCCCGCATTAC GTTTGGTGGACCCTCAGATT CAACTGGCAGTAACCAGAAT GGAGAACGCAGTGGGGCGCG ATCAAAACAACGTCGGCCCC 28400

28401 AAGGTTTACCCAATAATACT GCGTCTTGGTTCACCGCTCT CACTCAACATGGCAAGGAAG ACCTTAAATTCCCTCGAGGA CAAGGCGTTCCAATTAACAC 28500

28501 CAATAGCAGTCCAGATGACC AAATTGGCTACTACCGAAGA GCTACCAGACGAATTCGTGG TGGTGACGGTAAAATGAAAG ATCTCAGTCCAAGATGGTAT 28600

28601 TTCTACTACCTAGGAACTGG GCCAGAAGCTGGACTTCCCT ATGGTGCTAACAAAGACGGC ATCATATGGGTTGCAACTGA GGGAGCCTTGAATACACCAA 28700

28701 AAGATCACATTGGCACCCGC AATCCTGCTAACAATGCTGC AATCGTGCTACAACTTCCTC AAGGAACAACATTGCCAAAA GGCTTCTACGCAGAAGGGAG 28800

28801 CAGAGGCGGCAGTCAAGCCT CTTCTCGTTCCTCATCACGT AGTCGCAACAGTTCAAGAAA TTCAACTCCAGGCAGCAGTA GGGGAACTTCTCCTGCTAGA 28900

28901 ATGGCTGGCAATGGCGGTGA TGCTGCTCTTGCTTTGCTGC TGCTTGACAGATTGAACCAG CTTGAGAGCAAAATGTCTGG TAAAGGCCAACAACAACAAG 29000

29001 GCCAAACTGTCACTAAGAAA TCTGCTGCTGAGGCTTCTAA GAAGCCTCGGCAAAAACGTA CTGCCACTAAAGCATACAAT GTAACACAAGCTTTCGGCAG 29100

29101 ACGTGGTCCAGAACAAACCC AAGGAAATTTTGGGGACCAG GAACTAATCAGACAAGGAAC TGATTACAAACATTGGTCGC AAATTGCACAATTTGCCCCC 29200

29201 AGCGCTTCAGCGTTCTTCGG AATGTCGCGCATTGGCATGG AAGTCACACCTTCGGGAACG TGGTTGACCTACACAGGTGC CATCAAATTGGATGACAAAG 29300

29301 ATCCAAATTTCAAAGATCAA GTCATTTTGCTGAATAAGCA TATTGACGCATACAAAACAT TCCCACCAACAGAGCCAAAA AAGGACAAAAAGAAGAAGGC 29400

29401 TGATGAAACTCAAGCCTTAC CGCAGAGACAGAAGAAACAG CAAACTGTGACTCTTCTTCC TGCTGCAGATTTGGATGATT TCTCCAAACAATTGCAACAA 29500

29501 TCCATGAGCAGTGCTGACTC AACTCAGGCCTAAACTCATG CGACCGCTTGGCCTCCGACT TGCTATATAAACGTTTTCGC TTTTCCGTTTACGATATATA 29600

29601 GTCTACTCTTGTGCAGAATG AATTCTCGTAACTACATAGC ACAAGTAGATGTAGTTAACT TTAATCTCACATAGCAATCT TTAATCAGTGTGTAACATTA 29700

29701 GGGAGGACTTGAAAGAGCCA CCAAGTCGGATCGTAGCCAT GTCGTAGTACGATCGAGTGT ACAGTGAACAATGCTAGGGA GAGCTGCCTATATGGAAGAG 29800

29801 CCCTAATGTGTAAAATTAAT TTTAGTAGTGCTATCCCCAT GTGATTTTAATAGCTTCTTA GGAGAATGACAAAAAAAAAA AAAAAAAAAA 29891

1.9. SRR12596175 Assembled Contig Spike Protein Sequence

1 MFLLTTKRTMFVFLVLLPLV SSQCVNLTTRTQLPPAYTNS FTRGVYYPDKVFRSSVLHST QDLFLPFFSNVTWFHAIHVS GTNGTKRFDNPVLPFNDGVY 100

101 FASTEKSNIIRGWIFGTTLD SKTQSLLIVNNATNVVIKVC EFQFCNDPFLGVYYHKNNKS WMESEFRVYSSANNCTFEYV SQPFLMDLEGKQGNFKNLRE 200

201 FVFKNIDGYFKIYSKHTPIN LVRDLPQGFSALEPLVDLPI GINITRFQTLLALHRSYLTP GDSSSGWTAGAAAYYVGYLQ PRTFLLKYNENGTITDAVDC 300

301 ALDPLSETKCTLKSFTVEKG IYQTSNFRVQPTESIVRFPN ITNLCPFGEVFNATRFASVY AWNRKRISNCVADYSVLYNS ASFSTFKCYGVSPTKLNDLC 400

401 FTNVYADSFVIRGDEVRQIA PGQTGKIADYNYKLPDDFTG CVIAWNSNNLDSKVGGNYNY LYRLFRKSNLKPFERDISTE IYQAGSTPCNGVEGFNCYFP 500

501 LQSYGFQPTNGVGYQPYRVV VLSFELLHAPATVCGPKKST NLVKNKCVNFNFNGLTGTGV LTESNKKFLPFQQFGRDIAD TTDAVRDPQTLEILDITPCS 600

601 FGGVSVITPGTNTSNQVAVL YQGVNCTEVPVAIHADQLTP TWRVYSTGSNVFQTRAGCLI GAEHVNNSYECDIPIGAGIC ASYQTQTNSPRRARSVASQS 700

701 IIAYTMSLGAENSVAYSNNS IAIPTNFTISVTTEILPVSM TKTSVDCTMYICGDSTECSN LLLQYGSFCTQLNRALTGIA VEQDKNTQEVFAQVKQIYKT 800

801 PPIKDFGGFNFSQILPDPSK PSKRSFIEDLLFNKVTLADA GFIKQYGDCLGDIAARDLIC AQKFNGLTVLPPLLTDEMIA QYTSALLAGTITSGWTFGAG 900

901 AALQIPFAMQMAYRFNGIGV TQNVLYENQKLIANQFNSAI GKIQDSLSSTASALGKLQDV VNQNAQALNTLVKQLSSNFG AISSVLNDILSRLDKVEAEV 1000

1001 QIDRLITGRLQSLQTYVTQQ LIRAAEIRASANLAATKMSE CVLGQSKRVDFCGKGYHLMS FPQSAPHGVVFLHVTYVPAQ EKNFTTAPAICHDGKAHFPR 1100

1101 EGVFVSNGTHWFVTQRNFYE PQIITTDNTFVSGNCDVVIG IVNNTVYDPLQPELDSFKEE LDKYFKNHTSPDVDLGDISG INASVVNIQKEIDRLNEVAK 1200

1201 NLNESLIDLQELGKYEQYIK WPWYIWLGFIAGLIAIVMVT IMLCCMTSCCSCLKGCCSCG SCCKFDEDDSEPVLKGVKLH YT 1282

1.10. SRR12596175 Discovered Strain 1 Possible Spike Protein Sequence

1 MFLLTTIRTMFVFLVLLPLV SSQCVNLKSRTQLPPAYTNS FTRGVYYPGKVFRSSVLHST QDLFLPFFSNVTWFHAIHVS GTIGTKRFDNPVLPFNDGVY 100

101 FASTEKSIIIRGWIFGTTLD SKTQSLLIVNNSTNVVIKVC EFQFCNDPFLGVYYHKNNKS WMESEFRVYSSANNCTFE-V SQPFLMDLEGKQGNFKNLRE 200

201 FVFKNIDGYFKIYSKHTPIN LVRDLPQGFSALEPLVDLPI GINITRFQIILSLHRSYLTP GDSSSGWTAGAAAYYVGYLQ PRSFLLKYNENGTITDAVDC 300

301 ALDPLSGTKCTLKSFTVEKG IYQTSNFRVQPTESIVRFPN IKYLCPFGEVFNATRFASVY AWNRKRISNCVADYSVLYNS VPFSTFKCYGVSPTKLNDLC 400

401 FTNVYADSFVIRGDEVRQIA PGQTGKIADYNYKLPDDFTG CVIAWNSNNLDSKVGGNYNY LYRLFRKSNLKPFERDISTE IYQAGSTPCNGVEGFNCYFL 500

501 LQLYGFQPTNGVGYQPYRVV VLSFELLHAPATVCASKKST NLVKNKCVNFNFNGLTGTGV LTESNKSFCLFNNLAETLLT LLMLSEVHRHLRFLTLHHVL 600

601 LVVSVL-RQEQILLTRLLFF IRVSTAQKSLLLFMQINLLL LGVFILQVLKFLKHVQAV– GLNMSTTHMSVTYPLVQVYA LVIRLSLILLGGHVV-LVNP 700

701 SLHSLCHLVQKIQLLTLITL LPYPQILLLVLPQKFYQCL- PRHQ-IVQCTFGMIQLNAAI YCCNMAVFVHN-TVL-LE-L LNKTKTPKKFVHKSNKFTKH 800

801 HQLKILVVLIFHK-FQIHQN QARGHLLKIYFSTK-HLQLL ASSNNMVIALVILLLEPSFV HKSLTALLFCHLCSQIR-LL NTLLHC-RVLSLLVGPLVQV 900

901 LHYKYHVLCKWLIGLMVLEL HRMFSMRTKN-LPTNLIVLL AKFKTHFLPQQANLENFKMW STKMHKL-TRLLNNLAPILV QFQVF-MISFHVLTKLRLKC 1000

1001 KLIG-SQADFNVCRHM-LNN -LELQKSELLLILLLLNCQS VYLDNQKELIFVERAIILCP SLSQHLMV-FSCM-LMSLHK KRTSQLLLPFVMME-HTFLV 1100

1101 KVSLFQMAHTGL-HKGIFMN HKSLLQTTHLCLVTVML–E VSTTQFMILCNLN-THSRRS -INILRIIHHQMLI-VTSLA LMLQL-TYKKKLTASMRLQ- 1200

1201 I-MNLSSISKNLESMSSI-M AMVHLARFYSWLDCHSNGDN YALLYDQLL-LSQGLLFLGI LLQI–RRL-ASAQRSQITL HI 1282

1.11. Difference of SRR11092062 Discovered Strain 1 Spike Protein Sequence and SARS-CoV-2 Spike Protein Sequence

The format of EMBOSS Needle is that each three lines followed an empty line, the first in the three lines is 50 amino acid bases of SARS-CoV-2 original strain spike protein, the second line is the matching status, and the third line is 50 amino acid bases of discovered strain 1. | means the two bases at the position in the first protein sequence and the second protein sequence are the same, other signs mean the two bases are different. The figure is located at 1 and 1.

1.12. SRR12596175 Discovered Strain 2 Possible Spike Protein Sequence

1 MFLLTTKRTMFVFLVLLPLD SSQCVNLTTRTQLPPAYTNS FTRGVYYPDKVFRSSVLHST QDLFLPFFSNVTWFHAIHVS GTNGTKRFDNPVLPFNDGVY 100

101 FASTEKSNIIRGWIFGTTLD SKTQSLLIVNNATNVVIKVC EFQFCNDPFLGVYYHKNNKS WMESEFRVYSSANNCTFEYV SQPFLMDLEGKQGNFKNLRE 200

201 FVFKNIDGYFKIYSKHTPIN LVRDLPQGFSALEPLVDLPM GINITRFQTLLALHRSYLTP GDSSSGWTAGAAAYYVGYLQ PRTFLLKYNENGTITDAVDC 300

301 ALDPLSETKCTLKSFTVEKG IYQTSNFRVQPTESIVRFPN ITNLCPFGEVFNATRFASVY AWNRKRISNCVADYSVLYNS ASFSTFKCYGVSPTKLNDLC 400

401 FTNVYADSFVIRGDEVRQIA PGQTGKIADYNYKLPDDFTG CVIAWNSNNLDSKVGGNYNY LYRLFRKSNLKPFERDISTE IYQAGSTPCNGVEGFNCYFP 500

501 LQSYGFQPTNGVGYQPYRVV VLSFELLHAPATVCGPKKST NLVKNKCVNFNFNGLTGTGV LTESNKKFLPFQQFGRDIAD TTDAVRDPQTLEILDITPCS 600

601 FGGVSVITPGTNTSNQVAVL YQGVNCTEVPVAIHADQLTP TWRVYSTGSNVFQTRAGCLI GAEHVNNSYECDIPIGAGIC ASYQTQTNSPRRARSVASQS 700

701 IIAYTMSLGAENSVAYSNNS IAIPTNFTISVTTEILPVSM TKTSVDCTMYICGDSTECSN LLLQYGSFCTQLNRALTGIA VEQDKNTQEVFAQVKQIYKT 800

801 PPIKDFGGFNFSQILPDPSK PSKRSFIEDLLFNKVTLADA GFIKQYGDCLGDIAARDLIC AQKFNGLTVLPPLLTDEMIA QYTSALLAGTITSGWTFGAG 900

901 AALQIPFAMQMAYRFNGIGV TQNVLYENQKLIANQFNSAI GKIQDSLSSTASALGKLQDV VNQNAQALNTLVKQLSSNFG AISSVLNDILSRLDKVEAEV 1000

1001 QIDRLITGRLQSLQTYVTQQ LIRAAEIRASANLAATKMSE CVLGQSKRVDFCGKGYHLMS FPQSAPHGVVFLHVTYVPAQ EKNFTTAPAICHDGKAHFPR 1100

1101 EGVFVSNGTHWFVTQRNFYE PQIITTDNTFVSGNCDVVIG IVNNTVYDPLQPELDSFKEE LDKYFKNHTSPDVDLGDISG INASVVNIQKEIDRLNEVAK 1200

1201 NLNESLIDLQELGKYEQYIK WPWYIWLGFIAGLIAIVMVT IMLCCMTSCCSCLKGCCSCG SCCKFDEDDSEPVLKGVKLH YT 1282

1.12.1. Difference of Discovered Strain 2 Spike Protein Sequence and SARS-CoV-2 Spike Protein Sequence

The format of EMBOSS Needle is that each three lines followed an empty line, the first in the three lines is 50 amino acid bases of SARS-CoV-2 original strain spike protein, the second line is the matching status, and the third line is 50 amino acid bases of discovered strain 2. | means the two bases at the position in the first protein sequence and the second protein sequence are the same, other signs mean the two bases are different.The figure is located at 2 and 2.

1.13. SRR12596175 Assembled Contig Nucleotide Sequence

1 GTGCTATCCCCATGTGATTT TTTTTTTGTCATTCTCCTAA GAAGCTATTAAAATCACATG GGGATAGCACTACTAAAATT AATTTTACACATTAGGGCTC 100

101 TTCCATATAGGCAGCTCTCC CTAGCATTGTTCACTGTACA CTCGATCGTACTCCGCGTGG CCTCGGTGAAAATGTGGTGG CTCTTTCAAGTCCTCCCTAA 200

201 TGTTACACACTGATTAAAGA TTGCTATGTGAGATTAAAGT TAACTACATCTACTTGTGCT ATGTAGTTACGAGAATTCAT TCTGCACAAGAGTAGACTAT 300

301 ATATCGTAAACGGAAAAGCG AAAACGTTTATATAGCCCAT CTGCCTTGTGTGGTCTGCAT GAGTTTAGGCCTGAGTTGAG TCAGCACTGCTCATGGATTG 400

401 TTGCAATTGTTTGGAGAAAT CATCCAAATCTGCAGCAGGA AGAAGAGTCACAGTTTGCTG TTTCTTCTGTCTCTGCGGTA AGGCTTGAGTTTCATCAGCC 500

501 TTCTTCTTTTTGTCCTTTTT AGGCTCTGTTGGTGGGAATG TTTTGTATGCGTCAATATGC TTATTCAGCAAAATGACTTG ATCTTTGAAATTTGGATCTT 600

601 TGTCATCCAATTTGATGGCA CCTGTGTAGGTCAACCACGT TCCCGAAGGTGTGACTTCCA TGCCAATGCGCGACATTCCG AAGAACGCTGAAGCGCTGGG 700

701 GGCAAATTGTGCAATTTGCG GCCAATGTTTGTAATCAGTT CCTTGTCTGATTAGTTCCTG GTCCCCAAAATTTCCTTGGG TTTGTTCTGGACCACGTCTG 800

801 CCGAAAGCTTGTGTTACATT GTATGCTTTAGTGGCAGTAC GTTTTTGCCGAGGCTTCTTA GAAGCCTCAGCAGCAGATTT CTTAGTGACAGTTTGGCCTT 900

901 GTTGTTGTTGGCCTTTACCA GACATTTTGCTCTCAAGCTG GTTCAATCTGTCAAGCAGCA GCAAAGCAAGAGCAGCATCA CCGCCATTGCCAGCCATTCT 1000

1001 AGCAGGAGAAGTTCCCCTAC TGCTGCCTGGAGTTGAATTT CTTGAACTGTTGCGACTACG TGATGAGGAACGAGAAGAGG CTTGACTGCCGCCTCTGCTC 1100

1101 CCTTCTGCGTAGAAGCCTTT TGGCAATGTTGTTCCTTGAG GAAGTTGTAGCACGATTGCA GCATTGTTAGCAGGATTGCG GGTGCCAATGTGATCTTTTG 1200

1201 GTGTATTCAAGGCTCCCTCA GTTGCAACCCATATGATGCC GTCTTTGTTAGCACCATAGG GAAGTCCAGCTTCTGGCCCA GTTCCTAGGTAGTAGAAATA 1300

1301 CCATCTTGGACTGAGATCTT TCATTTTACCGTCACCACCA CGAATTCGTCTGGTAGCTCT TCGGTAGTAGCCAATTTGGT CATCTGGACTGCTATTGGTG 1400

1401 TTAATTGGAACGCCTTGTCC TCGAGGGAATTTAAGGTCTT CCTTGCCATGTTGAGTGAGA GCGGTGAACCAAGACGCAGT ATTATTGGGTAAACCTTGGG 1500

1501 GCCGACGTTGTTTTGATCGC GCCCCACTGCGTTCTCCATT CTGGTTACTGCCAGTTGAAT CTGAGGGTCCACCAAACGTA ATGCGGGGTGCATTTCGCTG 1600

1601 ATTTTGGGGTCCATTATCAG ACATTTTAGTTTGTTCGTTT AGATGAAATCTAAAACAACA CGAACGTCATGATACTCTAA AAAGTCTTCATAGAACGAAC 1700

1701 AACGCACTACAAGACTACCC AATTTAGGTTCCTGGCAATT AATTGTAAAAGGTAAACAGG AAACTGTATAATTACCGATA TCGATGTACTGAATGGGTGA 1800

1801 TTTAGAACCAACCTCATCCA CGCACAATTCAATTAAAGGT GCTGATTTTCTAGCTCCTAC TCTAATATACCATTTAGAAT AGAAGTGAATAGGACACGGG 1900

1901 TCATCAACTACATATGGTTG ATGTTGAGTACATGACTGTA AACTACATTCTTGGTGAAAT GCAGCTACAGTTGTGATGAT TCCTAAGAAAACAAGAAATT 2000

2001 TCATGTTCGTTTAGGCGTGA CAAGTTTCATTATGATCTTG CAGTTCAAGTGAGAACCAAA AGATAATAAGCATAATTAAA ACAAGGAATAGCAGAAAGGC 2100

2101 TAAAAAGCACAAATAGAAGT CAATTAATGAAAGTTCAATC ATTCTGTCTTTCTTTTGAGT GTGAAGCAAAGTGTTATAAA CACTATTGCCGCAACAATAA 2200

2201 GAAAAATTGGAGAGTAAAGT TCTTGAACTTCCTCTTGTCT GATGAACAGTTTAGGTGAAA CTGATCTGGCACGTAACTGA TAGACGTGTTTTACGCCGTC 2300

2301 AGGACAAGCAAAAGCAAATT GAGTGCTAAAGCAAGTCAGT GCAAATTTGTTATCAGCTAG AGGATGAAATGGTGAATTGC CCTCGTATGTTCCAGAAGAG 2400

2401 CAAGGTTCTTTTAAAAGTAC TGTTGTACCTCTAACACACT CTTGGTAGTGATAAAGCTCA CAAGTAGCGAGTGTTATCAG TGCCAAGAAAAGAATAATTT 2500

2501 TCATGTTCGTTTAATCAATC TCCATTGGTTGCTCTTCATC TAATTGAGAATATTTATTCT CAGTTAGTGACTTAGATAAA TTTTTAATTATGAGGTTTAT 2600

2601 GATGTAATCAAGATTCCAAA TGGAAACTTTAAAAGTCCTC ATAATAATTAGTAATATCTC TGCTATAGTAACCTGAAAGT CAACGAGATGAAACATCTGT 2700

2701 TGTCACTTACTGTACAAGCA AAGCAATATTGTCACTGCTA CTGGAATGGTCTGTGTTTAA TTTATAGTTGCCAATCCTGT AGCGACTGTATGCAGCAAAA 2800

2801 CCTGAGTCACCTGCTACACG CTGCGAAGCTCCCAATTTGT AATAAGAAAGCGTTCGTGAT GTAGCAACAGTGATTTCTTT AGGCAGGTCCTTGATGTCAC 2900

2901 AGCGTCCTAGATGGTGTCCA GCAATACGAAGATGTCCACG AAGGATCACAGCTCCGATTA CGAGTTCACTTTCTAGAAGC GGTCTGGTCAGAATAGTGCC 3000

3001 ATGGAGTGGCACGTTGAGAA GAATGTTAGTTTCTGGATTG AATGACCACATGGAACGCGT ACGCGCAAACAGTCTGAAAG AAGCAATGAAGTAGCTGAGC 3100

3101 CACATCAAGCCTACAAGACA AGCCATTGCGATAGCAATTC CACCGGTGATCCAATTTATT CTGTAAACAGCAGCAAGCAC AAAACAAGCTAAAGTTACTG 3200

3201 GCCATAACAGCCAGAGGAAA ATTAACTTAATTATATACAA AAACCTATTCCTGTTGGCAT AGGCAAATTGTAGAAGACAA ATCCATGTAAGGAATAGGAA 3300

3301 ACCTATTACTAGGTTCCATT GTTCAAGGAGCTTTTTAAGC TCTTCAACGGTAATAGTACC GTTGGAATCTGCCATGGCTA AAATTAAAGTTCCAAACAGA 3400

3401 AAAACTAATATAATATTTAG TTCGTTTAGACCAGAAGATC AGGAACTCTAGAAGAATTCA GATTTTTAACACGAGAGTAA ACGTAAAAAGAAGGTTTTAC 3500

3501 AAGACTCACGTTAACAATAT TGCAGCAGTACGCACACAAT CGAAGCGCAGTAAGGATGGC TAGTGTAACTAGCAAGAATA CCACGAAAGCAAGAAAAAGA 3600

3601 AGTACGCTATTAACTATTAA CGTACCTGTCTCTTCCGAAA CGAATGAGTACATAAGTTCG TACTCATCAGCTTGTGCTTA CAAAGGCACGCTAGTAGTCG 3700

3701 TCGTCGGTTCATCATAAATT GGTTCCATTACTGGATTAAC AACTCCGGATGAACCGTCGA TTGTGTGAATTTGGACATGT TCTTCAGGCTCATCAACAAT 3800

3801 TTTATTGTAGATGAAGAAGG TAACATGTTCAACACCAGTG TCTGTACTCAATTGAGTTGA GTACAGCTGGTAATAGTCTG AAGTGAAGTAACTGTGTAAT 3900

3901 ACAACACAGTCTTTTACTCC AGATTCCCATTTTTCAGTAT AACCACCAATCTGGTAGTCA TGTTCAGAAATAGGACTTGT TGTGCCATCACCTGAAGTAA 4000

4001 TGACAATTGAAGAAGTTACA CTATTGTAAGGTATACAATA GTCGTAACAATTAGTATGCC AGCAAAGAAAATAGTTGGCA TCATAAAGTAATGGGTTTTT 4100

4101 GGAACGGCATTTCCAGCAAA GCCAAAGCCTCATTATTATT CTTACAAAGTTTATACTCTG CAAGAAGTAGACTAAAGCAT AAAGATAGAGAAAAGGGGCT 4200

4201 TCAAGGCCAGCAGCAACGAG CAAAAGGTGTGAGTAAACTG TTACAAACAACAACAGCAAG TTGCAAACAAAGTGAACACC CTTGGAGAGTGCTAGTTGCC 4300

4301 ATCTCTTTTTGAGGGTTATG ATTTTGGAAGCGCTATGAAA AACAGCAAGAAGTGCAACGC CAACAATAAGCCATCCGAAA GGGAGTGAGGCTTGTATCGG 4400

4401 TATCGTTGCAGTAGCGCGAA CAAAATCTGAAGGAGTAGCA TCCTTGATTTCACCTTGCTT CAAAGTTACAGTTCCAATTG TGAAGATTCTCATAAACAAA 4500

4501 TCCATAAGTTCGTTTATGTG TAATGTAATTTGACTCCTTT GAGCACTGGCTCAGAGTCGT CTTCATCAAATTTGCAGCAG GATCCACAAGAACAACAGCC 4600

4601 CTTGAGACAACTACAGCAAC TGGTCATACAGCAAAGCATA ATTGTCACCATTACTATGGC AATCAAGCCAGCTATAAAAC CTAGCCAAATGTACCATGGC 4700

4701 CATTTTATATACTGCTCATA CTTTCCAAGTTCTTGGAGAT CGATGAGAGATTCATTTAAA TTCTTGGCAACCTCATTGAG GCGGTCAATTTCTTTTTGAA 4800

4801 TGTTTACAACTGAAGCATTA ATGCCAGAGATGTCACCTAA ATCAACATCTGGTGATGTAT GATTCTTAAAATATTTATCT AACTCCTCCTTGAATGAGTC 4900

4901 TAATTCAGGTTGCAAAGGAT CATAAACTGTGTTGTTGACA ATTCCTATTACAACATCACA GTTACCAGACACAAATGTGT TGTCTGTAGTAATGATTTGT 5000

5001 GGTTCATAAAAATTCCTTTG TGTTACAAACCAGTGTGTGC CATTTGAAACAAAGACACCT TCACGAGGAAAGTGTGCTTT TCCATCATGACAAATGGCAG 5100

5101 GAGCAGTTGTGAAGTTCTTT TCTTGTGCAGGGACATAAGT CACATGCAAGAAGACTACAC CATGAGGTGCTGACTGAGGG AAGGACATAAGATGATAGCC 5200

5201 CTTTCCACAAAAATCAACTC TTTTTGATTGTCCAAGTACA CACTCTGACATTTTAGTAGC AGCAAGATTAGCAGAAGCTC TGATTTCTGCAGCTCTAATT 5300

5301 AATTGTTGAGTCACATATGT CTGCAAACTTTGAAGTCTGC CTGTGATCAACCTATCAATT TGCACTTCAGCCTCAACTTT GTCAAGACGTGAAAGGATAT 5400

5401 CATTTAAAACACTTGAAATT GCACCAAAATTGGAGCTAAG TTGTTTAACAAGCGTGTTTA AAGCTTGTGCATTTTGGTTG ACCACATCTTGAAGTTTTCC 5500

5501 AAGTGCACTTGCTGTGGAAG AAAGTGAGTCTTGAATTTTG CCAATAGCACTATTAAATTG GTTGGCAATCAATTTTTGGT TCTCATAGAGAACATTCTGT 5600

5601 GTAACTCCAATACCATTAAA CCTATAAGCCATTTGCATAG CAAATGGTATTTGTAATGCA GCACCTGCACCAAAGGTCCA ACCAGAAGTGATTGTACCCG 5700

5701 CTAACAGTGCAGAAGTGTAT TGAGCAATCATTTCATCTGT GAGCAAAGGTGGCAAAACAG TAAGGCCGTTAAACTTTTGT GCACAAATGAGGTCTCTAGC 5800

5801 AGCAATATCACCAAGGCAAT CACCATATTGTTTGATGAAG CCAGCATCTGCAAGTGTCAC TTTGTTGAAAAGTAGATCTT CAATAAATGACCTCTTGCTT 5900

5901 GGTTTTGATGGATCTGGTAA TATTTGTGAAAAATTAAAAC CACCAAAATCTTTAATTGGT GGTGTTTTGTAAATTTGTTT GACTTGTGCAAAAACTTCTT 6000

6001 GGGTGTTTTTGTCTTGTTCA ACAGCTATTCCAGTTAAAGC ACGGTTTAATTGTGTACAAA AACTGCCATATTGCAACAAA AGATTGCTGCATTCAGTTGA 6100

6101 ATCACCACAAATGTACATTG TACAATCTACTGATGTCTTG GTCATAGACACTGGTAGAAT TTCTGTGGTAACACTAATAG TAAAATTTGTGGGTATGGCA 6200

6201 ATAGAGTTATTAGAGTAAGC AACTGAATTTTCTGCACCAA GTGACATAGTGTAGGCAATG ATGGATTGACTAGCTACACT ACGTGCCCGCCGAGGAGAAT 6300

6301 TAGTCTGAGTCTGATAACTA GCGCATATACCTGCACCAAT GGGTATGTCACACTCATATG AGTTGTTGACATGTTCAGCC CCTATTAAACAGCCTGCACG 6400

6401 TGTTTGAAAAACATTAGAAC CTGTAGAATAAACACGCCAA GTAGGAGTAAGTTGATCTGC ATGAATAGCAACAGGGACTT CTGTGCAGTTAACACCCTGA 6500

6501 TAAAGAACAGCAACCTGGTT AGAAGTATTTGTTCCTGGTG TTATAACACTGACACCACCA AAAGAACATGGTGTAATGTC AAGAATCTCAAGTGTCTGTG 6600

6601 GATCACGGACAGCATCAGTA GTGTCAGCAATGTCTCTGCC AAATTGTTGGAAAGGCAGAA ACTTTTTGTTAGACTCAGTA AGAACACCTGTGCCTGTTAA 6700

6701 ACCATTGAAGTTGAAATTGA CACATTTGTTTTTAACCAAA TTAGTAGACTTTTTAGGTCC ACAAACAGTTGCTGGTGCAT GTAGAAGTTCAAAAGAAAGT 6800

6801 ACTACTACTCTGTATGGTTG GTAACCAACACCATTAGTGG GTTGGAAACCATATGATTGT AAAGGAAAGTAACAATTAAA ACCTTCAACACCATTACAAG 6900

6901 GTGTGCTACCGGCCTGATAG ATTTCAGTTGAAATATCTCT CTCAAAAGGTTTGAGATTAG ACTTCCTAAACAATCTATAC AGGTAATTATAATTACCACC 7000

7001 AACCTTAGAATCAAGATTGT TAGAATTCCAAGCTATAACG CAGCCTGTAAAATCATCTGG TAATTTATAATTATAATCAG CAATCTTTCCAGTTTGCCCT 7100

7101 GGAGCGATTTGTCTGACTTC ATCACCTCTAATTACAAATG AATCTGCATAGACATTAGTA AAGCAGAGATCATTTAATTT AGTAGGAGACACTCCATAAC 7200

7201 ACTTAAAAGTGGAAAATGAT GCGGAATTATATAGGACAGA ATAATCAGCAACACAGTTGC TGATTCTCTTCCTGTTCCAA GCATAAACAGATGCAAATCT 7300

7301 GGTGGCGTTAAAAACTTCAC CAAAAGGGCACAAGTTTGTA ATATTAGGAAATCTAACAAT AGATTCTGTTGGTTGGACTC TAAAGTTAGAAGTTTGATAG 7400

7401 ATTCCTTTTTCTACAGTGAA GGATTTCAACGTACACTTTG TTTCTGAGAGAGGGTCAAGT GCACAGTCTACAGCATCTGT AATGGTTCCATTTTCATTAT 7500

7501 ATTTTAATAGAAAAGTCCTA GGTTGAAGATAACCCACATA ATAAGCTGCAGCACCAGCTG TCCAACCTGAAGAAGAATCA CCAGGAGTCAAATAACTTCT 7600

7601 ATGTAAAGCAAGTAAAGTTT GAAACCTAGTGATGTTAATA CCTATTGGCAAATCTACCAA TGGTTCTAAAGCCGAAAAAC CCTGAGGGAGATCACGCACT 7700

7701 AAATTAATAGGCGTGTGCTT AGAATATATTTTAAAATAAC CATCAATATTCTTAAACACA AATTCCCTAAGATTTTTGAA ATTACCCTGTTTTCCTTCAA 7800

7801 GGTCCATAAGAAAAGGCTGA GAGACATATTCAAAAGTGCA ATTATTCGCACTAGAATAAA CTCTGAACTCACTTTCCATC CAACTTTTGTTGTTTTTGTG 7900

7901 GTAATAAACACCCAAAAATG GATCATTACAAAATTGAAAT TCACAGACTTTAATAACAAC ATTAGTAGCGTTATTAACAA TAAGTAGGGACTGGGTCTTC 8000

8001 GAATCTAAAGTAGTACCAAA AATCCAGCCTCTTATTATGT TAGACTTCTCAGTGGAAGCA AAATAAACACCATCATTAAA TGGTAGGACAGGGTTATCAA 8100

8101 ACCTCTTAGTACCATTGGTC CCAGAGACATGTATAGCATG GAACCAAGTAACATTGGAAA AGAAAGGTAAGAACAAGTCC TGAGTTGAATGTAAAACTGA 8200

8201 GGATCTGAAAACTTTGTCAG GGTAATAAACACCACGTGTG AAAGAATTAGTGTATGCAGG GGGTAATTGAGTTCTGGTTG TAAGATTAACACACTGACTA 8300

8301 GAGACTAGTGGCAATAAAAC AAGAAAAACAAACATTGTTC GTTTAGTTGTTAACAAGAAC ATCACTAGAAATAACAACTC TGTTGTTTTCTCTAATTATA 8400

8401 AGTCTACCTTTACTAAGAAG AGATAAAATCATATCATTGA TTTGACCTTCTTTTAAAGAC ATAACAGCAGTACCCCTTAA TTTAAGGGGAAATTTACTCA 8500

8501 TGTCAAATAAAGAATAGGAA GACAACTGAATTGGATTTGT ATTCCTCCAAAATATGTAAT TTGCATGCATGACATAACCA TCTATTTGTTCGCGTGGTTT 8600

8601 GCCAAGATAATTACATCCAA TTAAAAATGCTTCAGATGAT GACGCATTCACATTAGTAAC AAAGGCTGTCCACCATGCGA AGTGTCCCATGAGCTTATAA 8700

8701 AGATCAGCATTCCAAGAATG TTCTGTTATCTTTATAGCCA CGGAACCTCCAAGAGCTAGC TTTTGTTGTATAAACCCACA AATGTAAGTGAAAAAACCCT 8800

8801 CTTTAGAGTCATTTTCTTTT GTAACATTTTTAGTCTTAGG GTCGTACATATCACTAATAA TGAGATCCCATTTATTAGCT GTATGTACAGTTGCACAATC 8900

8901 ACCAATCAAAGTTGAATCTG CATCAGAGACAAAGTCATTA AGATCTGAATCGACAAGCAG CGTACCCGTAGGCAACCACT GTCTTAAAACAGCTGTACCT 9000

9001 GGTGCAACTCCTTTATCAGA ACCAGCACCAAAATGTATAA CTCTCATATTATAGGGTACA GCTAATGTTAATGTGTTTAA ATATTGACACAGTTGAGTAT 9100

9101 ATTTTGCGACATTCATCATT ATGCCTTTAGGTAATGTTGC ACTATCACCATAATTTTGAA GGTCACACTTTTCTAATAGC ATTCTTTGCATTTTGTAAAG 9200

9201 ATTAGGCATAGCAACACCCG GTTGCCACGCTTGACTAGAT TGTAATTTTGGGTAAAATGT TTCTACATGGCCATCTTTAC ACCAAAGCATAAATGAAATT 9300

9301 TCTGTATAGTCAATAGTCAC TTTGACAACCTTAGAAACTA CAGATAAATCTTGGGATTTT ATTATTTCAACAAAATCATC AAGTAATAAATCAATAACAG 9400

9401 AACACACACACTTAGATGAA CCTGTTTGCGCATCTGTTAT GAAATAGTTTTTAACTGTAC TGTCCATAGGAATAAAATCT TCTAATTCAAAAGGTGATTC 9500

9501 CTTAAAACGTTTAGCTAGTC CAATCAGTAGATGTAAACCA CCTAACTGACTATGACTAAA ATCTCCATAAACGATATGTT CGAAGGCATAGCCTTCTAAT 9600

9601 TTATACCGTTCAATGAATTC ATCCATAGCTAATTCTAAGA AATCAATTTCCATTTGACTC CTGGGTTTAAATTCTTGTAA ATTTCTACTCTGAGTAAAGT 9700

9701 AAGTTTCAGGTAATTGTTGG ACAACACCATCAACTTTCTT ATAATAATTGAACTGTGTTT TTACGGCTTCTCCAATTAAT GTGACTCCATTAAGACTAGC 9800

9801 TTGTTTGGGACCTACAGATG GTTGTAAACCTTTAACACTA CCTTCTGTAATAAGAACACC ATTACGGGCATTTCTAAATA AGTCTACTTGACCATCAACT 9900

9901 CTACCATCAAAAAAGACAGT GAGTGGTGCACAAATCGTTT CAGTTGGTTTCTTGGCTATG TCAGTCATAGAACAAACACC AATAGTAGATATATGTGCTG 10000

10001 GAGCATCTCTTTTGTAGTCC CAGATCACAGTATTAGCAGC AATGTCCACACCCAAATTAT TGAGTATTTTCACCTCTGGT ACTGGTTTAATGTTGCGCTT 10100

10101 AGCCCAAAGCTCAAATGCTA CATTAACAGGTAATGTTGTT TTATTTTCAAACAATTCTAC ATCAACACCATCAACTTTTG TGTAAACAGTGTTATTAATG 10200

10201 ATAGAAACTGGTACTTCACC CTGTTGTCCATCAAAGTGTC CCTTATTTACAACATTAAAA GCCACATTTTCTAAACTCTG AAGTCTTGTAAAAGTGTTCC 10300

10301 AGAGGTTATAAGTATCAAAT TGTTTGTAAACCCACAAGCT AAAGCCAGCTGAGATCATCA TGTTATAAGCATCGAGATAC AATCTGTACTCATTAGCATG 10400

10401 ATGTCTACAGACAGCACCAC CTAAATTGCAACGTGTTATA CACGTAGCAGACTTTAGTGG TACATAATCTATATCTGACA CTACTTGTTTTCCATGAGAC 10500

10501 TCACATGGACTGTCAGAGTA ATAGAAAAATGGTAATTGTT TTAAATTAACAAAAGCACTT TTATCAAAAGCTGGTGTGTG GAATGCATGTTTATTTACAT 10600

10601 ACAAACTGCCACCATCACAA CCAGGCAAGTTAAGGTTAGA TAGCACTCTAGTGTCAAATC TACAAACAATGGAATTAGCA GGATATCTATCGACATTGCA 10700

10701 ATTCCAAAATAGGCATACAC CATCTGTGAATTTGTCAGAA TGTGTGGCATAAGAATAGAA TAATTCTTCTATTTTATAAG CTTTGTCACTACAAGGCTGT 10800

10801 GCATCATAGAACTTCCATTC TACATCAGCTTGAGGTACAC ACTTAATAGCTTTAGGGTTA CCAATGTCGTGAAGAACTGG GAATTTGTCTGCTAATAATG 10900

10901 CAGCTTTAACAACCATGTGT TGAACCTTTCTACAAGCCGC ATTAATCTTCAGTTCATCAC CAATTATAGGATATTCAATA GTCCAGTCAACACGCTTAAC 11000

11001 AAAGCACTCGTGGACAGCTA GACACCTAGTCATGATTGCA TCACAACTAGCTACATGTGC ATTACCATGGACTTGACAAT ACAGATCATGGTTGCTTTGT 11100

11101 AGGTTACCTGTAAAACCCCA TTGTTGAACATCAATCATAA ACGGATTATAGACGTAATCA AATCCAATAGAATGATGCCA ACAGGCATAAGTGTCTGAAG 11200

11201 CAGTGGAAAAGCATGTGGCA CGTCTATCACATAGACAACA GGTGCGCTCAGGTCCTATTT TCACAAAATACTTCATAGAT GTCAACTCAAAGCCATGTGC 11300

11301 CCATAAGACAAATACGACTC TGTCAGAGAGATTTTTAAGT GTGTCACTTAACATTTGTAC AATCTTTATACGCACTACAT TCCAAGGAAGTCCTTTGTAC 11400

11401 ATAAGTGGTATGAGGTGTTT AAATTGATCTCCAGGCGGTG GTTTAGCACTAACTCTGGAA AAATCTGTATTATTAGGTGT ATCAACATAACCTGTAGGTA 11500

11501 CAGCAACTAGGTTAACACCT GTAGAAAAACCTAGCTGTAA AGGTAAATTGGTACCAACAG CTTCTCTAGTAGCATGACAC CCCTCGACATCGAAGCCAAT 11600

11601 CCATGCACGTACATGTCTTA TAGCTTCTTCGCGGGTGATA AACATGTTAGGGTAACCATT AACTTGATAATTCATTTTAA AACCCATCATAGAGATGAGT 11700

11701 CTTCTATAGGTCATGTCCTT AGGTATGCCAGGTATGTCAA CACATAAACCTTCAGTTTTG AATTTAGTGTCAACACTGAG GTGTGTAGGTGCCTGTGTAG 11800

11801 GATGTAACCCAGTGATTACC TTACTACAATCTTTAAAGAG TCCTGTTACATTTTCAGCTT GTAAAGTTGCCACATTCCTA CGTGGAATTTCAAGACTTGT 11900

11901 AAATTGCAACTTGTCATAAA GGTCTCTATCAGACATTATG CAAAGTATGCCTACTTTTGC TCTGGTAATAGCAACATTAA ATCTGTTTACATTACAAGAG 12000

12001 TGAGCTGTTTCAGTGGTTTG AGTGAATATGACATAGTCAT ATTCTGAGCCCTGTGATGAA TCAACAGTTTGAGTTGGTAG TCCCAAAATCTTTGAGGCTA 12100

12101 CAGCATTCTGTGAATTATAA GGTGAAATAAAGACAGCTTT TCTCCAAGCAGGGTTACGTG TAAGGAATTCTCTTACCACG CCTATTTGTGGCCTGTTAAT 12200

12201 TGCAGATGAAACATCATGCG TGATAACACCCTTATAAAAC ATTTTAAAGCATTGAGCTGA TTTGTCTTTATGTGCTTTAA GCTTATTATCATAAACCAAA 12300

12301 GCACTCACAGTGTCAACAAT TTCAGCAGGACAACGCCGAC AAGTTCCGAGGAACATGTCT GGACCTATAGTTTTCATAAG TCTACACACTGAATTGAAAT 12400

12401 ATTCTGGTTCTAGTGTGCCC TTAGTTAGCAATGTGCGTGG TGCAGGTAATTGAGCAGGGT CGCCAATGTACACATAGTGC TTAGCACGTAATCTGGCATT 12500

12501 GACAACACTCAAATCATAAT TTGTGGCCATTGAAATTTCA TCAAAGACAACTATATCTGC TGTCGTCTCAGGCAATGCAT TTACAGTACAAAAGACATAC 12600

12601 TGTTCTAATGTTGAATTCAC TTTGAATTTATCAAAACACT CTACACGAGCACGTGCAGGT ATAATTCTACTACATTTATC TATAGGCAAATATTTTAATG 12700

12701 CCTTCTCACATAGTGCATCA ACAGCGGCATGAGAGCAAGC TGTATACACTATGCGAGCAG AAGGGTAGTAGAGAGCTAGG CCAATAGCAAAATGACTCTT 12800

12801 ACCAGTACCAGGTGGTCCCT GGAGTGTAGAATACTTTTGC ATACCAACCTTTTGATAATT TGCAACATTGCTAGAAAACT CATCTGAGATATTGAGTGTT 12900

12901 GGGTATAAGCCAGTAATTCT AACATAGTGCTCTTGTGGCA CTAGTGTAGGTGCACTTAAT GGCATTACTGTATGTGATGT CAGCACAAAATAATCACCAA 13000

13001 CATTTAATTTGTAAGTTGTT GTACCTCGGTAAACAACAGC ATCACCATAGTCACCTTTTT CAAAGGTGTACTCTCCTATT TGTACTTTACTGTTTTTAGT 13100

13101 TACACGATAACCAGTAAAGA CATAATTTCGGTTAAGTGGT GGTCTAGGTTTACCAACTTC CCATGAAAGATGTAATTCTC TGTCAGACAGCACTTCACGT 13200

13201 ACAGTAGCAATACCATAAGA CAGTTTAAATGTCTCCTCAG TAGCTTTGAGCGTTTCTGCT GCAAAAAGCTTGAGTCTTTC AGTACAGGTGTTAGCTAAAA 13300

13301 TGTAATCACCAGCATTTGTC CAGTCACATGTTGCAATTGC ATTAAAGTCAGTAACATTAT CGCTACCAACACATGTATTT TTATATAAACCAAAAACTTG 13400

13401 TCCATTAGCACACAATGGAA AACTAATGGGTGGTTTATGT GATTTACAATAATAGCTCAT ACCTCCTAAGTAAAGTTGAG TCACATCTGTGACATCACAA 13500

13501 CCTGGAGCATTGCAAACATA CGGATTAACAGACAAGACTA ATTTATGTGATGTTGATATG ACATGGTCGTAACAGCATTT ACAACATAAGAATGGTCTAC 13600

13601 GTATGCAAGCACCACATCTT AATGAAGTCTGTGAATTGCA AAGAACACAAGCCCCAACAG CCTGTAAGACTGTATGCGGT GTGTACATAGCCTCATAAAA 13700

13701 CTCAGGTTCCCAATACCTTG AAGTGTTATCATTAGTAAGC ATAACAGAATACATGTCTAA CATGTGTCCTGTTAACTCAT CATGTAGCTTTCTTATGTAT 13800

13801 TGTAAGTACAAATGAAAGAC ATCAGCATACTCCTGATTAG GATGTTTAGTAAGTGGGTAA GCATCTATAGCTAAAGACAC GAACCGTTCAATCATAAGTG 13900

13901 TACCATCTGTTTTTACGATA TCATCTACAAAACAGCCGGC CCCTAGGATTCTTGATGGAT CTGGGTAAGGAAGGTACACA TAATCATCACCCTGTTTAAC 14000

14001 TAGCATTGTATGTTGAGAGC AAAATTCATGAGGTCCTTTA GTAAGGTCAGTCTCAGTCCA ACATTTTGCTTCAGACATAA AAACATTGTTTTGATAATAA 14100

14101 AGAACTGACTTAAAGTTCTT TATGCTAGCCACTAGACCTT GAGATGCATAAGTGCTATTG AAACACACAACAGCATCGTC AGAGAGTATCATCATTGAGA 14200

14201 AATGTTTACGCAAATATGCG TAAAACTCATTCACAAAGTC TGTGTCAACATCTCTATTTC TATAGAGACACTCATAAAGT CTGTGTTGTAAATTGCGGAC 14300

14301 ATACTTATCGGCAATTTTGT TACCATCAGTAGATAAAAGT GCATTAACATTGGCCGTGAC AGCTTGACAAATGTTAAAAA CACTATTAGCATAAGCAGTT 14400

14401 GTGGCATCTCCTGATGAGGT TCCACCTGGTTTAACATATA GTGAACCGCCACACATGACC ATTTCACTCAATACTTGAGC ACACTCATTAGCTAATCTAT 14500

14501 AGAAACGGTGTGACAAGCTA CAACACGTTGTATGTTTGCG AGCAAGAACAAGTGAGGCCA TAATTCTAAGCATGTTAGGC ATGGCTCTATCACATTTAGG 14600

14601 ATAATCCCAACCCATAAGGT GAGGGTTTTCTACATCACTA TAAACAGTTTTTAACATGTT GTGCCAACCACCATAGAATT TGCTTGTTCCAATTACTACA 14700

14701 GTAGCTCCTCTAGTGGCGGC TATTGATTTCAATAATTTTT GATGAAACTGTCTATTGGTC ATAGTACTACAGATAGAGAC ACCAGCTACGGTGCGAGCTC 14800

14801 TATTCTTTGCACTAATGGCA TACTTAAGATTCATTTGAGT TATAGTAGGGATGACATTAC GTTTTGTATATGCGAAAAGT GCATCTTGATCCTCATAACT 14900

14901 CATTGAATCATAATAAAGTC TAGCCTTACCCCATTTATTA AATGGAAAACCAGCTGATTT GTCTAGGTTGTTGACGATGA CTTGGTTAGCATTAATACAG 15000

15001 CCACCATCGTAACAATCAAA GTACTTATCAACAACTTCAA CTACAAATAGTAGTTGTCTG ATATCACACATTGTTGGTAG ATTATAACGATAGTAGTCAT 15100

15101 AATCGCTGATAGCAGCATTA CCATCCTGAGCAAAGAAGAA GTGTTTTAATTCAACAGAAC TTCCTTCCTTAAAGAAACCC TTAGACACAGCAAAGTCATA 15200

15201 GAAGTCTTTGTTAAAATTAC CGGGTTTGACAGTTTGAAAA GCAACATTGTTAGTAAGTGC AGCTACTGAAAAGCACGTAG TGCGTTTATCTAGTAATAGA 15300

15301 TTACCAGAAGCAGCGTGCAT AGCAGGGTCAGCAGCATACA CAAGTAATTCCTTAAAACTA AGTCTAGAGCTATGTAAGTT TACATCCTGATTATGTACAA 15400

15401 CACCTAGCTCTCTGAAGTGG TATCCAGTTGAAACTACAAA TGGAACACCATCAACAAATA TTTTTCTCACTAGTGGTCCA AAACTTGTAAGTGGGAACAC 15500

15501 TGTAGAGAATAAAACATTAA AGTTTGCACAATGCAGAATG CATCTGTCATCCAAACAGTT AACACAATTTGGGTGGTATG TCTGATCCCAATATTTAAAA 15600

15601 TAACGGTCAAAGAGTTTTAA CCTCTCTTCCGTGAAGTCAT ATTTTAACAAATCCCACTTA ATGTAAGGCTTTGTTAAGTC AGTGTCAACATGTGACTCTG 15700

15701 CAGTTAAAGCCCTGGTCAAG GTTAATATAGGCATTAACAA TGAATAATAAGAATCTACAA CAGGAACTCCACTACCTGGC GTGGTTTGTATGAAATCACC 15800

15801 GAAATCATACCAGTTACCAT TGAGATCTTGATTATCTAAT GTCAGTACACCAACAATACC AGCATTTCGCATGGCATCAC AGAATTGTACTGTTTTTAAC 15900

15901 AAAGCTTGGCGTACACGTTC ACCTAAGTTGGCGTATACGC GTAATATATCTGGGTTTTCT ACAAAATCATACCAGTCCTT TTTATTGAAATAATCATCAT 16000

16001 CACAACAATTGTATGTGACA AGTATTTCTTTTAATGTGTC ACAATTACCTTCATCAAAAT GCCTTAAAGCATAGACGAGG TCTGCCATTGTGTATTTAGT 16100

16101 AAGACGTTGACGTGATATAT GTGGTACCATGTCACCGTCT ATTCTAAACTTAAAGAAGTC ATGTTTAGCAACAGCTGGAC AATCCTTAAGTAAATTATAA 16200

16201 ATTGTTTCTTCATGTTGGTA GTTAGAGAAAGTGTGTCTCT TAACTACAAAGTAAGAATCA ATTAAATTGTCATCTTCGTC CTTTTCTTGGAAGCGACAAC 16300

16301 AATTAGTTTTTAGGAATTTA GCAAAACCAGCTACTTTATC ATTGTAGATGTCAAAAGCCC TGTATACGACATCAGTACTA GTGCCTGTGCCGCACGGTGT 16400

16401 AAGACGGGCTGCACTTACAC CGCAAACCCGTTTAAAAACG ATTGTGCATCAGCTGACTGA AGCATGGGTTCGCGGAGTTG ATCACAACTACAGCCATAAC 16500

16501 CTTTCCACATACCGCAGACG GTACAGACTGTGTTTTTAAG TGTAAAACCCACAGGGTCAT TAGCACAAGTTGTAGGTATT TGTACATACTTACCTTTTAA 16600

16601 GTCACAAAATCCTTTAGGAT TTGGATGATCTATGTGGCAA CGGCAGTACAGACAACACGA TGCACCACCAAAGGATTCTT GATCCATATTGGCTTCCGGT 16700

16701 GTAACTGTTATTGCCTGACC AGTACCAGTGTGTGTACACA ACATCTTAACACAATTAGTG ATTGGTTGTCCCCCACTAGC TAGATAATCTTTGTAAGCTT 16800

16801 TAGCAGCATCTACAGCAAAA GCACAGAAAGATAATACAGT TGAATTGGCAGGCACTTCTG TTGCATTACCAGCTTGTAGA CGTACTGTGGCAGCTAAACT 16900

16901 ACCAAGTACCATACCTCTAT TTAGGTTGTTTAATCCTTTA ATAAAGTATAAATACTTCAC TTTAGGACCTTTAGGTGTGT CTGTAACAAACCTACAAGGT 17000

17001 GGTTCCAGTTCTGTATAGAT AGTACCAGTTCCATCACTCT TAGGGAATCTAGCCCATTTC AAATCCTGTAAATCGGATAA CAGTGCAAGTACAAACCTAC 17100

17101 CTCCCTTTGTTGTGTTGTAG TAAGCTAACGCATTGTCATC AGTGCAAGCAGTTTGTGTAG TACCGGCAGCACAAGACATC TGTCGTAGTGCAACAGGACT 17200

17201 AAGCTCATTATTCTGTAATT TGACAGCAGAATTGGCCCTT AAAGCTGTTACAATAAGAGG CCATGCTAAATTAGGTGAAT TGTCCATACTAATTTCACTA 17300

17301 AGTTGAACAATTTTACTATC TGCATCTACAACCTGTTGGA TTTCCCACAATGCTGATGCA TAAGTAAATGTTGTACCATC ACACGTATTTTTATATGTGT 17400

17401 TATAGTCTGGTATGACAACC ATTAGTTTGGCTGCTGTTGT AAGAGGTATTATGTTCAAGG GAACACAACCATCTCTTGCA TTGTTGATAATGTTGTTGAG 17500

17501 TGCATCATTATCCAACTTTC TAAGCATAGTGAAAAGCATT GTCTGCATAGCACTAGTAAC TTTTGCCCTCTTGTCCTCAG ATCTAGCCTGTTTATACATT 17600

17601 TGGGTCATAGCTTGATCAGC CATCTTTTCCAACTTACGTT GCATGGCTGCATCACGGTCA AATTCAGATTTAGCCACATT CAAAGACTTCTTCAACTTTT 17700

17701 TAAGAACAACTTCAGAATCA CCATTAGCAACAGCCTGCTC ATAAGCTTCTTGAGCAGTAG CAAAAGCTGCATATGATGGA AGGGAACTAAACTCTGAGGC 17800

17801 TATAGCTTGTAAGGTTGCCC TGTTGTCCAGCATTTCTTCA CAAAGCTTGTTTATGTCTAC AGCACCCTGCATGGAAAGCA AAACAGAAAGTAGTGAAACC 17900

17901 ATTTTTTCAAAGGCTTCAGT AGTATCTTTAGCTAAGAGAA TGTCATTGTGTAACTGGACA CATTGAGCCCACAATTTAGA TAATGATTCTACTCTGAGTT 18000

18001 GTTGCAAAACTGAGAGTAAG ACTACTGATGTGCACTTTAC ATCTGACATTTTAGACTGTA CAGTGGCTACTTTGATACAA GGTTTGCCACCAACACCCAA 18100

18101 CAATTTAATGTTGAGTTTGA AGGCATCTATGCTATTCTTG GGTGGGAGTAGTCCCTGTGA ATTCATATATCTAAACTCCT GTGTAGAAACTAAGTAATCA 18200

18201 TAAACACCAAGAGTCAGTCT AAAGTAGCGGTTGAGTAAAC AAAAGAGGCCAAAGTAACAA GTACAAAAATAGCCTAAGAA ACAATAAACTAGCATTATAC 18300

18301 ACTGAAGTGTATTACCAGTT ATGAAGAAAATAGGGCAATA CTCAACACACATAAAAACAA TACCTCTGGCCAAAAACATG ACAGTTGTAACTACACCTGA 18400

18401 GTAGTTAGAAGTAACAGAGA TTATAAGAGCCCACATGGAA ATGGCTTGATCTAAAGCATT ACCATAATAAACTTTATAAA CGAGTGTCAAGACATTCATA 18500

18501 AGTGTCCACACTCTCCTAGC ACCATCATCATACACAGTTC TTGCTGTCATAAGGATTAGT AACACTACAGCTGATGCATA CATAACACAGTCTTTTAGCT 18600

18601 TAAAACCAGACAAACTAGTA TCAACCATATCCAACCATGT CATAATACGCATCACCCAAC TAGCAGGCATATAGACCATA TTAAAATAAGCTACAGTGGC 18700

18701 AAGAGAAGGTAACAAAAACA AACAGAGAAATGCATGCTTA TGTTTGACAAACATCATTGC AAAAGCAGACATAGCAATAA TACCCATAGCAAAAGGTAAA 18800

18801 AAGGCATTTTCATACAAAAA AAAGAACAAAGACCATTGAG TACTCTGGACTAAAACTAAA AGTGAAGTCAAAATTGTGAG TAACAACCAGTGGTGTGTAC 18900

18901 CCTTGATTGTTCTTTTCACT GCACTTTGGAAAGTAACACC TGAGCATTGTCTAACAACAT CAAAAGGTGTAAATTCATCT TCTAATAAAGCACTACCCAA 19000

19001 TATGGTACGTCCATTCATAC CATTTTGCAGTAATTCTTTT AATGAAGCACACATATCTAA AACGGCAATTCCAGTTTGAG CAGAAAGAGGTCCTAGTATG 19100

19101 TCAACATGGTCTTGTGTTAG AGGTTCATAATTGTACTTCA TAGCCACAAGGTTAAAGTCA TTAAGAGTTGTGGTAAATCG ATTGAGAAACCACCTGTCTC 19200

19201 CATTTATAACAGCAGCGTAC AACCAAGCTAAAACATTAAC TGTAATAGTTGTGTCCGTAC CAGCTGCTTGTGCTGTTTGC CTGTCAACAAAAGGTCCATA 19300

19301 AAAGTTACCTTCTAAGTCTG TGCCAGCATGAACTCCAGTT GGTAATTCCATATGGTGCAT GTAACAAAAAGAGACACAGT CATAATCTATGTTAAAACCA 19400

19401 ACACTACCACATGAACCATT AAGGAATGAACCCTTAATAG TGAAATTGGGCCTCATAGCA CATTGGTAAACACCAGATGG TGAACCATTGTAACAAGCTA 19500

19501 ACACTGAAAAAGTCTGTCCT GGTTGAATGCGAACAAACTT ATACTTAGGTGTCTTAGGAT TGGCTGTATCAACCTTAAGC TTAAGTACACAATTTTGCAT 19600

19601 AGAATGTCCAATAACCCTGA GTTGAACATTACCAGCCTGT ACCAAGAAATTATGATTAGA CTTACGAATGAGTAAATCTT CATAATTAGGGTTAAGCATG 19700

19701 TCTTCAGAGGTGCAGATCAC ATGTCTTGGACAGTAAACTA CGTCATCAAGCCAAAGACCG TTAAGTGTAGTTGTACCACA AGTTACTTGTACCATACAAC 19800

19801 CCTCAACTTTACCAGATGGG AATGCCATTTTTCTAAAACC ACTCTGCAAAACAGCTGAGG TGATAGAGGTTTGTGGTGGT TGGTAAAGAACATCAGAACC 19900

19901 TGAGTTACTGAAGTCATTGA GAGCCTTTGCGAGATGACAA CAAGCAGCTTCTCTGTAGCT AGTTGTATCCATTGCTCCAC TAAAATACTTGTACTTATTA 20000

20001 TAAAGAGCTAAGTATCTATT ATATTGCGTAAGAGGTAATA GCACATCACTACGCAACTTT AGATACATTTCTTTATTTAA CAAAAAGGTGCACAGCGCAG 20100

20101 CTTCTTCAAAAGTACTAAAG GAAACACCATTAAAGACTAC ACGTCTCTTTAGGTAATTAC TAAAGAACCAATAGAAATGC TTTGTGGAAATACAAATGAT 20200

20201 ATAAGCAATTGTTATCCAGA AAGGTACTAAAGGTGTGAAC ATAACCATCCACTGAATATG TGCTAAAAAAGAAACATCAT TAGTAAGATAAAATGTCAAG 20300

20301 TACAAGTAAATAACAGAATA AACACCAGGTAAGAATGAGT AAACTGGTGTTAAACAGAGT ACAGTGAATGACATAAGGAA TAGTAAAGTATTAAAGGCAA 20400

20401 CTACATGACTGTATTCACCA AAAGCTCTTCTAAACCTCAT AAAATAGTAGGCAAGGCATG TTACTACGATAGCTACAATA CCACCAGCTACTATAGATGC 20500

20501 TGATATGTCCAAAGCACCAA TAGGTTGAATTAGTGGTGTA AACATATTAGTAAGTAAATT TACAGCATCTACACCACAGA AAACTCCTGGTAAAGATCTG 20600

20601 TAATAATCATTGTTAAGTAC CCATCTACCACTAGTAGATA CACAAACACCAGCTTCTGAT CTTTCACAAGTGCCGTGCCT ACAGTACTCAGAATCAAAAG 20700

20701 TTGTTACCACTCTAACAGAA CCTTCAAGGTAGGTGTTAGG AAATTGAATAATAGAGCCAT CCATGAGCACATAACGTGTG TCAGGGCGTAAACTTTCATA 20800

20801 AGCAACAGAACCTTCTAGTA CATTGGTATCATAACAATAT GGTACTGGCTTACCAGAAGC ATCTTTAAAAATTGTACATT CAGCAGCCAAAACACAAGCT 20900

20901 GATGTTGCAAAGTCAGTGTA CTCTATAAGTTTTGATGGTG TGTAACAGATGTTACCAACT GCACTAAAAACTCTAGGTAA GAAATGCAAAAAGTCACCAT 21000

21001 TAGTTGTGCGTAATATCGTG CCAGGCAAACCAGGCACGAC AAAACCCACTTCTCTTGTTA TGACTGCAGCAATCAATGGG CAAGCTTTGTCATTAGTATA 21100

21101 ACTACCACCACGCTGGCTAA ACCATGTGTCAAAATCAGCA TGTTTGTTAGCAAAACAAGT ATCTGTAGATGCTATGTCAC GAGTGACACCACCATCAATA 21200

21201 GCCTTGTATCCTATGATTTC ACTTGAAAAGTCAGTATGTT TAGACATGACATGAACAGGT GTTATTAAATAGAAAATAGC AGCAACAAAAAGGAACACAA 21300

21301 GTGTAACTTTAATTAACTGC TTCAACCAATTATTAACAAT TTTACCACCCTTAAGTGCTA TCTTTGTTGTTACAACATTA ACAACTTGTCTAGTAGTTGC 21400

21401 ACATGTCAACTTAAAAGGTA AGTTATTCTTTTTAGCAGCA CTACGTATTTGTTTTCGTAG TTGTTCAGACAATGACATGA AATCTTTAACGTTCCATATC 21500

21501 AAAGCAATGTTGTGACTTTT TGCTACCTGCGCATTAATAT GACGCGCACTACAGTCAATA CAAGCACCAAGGTCACGGGG TGTCATGTTTTCAACTTTGT 21600

21601 TATAGGTGAGCATATAGTTA TTACAACTATCGCCAGTAAC TTCTATGTCAGATTGATGTG ACAATTTAAGACATTCAACA ACATCTTTAGTTTCTACATC 21700

21701 TGAATCAACAAACCCTTGCC GAGCTGCTGAAATAAAAGTA GATAAGACATTGTCTAAGGA CACATTCTTTGCAAGTTCAG CTTCTGCAGTTGCAACTAGT 21800

21801 GTTTTGAGTTTTTCCATTGG TACGTTAAAAGTTGATGAAA ACGTATTAACGTAAGCATCA AACATTTTAACTGCAACTTC CGCACTATCACCAACATCAG 21900

21901 ACACTAATGCCTGATCTAGT AACAGTATAGGTTGACACAT AAGCTGACTGTAGTAAACAG ACGCTGATTTTGCAGATGAT TCTTCACATTTTGATTTACC 22000

22001 ATCAAAAACTATAACATTAA TAGGCAATGAACCTTTAGTG TTATTAGCTCTCAGGTTGTC TAAGTTAACAAAATGAGAGA GAGAATGTCTTTCATAAGTC 22100

22101 TTTTGACCAGCTTTATCAAA GTAAAGATGGATGGAACCAT TCTTCACTGTAACACTATCA ACGATGTAAGAAGACTGGTC AGTAGGATTTATTGGTCTTT 22200

22201 TAAACTGTAGTGACAAGTCT CTCGCAACTTCATCACTAAT AAATGTACTACCAGCACAGA ATGTATCACAATTAACACAA TTCCAATTGTGTAGTTTGCA 22300

22301 AAAGCCTTTACCTCCATTAG CATAGACATAAAAGGACCTT CTAACACCATTAACAATAGT TGTACATTCGACTCTTGTTG CTCTATTACGTTTGTAACAC 22400

22401 ATCATACAAGTTGATGAATT ACAACCGTCTACAACATGCA CATAACTTTTCCATACATAA TAAAATGATGCAAAGAAGAT GTACATTCTAACCATAGCTG 22500

22501 AAATCGGGGCCATTTGTACA AGATTAATTATTAACCACAT AAGCCAAGAATTACTAATAA AATGTACTGCAAAATAGCTG AAAAACAATTGCATGATTGC 22600

22601 AGCCAATCCAAGTACATAGA AAAACCTAGTGAAAAGAATA TATGCCAAAAACCACTCTGC AACTAAGCCAAAAGCAGTTA AATCCCATTTAAAAGATGAA 22700

22701 ATGGTAATTTGTATAGTTTC TAAAGAAGGATAGGTGTCTA AAGAATCTAAACCACTAAGA CAAACACTACAAGGTATAGA ACCAGTACAGTAGGTTGCAA 22800

22801 TAGTGACATTAGTAGAGTTC AAATAGCCTTCTCTGTAACC AGTACAGTAAGAAGGCATGC CTAAATTAGACATTAAAACA CCTAAAGCAGCGGTTGAGTA 22900

22901 GATTAAAGAACCTAGGCAAA CACTTAATAGTAAAAACCAA ATTATAATATTTATCAGTTT AGAAAAATTAGGTGACTTCA AATAATTAAATGAAGCCTCT 23000

23001 AGACAAAATTTACCGACACT CTTAACAGTATTCTTTGCTA TAGTAGTCGGCATAGATGCT TTAATTCTAGAATTTGTACT TCTAGTAAAAGTACACAATT 23100

23101 GTAGCAATAAAGTAAAGAAA TAAGGCATATAATTAGTACA AACACGGTTTAAACACCGTG TAACTATGTTAGTAGTTGTA CTAACAACTTTGTTAAGAAA 23200

23201 AGGCTTAGCATAATTAGCTA TAGTATCCCAAGGGACACTA TTAACAGCAGCTAAACCATG AGTAGCAAGGGTTTTCAAAC CTAATACTCTAGATAATTCA 23300

23301 TTAGGTTTCTTAATAGTAAG ACTAGAATTGTCTACATAAG CAGCCATTAGATCTGTGTGG CCAACCTCTTCTGTAATTTT TAAACTATTATTTGCTGGTT 23400

23401 TAAGTATAATGTCTCCTACA ACTTCGGTAGTTTTCACATT ACACTCAAGAACGTCTTTCT GTATGGTAGGATTTTCCACT ACTTCTTCAGAGACTGGTTT 23500

23501 TAGATCTTCGCAGGCAAGAT TATCCATTCCCTGCGCGTCC TCTGACTTCAGTACATCAAA CGAATTTGATGTTTCAACTG GTTTTGTGCTCCAAAGACAA 23600

23601 CGTATACACCAGGTATTTGG TTTATACGTGGCTTTATTAG TTGCATTGTTAACATGCCAA ACAATAGGTTTATGTAACAA TTTAGCTCCTTTCTTAAAAG 23700

23701 AGGGTGTGTAGTGTTTATAA TCAATAGCCACCACATCACC ATTTAAGTCAGGGAAAAATG TAACTTTAAGCTCTCTTGAA GCAGGTTTCTTATAACCAGT 23800

23801 TAACTGGTTTAAATCATCAG CAAATTTGATATTATCACAT ACAAACTTAAAATTATCGAA GCTTGCGTTTGGATATGGTT GGTTTGGTACAAGATCAATT 23900

23901 GGTTGCTCTGTGAAATAAGA ATTGTCTTTCTTATAATAAT TGTCCAACTTAGGGTCAATT TCTGTACAAACAACACCATC CAATTTATAAGTAACTGGTT 24000

24001 TTATGGTTGTTGTGTAACTG TTTTCTTTGTAGAAAACATC CGTAATAGGACCTTTGTATT CTGAGGACTTTGTAAGTAAA GCACCGTCTATGCAATACAA 24100

24101 AGTTTCTTTAGAAGTTATAT GTTTATAGTGACCACACTGG TAATTACCAGTGTACTCACT AGCACAAGTAAATGTACCAT GCTTAAGTTCATACTGAGCA 24200

24201 GGTGGTGCTGACATCATAAC AAAAGGTGACTCCTGTTGTA CTAGATATTTTGTAGCTTGT TTACCACACGTACAAGGTAT CTGAACACCTTTCTTAAATT 24300

24301 GTTCATAAGAAAGTGTGCCC ATGTACATAACAGCTTCTAC ACCCTTAAGGGTTGTCTGCT GTTGTCCACAAGTTTTACAC ACCACGTTCAAGACTCTTTT 24400

24401 GCAAGAATCTAAATTGGCAT GTTGAAACAAGTAACTCATT GTTTCTCTAACATCACCTAA CTCACCTACTGTCTTATTAC AGTAGGCTAAGATAAGTGCA 24500

24501 CAAAAGTTAGCAGCTTCACC AGCCCTTGCTCTGTAATAAG CATCTTGTAGAGCAGGTGGA TTAAACTTCAACTCTATTTG TTGGAGTGTTAACAATGCAG 24600

24601 TGGCAAGATAACAGTTGTTA TCTGCCCATTTAATAGAAGT TAAACCATTAACTTGTGGGT ATTTCCACTTTTTAGTGTGA TTTAATGCTGACATGTACCT 24700

24701 ACCCAGAAAACTAGGATCAG TTGTGTGGTAGTACTCAAAA GCCTCAACACGTAGAGTGTC ATCATTAGGTAAAACATAAA ATGTTTTACCTTCATGTGAA 24800

24801 TTATGAGGTTTTATTTTAGT AACATCAGCTCCATCCAAAT AAGTTGGACCAAACTGTTGT CCATATGTCATTGACATGTC CACAACTTGCGTGTGGAGGT 24900

24901 TAATGTTGTCTACTGTTGTA AACACCTTAATAGTCCTCAC TTCTCTCAAAGAAAGAAGTG TCTTAAGATTGTCAAAGGTG ATAACTTCACCATCTAGGTG 25000

25001 GAATGTGGTAGGATTACTAG TGTAATATACACTTTTATCA CCTCTCTTAAGAAATTCTAT ACCTAGTTGTGTAGATTGTC CAGAATAGGACCAATCTTTA 25100

25101 TAGGAACCAGCAAGTGAGAT GGTTTCAATAAAATGTTCTT CAGGTGTTTTAGAAGAAGAA GTAAGATAACCATTATACGC TGTAACAGCATCAGGTGAAG 25200

25201 AAACAGAAACTGTAGCTGGC ACTTTGAGAGATCTCATATA CCGAGCAGCTTCTTCCAAAT TTAAGCCATGTGTTACATAG CCAAGTGGCATTGTAACAAG 25300

25301 AGTTTCATTTAGATCGTTAA GTGTGTTGATAAGTGACGCT ACAGTTGTTTTACTGGTGTA AAAGTAAAATCTAGCACCAT AATCAACCACACCCTCTTGT 25400

25401 ATTTTAATACCCTTATATTT ACGCTGTATAGTTGAAACTA TGGCTTTAGTTTCCACACAG ACAGGCATTAATTTGCGTGT TTCTTCTGCATGTGCAAGCA 25500

25501 TTTCTCGCAAATTCCAAGAA ACAGTTCCAAGAATTTCTTG CTTCTCATTAGAGATAATAG ATGGTAGAATGTAAAAGGCA CTTTTACACTTTTTAAGCAC 25600

25601 TGTCTTTGCCTCCTCTACAG TGTAACCATTTAAACCCTGA CCCGGGTAAGTGGTTATATA ATTGTCTGTTGGCACTTTTC TCAAAGCTTTCGCTAGCATT 25700

25701 TCAGTAGTGCCACCAGCCTT TTTAGTAGGTATAACCACAG CAGTTAAAACACCCTCTTGA ACAACATCACCCACTATATA TGGAGCATCTTTCTTTAAGA 25800

25801 AAGTGATGTCAATGTCACTA ACAAGAGTGGCAGAATCTGG ATGAAGATTGCCATTAATGT CAATATAAAGTAACAAGTTT TCTGTGAGGAACTTAGTTTC 25900

25901 TTCCAGAGTTGTTGTAACTT CTTCAACACAAGCTTTGATT TTCTTATCATCTTGTTTTCT CTGTTCAACTGAAGGTTTAC TTTCAGTTATAAATGGCTTA 26000

26001 ACTTCCTCTTTAGGAATCTC AGCGATCTTTTGTTCAACTT GCTTTTCACTCTTCATTTCC AAAAAGCTTGAAACAAGTTT GTCATAGAGATTTTTATCAA 26100

26101 AGACAGCTAAGTAGACATTT GTGCGAACAGTATCTACACA AACTCTTAAAGAATGTATAG GGTCAGCACCAAAAATACCA GCTGATAATAATGGTGCAAG 26200

26201 TAGAACTTCGTGCTGATTAA AATTTTCATAAGCACTCTTA AGAAGTTGAATGTCTTCACC TTTGTTAACATTTGGGCCGA CAACATGAAGACAGTGTTTA 26300

26301 GCAAGATTGTGTCCGCTTAA AACACAACTACCACCCACTT TAAGTGGTCCATTAGTAGCT ATGTAATCATCAGATTCAAC TTGCATGGCATTGTTAGTAG 26400

26401 CCTTATTTAAGGCTCCTGCA ACACCTCCTCCATGTTTAAG GTAAACATTGGCTGCATTAA CAACCACTGTTGGTTTTACC TTTTTAGCTTCTTCCACAAT 26500

26501 GTCTGCATTTTTAATGTATA CATTGTCAGTAAGTTTTAAA TAACCACTAAAACTATTCAC TTCAATAGTCTGAACAACTG GTGTAAGTTCCATCTCTAAT 26600

26601 TGAGGTTGAACCTCAACAAT TGTTTGAATAGTAGTTGTCT GATTGTCCTCACTGCTGTCT TGTTGACCAACAGTTTGTTG ACTATCATCATCTAACCAAT 26700

26701 CTTCTTCTTGCTCTTCTTCA GGTTGAAGAGCAGCAGAAGT GGCACCAAATTCCAAAGGTT TACCTTGGTAATCATCTTCA GTACCATACTCATATTGAGT 26800

26801 TGATGGCTCAAACTCTTCTT CTTCACAATCACCTTCTTCT TCATCCTCATCTGGAGGGTA AAAAGAACAATACATATGTG AAGCCAATTTAAACTCACCA 26900

26901 GACTCATCAAATAAGTAGTA TGTAGCCATACTCCACTCAT CTAAATCAATGCCCAGTGGT GTAAGTAATTCAGATACTGG TTGCAAAGTTTTTATGACAG 27000

27001 CATCTGCCACAACACAGGCG AACTCATTTACTTCTGTACC GAGTTCAACTGTATAGGCAG AGCACTTCTCATTAAGTACT TTATCAATCCTTTCATCAAG 27100

27101 TTCAAAAGTGATATTCACAC TCTTGTAACCTTGCACTTCT ATCACAGTGTCATCACCAAA AGTAACCTTTGTTGGTGCAC CGCCTTTGAGTGTGAAGGTA 27200

27201 TTGTTTGTTACCATCATATT AGGTGCAAGGGCACAGTACT TTTCTGTGTCTTTGATTTCG AGCAACATAAGCCCGTTAAT ACAAACTGGTGTACCAACCA 27300

27301 ATGGAGCTTCAACAGCTTCA CTAGTAGGTTGTTCTAATGG TTGTAAATCACCAGTTTTCA AGACAACTTCCTCTGTTAAC ACTTCTGTGGGAAGTGTTTC 27400

27401 TCCCTCTAAGAAGATAATTT CTTTTGGGGCTTTTAGAGGC ATGAGTAGGCCAGTTTCTTC TCTGGATTTAACACACTTTC TGTACAATCCCTTTGAGTGC 27500

27501 GTGACAAATGTTTCACCTAA ATTCAAGGCTTTAAGTTTAG CTCCACCAATAATGATAGAG TCAGCACACAAAGCCAAAAA TTTATTTACAAGCTTAAAGA 27600

27601 ATGTCTGAACACTCTCCTTA ATTTCCTTTGCACAGGTGAC AATTTGTCCACCGACAATTT CACAAGCACAGGTTGAGATA AATTTAACAATTTCCCAACC 27700

27701 GTCTCTAAGAAACTCTACAC CTTCCTTAAACTTCTCTTCA AGCCAATCAAGGACGGGTTT GAGTTTTTCATAAACAGTGC CAAAGATGTTAGTTAGCCAC 27800

27801 TGCGAAGTCAACTGAACAAC ACCACCTGTAATGTAGGCCA TTACAACTAGATTGTTAGTA GCCAAATCAGATGTGAACAT CATAGCATCAATGAGTCTCA 27900

27901 GTGAATACTGTGAAATTCCA TCTAGTATTGTTATAGCGGC CTTCTGTAAAACACGCACAG AATTTTGAGCAGTTTCAAGA GTGCGGGAGAAAATTGATCG 28000

28001 TACAACACGAGCAGCCTCTG ATGCAAATGCATAAAGAGGA CTCAGTATTGATTTCTGTTC ACCAATATTCCAGGCACCTT TTTTAGCTTTTCCTTTTGTA 28100

28101 ACTTTAAAATTACCACAGGA TTCAACAATTTGTTTGAATG CTTTATAATCCAAACCTTTA ACAGTTTCCACAAAAGCACT TGTGGAAGCAGAAAAAGATG 28200

28201 CCAAAATAATGGCGATCTCT TCATTAAGTTTAAAGTCACC AACAATATTGATGTTGACTT TCTCTTTTTGGAGTATTTCA AGAAGGTTGTCATTAAGACC 28300

28301 TTCGGAACCTTCTCCAACAA CACCTGTATGGTTACAACCT ATGTTAGCGCTAGCACGTGG AACCCAATAGGCACACTTGT TATGGCAACCAACATAAGAG 28400

28401 AACACACAGCCTCCAAAGGC AATAGTGCGACCACCCTTAC GAAGAATGGTTTTCAAGCCA GATTCATTATGGTATTCGGC AAGACTATGCTCAGGTCCTA 28500

28501 CTTCTGAATTGTAACATGCT GGACAATAAATTTTAACAAC AGCATTTTGGGGTAAGTAAC CACAAGTAGTGGCACCTTCT TTAGTCAAATTCTCAGTGCC 28600

28601 ACAAAATTCGCAAGTGGCTT TAACAAAATCGCCCGTCTGC CATGAAGTTTCACCACAATG ATCACACTTCATGAGAGTTG AAAGGCACATTTGGTTGCAT 28700

28701 TCATTTGGTGACGCAACTGG ATAGACAGATCGAATTCTAC CCATAAAGCCATCAAGCTTT TTCTTTTCAACCCTTGGTTG AATAGTCTTGATTATGGAAT 28800

28801 TTAAGGGAAATACAAAATTT GGACATTCCCCATTGAAGAT GTCAAATTTCTTTGCCAATT TAATTTCAAAAGGTGTCTGC AATTCATAGCTCTTTTCAGA 28900

28901 ACGTTCCGTGTACCAAGCAA TTTCATGCTCATGTTCACGG CAGCAGTATACACCCCTCTT AGTGTCAATAAAGTCCAGTT GTTCGGACAAAGTGCATGAA 29000

29001 GCTTTACCAGCACGTGCTAG AAGGTCTTTAATGCACTCAA GAGGGTAGCCATCAGGGCCA CAGAAGTTGTTATCGACATA GCGAGTGTATGCCCCTCCGT 29100

29101 TAAGCTCACGCATGAGTTCA CGGGTAACACCACTGCTATG TTTAGTGTTCCAGTTTTCTT GAAAATCTTCATAAGGATCA GTGCCAAGCTCGTCGCCTAA 29200

29201 GTCAAATGACTTTAGATCGG CGCCGTAACTATGGCCACCA GCTCCTTTATTACCGTTCTT ACGAAGAAGAACCTTGCGGT AAGCCACTGGTATTTCGCCC 29300

29301 ACATGAGGGACAAGGACACC AAGTGTCTCACCACTACGAC CGTACTGAATGCCTTCGAGT TCTGCTACCAGCTCAACCAT AACATGACCATGAGGTGCAG 29400

29401 TTCGAGCATCCGAACGTTTG ATGAACACATAGGGCTGTTC AAGTTGAGGCAAAACGCCTT TTTCAACTTCTACTAAGCCA CAAGTGCCATCTTTAAGATG 29500

29501 TTGACGTGCCTCTGATAAGA CCTCCTCCACGGAGTCTCCA AAGCCACGTACGAGCACGTC GCGAACCTGTAAAACAGGCA AACTGAGTTGGACGTGTGTT 29600

29601 TTCTCGTTGAAACCAGGGAC AAGGCTCTCCATCTTACCTT TCGGTCACACCCGGACAAAA CCTAGATGTGCTGATGATCG GCTGCAACACGGACGAAACC 29700

29701 GTAAGCAGCCTGCAGAAGAT AGACGAGTTACTCGTGTCCT GTCAACGACAGTAATTAGTT ATTAATTATACTGCGTGAGT GCACTAAGCATGCAGCCGAG 29800

29801 TGACAGCCACACAGATTTTA AAGTTCGTTTAGAGAACAGA TCTACAAGAGATCGAAAGTT GGTTGGTTTATACCTTCCCA GGTAACAAACCAACCAACTT 29900

29901 TCGATCTCTTGTAGATCTGT TCTCT 29925

1.14. SRR12596175 Discovered Strain 1 Nucleotide Sequence

1 AGAGAACAGATCTACAAGAG ATCGAAAGTTGGTTGGTTTG TTACCTGGGAAGGTATAAAC CAACCAACTTTCGATCTCTT GTAGATCTGTTCTCTAAACG 100

101 AACTTTAAAATCTGTGTGGC TGTCACTCGGCTGCATGCTT AGTGCACTCACGCAGTATAA TTAATAACTAATTACTGTCG TTGACAGGACACGAGTAACT 200

201 CGTCTATCTTCTGCAGGCTG CTTACGGTTTCGTCCGTGTT GCAGCCGATCATCAGCACAT CTAGGTTTTGTCCGGGTGTG ACCGAAAGTTAAGATGGAGA 300

301 GCCTTGTCCCTGGTTTCAAC GAGAAAACACACGTCCAACT CAGTTTGCCTGTTTTACAGG TTCGCGACGTGCTCGTACGT GGCTTTGGAGACTCCGTGGA 400

401 GGAGGTCTTATCAGAGGCAC GTCAACATCTTAAAGATGGC ACTTGTGGCTTAGTAGAAGT TGAAAAAGGCGTTTTGCCTC AACTTGAACAGCCCTATGTG 500

501 TTCATCAAACGTTCGGATGC TCGAACTGCACCTCATGGTC ATGTTATGGTTGAGCTGGTA GCAGAACTCGAAGGCATTCA GTACGGTCGTAGTGGTGAGA 600

601 CACTTGGTGTCCTTGTCCCT CATGTGGGCGAAATACCAGT GGCTTACCGCAAGGTTCTTC TTCGTAAGAACGGTAATAAA GGAGCTGGTGGCCATAGTTA 700

701 CGGCGCCGATCTAAAGTCAT TTGACTTAGGCGACGAGCTT GGCACTGATCCTTATGAAGA TTTTCAAGAAAACTGGAACA CTAAACATAGCAGTGGTGTT 800

801 ACCCGTGAACTCATGCGTGA GCTTAACGGAGGGGCATACA CTCGCTATGTCGATAACAAC TTCTGTGGCCCTGATGGCTA CCCTCTTGAGTGCATTAAAG 900

901 ACCTTCTAGCACGTGCTGGT AAAGCTTCATGCACTTTGTC CGAACAACTGGACTTTATTG ACACTAAGAGGGGTGTATAC TGCTGCCGTGAACATGAGCA 1000

1001 TGAAATTGCTTGGTACACGG AACGTTCTGAAAAGAGCTAT GAATTGCAGACACCTTTTGA AATTAAATTGGCAAAGAAAT TTGACATCTTCAATGGGGAA 1100

1101 TGTCCAAATTTTGTATTTCC CTTAAATTCCATAATCAAGA CTATTCAACCAAGGGTTGAA AAGAAAAAGCTTGATGGCTT TATGGGTAGAATTCGATCTG 1200

1201 TCTATCCAGTTGCGTCACCA AATGAATGCAACCAAATGTG CCTTTCAACTCTCATGAAGT GTGATCATTGTGGTGAAACT TCATGGCAGACGGGCGATTT 1300

1301 TGTTAAAGCCACTTGCGAAT TTTGTGGCACTGAGAATTTG ACTAAAGAAGGTGCCACTAC TTGTGGTTACTTACCCCAAA ATGCTGTTGTTAAAATTTAT 1400

1401 TGTCCAGCATGTCACAATTC AGAAGTAGGACCTGAGCATA GTCTTGCCGAATACCATAAT GAATCTGGCTTGAAAACCAT TCTTCGTAAGGGTGGTCGCA 1500

1501 CTATTGCCTTTGGAGGCTGT GTGTTCTCTTATGTTGGTTG CCATAACAAGTGTGCCTATT GGGTTCCACGTGCTAGCGCT AACATAGGTTGTAACCATAC 1600

1601 AGGTGTTGTTGGAGAAGGTT CCGAAGGTCTTAATGACAAC CTTCTTGAAATACTCCAACA AGAGAAAGTCAACATCAATA TTGTTGGTGACTTTAAACTT 1700

1701 AATGAAGAGATCGCCATTAT TTTGGCATCTTTTTCTGCTT CCACAAGTGCTTTTGTGGAA ACTGTGAAAGGTTTGGATTA TAAAGCATTCAAACAAATTG 1800

1801 TTGAATCCTGTGGTAATTTT AAAGTTACAAAAGGAAAAGC TAAAAAAGGTGCCTGGAATA TTGGTGAACAGAAATCAATA CTGAGTCCTCTTTATGCATT 1900

1901 TGCATCAGAGGCTGCTCGTG TTGTACGATCAATTTTCTCC CGCACTCTTGAAACTGCTCA AAATTCTGTGCGTGTTTTAC AGAAGGCCGCTATAACAATA 2000

2001 CTAGATGGAATTTCACAGTA TTCACTGAGACTCATTGATG CTATGATGTTCACATCTGAT TTGGCTACTAACAATCTAGT TGTAATGGCCTACATTACAG 2100

2101 GTGGTGTTGTTCAGTTGACT TCGCAGTGGCTAACTAACAT CTTTGGCACTGTTTATGAAA AACTCAAACCCGTCCTTGAT TGGCTTGAAGAGAAGTTTAA 2200

2201 GGAAGGTGTAGAGTTTCTTA GAGACGGTTGGGAAATTGTT AAATTTATCTCAACCTGTGC TTGTGAAATTGTCGGTGGAC AAATTGTCACCTGTGCAAAG 2300

2301 GAAATTAAGGAGAGTGTTCA GACATTCTTTAAGCTTGTAA ATAAATTTTTGGCTTTGTGT GCTGACTCTATCATTATTGG TGGAGCTAAACTTAAAGCCT 2400

2401 TGAATTTAGGTGAAACATTT GTCACGCACTCAAAGGGATT GTACAGAAAGTGTGTTAAAT CCAGAGAAGAAACTGGCCTA CTCATGCCTCTAAAAGCCCC 2500

2501 AAAAGAAATTATCTTCTTAG AGGGAGAAACACTTCCCACA GAAGTGTTAACAGAGGAAGT TGTCTTGAAAACTGGTGATT TACAACCATTAGAACAACCT 2600

2601 ACTAGTGAAGCTGTTGAAGC TCCATTGGTTGGTACACCAG TTTGTATTAACGGGCTTATG TTGCTCGAAATCAAAGACAC AGAAAAGTACTGTGCCCTTG 2700

2701 CACCTAATATGATGGTAACA AACAATACCTTCACACTCAA AGGCGGTGCACCAACAAAGG TTACTTTTGGTGATGACACT GTGATAGAAGTGCAAGGTTA 2800

2801 CAAGAGTGTGAATATCACTT TTGAACTTGATGAAAGGATT GATAAAGTACTTAATGAGAA GTGCTCTGCCTATACAGTTG AACTCGGTACAGAAGTAAAT 2900

2901 GAGTTCGCCTGTGTTGTGGC AGATGCTGTCATAAAAAGTT TGCAACCAGTATCTGAATTA CTTACACCACTGGGCATTGA TTTAGATGAGTGGAGTATGG 3000

3001 CTACATACTACTTATTTGAT GAGTCTGGTGAGTTTAAATT GGCTTCACATATGTATTGTT CTTTTTACCCTCCAGATGAG GATGAAGAAGAAGGTGATTG 3100

3101 TGAAGAAGAAGAGTTTGAGC CATCAACTCAATATGAGTAT GGTACTGAAGATGATTACCA AGGTAAACCTTTGGAATTTG GTGCCACTTCTGCTGCTCTT 3200

3201 CAACCTGAAGAAGAGCAAGA AGAAGATTGGTTAGATGATG ATAGTCAACAAACTGTTGGT CAACAAGACGGCAGTGAGGA CAATCAGACAACTACTATTC 3300

3301 AAACAATTGTTGAGGTTCAA CCTCAATTAGAGATGGAACT TACACCAGTTGTTCAGACTA TTGAAGTGAATAGTTTTAGT GGTTATTTAAAACTTACTGA 3400

3401 CAATGTATACATTAAAAATG CAGACATTGTGGAAGAAGCT AAAAAGGTAAAACCAACAGT GGTTGTTAATGCAGCCAATG TTTACCTTAAACATGGAGGA 3500

3501 GGTGTTGCAGGAGCCTTAAA TAAGGCTACTAACAATGCCA TGCAAGTTGAATCTGATGAT TACATAGCTACTAATGGACC ACTTAAAGTGGGTGGTAGTT 3600

3601 GTGTTTTAAGCGGACACAAT CTTGCTAAACACTGTCTTCA TGTTGTCGGCCCAAATGTTA ACAAAGGTGAAGACATTCAA CTTCTTAAGAGTGCTTATGA 3700

3701 AAATTTTAATCAGCACGAAG TTCTACTTGCACCATTATTA TCAGCTGGTATTTTTGGTGC TGACCCTATACATTCTTTAA GAGTTTGTGTAGATACTGTT 3800

3801 CGCACAAATGTCTACTTAGC TGTCTTTGATAAAAATCTCT ATGACAAACTTGTTTCAAGC TTTTTGGAAATGAAGAGTGA AAAGCAAGTTGAACAAAAGA 3900

3901 TCGCTGAGATTCCTAAAGAG GAAGTTAAGCCATTTATAAC TGAAAGTAAACCTTCAGTTG AACAGAGAAAACAAGATGAT AAGAAAATCAAAGCTTGTGT 4000

4001 TGAAGAAGTTACAACAACTC TGGAAGAAACTAAGTTCCTC ACAGAAAACTTGTTACTTTA TATTGACATTAATGGCAATC TTCATCCAGATTCTGCCACT 4100

4101 CTTGTTAGTGACATTGACAT CACTTTCTTAAAGAAAGATG CTCCATATATAGTGGGTGAT GTTGTTCAAGAGGGTGTTTT AACTGCTGTGGTTATACCTA 4200

4201 CTAAAAAGGCTGGTGGCACT ACTGAAATGCTAGCGAAAGC TTTGAGAAAAGTGCCAACAG ACAATTATATAACCACTTAC CCGGGTCAGGGTTTAAATGG 4300

4301 TTACACTGTAGAGGAGGCAA AGACAGTGCTTAAAAAGTGT AAAAGTGCCTTTTACATTCT ACCATCTATTATCTCTAATG AGAAGCAAGAAATTCTTGGA 4400

4401 ACTGTTTCTTGGAATTTGCG AGAAATGCTTGCACATGCAG AAGAAACACGCAAATTAATG CCTGTCTGTGTGGAAACTAA AGCCATAGTTTCAACTATAC 4500

4501 AGCGTAAATATAAGGGTATT AAAATACAAGAGGGTGTGGT TGATTATGGTGCTAGATTTT ACTTTTACACCAGTAAAACA ACTGTAGCGTCACTTATCAA 4600

4601 CACACTTAACGATCTAAATG AAACTCTTGTTACAATGCCA CTTGGCTATGTAACACATGG CTTAAATTTGGAAGAAGCTG CTCGGTATATGAGATCTCTC 4700

4701 AAAGTGCCAGCTACAGTTTC TGTTTCTTCACCTGATGCTG TTACAGCGTATAATGGTTAT CTTACTTCTTCTTCTAAAAC ACCTGAAGAACATTTTATTG 4800

4801 AAACCATCTCACTTGCTGGT TCCTATAAAGATTGGTCCTA TTCTGGACAATCTACACAAC TAGGTATAGAATTTCTTAAG AGAGGTGATAAAAGTGTATA 4900

4901 TTACACTAGTAATCCTACCA CATTCCACCTAGATGGTGAA GTTATCACCTTTGACAATCT TAAGACACTTCTTTCTTTGA GAGAAGTGAGGACTATTAAG 5000

5001 GTGTTTACAACAGTAGACAA CATTAACCTCCACACGCAAG TTGTGGACATGTCAATGACA TATGGACAACAGTTTGGTCC AACTTATTTGGATGGAGCTG 5100

5101 ATGTTACTAAAATAAAACCT CATAATTCACATGAAGGTAA AACATTTTATGTTTTACCTA ATGATGACACTCTACGTGTT GAGGCTTTTGAGTACTACCA 5200

5201 CACAACTGATCCTAGTTTTC TGGGTAGGTACATGTCAGCA TTAAATCACACTAAAAAGTG GAAATACCCACAAGTTAATG GTTTAACTTCTATTAAATGG 5300

5301 GCAGATAACAACTGTTATCT TGCCACTGCATTGTTAACAC TCCAACAAATAGAGTTGAAG TTTAATCCACCTGCTCTACA AGATGCTTATTACAGAGCAA 5400

5401 GGGCTGGTGAAGCTGCTAAC TTTTGTGCACTTATCTTAGC CTACTGTAATAAGACAGTAG GTGAGTTAGGTGATGTTAGA GAAACAATGAGTTACTTGTT 5500

5501 TCAACATGCCAATTTAGATT CTTGCAAAAGAGTCTTGAAC GTGGTGTGTAAAACTTGTGG ACAACAGCAGACAACCCTTA AGGGTGTAGAAGCTGTTATG 5600

5601 TACATGGGCATACTTTCTTA TGAACAATTTAAGAAAGGTG TTCAGATACCTTGTACGTGT GGTAAACAAGCTACAAAATA TCTAGTACAACAGGAGTCAC 5700

5701 CTTTTGTTATGATGTCAGCA CCACCTGCTCAGTATGAACT TAAGCATGGTACATTTACTT GTGCTAGTGAGTACACTGGT AATTACCAGTGTGGTCACTA 5800

5801 TAAACATATAACTTCTAAAG AAACTTTGTATTGCATAGAC GGTGCTTTACTTACAAAGTC CTCAGAATACAAAGGTCCTA TTACGGATGTTTTCTACAAA 5900

5901 GAAAACAGTTACACAACAAC CATAAAACCAGTTACTTATA AATTGGATGGTGTTGTTTGT ACAGAAATTGACCCTAAGTT GGACAATTATTATAAGAAAG 6000

6001 ACAATTCTTATTTCACAGAG CAACCAATTGATCTTGTACC AAACCAACCATATCCAAACG CAAGCTTCGATAATTTTAAG TTTGTATGTGATAATATCAA 6100

6101 ATTTGCTGATGATTTAAACC AGTTAACTGGTTATAAGAAA CCTGCTTCAAGAGAGCTTAA AGTTACATTTTTCCCTGACT TAAATGGTGATGTGGTGGCT 6200

6201 ATTGATTATAAACACTACAC ACCCTCTTTTAAGAAAGGAG CTAAATTGTTACATAAACCT ATTGTTTGGCATGTTAACAA TGCAACTAATAAAGCCACGT 6300

6301 ATAAACCAAATACCTGGTGT ATACGTTGTCTTTGGAGCAC AAAACCAGTTGAAACATCAA ATTCGTTTGATGTACTGAAG TCAGAGGACGCGCAGGGAAT 6400

6401 GGATAATCTTGCCTGCGAAG ATCTAAAACCAGTCTCTGAA GAAGTAGTGGAAAATCCTAC CATACAGAAAGACGTTCTTG AGTGTAATGTGAAAACTACC 6500

6501 GAAGTTGTAGGAGACATTAT ACTTAAACCAGCAAATAATA GTTTAAAAATTACAGAAGAG GTTGGCCACACAGATCTAAT GGCTGCTTATGTAGACAATT 6600

6601 CTAGTCTTACTATTAAGAAA CCTAATGAATTATCTAGAGT ATTAGGTTTGAAAACCCTTG CTACTCATGGTTTAGCTGCT GTTAATAGTGTCCCTTGGGA 6700

6701 TACTATAGCTAATTATGCTA AGCCTTTTTCTTAACAAAGT TGTTAGTACAACTACTAACA TAGTTACACGGTGTTTAAAC CGTGTTTGTACTAATTATAT 6800

6801 GCCTTATTTCTTTACTTTAT TGCTACAATAGTGTACTTTT ACTAGAAGTACAAATTCTAG AATTAAAGCATCTATGCCGA CTACTATAGCAAAGAATACT 6900

6901 GTTAAGAGTGTCGGTAAATT TTGTCTAGAGGCTCGATTTA ATTATTTGAAGTCACCTAAT TTTTCTAAACTGATAAATAT TATAATTTGGTTTTTACTAT 7000

7001 TAAGTGTTTGCCTAGGTTCT TTAATCTACTCAACCGCTGC TTTAGTAGTTTTAATGTCTA ATTTAGGCATGCCTTCTTAC TGTACTGGTTACAGAGAAGG 7100

7101 CTATTTGAACTCTACTAATG TCACTATTGCAACTTACTGT ACTGGTTCTATACCTTGTAG TGTTTGTCTATGTGGTTTAG ATTCTTTAGACACTAACACT 7200

7201 TCTTTAGAAACTATACAAAT TACCATTTCATCTTTTAAAT GGGATTTAACTGCTTTTGGC TTAGTTGCAGAGTGGTTTTT GGCATATATTCTTTTCACTA 7300

7301 GGTTTTTCTATGTACTTGGA TTGGCTGCAATCATGCAATT GTTTTTCAGCTTCTTTGCAG TACATTTTATTAGTAATTCT TGGCTTATGTGGTTAATAAT 7400

7401 TAATCTTGTCAAAATGGCCC CGATTTCAGCTATGGTTAGA ATGTACATCTTCTTTGCATC ATTTTATTATGTATGGAAAA GTTATGTGCATGTTGTAGAC 7500

7501 GGTTGTAATCAATCAACTTG TATGATGTGTTACAACCGTA ATAGAGCAACAAGAGTCGAA TGTACAACTATTGTTAATGG TGTTAGAAGGTCCTTTTATG 7600

7601 TCTATGCTAATGGAGGATAA GGCTTTTGCAAACTACACAA TTGGAATTGTGTTAATTGTG ATACATTCTGTGCTGGTAGT ACATTTATTAGTGATGAAGT 7700

7701 TGCGAGAGACTTGTCACTAC GTTTTAAAAGACCAATAAAT CCTACTGACAAGTCTTCTTA CATCGTTGATAGTGTTACAG TGAAGAATGGTTCCATCCAT 7800

7801 CTTTACTTTGATAAAGCTGG TCAAAAGACTTATGAAAGAC ATTCTCTCTCTCATTTTGTT AACTTAGACAACCTGAGAGC TAATAACACTAAAGGTTCTA 7900

7901 TGCCTATTAATGTTATAGTT TTTGATGGTAATCAAAAATG TGAAGAATCATCTGCAAAAT CAGCGTCTGTTTACTACAGT CAGCTTATGTGTCAACCTAT 8000

8001 ATAGTTACTAGATCAGGCAT TAGTGTCTGATGTTGGTGAT AGTGCGGAAGTTGCAGTTAA AATGTTTGATGCTTACGTTA ATACGTTTTCATCAACTTTT 8100

8101 AACGTACCAATAGAAAAACT CAAAACACTAGTTGCAACTG CAGAAGCTGAACTTGCAAAG AATGTGTCCTTAGACAATGT CTTATCTATGTTTATTTCAG 8200

8201 CAGCTCGGCAAGGGTTTGTT GATTCAGATGTAGAAACTAA AGATGTTGTTGAATGTCTTA AATTGCTACATCAATCTGAC ATAGAAGTTACTGGCGATAG 8300

8301 TTGTAATAACTATATGCTCA CCTATAACAAAGTTGAAAAC ATGACACCCCGTGACCTTGG TGCTTGTTTTGACTGTAGTG CGCGTCATATTAATGCGCAG 8400

8401 GTAGCAAAAAGTCACAACAT TGCTTTGATATGGAACGTTA AAGATTTCATTCCATTGTCT GAACAACTACGAAAACAAAT ACGTAGTGCTGCTAAAAAGA 8500

8501 ATAACTTACCTTTTAAGTTG ACATGTGCAACTACTAGACA AGTTGTTAATGTTGTAAATA CAAAGATAGCACTTAAGGGT GGTAAAATTGTTAATAATTG 8600

8601 GTTGAAGCAGTTAATTAAAG TTACACTTGTGTTCTTTTTT GTTGCTGCTATTTTCTATTT AATAAATCCTGTTCATGTCA TGTCTAAACATACTGACTTT 8700

8701 TCAAGTGAAATCATAGGATA CAAGGCTATTGATGGTGGTG TCACTCGTGACATAGCATCT ACAGATACTAGTTTTGCTAA CAAACATGCTGATTTTGACA 8800

8801 CATGGTTTAGCCAGCGTGGT GGTAGTTATACTAATGACAA AGCTTGCCCATTGATTGCTG CAGTCATAACAAGAGAAGTG GTTTTTGTCGTGCCTGGTTT 8900

8901 GCCTGGCACGATATTACGCA CAACTAATGGTACCTTTTTG CATTTCTTACCTAGAGTTTT TAGTGCAGTTGGTAACATCT GTTACACACCATCAAAACTA 9000

9001 TTAGAGTACACTGACTTTGC AACATCAGCTTGTGTTTTGG CTGCTGAATGTACAATTTTT AAAGATGCTTCTGGTAAGCC AGTACATTATTGTTATGATA 9100

9101 CCAATGTACTAAAAGGTTCT GTTGCTTATGAAAGTTTACG CCCTGACACACGTTATGTGC TCATGGATGGCTCTATTATT CAATTTCCTACAACTAACCT 9200

9201 TGAAGGTTCTGTAAGAGGCG TAACAACTTTTGATTCTGAG TACTGTAGGCACGGCACTTG TGAAAGATCAGAAGCTGGTG TTTGTGTATCTACTAGTGGT 9300

9301 AGATGGGTACTTAACAATGA TTATTACAGATCTTTACCGT GAGTTTTCTGTGGTGTAGAT GCTGTAAATTTACTTACTAA TATGTTTACACCACTAATTC 9400

9401 AACCTATTGGTGCTTTGGAC ATATCAGCATTAATAGTAGC TGGTGGTATTGTAGCTATCG TAGTAACATGCCTTGCCTAC TATTTTATGAGGTTTAGAAG 9500

9501 AGCTTTTGGTGAATACAGTC ATGTGCTTGCCTTTAATACT TTACTATTCCTTATGTCATT CACTTCACTCTGTTTAACAC CAGTTTACTCATTCTTACCT 9600

9601 GGTGTTTATTCTGTTATTTA CTTGTACTTGACATTTTATC TTACATATGATGTTTCTTTT TTAGCACATATTCAGTGGAT GGTTATGTTCACACCTTTAG 9700

9701 TACCTTTCTGGATAACAATT GCTTATATCATTTGTATTTC ATCATAGCATTTCTATTGGT TCTTTAGTAATTACCTAAAG AGACGTGTAGTCTTTAATGG 9800

9801 TGTTCCCTTTAGTACTTTTG AAGAAGCTGCGCTGTGCACC TTTTTGTTAAATAAAGAAGT GTATCTAAAGTTGCGTAGTG ATGTGCTATTACCTCTTACG 9900

9901 CAATATAATAGATACTTAGC TCTTTATAATAAGTACAAGT ATTGTAGTGGAGCAATGGAT ACAACTAGCTACAGAGAAGC TGCTTGTTGTCATCTCGCAA 10000

10001 AGGCTCTCAATGACTTCAGT AACTCAGGTTCTGATGTTCT TTACCAACCACCACAAACCT CTATCACCTCAGCTGTTTTG CAGAGGTGTTTTAGAAAAAT 10100

10101 GGCATTCCCATCTGGTAAAG TTGAGGGTTGTATGGTACAA GTAACTTGTGGTACAACTAC ACTTAACGGTCTTTGGCTTG TAGACGTAGTTTACTGTCCA 10200

10201 AGACATGTGATCTGCACCTC TAGAGACATGCTTAACCCTA ATTATGAAGATTTACTCATT CGTAAGTCTAATCATAATTT CTTGGTACAGGCTGGTAATG 10300

10301 TTCAACTCAGGGTTATTGGA CATTCTATGCAAAATTGTGT ACTTAAGTTTAAGGTTGATA CAGCCAATCCTAAGACACCT AAGTATAAGTTTGTTCGCAT 10400

10401 TCAACCAGGACAGACTTTTT CAGTGTTAGCTTGATACAAT GGTTCACCATCTGGTGTTCA CCAATGTGCTATGAGGCCCA ATTTCACTATTAAGGGTTCA 10500

10501 TTCCTTAATGGTTCTGGTGG TAGTGTTGGTTTTAACATAG ATTATGACTGTGTCTCTTTT TGTTACATGCACCATATGGA ATTACCAACTGGAGTTCATG 10600

10601 CTGGCACAGACTTAGAAGGT AACTTTTAGTGACCTTTTGT TGACAGGCAAACAGCACAAG CAGCTGGTACGGACACAACT ATTACGATTAATGTTTTAGC 10700

10701 TTGGTTGTACGCTGCTTCTA TAAATGGAGACAGGTGGTTT CTCAATCGATTTACCACAAC TCTTAATGACTTTAACCTTG TGGCTATGAAGTACAATTAT 10800

10801 GAACCTCTAACACAAGACCA TGTTAGCATACTAGGACCTC TTTCTGCTCAAACTGGAATT GCCGTTTTAGATATGTGTGC TTCATTAAAAGAATTACTGC 10900

10901 AAAATGGTATGAATGGACGT ACCATTTTGGGTAGTGCTTT ATTAGAAGATGAATTTACAC CTTTTGATGTTGTTAGACAA TGCTCAGGTGTTACTTTCCA 11000

11001 AAGTGCAGTGAAAAGAACAA TCAAGGGTCGACACCACTGG TTGTTACTCACAATTTTGAC TTCACTTTTAGTTTTAGTCC AGAGTACTCAATGGTCTTTG 11100

11101 TTCTTTTTTTGTATGAAAA TGCCTATATACCTTTTGCTA TGGTAATTATTGCTATGTCT GCTTTTGCAATGATGTTTGT CAAACATAAGCATGCATTTC 11200

11201 TCTGTTTGTTTTTGTTACCT TCTTATGCCACTGTAGCTTA TTTTAATATGGTCTATATGC CTGCTAGTAGGGTGATGCGT ATTATGACATGGTTGGATAT 11300

11301 GGTTGATACTAGTTTGTCTG GTTTTAAGCTAAAAGACTGT GTTATGTATGCATCAGCTGT AGTGTTACTAATCCTTATGA CAGCAAGAACTGTGTATGAT 11400

11401 GATGGTGCTAGGAAGGTGTG GACACTTATGAATGTCTTGA CACTCGTTTATAAAGTTTTA TATGGTAATGCTTTAGATCA AGCCATTTCCTCGTGGGCTC 11500

11501 TTATAATCTCTGTTACTTCT AACTACTCAGGTGTAGTTAC AACGATCATGTTTTTGGCCA GAGGTATTGTTTTTATGTGT GTTGAGTATTGCCCTATTTT 11600

11601 CTTCATAACTGGTAATACAC TTCATAGTATAATGTAAGTT TATTGTTTCTTAGGCTATTT TTGTACTTGTTACTTTGGCC TCTTTTGTTTACTCAACCGC 11700

11701 TATCTTAGACTGACTCTTGG TGTTTATGATTACTTAGTTT CTACACAGGAGTTTAGATAT ATGAATTCACAGGGACTACT CCCACCCAAGAATAGCATAG 11800

11801 ATGCCTTCAAACTCAACATT AAATTGTTGGGTGTTGGTGG AAAACCTTGTATCAAAGTAG CCACTGTACAGTCTAAAATG TCAGATGTAAAGTGCACATC 11900

11901 AGTAGTCTTACTCTCAGTTT TGCAACAACTCAGAGTAGAA TCATCATCTAAATTGTGGGC TCAATGTGTCCAGTTACACA ATGACATTCTCTTAGCTAAA 12000

12001 GATACTACTGAAGCCTTTGA AAAAATGGTTTCACTACTTT CTGTCTTGCTTTCCATGCAG GGTGCTGTAGACATAAACAA GCTTTGTGAAGAAATGCTGG 12100

12101 ACAACAGGGCAACCTTACAA GCTATAGCCTCAGAGTTTAG TTCCCTTCCATCATATGCAG CTTTTGCTGCTGCTCAAGAA GCTTATGAGCAGGCTGTTGC 12200

12201 TAATGGTGATTCTGAAGTTG TTCTTAAAATGTTGAAGAAG TCTTTGAATGTGGCTAAATC TGAATTTGACCGTGATGCAG CCATGCAACGTAAGTTGGAA 12300

12301 AAGATGGCTACTCAAGCTAT GACCCAAATGTATAAACAGG CTAGATCTGAGGACAAGAGG GCAAAAGTTACTAGTGCTAT GCAATCAATGCTTTTCACTA 12400

12401 TGCTTAGAAAGTTGGATAAT GATGCACTCAACAACATTAT CAACAATGCAAGAGATGTAT GTGTTCCCTTGAACATAATA CCTCTTACAACAGCAGCCAA 12500

12501 ACTAATGGTTGTCATACCAG ACTATAACACATATAAAAAT ACGTGTGATGTCACAACATT TACTTATGCATCAGCATTGT GGGAAATCCAACAGGTTGTA 12600

12601 GATGCAGATAGTAAAATTGT TCAACTTAGGAAAATTAGTA TGGACAATTCACCTAATTTA GCATGGCCTCTTATTGTAAC AGCTTTAAGGGCCAATTCTG 12700

12701 CTGTCAAATTACAGAATAAT GAGCTTAGCACTGTTGCACT ACGACAGATGTCTTGTGCTG CCGGTACTACACAAACTGCT TGCACTGATGACAATGCGTT 12800

12801 AGCTTACTACAACACAACAA AGGGAGTTAGGTTTGTACTT GCACTGTTATCCGATTTACA GGATTTGAAATGGGCTAGAT TCCCTAAGAGTGATGGACCT 12900

12901 GGTACTATCTATACAGAACT GGAACCACCTTGTAGGTTTG TTACAGACACACCTAAAGGT CCTAAAGTGAAGTATTTATA CTTTATAAAAGGATTAAACA 13000

13001 ACCTAAATAGAGGTATGGTA CTTGGTAGTTTAGCTGCCAC AGTACGTCTACAAGCTGGTA ATGCAACAGAAGTGCCTGCT AATTCAACTGTATTATCTTT 13100

13101 CTGTGCTTTTGCTGTAGATG CTGCTAAAGCTTACAAAGAT TATCTAGCTAGTGGGGGACA ACCAATCACTAATTGTGTTA AACTGTTGTGTACACACACT 13200

13201 GGTACTGGTCAGGCATATAC AGTTACACCGGAAGCCAATA TGGATCAAGAATCCTTTGGT GGTGCATCGTGTTGTCTGTA CTGCCGTTGCCACATGTATC 13300

13301 ATCCAAATCCTAAAGGATTT TGTGACTTAAAAAGGTAAGT ATGTACAAATACCTACAACT TGTGCTAATGACCCTGTGGG TTTTACACTTAAAAACACAG 13400

13401 TCTGTACTGACTGCGGTATG TGGAAAGGTTATGGCTGTAG TTGTGATCAACTCCGCGAAC CCATGCTTCAGTCAGCTGAT GCACAATCGTTTTTAAACGG 13500

13501 GTTTGCGGTGTAAGTGCAGC CTGCCTTACACCGTGCGGCA CAGGCACTAGTACTGATGTC GTATACAGGGCTTTTGACAT CTACAATGATAAAATTGCTG 13600

13601 GTTTTGCTAAATTCCTAAAA ACTAATTGTTGTCGCTTCCA AGTAAAGGACGAAGATGACA ATTTAATTGATTCTTACTTT GTAGTTAAGAGACACACTTT 13700

13701 CTCTAACTACCAACATGAAG AAACAATTTATAATTTACTT AAGTAATGTCCAGCTGTTGC TAAACATGACTTCTTTAAGT TTAGAATAGACGGTGACATG 13800

13801 GTACCATACATATCACGTCA ACGTCTTACTAAATACACAA TGGCAGACCTCGTCTATGCT TTAAGGCATTTTGATGAAGG TAATTGTGACACATTAAAAG 13900

13901 AATTACTTGTCACATACAAT TGTTGTGATGATGATTATTT CAATAAAAAGGACTGGTATG ATTTTGTAGAAAGCCCAGAT ATATTACGCGTATACGCCAA 14000

14001 CTTAGGTGAACGTGTACGCC AAGCTTTGTTAAAAACAGTA AATTTCTGTGATGCCATGCA ATATGCTGGTATTGTTGGTA TTCTGACATTAGAATTTCAA 14100

14101 GATCTCAATGGTAACTGGTA TGATTTCGGTGATTTCATAC AAACCACGCCAGGTAGTGGA GTTCCTGTTGTAGATTCTTA TTATACTTTGTTAATGCCTA 14200

14201 TATTAACCTTGAACCGGGCT TTAACTGCAGAGTCACATGT TGACACTGACTTAACAAAGC CTTACATTAAGTGGGATTAG TTAAAATATGACTTCACGGA 14300

14301 AGAGAGGTTAAAACTCTTTG ACCGTTATTTAAATATTGG GATCAGACATACCACCCAAA TTGTTTTAACTGTTTGGATG ACAGATGCATTCTGCATTGT 14400

14401 GCAAACTTTAATGTTTTATT CTCTACAGTGTTCCCACTTA CAAGTTTTGGACCACTAGTG AGAAAAATATTTGTTGATTG CGTTCCATTTGTAGTTTCAA 14500

14501 CTGGATACCACTTCAGAGAG CTAGGTGTTGTACATAATCA GGATGTAAACTTACATAGCT CTAGACTTAGTTTTAGGTAA TTACTTGTGTATGCTGCTGA 14600

14601 CCCTGCTATGCACGCTGCTT CTGGTAATCTATTACTAGAT AAACGCACTACGTGCTTTTC AGTAGCTGCACTTACTAACA GTCTTGCTTTTCAAACTGTC 14700

14701 AAACCCGGTAATTTTAACAA AGACTTCTATGACTTTGCTG TGTCTAAGGGTTTCTTTAAG GAAGGAAGTTCTGTTGAATT AAAACACTTCTTCTTTGCTC 14800

14801 AGGATGGTAATGCTGCTATC AGCGATTATGACTATTATCG TTATAATCTACCAACAATGT GTGATATCAGACAACTACTA TTTGTAGTTGAAGTTGTTGA 14900

14901 TAAGTTCCTTGATTGTTACG ATGGTGGCTGTATTAATGCT AACCAAGTCATCGTCAACAA CCTAGACAAATCAGCTGGTT TTCCATTTAATAAAGGTGGT 15000

15001 AAGGCTAGACTTTATTATGA TTCAATGAGTTATGGGTATC AAGATGCACTTTTCGCATAT ACAAAACGTAATGTCATCCC TAC-ATAACTCAAATGAATC 15100

15101 TTAAGTATGCCATTTGCGCA AAGAATAGAGCTCGCACCGT AGCTGGTGTCTCTATCTGTA GTTCAATGACCAATAGACAT TATCCTAAAAAAATTTTGAA 15200

15201 ATCAATAGCCGCCACTAGAG GAGCTACTGTAGTAATTGGA ACAAGCAAATTCTATGGTGG TTGGCACAACATGTTAAAAA CTGTTTATAGTGATGTAGAA 15300

15301 AACCCTCACCTTATGGGTTG GGATTATTCCAAATGTGATA GAGCCATGCCTAACATGCTT AGAATTATGGCCTCACTTGT TCTTGCTCGCAAACATACAA 15400

15401 CGTGTTGTAGCTTGTCACAG CGTTTCTATAGATTAGCTAA TGAGTGTGCTCAAGTTTAGA GTGAAATGGTTAAGTGTGGC GGTTCACTATATGTTAAACC 15500

15501 AGGTGGAACCTCATCAGGAG ACGACACAACTGCTTATGCT AATAGTGTTTTTAACATTTG TCAAGCTGTCACGGCCAATG TTAATGCACTTTTATCTACT 15600

15601 GATGGTAACAAAATTGCCGA TAAGTATGTCCGCAATTTAC AACACACATTTTATGAGTGT CTCTATAGAAATAGAGATGT TGACACAGACTTTGTGAATG 15700

15701 AGTTTTACGCATATTTGCGT AAACATTTCTCAATGATGAT ACTCTCTGACGATGCTGTTG TGTGTTTCAATAGCACTTAT GCATCTCAAGGTCTAGGGGC 15800

15801 TAGCATAAAGAACTTTAAGT CAGTTCTTTATTATCAAAAC AATGTTTTTATGTCTGAAGC AAAATGTTGGACTGAGACTG ACCTTACTAAAGGACCTCGT 15900

15901 TAATTTTGCTCTCAACATAC AATGCTAGTTAAACAGGGTG ATGATTATGTGTCCTTTCCT TACCCAGATCCATCAAGAAT CCTAGGGGCCGGCTGTTTTG 16000

16001 TAGATGATATCGTAAAAACA GATGGTACATTAATGATTGA ACGGTTCGTGTCTTTAGCTA TAGATGCTTACCCACTTACT AAACATCCTAATCAGGAGTA 16100

16101 TGCTGATGTCTTTCATTTGT ACTTACAATACATAAGAAAG CTACATGATGAGTAATCAGG ACACATGTTAGACATGTATT CTGTTATGCTTACTAATGAT 16200

16201 AACACTTCAAGGTATTGAGT ACCTGAGTTTTATGAGGCTA TGTACACACCGCATACAGTC TTACAGGCTGTTGGGGCTTG TGTTCTTTGTAATTCACAGA 16300

16301 CTTCATTAAGATGTGGTGCT TGCATACGTAGACCATTCTT ATGTTGTAAATGGTTTTACG ACCATGTCATATCAACATCA CATAAATTACTATTGTCTGT 16400

16401 TAATCCGTATGTTTGCAATG CTCCAGGTTGTGATGTCACA GATGTGACTCAACTTTACTT AGGAGGTATGAGCTATTATT GTAAATCACATAAAACTCCG 16500

16501 ATTAGTTTTCCATTGTGTTC TAATGGACAAGTTTTTGGTT TATATAAAAATACATGTGTT GGTAGCGATAATGTTACTGA CTTTAATGCAATTTCAACAT 16600

16601 GTGACTGGACAAATGCTGGT GATTACATTTTAGCTAACAC CTGTACTGAACGACTCAAGC TTTTTGCAGCAGAAACGCTC AAAGCTAC-GAGGAGACATT 16700

16701 TAAACTGTCTTATGGTATTG CTACTGTACGTGAAGTTCAG TCCGACAGAGAATTACATCT TTCATGGGAAGTTGGTAAAC CTAGACCACCACTTAACCGA 16800

16801 AATTATGTCTTTACTGGTTA TCGTGTAACTAAAAACAGTA AAGTAAATATAGGAGAGTAC ACCTTTGAAAAAGGTGACTA TGGTGATTCCGTTGTTTACC 16900

16901 GAGGTACAACAACTTACAAA TTAAATGTGGTTGATTATTT TGTGCTGACATCACATACAG TAATGCCATTAAGTGCACCT ACACTAGTGCCACAAGAGCA 17000

17001 CTATGTTAGAATTTCAGGCT TATACCCAACACTCAATATC TCAGATGAGTTTTCTAGCAA TGTTGCAAATTATCAAAAGG TGGTTATGCAAAAGTATTCT 17100

17101 ACACTCCAGGGACCACCTGG TACTGGTAAGAGTCATTTTG CTATTGGCCTAGCTCTCAAC TACCCTTCTGCTCGCATAGT GTATACAGCTTGCTCTCATG 17200

17201 CCGCTGTTGATGCACTATGT GAGAAGGCATTAAAATATTT GTCAATAGATAAATGTAGTA GAATTATACCTGCACGTGCT CGTGTAGAGTGTTTAGATAA 17300

17301 ATTCAATGAGAATTCAACAT TAGAACAGTATGTCTTTTGT ACTGTAAATGCATTGCCTGA GACGACAGCAGATATAGTTG TCTTTGATGAATTATCAATG 17400

17401 GCCACAAATTATGATTTGAG TGTTGTCAATGCCAGATTAC GTGCTAAGCACTATGTGTAC ATTGGCGACCCTGCTCAATT ACCTGCACCACGCACATTGC 17500

17501 TAACTAAGGGCACACTAGAA CCAGAATATTTCAATTCAGT GTGCAGACTTATGAAAACTA TAGGTCCAGACATGTTCCTC GGAACTTGTCGGCGTTGTCC 17600

17601 TGCTGAAATTGTTGACACTG TGAGTGCTTTGTTCTATGAT AATAAGCTTAAAGCACATAA AGACAAATCAGCTCAATGCT TTAAAATGTTTTAAAAGTGA 17700

17701 TTAATCACGCATGATGTTTC ATCTGCAATTAACAGGCCAC AAATAGGCGTGGTAAGAGAA TTCCTTACACGTAACCCTGC TTGGAGAAAAGCTGTCTTTA 17800

17801 TTTCACCTTATAATTCACAG AATTCAGTAGCCTCAAAGAT TTTGGGACTACCAACTCAAA CTGTTGATTCATCACAGGGC TCAGAATATGACTATGACAT 17900

17901 ATTCACTCAAACCACTGAAA CAGCTCACTCTTGTAATGTA AACAGATTTAATGTTGCTAT TACCAGAGCAAAAGTAGGCA TACTTTGTACAATGTCTGAT 18000

18001 AGAGACCTTTATGACAAGTT GCAATTTACAAGTCTTGAAA TTCCACGTAGGAATGTGGCA ACTTTACAAGCTGAAAATGT AACAGGACTCTTTAAAGATT 18100

18101 GTAGTAAGGTAATCACTGGG TTACATCCTACACAGGCACC TACACCCCTCAGTGTTGACA CTAAATTCAAAACTGAAGGT TTATGTGTTGACATACCTGG 18200

18201 CATACCTAAGGACATGACCT ATAGAAGACTCATCTCTATG ATGGGTTTTAAAATGAATTC TAAAGTTAATGGTTACCCTA ACATGTTTATCACCCGCGAA 18300

18301 GAAGCTATAAGACATGTACG TGCATGGATTGGCTTCGATC TTGAGGGGTGTCATGCTACT AGAGAAGCTGTTGGTACCAA TTTACCTTTACAGCTAGGTT 18400

18401 TTTCTACAGGTGTTAACCTA GTTGCTGTACCTACAGGTTA TGTTGATACACCTGATAATA CAGATTTTTCCAGAGTTAGT GCTAAACCACCGCCTGGAGA 18500

18501 TCAATTTAAACACCTCATAC CACTTATGTACAAAGGACTT CCTTGGTACGTAGTGCGTAT AAAGATTGTACAAATGTTAA GTGACACACTTAAAAATCTC 18600

18601 TCTGACAGAGTCATGTGTCT GTTATGGGCACATGGCTTTG AGTTGACATCTATGAAGTAT TTTGTGAAAATAGGACCTGA GCGCACCTGTTGTCTATGTG 18700

18701 ATAGACGTGCCACATTCCTT TCCACTGCTTCAGACACTTA TGCCTGTTGGCATCATTCTA TTGGATTTGATTACGTCTAT AATCCGTTTATGATTGATGT 18800

18801 TCAAAAATGGGGTTTTACAG GTAACCTACAAAGCAACCAT GATCTGTATTGTCAAGTCCA TGGTAATGCACATGTAGCTA GTTGTGATGTAATCATGACT 18900

18901 AGGTGTCTAGCTGTCCACGA GTGCTTTGTTAAGCGTGTTG ACTGGACTATTGAATATCCT ATAATTGGAGTTGAACTGAA GATTAATGCGGCTTGTAGAA 19000

19001 AGGTTCAACACATGGTTGTA ATAGCTGCATTATTAGCAGA CAAATTCCCAGTTCTTCACG ACATTGGTAACCCTAAAGCT ATTAAGTGTGTACCTCAAGC 19100

19101 TGATGTAGAATAGTAGTTCT ATGATGCACAGCCTTGTAGT GACAAAGCTTATAAAATAGA AGAATTATTCTATTCTTATG CCACACATTCTGACAAATTC 19200

19201 ACAGATGGTGTATGTCAATT TTGGAATTGCAATGTCGATA GATATCCTGCTAATTCCATT GTTAGTAGATTTGACACTAG AGTGCTATCTAACCTTAACT 19300

19301 TGCCTGGTTGTGATGGTGGC AGTTTGTATGTAAATAAACA TGCATTCCACACACCAGCTT GTAATAAAAGTGCTTTTGTT AATTTAAAACAATTACCATT 19400

19401 TTTCTATTACTCTGACAGTC CATGTGAGTCTCATGGAAAA CAAGTAGTGTCAGATATAGA TTATGTACCACTAAGGTCTG CTACGTGTATAACACGTTGC 19500

19501 AATTTAGGTGGTGCTGTCTG TAGACATCATGCTAATGAGT ACAGATTGTATCTCGATGCT TATAACATGATGATCTCAGC TGGTTATAGCTTGTGGGTTT 19600

19601 ACAAACAATTTGATACTTAT AACCTCTAGGACACTTTTAC AAGACTTCAGAGTTTAGAAA ATGTGGCTTTTAATGTTGTA AATAAGGGACACTTTGATGG 19700

19701 ACAACGGTGTGAAGTACCAG TTTCTATCATTAATAACACT GTTTACACAAAATTAGATGG TGTTGATGTAGAATTGTGTA AAAATAAAACAACAATTCCT 19800

19801 GTTAATGTAGCATTTGAGCT TTGGGCTAAGCGCAACATTA AACCAGTACCAGAGGTGAAA ATACTCAAAAATTTGGGTGT GGACATTGCTGCTAATACTG 19900

19901 TGATCTGGGACTACAAAAGA GATGCTCCAGCACATATATC TACTATTGGTGTTAGTTCTA TGACTGACATAGCCAAGAAA CCAACTGAAACGATTTGTGC 20000

20001 ACCACTCACTGTTTTTTTTG ATGGTAGAGTTGATGGTCAA GTAGACTTATTTAGAAATGC CCGTAATGGTGTTCTTATTA CAGAAGGTAGTGTTAAAGGT 20100

20101 TTACAACCATCTGTAGGTCC CAAACAAGCTAGTCTTAATG GAGTCAGATTAATTGGAGAA GCCGTAAAAACACAGTTCAA TTATTATAAGAAAGTTGATG 20200

20201 GTGTTGTCCAACAATTACCT GAAACTTACTTTACACTGAG TAGAAATTTACAAGAATTAA AACCCAGGAGTCAAATGGAA ATTGATTTCTTAGAATTAGC 20300

20301 TATGGATGAATTCATTGAAC GATTTAAATTAGAAGGCTAT GCCTTCGAACATATCGTTTA TGGAGATTTTAGTCATAGTC AGTTAGGTGGTTTACATCTA 20400

20401 CTGATTGGACTAGCTAAACG TTTTAAGAATTCACCTTTTG AATTAGAAGATTTTATTCCT ATGGACAGTACAGTTAAAAA CTATTTCATAACAGATGCGA 20500

20501 ACACAGGTTCATCTAAGTGT GTGTGTTCTGTTATTGATTT ATTACTTGATTAATTTGTTG AAATAATAAAATCCCAAGAT TTATCTGTAGTTTCTAAGGT 20600

20601 TGTCAAAGTGACTATTGACT ATACGGAAATTTCATTTATG CTTTGGTGTAAAGATCGTCA TGTAGAAACATTTTACCCAA AATTACAATCTAGTCAAGCG 20700

20701 TGGCAACCGGGTGTTGCTAT GCCTAATCTTTACAAAACGA AAAGAATGCTATTAGAAAAG TGTGACCTTCAAAATTATGG TGATAGTGCAACATTACCTA 20800

20801 ATGGCATAATGATGAATGTC GCAAAATATACTCAACTGTG TCAATATTTAAACACATTAA CATTAGCTGTACCCTATAAT ATGAGAGTTATACTTGTTGG 20900

20901 TGCTGGTTCTGATAAAGGAG TTGCACCAGGTACAGCTGTT TTAAGACAGTGGTTGCCTAC GGGTACGCTGCTTGTCGTTC CAGATCTTAATGACTTTGTC 21000

21001 TCTGATGCAGATTCAACTTT GATTGGTGATTGTGCAACTG TACATACAGCTAATAAATGG GATCTCATTATTAGTGATAT GTACAACCCTAAGACTAAAA 21100

21101 ATGTTACAAAAGAAAATGAC TCTAAAGAGGGTTTTTCAC TTACATTTGTGGGTTTATAA ATCAATAGCTAGCTCTTGGA GGTTCCGTGGCTATAAGGAT 21200

21201 AACAGAACATTCTTGGAATG CTGATCTTTATAAGCTCATG GGACACTTCGCATGGTGGAC AGCCTTTGTTACTAATGTGA ATGCGTCATCATCTGAAGCA 21300

21301 TTTTTAATTGGATGTAATTA TCTTGGCAAACCATGCGAAC AAATAGATGGTTATGTCATG CATGCAAATTACATATTTTG GAGGAATACAAATCCAATTC 21400

21401 AGTTGTCTTCCTATTCTTTA TTTGACATGAGTAAATTTCC CCTTAAATTAAGGGGTACTG CTTTCATGTCTTTAAAAGAA GGTCAAATCAATGATATGAT 21500

21501 TTTATCTCTTCTTAGTAAAG GTAGACTTATAATTAGAGAA AACAACAGAGTTGTTATCTA TAGTGATGTTCTTGTTAACA ACAATACGAACAATGTTTGT 21600

21601 TTTTCTTGTTTTATTGCCAC TAGTCTCTAGTCAGTGTGTT AATCTTAAATCCAGAACTCA ATTACCCCCTGCATACACTA ATTCTTTCACACGTGGTGTT 21700

21701 TATTACCCAGGCAAAGTTTT CAGATCCTCAGTTTTACATT CAACTCAGGACTTGTTCTTA CCTTTCTTTTCCAATGTTAC TTGGTTCCATGCTATACATG 21800

21801 TCTCTGGGACAATTGGTACT AAGAGGTTTGATAACCCTGT CCTACCATTTAATGATGGTG TTTATTTTGCTTCCACTGAG AAGTCAATCATAATAAGAGG 21900

21901 CTGGATTTTTGGTACTACTT TAGATTCGAAGACCCAGTCC CTACTTATTGTTAATAACTC AACTAATGTTGTTATTAAAG TCTGTGAATTTCAATTTTGT 22000

22001 AATGATCCATTTTTGGGTGT TTATTACCACAAAAACAACA AAAGTTGGATGGAAAGTGAG TTCAGAGTTTATTCTAGTGC GAATAATTGCACTTTTGAAT 22100

22101 AGGTCTCTCAGCCTTTTCTT ATGGACCTTGAAGGAAAACA GGGTAATTTCAAAAATCTTA GGGAATTTGTGTTTAAGAAT ATTGATGGTTATTTTAAAAT 22200

22201 ATATTCTAAACACACGCCTA TTAATTTAGTGCGTGATCTC CCTCAGGGTTTTTCGGCTTT AGAACCATTGGTAGATTTGC CAATAGGTATTAACATCACT 22300

22301 AGGTTTCAAATTATACTTTC ATTACATAGAAGTTATTTGA CTCCTGGTGATTCTTCTTCA GGTTGGACAGCTGGTGCTGC AGCTTATTATGTGGGTTATC 22400

22401 TTCAACCTAGGTCATTTCTA TTAAAATATAATGAAAATGG AACCATTACAGATGCTGTAG ACTGTGCACTTGACCCTCTC TCAGGAACAAAGTGTACGTT 22500

22501 GAAATCCTTCACTGTAGAAA AAGGAATCTATCAAACTTCT AACTTTAGAGTCCAACCAAC AGAATCTATTGTTAGATTTC CTAATATTAAATACTTGTGC 22600

22601 CCTTTTGGTGAAGTTTTTAA CGCCACCAGATTTGCATCTG TTTATGCTTGGAACAGGAAG AGAATCAGCAACTGTGTTGC TGATTATTCTGTCCTATATA 22700

22701 ATTCCGTACCATTTTCCACT TTTAAGTGTTATGGAGTGTC TCCTACTAAATTAAATGATC TCTGCTTTACTAATGTCTAT GCAGATTCATTTGTAATTAG 22800

22801 AGGTGATGAAGTCAGACAAA TCGCTCCAGGACAAACTGGA AAGATTGCTGATTATAATTA TAAATTACCAGATGATTTTA CAGGCTGCGTTATAGCTTGG 22900

22901 AATTCTAACAATCTTGATTC TAAGGTTGGTGGTAATTATA ATTACCTATATAGATTGTTT AGGAAGTCTAATCTCAAACC TTTTGAGAGAGATATTTCAA 23000

23001 CTGAAATCTATCAGGCCGGT AGCACACCTTGTAATGGTGT TGAAGGTTTTAATTGTTACT TCCTTTTACAATTATATGGT TTCCAACCCACTAATGGTGT 23100

23101 TGGTTACCAACCATACAGAG TAGTAGTACTTTCTTTTGAA CTTCTACATGCACCAGCAAC TGTTTGTGCATCTAAAAAGT CTACTAATTTGGTTAAAAAC 23200

23201 AAATGTGTCAATTTCAACTT CAATGGTTTAACAGGCACAG GTGTTCTTACTGAGTCTAAC -AAAAGTTTCTGCCTTTTCA ACAATTTGGCAGAGACATTG 23300

23301 CTGACACTACTGATGCTGTC CGAGGTCCACAGACACTTGA GATTCTTGACATTACACCAT GTTCTTTTGGTGGTGTCAGT GTTATAACGCCAGGAACAAA 23400

23401 TACTTCTAACCAGGTTGCTG TTCTTTATCAGGGTTTCAAC TGCACAGAAGTCCCTGTTGC TATTCATGCAGATCAACTTA CTCCTACTTGGCGTGTTTAT 23500

23501 TCTACAGGTTCTTAAGTTTC TAAAACACGTGCAGGCTGTT TAATAGGGGCTGAACATGTC AACAACTCATATGAGTGTGA CATACCCATTGGTGCAGGTA 23600

23601 TATGCGCTAGTTATCAGACT CAGTCTAATTCTCCTCGGCG GGCACGTAGTGTAGCTAGTC AATCCATCATTGCATTCACT ATGTCACTTGGTGCAGAAAA 23700

23701 TTCAGTTGCTTACTCTAATA ACTCTATTGCCATACCCACA AATTTTACTATTAGTGTTAC CACAGAAATTCTACCAGTGT CTATGACCAAGACATCAGTA 23800

23801 GATTGTACAATGTACATTTG GGATGATTCAACTGAATGCA GCAATTTATTGTTGCAATAT GGCAGTTTTTGTACACAATT AAACCGTGCTTTAACTGGAA 23900

23901 TAGCTGTTGAACAAGACAAA AACACCCAAGAAGTTTGTAC ACAAGTCAAACAAATTTACA AAACACCACCAATTAAAGAT TTTGGTGGTTTTAATTTTTC 24000

24001 ACAAATAATTCCAGATCCAT CAAAACCAAGCAAGAGGTCA TTTATTGAAGATCTACTTTT CAACAAAGTGACACTTGCAG TTGCTGGCTTCATCAAACAA 24100

24101 TATGGTGATTGCCTTGGTGA TATTGCTGCTAGAGCCCTCA TTTGTGCACAAAAGTTTAAC GGCCTTACTGTTTTGCCACC TTTGCTCACAGATAAGATGA 24200

24201 TTGCTCAATACACTTCTGCA CTGTTAGCGGGTACTATCAC TTCTGGTTGGACCTTTGGTG CAGGTGCTGCATTACAAATA CCATGTACTATGCAAATGGC 24300

24301 TTATAGGTTTAATGGTATTG GAGTTACACAGAATGTTCTC TATGAGAACCAAAAATTGAT TGCCAACCAATTTAATAGTG CTATTGGCAAAATTCAAGAC 24400

24401 TCACTTTCTTCCACAGCAAG CGAACTTGGAAAACTTCAAG ATGTGGTCAACCAAAATGCA CAAGCTTTAAACACGCTTGT TAAACAACTTAGCTCCAATT 24500

24501 TTGGTGCAATTTCAAGTGTT TTAAATGATATCCTTTCACG TCTTGACAAAGTTGAGGCTG AAGTGCAAATTGATAGGTTG ATCACAGGCAGACTTCAATG 24600

24601 TTTGCAGACATATGTGACTC AACAATTAATTAGAGCTGCA GAAATCAGAGCTTCTGCTAA TCTTGCTGCTACTAAATTGT CAGAGTGTGTACTTGGACAA 24700

24701 TCAAAAAGAGTTGATTTTTG TGGAAAGGGCTATCATCTTA TGTCCTTCCCTCAGTCAGCA CCTCATGGTGTAGTTTTCTT GCATGTGACTTATGTCCCTG 24800

24801 CACAAGAAAAGAACTTCACA ACTGCTCCTGCCATTTGTCA TGATGGAATAGCACACTTTC CTCGTGAAGGTGTCTTTGTT TCAAATGGCACACACTGGTT 24900

24901 TGTAACACAAAGGAATTTTT ATGAACCACAAATCATTACT ACAGACAACACATTTGTGTC TGGTAACTGTGATGTTGTAA TAGGAAGTCTCAACAACACA 25000

25001 GTTTATGATCCTTTGCAACC TGAATTAGACTCATTCAAGG AGGAGTTAGATAAATATTTT AAGAATCATACATCACCAGA TGTTGATTTAGGTGACATCT 25100

25101 CTGGCATTAATGCTTCAGTT GTAAACTTACAAAAAGAAAT TGACCGCCTCAATGAGGTTG CAATGAATTTAAATGAATCT CTCATCGATCTCCAAGAACT 25200

25201 TGGAAAGTATGAGCAGTATA T-AAATGGCCATGGTACATT TGGCTAGGTTTTATAGCTGG CTTGATTGCCATAGTAATGG TGACAATTATGCTTTGCTGT 25300

25301 ATGACCAGTTGCTGTAGTTG TCTCAAGGGCTGTTGTTCTT GGGTATCCTGCTGCAAATTT GATGAAGACGACTCTGAGCC AGTGCTCAAAGGAGTCAAAT 25400

25401 TACATTACACATAAACGAAC TTATGGATTTGTTTATGAGA ATCCTCACAATTGGAACTGT AACTTTGAAGCAAGGTGAAA TCAAGGATGCTACTCCTTCA 25500

25501 GATTTTGTTCGCTCCACTGC AACGATACCGATACAAGCCT CACTCCCTTTCGGATGGCTT ATTGTTGGCGTTGCACTTCT TGCTGTTTTTCATAGCGCTT 25600

25601 ACTAAATCATAACCCTCAAA AAGAGATGGCAACTAGCACT CTCCAAGGGTGTTCACTTTG TTTGCAACTTGCTGTTGTTG TTTGTAACAGTTTACTCACA 25700

25701 CCATTTGCTCGTTGCTGCTG GCCTTGAAGCCCCTTTTTTC TATCTTTATGCTTTAGTCTA CTTCTTGCAGAGTATAAATT ATGTAAGAATAATAATGAGT 25800

25801 CATTGGCTTTGCTGGAAATG CTGATCCAAAAACCCATTAC TTTATGATGACTACTATTTT CTTTGCTGGCATACTAATTG TTACGACTATTGTATACCTT 25900

25901 ACAATAGTGTAACTTCTTCA ATTGTCATTACTTCAGTTGA TGGCACAACAAGTCCTATTT CTGAACATGACTACCAGATT GGTGGTTATACTGAAAAATG 26000

26001 GGAATCTGGAGTAAAAGACT GTGTTGTATTACACAGTTAC TTCAATTCAGACTATTACCA GCTGTACTCAACTCAATTGA GTACAGACACTGGTGTTGAA 26100

26101 CATGTTACCTTCTTCATCTA CAATAATATTGTTGATGAGC CTGAAGAACATGTCCAAATT CACACAATCGACGGTTCATC CGGAGTTGTTACTGCAGTAG 26200

26201 TAGCAGCAATTTATGATGAA CCGACGACGACTACTAGCGC GACTTTGTAAGCACAAGCTG ATGAGTGCTAACTTATGTAC TCATTCGTTTCGGAAGAGAC 26300

26301 AGGTACGTTAATAGTTAATA GCGTACTTCCTTTTCTTGCT TTCGTGGTATTTTAGCTAGT TACACTAGCCATCCTTACTG CGCTTCGATTGTGTGCGTAC 26400

26401 TGCTGCAATATTGTTAACGA GTGTCTTGTAAAACCTTCTT TTTACGTTTACTCTCGTGTT AAAAATCTGAATTCTTCTAG AGTTCCTGATCTTCTGGTCT 26500

26501 AAACGAACTAAATACTATAT TAGTTTTTCTGTTTGGAACT TTAATTTTAGCCATGGCAGA TTCCAACGGTACTATTAGCC TTGAAGAGTTAAAAAAGCTC 26600

26601 CGTCAACAATGGAACCTAGT AATAGGTTTCCTATTCCTTA CATGGATTTGTCTTCTACAA TTTGCCTATGCCAACAGGAA TGGTTTTTTGTATATAATTA 26700

26701 AGTTAATTTTCCTCTGGCTG TTATGGCCAGTAACTTTAGC TTGTTTTGTGCTTGCTGCTG TTTACAGAATAAATTGGATC AGCCGTGGAATTGCTATCGC 26800

26801 AATGGCTTGTCTTGTAGGCT TGATGTGGCTCAGCTACTTC ATTGCTTCTTTCAGACTGTT TGCGCGTACGTGATCCATGT GGTCATTCAATCCAGAAACT 26900

26901 AACATTCTTCTCAACGTGCC ACTCCATGGCACTATTCTGA CCAGCCAGCTTCTAGAAAGT GAACTCGTAATCGGAGCTGT GATCCTTCGTGGACATCTTC 27000

27001 GTATTGCTGGACACCATCTA GGACGCTGTGACATCAAGGA CCTGCCTAAAGAAATCACTG TTGCTACATCACGAACGCTT TCTTATTGCAAATTGGGAGC 27100

27101 TTCGCAGCGTGTAGCAGGTG ACTCAGGTTTTGCTGCATAC AGTCGCTACAGGATTGGCAA CTATAAATTAAACACAGACC ATCCTAGTAGCAGTGACAAT 27200

27201 ATTGCTTTGCTTGTACAGTA AGTGACAACAGATGTTTCAT CTCGTTGACTTTCAGGTTAC TATAGCAGAGATATTACTAA TTATTATGAGGACTTTTAAA 27300

27301 TTATCCATTTGGAATCTTGA TTACATCATAAACCTCATAA TTAAAAATTTATCTAAGTCA CTAACTGAGAATAAATATTC ACAATTAGATGAAGAGCAAC 27400

27401 CAATGGATAATGATTAAACG AACATGAAAATTATTCTTTT CTTGGCACTGATAACACTCG CTACTTGTGAGCTTTATCAC TACCAAGAGTGTGTTAGAGG 27500

27501 TACAACAGTACTTATTAAAG AACCTTGCTCTTCTGGAACA TACGAGGGCAATTCACCATT TCATCCTCTAGCTGATAACA AATTCGAACTGACTTGCTTT 27600

27601 AGCACTCAATTTGCTTTTGC TTGTCCTGACGGCGTAAAAC ACGTCTATCAGTTACGTGCC AGATCAGTCTTACCTAAACT GTTCATCAGACAAGAGGAAG 27700

27701 TTCAAGAACTTTACTCTCCA ATTTTTCTTATTGTTGCGGC AATAGTGTTTATAACCTATT GCTTCACACTCAAAAGAAAG ACAGAATGATTGAACTTTCA 27800

27801 TTAATTGACTTCTATTTGTG CTTTTTAGCCTTTCTGCTAT TCCTTGTTTTAATTACGATT ATTATCTTTTGGTTCTCACT TGAACTGCAAGATCATAATT 27900

27901 AAACTTGTCACGCCTAAACG AACATGAAATTTCTTGTTTT CTTAGGAATCATCACAACTG TAGCTGCATTTCACCAAGAA TGTAGTTTACAGTTATGTAC 28000

28001 TCAACATCAACCATATGTAG TTGATGACCCGTGTCCTATT CACTTCTATTCTAAATGGTA TATTAGAGTAGGAGCTAGAA AATCAGCACCTTTAATTGAA 28100

28101 TTGTGCGTGGATGAGGCTGG TTCTAAATCACCCATTCAGT ACATCGATATCGGTAATTAT ACAGTTTCCTGTTTACCTTT TACAATTAATTGCCAGGAAC 28200

28201 CTAAATTGGGTAGTCTTGTA GTGCGTTGTTCGTTCTATGA AGACTTTTTTAGAGTATCAT GACGTTCGTGTTGTTTTAGA TTTCATCTAAACGAACAAAC 28300

28301 TAAAATGTCTGATAATGCAC TCCAAAATCAGCGAAATGCA CCCCGCATTACGTTTGGTGG ACCCTCAGATTCAACTGGCA GTAACCAGAAAGGCGAACGC 28400

28401 AGTGGGGCGCGATCAAAACA ACGTCGGCCCCAAGGTTTAC CCAATAATACTGCGTCTTGG TTCACCGCTCTCACTCAACA TGGCAAGGAAGACCTTAAAT 28500

28501 TCTCTCGAGGACAAGGCGTT CCAATTAACACCAATAGCAG TCCAGATGACCAAATTGGCT ACTACCGAAGAGCTACCAGA CGAATTCGTGGTGGTGACGG 28600

28601 TAAAATGAATGAACTCAGTC CAAGATGGTATTTCTACTAC CTAGGAACTGGGCCAGAAGC TGGACTTCCCTATGGTGCTA ACAAAGACGGCATCATATGG 28700

28701 GTTGCAACTGAGGGAGCCTT GACTATACCAAAAGATCACA TTGGCACCCGCAATCCTGCT AACAATGCTGCAATCGTGCA ACTACTTCCTCAAGGAACAA 28800

28801 CATTGCCAAAAGGCTACTAC GCAGAAGGGAGCAGAGGCGG CAGTCAAGCCTCTTCTCGTT CCTCATCACGTAGTCGCAAC AGTTCAAGAAATTCAACTCT 28900

28901 AGGCAGCAGTAGGGGAACTT CTCCTGCTAGAATGGCTGGC AATGGCGGTGATGCTGCTCT TGCTTTGCTGCTGCTTAACT GATTGAACCAGCTTGAGAGC 29000

29001 AAAATGTCTGGTAAAGGCCA ACAACAACAATGCCAAACTG TCACTAAGAAATCTGCTGCT GAGGCTTCTAAGAAGCCTCG GCAAAAAACGTACTGCCACT 29100

29101 AAAGCATACAATGTAACACA AGCTTTCGGCAGACGTGGTC CAGAACAAACCCAAGGAAAT GTTGTGGACCAGGAACTAAT CAGACAAGGAACTGATTACA 29200

29201 ATCATAGGCCGCAAATTGCA CAATTTGCCCCCAGCGCTTC AGCGTTCTTCGGAATGTCGC GCATTGGCATGGAAGTCACA CCTTCGGGAACGTGGTTGAC 29300

29301 CTAGACTCCTGAGATCAAAA TGGTTGACAAAGATCCAAAA TTCTACGATTAAGTCATTTT GCTGAATAAACATTTTGACG CATACAAAACATTCCCACCA 29400

29401 ACAGAGCCTAAAAAGGACAA AAAGAAGAAGGCTGATGAAA CTCAAGCCTTACCGCAGAGA CGGAAAAAACAGCAAACTGT GACTCTTCTTCCTGCTGCAG 29500

29501 ATTTGGATGATTTCTCCAAA CAATTGCTACAATCCATGAG CAGTGCTGACTCAACTCAGG CCTAAACTCATGCAGACCAC ACAAGGCAGATGGGCTATAT 29600

29601 AAACGTTTTCGCCTTTCCGT TTACGATATATAGTCTACTC TTGTGCAGAATGAATTCTCG TAACTACATAGCACAAGTAG ATGTAGTTAACTTTAATCTC 29700

29701 ACATAGCAATATTTGATCAG TGTGTAACATTAGGGAGGAC TTGAAAGAGCCACCACATTT TCAGCGACGCCACGCGGAGT ACGATCGAGTGTACAGTGAA 29800

29801 CAATGCTAGGGAGAGCTGCC TATAAGGATGAGCCCTAATG TGTAAAATTAATTTTAGTAG TGCTATCCCCATGTGATTTT AATAGCTTCTTAGGAGAATG 29900

29901 ACAAAAAAAAAAATCACATGG GGATAGCAC 29929

1.15. SRR12596175 Discovered Strain 2 Nucleotide Sequence

1 AGAGAACAGATCTACAAGAG ATCGAAAGTTGGTTGGTTTG TTACCTGGGAAGGTATAAAC CAACCAACTTTCGATCTCTT GTAGATCTGTTCTCTAAACG 100

101 AACTTTAAAATCTGTGTGGC TGTCACTCGGCTGCATGCTT AGTGCACTCACGCAGTATAA TTAATAACTAATTACTGTCG TTGACAGGACACGAGTAACT 200

201 CGTCTATCTTCTGCAGGCTG CTTACGGTTTCGTCCGTGTT GCAGCCGATCATCAGCACAT CTAGGTTTTGTCCGGGTGTG ACCGAAAGGTAAGATGGAGA 300

301 GCCTTGTCCCTGGTTTCAAC GAGAAAACACACGTCCAACT CAGTTTGCCTGTTTTACAGG TTCGCGACGTGCTCGTACGT GGCTTTGGAGACTCCGTGGA 400

401 GGAGGTCTTATCAGAGGCAC GTCAACATCTTAAAGATGGC ACTTGTGGCTTAGTAGAAGT TGAAAAAGGCGTTTTGCCTC AACTTGAACAGCCCTATGTG 500

501 TTCATCAAACGTTCGGATGC TCGAACTGCACCTCATGGTC ATGTTATGGTTGAGCTGGTA GCAGAACTCGAAGGCATTCA GTACGGTCGTAGTGGTGAGA 600

601 CACTTGGTGTCCTTGTCCCT CATGTGGGCGAAATACCAGT GGCTTACCGCAAGGTTCTTC TTCGTAAGAACGGTAATAAA GGAGCTGGTGGCCATAGTTA 700

701 CGGCGCCGATCTAAAGTCAT TTGACTTAGGCGACGAGCTT GGCACTGATCCTTATGAAGA TTTTCAAGAAAACTGGAACA CTAAACATAGCAGTGGTGTT 800

801 ACCCGTGAACTCATGCGTGA GCTTAACGGAGGGGCATACA CTCGCTATGTCGATAACAAC TTCTGTGGCCCTGATGGCTA CCCTCTTGAGTGCATTAAAG 900

901 ACCTTCTAGCACGTGCTGGT AAAGCTTCATGCACTTTGTC CGAACAACTGGACTTTATTG ACACTAAGAGGGGTGTATAC TGCTGCCGTGAACATGAGCA 1000

1001 TGAAATTGCTTGGTACACGG AACGTTCTGAAAAGAGCTAT GAATTGCAGACACCTTTTGA AATTAAATTGGCAAAGAAAT TTGACATCTTCAATGGGGAA 1100

1101 TGTCCAAATTTTGTATTTCC CTTAAATTCCATAATCAAGA CTATTCAACCAAGGGTTGAA AAGAAAAAGCTTGATGGCTT TATGGGTAGAATTCGATCTG 1200

1201 TCTATCCAGTTGCGTCACCA AATGAATGCAACCAAATGTG CCTTTCAACTCTCATGAAGT GTGATCATTGTGGTGAAACT TCATGGCAGACGGGCGATTT 1300

1301 TGTTAAAGCCACTTGCGAAT TTTGTGGCACTGAGAATTTG ACTAAAGAAGGTGCCACTAC TTGTGGTTACTTACCCCAAA ATGCTGTTGTTAAAATTTAT 1400

1401 TGTCCAGCATGTTACAATTC AGAAGTAGGACCTGAGCATA GTCTTGCCGAATACCATAAT GAATCTGGCTTGAAAACCAT TCTTCGTAAGGGTGGTCGCA 1500

1501 CTATTGCCTTTGGAGGCTGT GTGTTCTCTTATGTTGGTTG CCATAACAAGTGTGCCTTTT GGGTTCCACGTGCTAGCGCT AACATAGGTTGTAACCATAC 1600

1601 AGGTGTTGTTGGAGAAGGTT CCGAAGGTCTTAATGACAAC CTTCTTGAAATACTCCAAAA AGAGAAAGTCAACATCAATA TTGTTGGTGACTTTAAACTT 1700

1701 AATGAAGAGATCGCCATTAT TTTGGCATCTTTTTCTGCTT CCACAAGTGCTTTTGTGGAA ACTGTTAAAGGTTTGGATTA TAAAGCATTCAAACAAATTG 1800

1801 TTGAATCCTGTGGTAATTTT AAAGTTACAAAAGGAAAAGC TAAAAAAGGTGCCTGGAATA TTGGTGAACAGAAATCAATA CTGAGTCCTCTTTATGCATT 1900

1901 TGCATCAGAGGCTGCTCGTG TTGTACGATCAATTTTCTCC CGCACTCTTGAAACTGCTCA AAATTCTGTGCGTGTTTTAC AGAAGGCCGCTATAACAATA 2000

2001 CTAGATGGAATTTCACAGTA TTCACTGAGACTCATTGATG CTATGATGTTCACATCTGAT TTGGCTACTAACAATCTAGT TGTAATGGCCTACATTACAG 2100

2101 GTGGTGTTGTTCAGTTGACT TCGCAGTGGCTAACTAACAT CTTTGGCACTGTTTATGAAA AACTCAAACCCGTCCTTGAT TGGCTTGAAGAGAAGTTTAA 2200

2201 GGAAGGTGTAGAGTTTCTTA GAGACGGTTGGGAAATTGTT AAATTTATCTCAACCTGTGC TTGTGAAATTGTCGGTGGAC AAATTGTCACCTGTGCAAAG 2300

2301 GAAATTAAGGAGAGTGTTCA GACATTCTTTAAGCTTGTAA ATAAATTTTTGGCTTTGTGT GCTGACTCTATCATTATTGG TGGAGCTAAACTTAAAGCCT 2400

2401 TGAATTTAGGTGAAACATTT GTCACGCACTCAAAGGGATT GTACAGAAAGTGTGTTAAAT CCAGAGAAGAAACTGGCCTA CTCATGCCTCTAAAAGCCCC 2500

2501 AAAAGAAATTATCTTCTTAG AGGGAGAAACACTTCCCACA GAAGTGTTAACAGAGGAAGT TGTCTTGAAAACTGGTGATT TACAACCATTAGAACAACCT 2600

2601 ACTAGTGAAGCTGTTGAAGC TCCATTGGTTGGTACACCAG TTTGTATTAACGGGCTTATG TTGCTCGAAATCAAAGACAC AGAAAAGTACTGTGCCCTTG 2700

2701 CACCTAATATGATGGTAACA AACAATACCTTCACACTCAA AGGCGGTGCACCAACAAAGG TTACTTTTGGTGATGACACT GTGATAGAAGTGCAAGGTTA 2800

2801 CAAGAGTGTGAATATCACTT TTGAACTTGATGAAAGGATT GATAAAGTACTTAATGAGAA GTGCTCTGCCTATACAGTTG AACTCGGTACAGAAGTAAAT 2900

2901 GAGTTCGCCTGTGTTGTGGC AGATGCTGTCATAAAAACTT TGCAACCAGTATCTGAATTA CTTACACCACTGGGCATTGA TTTAGATGAGTGGAGTATGG 3000

3001 CTACATACTACTTATTTGAT GAGTCTGGTGAGTTTAAATT GGCTTCACATATGTATTGTT CTTTTTACCCTCCAGATGAG GATGAAGAAGAAGGTGATTG 3100

3101 TGAAGAAGAAGAGTTTGAGC CATCAACTCAATATGAGTAT GGTACTGAAGATGATTACCA AGGTAAACCTTTGGAATTTG GTGCCACTTCTGCTGCTCTT 3200

3201 CAACCTGAAGAAGAGCAAGA AGAAGATTGGTTAGATGATG ATAGTCAACAAACTGTTGGT CAACAAGACAGCAGTGAGGA CAATCAGACAACTACTATTC 3300

3301 AAACAATTGTTGAGGTTCAA CCTCAATTAGAGATGGAACT TACACCAGTTGTTCAGACTA TTGAAGTGAATAGTTTTAGT GGTTATTTAAAACTTACTGA 3400

3401 CAATGTATACATTAAAAATG CAGACATTGTGGAAGAAGCT AAAAAGGTAAAACCAACAGT GGTTGTTAATGCAGCCAATG TTTACCTTAAACATGGAGGA 3500

3501 GGTGTTGCAGGAGCCTTAAA TAAGGCTACTAACAATGCCA TGCAAGTTGAATCTGATGAT TACATAGCTACTAATGGACC ACTTAAAGTGGGTGGTAGTT 3600

3601 GTGTTTTAAGCGGACACAAT CTTGCTAAACACTGTCTTCA TGTTGTCGGCCCAAATGTTA ACAAAGGTGAAGACATTCAA CTTCTTAAGAGTGCTTATGA 3700

3701 AAATTTTAATCAGCACGAAG TTCTACTTGCACCATTATTA TCAGCTGGTATTTTTGGTGC TGACCCTATACATTCTTTAA GAGTTTGTGTAGATACTGTT 3800

3801 CGCACAAATGTCTACTTAGC TGTCTTTGATAAAAATCTCT ATGACAAACTTGTTTCAAGC TTTTTGGAAATGAAGAGTGA AAAGCAAGTTGAACAAAAGA 3900

3901 TCGCTGAGATTCCTAAAGAG GAAGTTAAGCCATTTATAAC TGAAAGTAAACCTTCAGTTG AACAGAGAAAACAAGATGAT AAGAAAATCAAAGCTTGTGT 4000

4001 TGAAGAAGTTACAACAACTC TGGAAGAAACTAAGTTCCTC ACAGAAAACTTGTTACTTTA TATTGACATTAATGGCAATC TTCATCCAGATTCTGCCACT 4100

4101 CTTGTTAGTGACATTGACAT CACTTTCTTAAAGAAAGATG CTCCATATATAGTGGGTGAT GTTGTTCAAGAGGGTGTTTT AACTGCTGTGGTTATACCTA 4200

4201 CTAAAAAGGCTGGTGGCACT ACTGAAATGCTAGCGAAAGC TTTGAGAAAAGTGCCAACAG ACAATTATATAACCACTTAC CCGGGTCAGGGTTTAAATGG 4300

4301 TTACACTGTAGAGGAGGCAA AGACAGTGCTTAAAAAGTGT AAAAGTGCCTTTTACATTCT ACCATCTATTATCTCTAATG AGAAGCAAGAAATTCTTGGA 4400

4401 ACTGTTTCTTGGAATTTGCG AGAAATGCTTGCACATGCAG AAGAAACACGCAAATTAATG CCTGTCTGTGTGGAAACTAA AGCCATAGTTTCAACTATAC 4500

4501 AGCGTAAATATAAGGGTATT AAAATACAAGAGGGTGTGGT TGATTATGGTGCTAGATTTT ACTTTTACACCAGTAAAACA ACTGTAGCGTCACTTATCAA 4600

4601 CACACTTAACGATCTAAATG AAACTCTTGTTACAATGCCA CTTGGCTATGTAACACATGG CTTAAATTTGGAAGAAGCTG CTCGGTATATGAGATCTCTC 4700

4701 AAAGTGCCAGCTACAGTTTC TGTTTCTTCACCTGATGCTG TTACAGCGTATAATGGTTAT CTTACTTCTTCTTCTAAAAC ACCTGAAGAACATTTTATTG 4800

4801 AAACCATCTCACTTGCTGGT TCCTATAAAGATTGGTCCTA TTCTGGACAATCTACACAAC TAGGTATAGAATTTCTTAAG AGAGGTGATAAAAGTGTATA 4900

4901 TTACACTAGTAATCCTACCA CATTCCACCTAGATGGTGAA GTTATCACCTTTGACAATCT TAAGACACTTCTTTCTTTGA GAGAAGTGAGGACTATTAAG 5000

5001 GTGTTTACAACAGTAGACAA CATTAACCTCCACACGCAAG TTGTGGACATGTCAATGACA TATGGACAACAGTTTGGTCC AACTTATTTGGATGGAGCTG 5100

5101 ATGTTACTAAAATAAAACCT CATAATTCACATGAAGGTAA AACATTTTATGTTTTACCTA ATGATGACACTCTACGTGTT GAGGCTTTTGAGTACTACCA 5200

5201 CACAACTGATCCTAGTTTTC TGGGTAGGTACATGTCAGCA TTAAATCACACTAAAAAGTG GAAATACCCACAAGTTAATG GTTTAACTTCTATTAAATGG 5300

5301 GCAGATAACAACTGTTATCT TGCCACTGCATTGTTAACAC TCCAACAAATAGAGTTGAAG TTTAATCCACCTGCTCTACA AGATGCTTATTACAGAGCAA 5400

5401 GGGCTGGTGAAGCTGCTAAC TTTTGTGCACTTATCTTAGC CTACTGTAATAAGACAGTAG GTGAGTTAGGTGATGTTAGA GAAACAATGAGTTACTTGTT 5500

5501 TCAACATGCCAATTTAGATT CTTGCAAAAGAGTCTTGAAC GTGGTGTGTAAAACTTGTGG ACAACAGCAGACAACCCTTA AGGGTGTAGAAGCTGTTGTG 5600

5601 TACATGGGCACACTTTCTTA TGAACAATTTAAGAAAGGTG TTCAGATACCTTGTACGTGT GGTAAACAAGCTACAAAATA TCTAGTACAACAGGAGTCAC 5700

5701 CTTTTGTTATGATGTCAGCA CCACCTGCTCAGTATGAACT TAAGCATGGTACATTTACTT GTGCTAGTGAGTACACTGGT AATTACCAGTGTGGTCACTA 5800

5801 TAAACATATAACTTCTAAAG AAACTTTGTATTGCATAGAC GGTGCTTTACTTACAAAGTC CTCAGAATACAAAGGTCCTA TTACGGATGTTTTCTACAAA 5900

5901 GAAAACAGTTACACAACAAC CATAAAACCAGTTACTTATA AATTGGATGGTGTTGTTTGT ACAGAAATTGACCCTAAGTT GGACAATTATTATAAGAAAG 6000

6001 ACAATTCTTATTTCACAGAG CAACCAATTGATCTTGTACC AAACCAACCATATCCAAACG CAAGCTTCGATAATTTTAAG TTTGTATGTGATAATATCAA 6100

6101 ATTTGCTGATGATTTAAACC AGTTAACTGGTTATAAGAAA CCTGCTTCAAGAGAGCTTAA AGTTACATTTTTCCCTGACT TAAATGGTGATGTGGTGGCT 6200

6201 ATTGATTATAAACACTACAC ACCCTCTTTTAAGAAAGGAG CTAAATTGTTACATAAACCT ATTGTTTGGCATGTTAACAA TGCAACTAATAAAGCCACGT 6300

6301 ATAAACCAAATACCTGGTGT ATACGTTGTCTTTGGAGCAC AAAACCAGTTGAAACATCAA ATTCGTTTGATGTACTGAAG TCAGAGGACGCGCAGGGAAT 6400

6401 GGATAATCTTGCCTGCGAAG ATCTAAAACCAGTCTCTGAA GAAGTAGTGGAAAATCCTAC CATACAGAAAGACGTTCTTG AGTGTAATGTGAAAACTACC 6500

6501 GAAGTTGTAGGAGACATTAT ACTTAAACCAGCAAATAATA GTTTAAAAATTACAGAAGAG GTTGGCCACACAGATCTAAT GGCTGCTTATGTAGACAATT 6600

6601 CTAGTCTTACTATTAAGAAA CCTAATGAATTATCTAGAGT ATTAGGTTTGAAAACCCTTG CTACTCATGGTTTAGCTGCT GTTAATAGTGTCCCTTGGGA 6700

6701 TACTATAGCTAATTATGCTA AGCCTTTTCTTAACAAAGTT GTTAGTACAACTACTAACAT AGTTACACGGTGTTTAAACC GTGTTTGTACTAATTATATG 6800

6801 CCTTATTTCTTTACTTTATT GCTACAATTGTGTACTTTTA CTAGAAGTACAAATTCTAGA ATTAAAGCATCTATGCCGAC TACTATAGCAAAGAATACTG 6900

6901 TTAAGAGTGTCGGTAAATTT TGTCTAGAGGCTTCATTTAA TTATTTGAAGTCACCTAATT TTTCTAAACTGATAAATATT ATAATTTGGTTTTTACTATT 7000

7001 AAGTGTTTGCCTAGGTTCTT TAATCTACTCAACCGCTGCT TTAGGTGTTTTAATGTCTAA TTTAGGCATGCCTTCTTACT GTACTGGTTACAGAGAAGGC 7100

7101 TATTTGAACTCTACTAATGT CACTATTGCAACCTACTGTA CTGGTTCTATACCTTGTAGT GTTTGTCTTAGTGGTTTAGA TTCTTTAGACACCTATCCTT 7200

7201 CTTTAGAAACTATACAAATT ACCATTTCATCTTTTAAATG GGATTTAACTGCTTTTGGCT TAGTTGCAGAGTGGTTTTTG GCATATATTCTTTTCACTAG 7300

7301 GTTTTTCTATGTACTTGGAT TGGCTGCAATCATGCAATTG TTTTTCAGCTATTTTGCAGT ACATTTTATTAGTAATTCTT GGCTTATGTGGTTAATAATT 7400

7401 AATCTTGTACAAATGGCCCC GATTTCAGCTATGGTTAGAA TGTACATCTTCTTTGCATCA TTTTATTATGCATGGAAAAG TTATGTGCATGTTGTAGACG 7500

7501 GTTGTAATTCATCAACTTGT ATGATGTGTTACAAACGTAA TAGAGCAACAAGAGTCGAAT GTACAACTATTGTTAATGGT GTTAGAAGGTCCTTTTATGT 7600

7601 CTATGCTAATGGAGGTAAAG GCTTTTGCAAACTACACAAT TGGAATTGTGTTAATTGTGA TACATTCTGTGCTGGTAGTA CATTTATTAGTGATGAAGTT 7700

7701 GCGAGAGACTTGTCACTACA GTTTAAAAGACCAATAAATC CTACTGACCAGTCTTCTTAC ATCGTTGATAGTGTTACAGT GAAGAATGGTTCCATCCATC 7800

7801 TTTACTTTGATAAAGCTGGT CAAAAGACTTATGAAAGACA TTCTCTCTCTCATTTTGTTA ACTTAGACAACCTGAGAGCT AATAACACTAAAGGTTCATT 7900

7901 GCCTATTAATGTTATAGTTT TTGATGGTAAATCAAAATGT GAAGAATCATCTGCAAAATC AGCGTCTGTTTACTACAGTC AGCTTATGTGTCAACCTATA 8000

8001 CTGTTACTAGATCAGGCATT AGTGTCTGATGTTGGTGATA GTGCGGAAGTTGCAGTTAAA ATGTTTGATGCTTACGTTAA TACGTTTTCATCAACTTTTA 8100

8101 ACGTACCAATGGAAAAACTC AAAACACTAGTTGCAACTGC AGAAGCTGAACTTGCAAAGA ATGTGTCCTTAGACAATGTC TTATCTACTTTTATTTCAGC 8200

8201 AGCTCGGCAAGGGTTTGTTG ATTCAGATGTAGAAACTAAA GATGTTGTTGAATGTCTTAA ATTGTCACATCAATCTGACA TAGAAGTTACTGGCGATAGT 8300

8301 TGTAATAACTATATGCTCAC CTATAACAAAGTTGAAAACA TGACACCCCGTGACCTTGGT GCTTGTATTGACTGTAGTGC GCGTCATATTAATGCGCAGG 8400

8401 TAGCAAAAAGTCACAACATT GCTTTGATATGGAACGTTAA AGATTTCATGTCATTGTCTG AACAACTACGAAAACAAATA CGTAGTGCTGCTAAAAAGAA 8500

8501 TAACTTACCTTTTAAGTTGA CATGTGCAACTACTAGACAA GTTGTTAATGTTGTAACAAC AAAGATAGCACTTAAGGGTG GTAAAATTGTTAATAATTGG 8600

8601 TTGAAGCAGTTAATTAAAGT TACACTTGTGTTCCTTTTTG TTGCTGCTATTTTCTATTTA ATAACACCTGTTCATGTCAT GTCTAAACATACTGACTTTT 8700

8701 CAAGTGAAATCATAGGATAC AAGGCTATTGATGGTGGTGT CACTCGTGACATAGCATCTA CAGATACTTGTTTTGCTAAC AAACATGCTGATTTTGACAC 8800

8801 ATGGTTTAGCCAGCGTGGTG GTAGTTATACTAATGACAAA GCTTGCCCATTGATTGCTGC AGTCATAACAAGAGAAGTGG GTTTTGTCGTGCCTGGTTTG 8900

8901 CCTGGCACGATATTACGCAC AACTAATGGTGACTTTTTGC ATTTCTTACCTAGAGTTTTT AGTGCAGTTGGTAACATCTG TTACACACCATCAAAACTTA 9000

9001 TAGAGTACACTGACTTTGCA ACATCAGCTTGTGTTTTGGC TGCTGAATGTACAATTTTTA AAGATGCTTCTGGTAAGCCA GTACCATATTGTTATGATAC 9100

9101 CAATGTACTAGAAGGTTCTG TTGCTTATGAAAGTTTACGC CCTGACACACGTTATGTGCT CATGGATGGCTCTATTATTC AATTTCCTAACACCTACCTT 9200

9201 GAAGGTTCTGTTAGAGTGGT AACAACTTTTGATTCTGAGT ACTGTAGGCACGGCACTTGT GAAAGATCAGAAGCTGGTGT TTGTGTATCTACTAGTGGTA 9300

9301 GATGGGTACTTAACAATGAT TATTACAGATCTTTACCAGG AGTTTTCTGTGGTGTAGATG CTGTAAATTTACTTACTAAT ATGTTTACACCACTAATTCA 9400

9401 ACCTATTGGTGCTTTGGACA TATCAGCATCTATAGTAGCT GGTGGTATTGTAGCTATCGT AGTAACATGCCTTGCCTACT ATTTTATGAGGTTTAGAAGA 9500

9501 GCTTTTGGTGAATACAGTCA TGTAGTTGCCTTTAATACTT TACTATTCCTTATGTCATTC ACTGTACTCTGTTTAACACC AGTTTACTCATTCTTACCTG 9600

9601 GTGTTTATTCTGTTATTTAC TTGTACTTGACATTTTATCT TACTAATGATGTTTCTTTTT TAGCACATATTCAGTGGATG GTTATGTTCACACCTTTAGT 9700

9701 ACCTTTCTGGATAACAATTG CTTATATCATTTGTATTTCC ACAAAGCATTTCTATTGGTT CTTTAGTAATTACCTAAAGA GACGTGTAGTCTTTAATGGT 9800

9801 GTTTCCTTTAGTACTTTTGA AGAAGCTGCGCTGTGCACCT TTTTGTTAAATAAAGAAATG TATCTAAAGTTGCGTAGTGA TGTGCTATTACCTCTTACGC 9900

9901 AATATAATAGATACTTAGCT CTTTATAATAAGTACAAGTA TTTTAGTGGAGCAATGGATA CAACTAGCTACAGAGAAGCT GCTTGTTGTCATCTCGCAAA 10000

10001 GGCTCTCAATGACTTCAGTA ACTCAGGTTCTGATGTTCTT TACCAACCACCACAAACCTC TATCACCTCAGCTGTTTTGC AGAGTGGTTTTAGAAAAATG 10100

10101 GCATTCCCATCTGGTAAAGT TGAGGGTTGTATGGTACAAG TAACTTGTGGTACAACTACA CTTAACGGTCTTTGGCTTGA TGACGTAGTTTACTGTCCAA 10200

10201 GACATGTGATCTGCACCTCT GAAGACATGCTTAACCCTAA TTATGAAGATTTACTCATTC GTAAGTCTAATCATAATTTC TTGGTACAGGCTGGTAATGT 10300

10301 TCAACTCAGGGTTATTGGAC ATTCTATGCAAAATTGTGTA CTTAAGCTTAAGGTTGATAC AGCCAATCCTAAGACACCTA AGTATAAGTTTGTTCGCATT 10400

10401 CAACCAGGACAGACTTTTTC AGTGTTAGCTTGTTACAATG GTTCACCATCTGGTGTTTAC CAATGTGCTATGAGGCCCAA TTTCACTATTAAGGGTTCAT 10500

10501 TCCTTAATGGTTCATGTGGT AGTGTTGGTTTTAACATAGA TTATGACTGTGTCTCTTTTT GTTACATGCACCATATGGAA TTACCAACTGGAGTTCATGC 10600

10601 TGGCACAGACTTAGAAGGTA ACTTTTATGGACCTTTTGTT GACAGGCAAACAGCACAAGC AGCTGGTACGGACACAACTA TTACAGTTAATGTTTTAGCT 10700

10701 TGGTTGTACGCTGCTGTTAT AAAAGGAGACAGGTGGTTTC TCAATCGATTTACCACAACT CTTAATGACTTTAACCTTGT GGCTATGAAGTACAATTATG 10800

10801 AACCTCTAACACAAGACCAT GTTGACATACTAGGACCTCT TTCTGCTCAAACTGGAATTG CCGTTTTAGATATGTGTGCT TCATTAAAAGAATTACTGCA 10900

10901 AAATGGTATGAATGGACGTA CCATATTGGGTAGTGCTTTA TTAGAAGATGAATTTACACC TTTTGATGTTGTTAGACAAT GCTCAGGTGTTACTTTCCAA 11000

11001 AGTGCAGTGAAAAGAACAAT CAAGGGTACACACCACTGGT TGTTACTCACAATTTTGACT TCACTTTTAGTTTTAGTCCA GAGTACTCAATGGTCTTTGT 11100

11101 TCTTTTTTTTGTATGAAAAT GCCTTTTTACCTTTTGCTAT GGGTATTATTGCTATGTCTG CTTTTGCAATGATGTTTGTC AAACATAAGCATGCATTTCT 11200

11201 CTGTTTGTTTTTGTTACCTT CTCTTGCCACTGTAGCTTAT TTTAATATGGTCTATATGCC TGCTAGTTGGGTGATGCGTA TTATGACATGGTTGGATATG 11300

11301 GTTGATACTAGTTTGTCTGG TTTTAAGCTAAAAGACTGTG TTATGTATGCATCAGCTGTA GTGTTACTAATCCTTATGAC AGCAAGAACTGTGTATGATG 11400

11401 ATGGTGCTAGGAGAGTGTGG ACACTTATGAATGTCTTGAC ACTCGTTTATAAAGTTTATT ATGGTAATGCTTTAGATCAA GCCATTTCCATGTGGGCTCT 11500

11501 TATAATCTCTGTTACTTCTA ACTACTCAGGTGTAGTTACA ACTGTCATGTTTTTGGCCAG AGGTATTGTTTTTATGTGTG TTGAGTATTGCCCTATTTTC 11600

11601 TTCATAACTGGTAATACACT TCAGTGTATAATGCTAGTTT ATTGTTTCTTAGGCTATTTT TGTACTTGTTACTTTGGCCT CTTTTGTTTACTCAACCGCT 11700

11701 ACTTTAGACTGACTCTTGGT GTTTATGATTACTTAGTTTC TACACAGGAGTTTAGATATA TGAATTCACAGGGACTACTC CCACCCAAGAATAGCATAGA 11800

11801 TGCCTTCAAACTCAACATTA AATTGTTGGGTGTTGGTGGC AAACCTTGTATCAAAGTAGC CACTGTACAGTCTAAAATGT CAGATGTAAAGTGCACATCA 11900

11901 GTAGTCTTACTCTCAGTTTT GCAACAACTCAGAGTAGAAT CATTATCTAAATTGTGGGCT CAATGTGTCCAGTTACACAA TGACATTCTCTTAGCTAAAG 12000

12001 ATACTACTGAAGCCTTTGAA AAAATGGTTTCACTACTTTC TGTTTTGCTTTCCATGCAGG GTGCTGTAGACATAAACAAG CTTTGTGAAGAAATGCTGGA 12100

12101 CAACAGGGCAACCTTACAAG CTATAGCCTCAGAGTTTAGT TCCCTTCCATCATATGCAGC TTTTGCTACTGCTCAAGAAG CTTATGAGCAGGCTGTTGCT 12200

12201 AATGGTGATTCTGAAGTTGT TCTTAAAAAGTTGAAGAAGT CTTTGAATGTGGCTAAATCT GAATTTGACCGTGATGCAGC CATGCAACGTAAGTTGGAAA 12300

12301 AGATGGCTGATCAAGCTATG ACCCAAATGTATAAACAGGC TAGATCTGAGGACAAGAGGG CAAAAGTTACTAGTGCTATG CAGACAATGCTTTTCACTAT 12400

12401 GCTTAGAAAGTTGGATAATG ATGCACTCAACAACATTATC AACAATGCAAGAGATGGTTG TGTTCCCTTGAACATAATAC CTCTTACAACAGCAGCCAAA 12500

12501 CTAATGGTTGTCATACCAGA CTATAACACATATAAAAATA CGTGTGATGGTACAACATTT ACTTATGCATCAGCATTGTG GGAAATCCAACAGGTTGTAG 12600

12601 ATGCAGATAGTAAAATTGTT CAACTTAGTGAAATTAGTAT GGACAATTCACCTAATTTAG CATGGCCTCTTATTGTAACA GCTTTAAGGGCCAATTCTGC 12700

12701 TGTCAAATTACAGAATAATG AGCTTAGTCCTGTTGCACTA CGACAGATGTCTTGTGCTGC CGGTACTACACAAACTGCTT GCACTGATGACAATGCGTTA 12800

12801 GCTTACTACAACACAACAAA GGGAGGTAGGTTTGTACTTG CACTGTTATCCGATTTACAG GATTTGAAATGGGCTAGATT CCCTAAGAGTGATGGAACTG 12900

12901 GTACTATCTATACAGAACTG GAACCACCTTGTAGGTTTGT TACAGACACACCTAAAGGTC CTAAAGTGAAGTATTTATAC TTTATTAAAGGATTAAACAA 13000

13001 CCTAAATAGAGGTATGGTAC TTGGTAGTTTAGCTGCCACA GTACGTCTACAAGCTGGTAA TGCAACAGAAGTGCCTGCCA ATTCAACTGTATTATCTTTC 13100

13101 TGTGCTTTTGCTGTAGATGC TGCTAAAGCTTACAAAGATT ATCTAGCTAGTGGGGGACAA CCAATCACTAATTGTGTTAA GATGTTGTGTACACACACTG 13200

13201 GTACTGGTCAGGCAATAACA GTTACACCGGAAGCCAATAT GGATCAAGAATCCTTTGGTG GTGCATCGTGTTGTCTGTAC TGCCGTTGCCACATAGATCA 13300

13301 TCCAAATCCTAAAGGATTTT GTGACTTAAAAGGTAAGTAT GTACAAATACCTACAACTTG TGCTAATGACCCTGTGGTTT TTACACTTAAAAACACAGTC 13400

13401 TGTACCGTCTGCGGTATGTG GAAAGGTTATGGCTGTAGTT GTGATCAACTCCGCGAACCC ATGCTTCAGTCAGCTGATGC ACAATCGTTTTTAAACGGGT 13500

13501 TTGCGGTGTAAGTGCAGCCC GTCTTACACCGTGCGGCACA GGCACTAGTACTGATGTCGT ATACAGGGCTTTTGACATCT ACAATGATAAAGTAGCTGGT 13600

13601 TTTGCTAAATTCCTAAAAAC TAATTGTTGTCGCTTCCAAG AAAAGGACGAAGATGACAAT TTAATTGATTCTTACTTTGT AGTTAAGAGACACACTTTCT 13700

13701 CTAACTACCAACATGAAGAA ACAATTTATAATTTACTTAA GGATTGTCCAGCTGTTGCTA AACATGACTTCTTTAAGTTT AGAATAGACGGTGACATGGT 13800

13801 ACCACATATATCACGTCAAC GTCTTACTAAATACACAATG GCAGACCTCGTCTATGCTTT AAGGCATTTTGATGAAGGTA ATTGTGACACATTAAAAGAA 13900

13901 ATACTTGTCACATACAATTG TTGTGATGATGATTATTTCA ATAAAAAGGACTGGTATGAT TTTGTAGAAAACCCAGATAT ATTACGCGTATACGCCAACT 14000

14001 TAGGTGAACGTGTACGCCAA GCTTTGTTAAAAACAGTACA ATTCTGTGATGCCATGCGAA ATGCTGGTATTGTTGGTGTA CTGACATTAGATAATCAAGA 14100

14101 TCTCAATGGTAACTGGTATG ATTTCGGTGATTTCATACAA ACCACGCCAGGTAGTGGAGT TCCTGTTGTAGATTCTTATT ATTCATTGTTAATGCCTATA 14200

14201 TTAACCTTGACCAGGGCTTT AACTGCAGAGTCACATGTTG ACACTGACTTAACAAAGCCT TACATTAAGTGGGATTTGTT AAAATATGACTTCACGGAAG 14300

14301 AGAGGTTAAAACTCTTTGAC CGTTATTTTAAATATTGGGA TCAGACATACCACCCAAATT GTGTTAACTGTTTGGATGAC AGATGCATTCTGCATTGTGC 14400

14401 AAACTTTAATGTTTTATTCT CTACAGTGTTCCCACTTACA AGTTTTGGACCACTAGTGAG AAAAATATTTGTTGATGGTG TTCCATTTGTAGTTTCAACT 14500

14501 GGATACCACTTCAGAGAGCT AGGTGTTGTACATAATCAGG ATGTAAACTTACATAGCTCT AGACTTAGTTTTAAGGAATT ACTTGTGTATGCTGCTGACC 14600

14601 CTGCTATGCACGCTGCTTCT GGTAATCTATTACTAGATAA ACGCACTACGTGCTTTTCAG TAGCTGCACTTACTAACAAT GTTGCTTTTCAAACTGTCAA 14700

14701 ACCCGGTAATTTTAACAAAG ACTTCTATGACTTTGCTGTG TCTAAGGGTTTCTTTAAGGA AGGAAGTTCTGTTGAATTAA AACACTTCTTCTTTGCTCAG 14800

14801 GATGGTAATGCTGCTATCAG CGATTATGACTACTATCGTT ATAATCTACCAACAATGTGT GATATCAGACAACTACTATT TGTAGTTGAAGTTGTTGATA 14900

14901 AGTACTTTGATTGTTACGAT GGTGGCTGTATTAATGCTAA CCAAGTCATCGTCAACAACC TAGACAAATCAGCTGGTTTT CCATTTAATAAATGGGGTAA 15000

15001 GGCTAGACTTTATTATGATT CAATGAGTTATGAGGATCAA GATGCACTTTTCGCATATAC AAAACGTAATGTCATCCCTA CTATAACTCAAATGAATCAT 15100

15101 AAGTATGCCATTAGTGCAAA GAATAGAGCTCGCACCGTAG CTGGTGTCTCTATCTGTAGT ACTATGACCAATAGACAGTT TCATCAAAAATTATTGAAAT 15200

15201 CAATAGCCGCCACTAGAGGA GCTACTGTAGTAATTGGAAC AAGCAAATTCTATGGTGGTT GGCACAACATGTTAAAAACT GTTTATAGTGATGTAGAAAA 15300

15301 CCCTCACCTTATGGGTTGGG ATTATCCTAAATGTGATAGA GCCATGCCTTACATGCTTAG AATTATGGCCTCACTTGTTC TTGCTCGCAAACATACAACG 15400

15401 TGTTGTAGCTTGTCACACCG TTTCTATAGATTAGCTAATG AGTGTGCTCAAGTATTGAGT GAAATGGTCATGTGTGGCGG TTCACTATATGTTAAACCAG 15500

15501 GTGGAACCTCATCAGGAGAT GCCACAACTGCTTATGCTAA TAGTGTTTTTAACATTTGTC AAGCTGTCACGGCCAATGTT AATGCACTTTTATCTACTGA 15600

15601 TGGTAACAAAATTGCCGATA AGTATGTCCGCAATTTACAA CACAGACTTTATGAGTGTCT CTATAGAAATAGAGATGTTG ACACAGACTTTGTGAATGAG 15700

15701 TTTTACGCATATTTGCGTAA ACATTTCTCAATGATGATAC TCTCTGACGATGCTGTTGTG TGTTTCAATAGCACTTATGC ATCTCAAGGTCTAGTGGCTA 15800

15801 GCATAAAGAACTTTAAGTCA GTTCTTTATTATCAAAACAA TGTTTTTATGTCTGTAGCAA AATGTTGGACTGAGACTGAC CTTACTAAAGGACCTCATGA 15900

15901 ATTTTGCTCTCAACATACAA TGCTAGTTAAACAGGGTGAT GATTATGTGTACCTTCCTTA CCCAGATCCATCAAGAATCC TAGGGGCCGGCTGTTTTGTA 16000

16001 GATGATATCGTAAAAACAGA TGGTACACTTATGATTGAAC GGTTCGTGTCTTTAGCTATA GATGCTTACCCACTTACTAA ACATCCTAATCAGGAGTATG 16100

16101 CTGATGTCTTTCATTTGTAC TTACAATACATAAGAAAGCT ACATGATGAGTTAACAGGAC ACATGTTAGACATGTATTCT GTTATGCTTACTAATGATAA 16200

16201 CACTTCAAGGTATTGGGAAC CTGAGTTTTATGAGGCTATG TACACACCGCATACAGTCTT ACAGGCTGTTGGGGCTTGTG TTCTTTGCAATTCACAGACT 16300

16301 TCATTAAGATGTGGTGCTTG CATACGTAGACCATTCTTAT GTTGTAAATGCTGTTACGAC CATGTCATATCAACATCACA TAAATTAGTCTTGTCTGTTA 16400

16401 ATCCGTATGTTTGCAATGCT CCAGGTTGTGATGTCACAGA TGTGACTCAACTTTACTTAG GAGGTATGAGCTATTATTGT AAATCACATAAACCACCCAT 16500

16501 TAGTTTTCCATTGTGTGCTA ATGGACAAGTTTTTGGTTTA TATAAAAATACATGTGTTGG TAGCGATAATGTTACTGACT TTAATGCAATTGCAACATGT 16600

16601 GACTGGACAAATGCTGGTGA TTACATTTTAGCTAACACCT GTACTGAAAGACTCAAGCTT TTTGCAGCAGAAACGCTCAA AGCTACTGAGGAGACATTTA 16700

16701 AACTGTCTTATGGTATTGCT ACTGTACGTGAAGTGCTGTC TGACAGAGAATTACATCTTT CATGGGAAGTTGGTAAACCT AGACCACCACTTAACCGAAA 16800

16801 TTATGTCTTTACTGGTTATC GTGTAACTAAAAACAGTAAA GTACAAATAGGAGAGTACAC CTTTGAAAAAGGTGACTATG GTGATGCTGTTGTTTACCGA 16900

16901 GGTACAACAACTTACAAATT AAATGTTGGTGATTATTTTG TGCTGACATCACATACAGTA ATGCCATTAAGTGCACCTAC ACTAGTGCCACAAGAGCACT 17000

17001 ATGTTAGAATTACTGGCTTA TACCCAACACTCAATATCTC AGATGAGTTTTCTAGCAATG TTGCAAATTATCAAAAGGTT GGTATGCAAAAGTATTCTAC 17100

17101 ACTCCAGGGACCACCTGGTA CTGGTAAGAGTCATTTTGCT ATTGGCCTAGCTCTCCACTA CCCTTCTGCTCGCATAGTGT ATACAGCTTGCTCTCATGCC 17200

17201 GCTGTTGATGCACTATGTGA GAAGGCATTAAAATATTTGC CTATAGATAAATGTAGTAGA ATTATACCTGCACGTGCTCG TGTAGAGTGTTTTGATAAAT 17300

17301 TCAAAGTGAATTCAACATTA GAACAGTATGTCTTTTGTAC TGTAAATGCATTGCCTGAGA CGACAGCAGATATAGTTGTC TTTGATGAAATTTCAATGGC 17400

17401 CACAAATTATGATTTGAGTG TTGTCAATGCCAGATTACGT GCTAAGCACTATGTGTACAT TGGCGACCCTGCTCAATTAC CTACACCACGCACATTGCTA 17500

17501 ACTAAGGGCACACTAGAACC AGAATATTTCAATTCAGTGT GTAGACTTATGAAAACTATA GGTCCAGACATGTTCCTCGG AACTTGTCTGCGTTGTCCTG 17600

17601 CTGAAATTGTTGACACTGTG AGTGCTTTGGTTTATGATAA TAAGCTTAAAGCACATAAAG ACAAATCAGCTCAATGCTTT AAAATGTTTTATAAGGGTGT 17700

17701 TATCACGCATGATGTTTCAT CTGCAATTAACAGGCCACAA ATAGGCGTGGTAAGAGAATT CCTTACACGTAACCCTGCTT GGAGAAAAGCTGTCTTTATT 17800

17801 TCACCTTATAATTCACAGAA TGCTGTAGCCTCAAAGATTT TGGGACTACCAACTCAAACT GTTGATTCATCACAGGGCTC AGAATATGACTATGTCATAT 17900

17901 TCACTCAAACCACTGAAACA GCTCACTCTTGTAATGTAAA CAGATTTAATGTTGCTATTA CCAGAGCAAAAGTAGGCATA CTTTGCATAATGTCTGATAG 18000

18001 AGACCTTTATGACAAGTTGC AATTTACAAGTCTTGAAATT CCACGTAGGAATGTGGCAAC TTTACAAGCTGAAAATGTAA CAGGACTCTTTAAAGATTGT 18100

18101 AGTAAGGTAATCACTGGGTT ACATCCTACACAGGCACCTA CACACCTCAGTGTTGACACT AAATTCAAAACTGAAGGTTT ATGTGTTGACATACCTGGCA 18200

18201 TACCTAAGGACATGACCTAT AGAAGACTCATCTCTATGAT GGGTTTTAAAATGAATTATC AAGTTAATGGTTACCCTAAC ATGTTTATCACCCGCGAAGA 18300

18301 AGCTATAAGACATGTACGTG CATGGATTGGCTTCGATGTC GAGGGGTGTCATGCTACTAG AGAAGCTGTTGGTACCAATT TACCTTTACAGCTAGGTTTT 18400

18401 TCTACAGGTGTTAACCTAGT TGCTGTACCTACAGGTTATG TTGATACACCTAATAATACA GATTTTTCCAGAGTTGGTGC TAAACCACCGCCTGGAGATC 18500

18501 AATTTAAACACCTCATACCA CTTATGTACAAAGGACTTCC TTGGAATGTAGTGCGTATAA AGATTGTACAAATGTTAAGT GACACACTTAAAAATCTCTC 18600

18601 TGACAGAGTCGTATTTGTCT TATGGGCACATGGCTTTGAG TTGACATCTATGAAGTATTT TGTGAAAATAGGACCTGAGC GCACCTGTTGTCTATGTGAT 18700

18701 AGACGTGCCACATGCTTTTC CACTGCTTCAGACACTTATG CCTGTTGGCATCATTCTATT GGATTTGATTACGTCTATAA TCCGTTTATGATTGATGTTC 18800

18801 AACAATGGGGTTTTACAGGT AACCTACAAAGCAACCATGA TCTGTATTGTCAAGTCCATG GTAATGCACATGTAGCTAGT TGTGATGCAATCATGACTAG 18900

18901 GTGTCTAGCTGTCCACGAGT GCTTTGTTAAGCGTGTTGAC TGGACTATTGAATATCCTAT AATTGGTGATGAACTGAAGA TTAATGCGGCTTGTAGAAAG 19000

19001 GTTCAACACATGGTTGTTAA AGCTGCATTATTAGCAGACA AATTCCCAGTTCTTCACGAC ATTGGTAACCCTAAAGCTAT TAAGTGTGTACCTCAAGCTG 19100

19101 ATGTAGAATGGAAGTTCTAT GATGCACAGCCTTGTAGTGA CAAAGCTTATAAAATAGAAG AATTATTCTATTCTTATGCC ACACATTCTGACAAATTCAC 19200

19201 AGATGGTGTATGCCTATTTT GGAATTGCAATGTCGATAGA TATCCTGCTAATTCCATTGT TTGTAGATTTGACACTAGAG TGCTATCTAACCTTAACTTG 19300

19301 CCTGGTTGTGATGGTGGCAG TTTGTATGTAAATAAACATG CATTCCACACACCAGCTTTT GATAAAAGTGCTTTTGTTAA TTTAAAACAATTACCATTTT 19400

19401 TCTATTACTCTGACAGTCCA TGTGAGTCTCATGGAAAACA AGTAGTGTCAGATATAGATT ATGTACCACTAAAGTCTGCT ACGTGTATAACACGTTGCAA 19500

19501 TTTAGGTGGTGCTGTCTGTA GACATCATGCTAATGAGTAC AGATTGTATCTCGATGCTTA TAACATGATGATCTCAGCTG GCTTTAGCTTGTGGGTTTAC 19600

19601 AAACAATTTGATACTTATAA CCTCTGGAACACTTTTACAA GACTTCAGAGTTTAGAAAAT GTGGCTTTTAATGTTGTAAA TAAGGGACACTTTGATGGAC 19700

19701 AACAGGGTGAAGTACCAGTT TCTATCATTAATAACACTGT TTACACAAAAGTTGATGGTG TTGATGTAGAATTGTTTGAA AATAAAACAACATTACCTGT 19800

19801 TAATGTAGCATTTGAGCTTT GGGCTAAGCGCAACATTAAA CCAGTACCAGAGGTGAAAAT ACTCAATAATTTGGGTGTGG ACATTGCTGCTAATACTGTG 19900

19901 ATCTGGGACTACAAAAGAGA TGCTCCAGCACATATATCTA CTATTGGTGTTTGTTCTATG ACTGACATAGCCAAGAAACC AACTGAAACGATTTGTGCAC 20000

20001 CACTCACTGTCTTTTTGAT GGTAGAGTTGATGGTCAAGT AGACTTATTTAGAAATGCCC GTAATGGTGTTCTTATTACA GAAGGTAGTGTTAAAGGTTT 20100

20101 ACAACCATCTGTAGGTCCCA AACAAGCTAGTCTTAATGGA GTCACATTAATTGGAGAAGC CGTAAAAACACAGTTCAATT ATTATAAGAAAGTTGATGGT 20200

20201 GTTGTCCAACAATTACCTGA AACTTACTTTACTCAGAGTA GAAATTTACAAGAATTTAAA CCCAGGAGTCAAATGGAAAT TGATTTCTTAGAATTAGCTA 20300

20301 TGGATGAATTCATTGAACGG TATAAATTAGAAGGCTATGC CTTCGAACATATCGTTTATG GAGATTTTAGTCATAGTCAG TTAGGTGGTTTACATCTACT 20400

20401 GATTGGACTAGCTAAACGTT TTAAGGAATCACCTTTTGAA TTAGAAGATTTTATTCCTAT GGACAGTACAGTTAAAAACT ATTTCATAACAGATGCGCAA 20500

20501 ACAGGTTCATCTAAGTGTGT GTGTTCTGTTATTGATTTAT TACTTGATGATTTTGTTGAA ATAATAAAATCCCAAGATTT ATCTGTAGTTTCTAAGGTTG 20600

20601 TCAAAGTGACTATTGACTAT ACAGAAATTTCATTTATGCT TTGGTGTAAAGATGGCCATG TAGAAACATTTTACCCAAAA TTACAATCTAGTCAAGCGTG 20700

20701 GCAACCGGGTGTTGCTATGC CTAATCTTTACAAAATGCAA AGAATGCTATTAGAAAAGTG TGACCTTCAAAATTATGGTG ATAGTGCAACATTACCTAAA 20800

20801 GGCATAATGATGAATGTCGC AAAATATACTCAACTGTGTC AATATTTAAACACATTAACA TTAGCTGTACCCTATAATAT GAGAGTTATACATTTTGGTG 20900

20901 CTGGTTCTGATAAAGGAGTT GCACCAGGTACAGCTGTTTT AAGACAGTGGTTGCCTACGG GTACGCTGCTTGTCGATTCA GATCTTAATGACTTTGTCTC 21000

21001 TGATGCAGATTCAACTTTGA TTGGTGATTGTGCAACTGTA CATACAGCTAATAAATGGGA TCTCATTATTAGTGATATGT ACGACCCTAAGACTAAAAAT 21100

21101 GTTACAAAAGAAAATGACTC TAAAGAGGGTTTTTTCACTT ACATTTGTGGGTTTATACAA CAAAAGCTAGCTCTTGGAGG TTCCGTGGCTATAAAGATAA 21200

21201 CAGAACATTCTTGGAATGCT GATCTTTATAAGCTCATGGG ACACTTCGCATGGTGGACAG CCTTTGTTACTAATGTAAAT GCGTCATCATCTGAAGCATT 21300

21301 TTTAATTGGATGTAATTATC TTGGCAAACCACGCGAACAA ATAGATGGTTATGTCATGCA TGCAAATTACATATTTTGGA GGAATACAAATCCAATTCAG 21400

21401 TTGTCTTCCTATTCTTTATT TGACATGAGTAAATTTCCCC TTAAATTAAGGGGTACTGCT GTTATGTCTTTAAAAGAAGG TCAAATCAATGATATGATTT 21500

21501 TATCTCTTCTTAGTAAAGGT AGACTTATAATTAGAGAAAA CAACAGAGTTGTTATTTCTA GTGATGTTCTTGTTAACAAC TAAACGAACAATGTTTGTTT 21600

21601 TTCTTGTTTTATTGCCACTA GACTCTAGTCAGTGTGTTAA TCTTACAACCAGAACTCAAT TACCCCCTGCATACACTAAT TCTTTCACACGTGGTGTTTA 21700

21701 TTACCCTGACAAAGTTTTCA GATCCTCAGTTTTACATTCA ACTCAGGACTTGTTCTTACC TTTCTTTTCCAATGTTACTT GGTTCCATGCTATACATGTC 21800

21801 TCTGGGACCAATGGTACTAA GAGGTTTGATAACCCTGTCC TACCATTTAATGATGGTGTT TATTTTGCTTCCACTGAGAA GTCTAACATAATAAGAGGCT 21900

21901 GGATTTTTGGTACTACTTTA GATTCGAAGACCCAGTCCCT ACTTATTGTTAATAACGCTA CTAATGTTGTTATTAAAGTC TGTGAATTTCAATTTTGTAA 22000

22001 TGATCCATTTTTGGGTGTTT ATTACCACAAAAACAACAAA AGTTGGATGGAAAGTGAGTT CAGAGTTTATTCTAGTGCGA ATAATTGCACTTTTGAATAT 22100

22101 GTCTCTCAGCCTTTTCTTAT GGACCTTGAAGGAAAACAGG GTAATTTCAAAAATCTTAGG GAATTTGTGTTTAAGAATAT TGATGGTTATTTTAAAATAT 22200

22201 ATTCTAAGCACACGCCTATT AATTTAGTGCGTGATCTCCC TCAGGGTTTTTCGGCTTTAG AACCATTGGTAGATTTGCCA ATGGGTATTAACATCACTAG 22300

22301 GTTTCAAACTTTACTTGCTT TACATAGAAGTTATTTGACT CCTGGTGATTCTTCTTCAGG TTGGACAGCTGGTGCTGCAG CTTATTATGTGGGTTATCTT 22400

22401 CAACCTAGGACTTTTCTATT AAAATATAATGAAAATGGAA CCATTACAGATGCTGTAGAC TGTGCACTTGACCCTCTCTC AGAAACAAAGTGTACGTTGA 22500

22501 AATCCTTCACTGTAGAAAAA GGAATCTATCAAACTTCTAA CTTTAGAGTCCAACCAACAG AATCTATTGTTAGATTTCCT AATATTACAAACTTGTGCCC 22600

22601 TTTTGGTGAAGTTTTTAACG CCACCAGATTTGCATCTGTT TATGCTTGGAACAGGAAGAG AATCAGCAACTGTGTTGCTG ATTATTCTGTCCTATATAAT 22700

22701 TCCGCATCATTTTCCACTTT TAAGTGTTATGGAGTGTCTC CTACTAAATTAAATGATCTC TGCTTTACTAATGTCTATGC AGATTCATTTGTAATTAGAG 22800

22801 GTGATGAAGTCAGACAAATC GCTCCAGGGCAAACTGGAAA GATTGCTGATTATAATTATA AATTACCAGATGATTTTACA GGCTGCGTTATAGCTTGGAA 22900

22901 TTCTAACAATCTTGATTCTA AGGTTGGTGGTAATTATAAT TACCTGTATAGATTGTTTAG GAAGTCTAATCTCAAACCTT TTGAGAGAGATATTTCAACT 23000

23001 GAAATCTATCAGGCCGGTAG CACACCTTGTAATGGTGTTG AAGGTTTTAATTGTTACTTT CCTTTACAATCATATGGTTT CCAACCCACTAATGGTGTTG 23100

23101 GTTACCAACCATACAGAGTA GTAGTACTTTCTTTTGAACT TCTACATGCACCAGCAACTG TTTGTGGACCTAAAAAGTCT ACTAATTTGGTTAAAAACAA 23200

23201 ATGTGTCAATTTCAACTTCA ATGGTTTAACAGGCACAGGT GTTCTTACTGAGTCTAACAA AAAGTTTCTGCCTTTCCAAC AATTTGGCAGAGACATTGCT 23300

23301 GACACTACTGATGCTGTCCG TGATCCACAGACACTTGAGA TTCTTGACATTACACCATGT TCTTTTGGTGGTGTCAGTGT TATAACACCAGGAACAAATA 23400

23401 CTTCTAACCAGGTTGCTGTT CTTTATCAGGGTGTTAACTG CACAGAAGTCCCTGTTGCTA TTCATGCAGATCAACTTACT CCTACTTGGCGTGTTTATTC 23500

23501 TACAGGTTCTAATGTTTTTC AAACACGTGCAGGCTGTTTA ATAGGGGCTGAACATGTCAA CAACTCATATGAGTGTGACA TACCCATTGGTGCAGGTATA 23600

23601 TGCGCTAGTTATCAGACTCA GACTAATTCTCCTCGGCGGG CACGTAGTGTAGCTAGTCAA TCCATCATTGCCTACACTAT GTCACTTGGTGCAGAAAATT 23700

23701 CAGTTGCTTACTCTAATAAC TCTATTGCCATACCCACAAA TTTTACTATTAGTGTTACCA CAGAAATTCTACCAGTGTCT ATGACCAAGACATCAGTAGA 23800

23801 TTGTACAATGTACATTTGTG GTGATTCAACTGAATGCAGC AATCTTTTGTTGCAATATGG CAGTTTTTGTACACAATTAA ACCGTGCTTTAACTGGAATA 23900

23901 GCTGTTGAACAAGACAAAAA CACCCAAGAAGTTTTTGCAC AAGTCAAACAAATTTACAAA ACACCACCAATTAAAGATTT TGGTGGTTTTAATTTTTCAC 24000

24001 AAATATTACCAGATCCATCA AAACCAAGCAAGAGGTCATT TATTGAAGATCTACTTTTCA ACAAAGTGACACTTGCAGAT GCTGGCTTCATCAAACAATA 24100

24101 TGGTGATTGCCTTGGTGATA TTGCTGCTAGAGACCTCATT TGTGCACAAAAGTTTAACGG CCTTACTGTTTTGCCACCTT TGCTCACAGATGAAATGATT 24200

24201 GCTCAATACACTTCTGCACT GTTAGCGGGTACAATCACTT CTGGTTGGACCTTTGGTGCA GGTGCTGCATTACAAATACC ATTTGCTATGCAAATGGCTT 24300

24301 ATAGGTTTAATGGTATTGGA GTTACACAGAATGTTCTCTA TGAGAACCAAAAATTGATTG CCAACCAATTTAATAGTGCT ATTGGCAAAATTCAAGACTC 24400

24401 ACTTTCTTCCACAGCAAGTG CACTTGGAAAACTTCAAGAT GTGGTCAACCAAAATGCACA AGCTTTAAACACGCTTGTTA AACAACTTAGCTCCAATTTT 24500

24501 GGTGCAATTTCAAGTGTTTT AAATGATATCCTTTCACGTC TTGACAAAGTTGAGGCTGAA GTGCAAATTGATAGGTTGAT CACAGGCAGACTTCAAAGTT 24600

24601 TGCAGACATATGTGACTCAA CAATTAATTAGAGCTGCAGA AATCAGAGCTTCTGCTAATC TTGCTGCTACTAAAATGTCA GAGTGTGTACTTGGACAATC 24700

24701 AAAAAGAGTTGATTTTTGTG GAAAGGGCTATCATCTTATG TCCTTCCCTCAGTCAGCACC TCATGGTGTAGTCTTCTTGC ATGTGACTTATGTCCCTGCA 24800

24801 CAAGAAAAGAACTTCACAAC TGCTCCTGCCATTTGTCATG ATGGAAAAGCACACTTTCCT CGTGAAGGTGTCTTTGTTTC AAATGGCACACACTGGTTTG 24900

24901 TAACACAAAGGAATTTTTAT GAACCACAAATCATTACTAC AGACAACACATTTGTGTCTG GTAACTGTGATGTTGTAATA GGAATTGTCAACAACACAGT 25000

25001 TTATGATCCTTTGCAACCTG AATTAGACTCATTCAAGGAG GAGTTAGATAAATATTTTAA GAATCATACATCACCAGATG TTGATTTAGGTGACATCTCT 25100

25101 GGCATTAATGCTTCAGTTGT AAACATTCAAAAAGAAATTG ACCGCCTCAATGAGGTTGCC AAGAATTTAAATGAATCTCT CATCGATCTCCAAGAACTTG 25200

25201 GAAAGTATGAGCAGTATATA AAATGGCCATGGTACATTTG GCTAGGTTTTATAGCTGGCT TGATTGCCATAGTAATGGTG ACAATTATGCTTTGCTGTAT 25300

25301 GACCAGTTGCTGTAGTTGTC TCAAGGGCTGTTGTTCTTGT GGATCCTGCTGCAAATTTGA TGAAGACGACTCTGAGCCAG TGCTCAAAGGAGTCAAATTA 25400

25401 CATTACACATAAACGAACTT ATGGATTTGTTTATGAGAAT CTTCACAATTGGAACTGTAA CTTTGAAGCAAGGTGAAATC AAGGATGCTACTCCTTCAGA 25500

25501 TTTTGTTCGCGCTACTGCAA CGATACCGATACAAGCCTCA CTCCCTTTCGGATGGCTTAT TGTTGGCGTTGCACTTCTTG CTGTTTTTCATAGCGCTTCC 25600

25601 AAAATCATAACCCTCAAAAA GAGATGGCAACTAGCACTCT CCAAGGGTGTTCACTTTGTT TGCAACTTGCTGTTGTTGTT TGTAACAGTTTACTCACACC 25700

25701 TTTTGCTCGTTGCTGCTGGC CTTGAAGCCCCTTTTCTCTA TCTTTATGCTTTAGTCTACT TCTTGCAGAGTATAAACTTT GTAAGAATAATAATGAGGCT 25800

25801 TTGGCTTTGCTGGAAATGCC GTTCCAAAAACCCATTACTT TATGATGCCAACTATTTTCT TTGCTGGCATACTAATTGTT ACGACTATTGTATACCTTAC 25900

25901 AATAGTGTAACTTCTTCAAT TGTCATTACTTCAGGTGATG GCACAACAAGTCCTATTTCT GAACATGACTACCAGATTGG TGGTTATACTGAAAAATGGG 26000

26001 AATCTGGAGTAAAAGACTGT GTTGTATTACACAGTTACTT CACTTCAGACTATTACCAGC TGTACTCAACTCAATTGAGT ACAGACACTGGTGTTGAACA 26100

26101 TGTTACCTTCTTCATCTACA ATAAAATTGTTGATGAGCCT GAAGAACATGTCCAAATTCA CACAATCGACGGTTCATCCG GAGTTGTTAATCCAGTAATG 26200

26201 GAACCAATTTATGATGAACC GACGACGACTACTAGCGTGC CTTTGTAAGCACAAGCTGAT GAGTACGAACTTATGTACTC ATTCGTTTCGGAAGAGACAG 26300

26301 GTACGTTAATAGTTAATAGC GTACTTCTTTTTCTTGCTTT CGTGGTATTCTTGCTAGTTA CACTAGCCATCCTTACTGCG CTTCGATTGTGTGCGTACTG 26400

26401 CTGCAATATTGTTAACGTGA GTCTTGTAAAACCTTCTTTT TACGTTTACTCTCGTGTTAA AAATCTGAATTCTTCTAGAG TTCCTGATCTTCTGGTCTAA 26500

26501 ACGAACTAAATATTATATTA GTTTTTCTGTTTGGAACTTT AATTTTAGCCATGGCAGATT CCAACGGTACTATTACCGTT GAAGAGCTTAAAAAGCTCCT 26600

26601 TGAACAATGGAACCTAGTAA TAGGTTTCCTATTCCTTACA TGGATTTGTCTTCTACAATT TGCCTATGCCAACAGGAATA GGTTTTTGTATATAATTAAG 26700

26701 TTAATTTTCCTCTGGCTGTT ATGGCCAGTAACTTTAGCTT GTTTTGTGCTTGCTGCTGTT TACAGAATAAATTGGATCAC CGGTGGAATTGCTATCGCAA 26800

26801 TGGCTTGTCTTGTAGGCTTG ATGTGGCTCAGCTACTTCAT TGCTTCTTTCAGACTGTTTG CGCGTACGCGTTCCATGTGG TCATTCAATCCAGAAACTAA 26900

26901 CATTCTTCTCAACGTGCCAC TCCATGGCACTATTCTGACC AGACCGCTTCTAGAAAGTGA ACTCGTAATCGGAGCTGTGA TCCTTCGTGGACATCTTCGT 27000

27001 ATTGCTGGACACCATCTAGG ACGCTGTGACATCAAGGACC TGCCTAAAGAAATCACTGTT GCTACATCACGAACGCTTTC TTATTACAAATTGGGAGCTT 27100

27101 CGCAGCGTGTAGCAGGTGAC TCAGGTTTTGCTGCATACAG TCGCTACAGGATTGGCAACT ATAAATTAAACACAGACCAT TCCAGTAGCAGTGACAATAT 27200

27201 TGCTTTGCTTGTACAGTAAG TGACAACAGATGTTTCATCT CGTTGACTTTCAGGTTACTA TAGCAGAGATATTACTAATT ATTATGAGGACTTTTAAAGT 27300

27301 TTCCATTTGGAATCTTGATT ACATCATAAACCTCATAATT AAAAATTTATCTAAGTCACT AACTGAGAATAAATATTCTC AATTAGATGAAGAGCAACCA 27400

27401 ATGGAGATTGATTAAACGAA CATGAAAATTATTCTTTTCT TGGCACTGATAACACTCGCT ACTTGTGAGCTTTATCACTA CCAAGAGTGTGTTAGAGGTA 27500

27501 CAACAGTACTTTTAAAAGAA CCTTGCTCTTCTGGAACATA CGAGGGCAATTCACCATTTC ATCCTCTAGCTGATAACAAA TTTGCACTGACTTGCTTTAG 27600

27601 CACTCAATTTGCTTTTGCTT GTCCTGACGGCGTAAAACAC GTCTATCAGTTACGTGCCAG ATCAGTTTCACCTAAACTGT TCATCAGACAAGAGGAAGTT 27700

27701 CAAGAACTTTACTCTCCAAT TTTTCTTATTGTTGCGGCAA TAGTGTTTATAACACTTTGC TTCACACTCAAAAGAAAAAC AGAATGATTGAACTTTCATT 27800

27801 AATTGACTTCTATTTGTGCT TTTTAGCCTTTCTGCTATTC CTTGTTTTAATTATGCTTAT TATCTTTTGGTTCTCACTTG AACTGCAAGATCATAATGAA 27900

27901 ACTTGTCACGCCTAAACGAA CATGAAATTTCTTGTTTTCT TAGGAATCATCACAACTGTA GCTGCATTTCACCAAGAATG TAGTTTACAGTCATGTACTC 28000

28001 AACATCAACCATATGTAGTT GATGACCCGTGTCCTATTCA CTTCTATTCTAAATGGTATA TTAGAGTAGGAGCTAGAAAA TCAGCACCTTTAATTGAATT 28100

28101 GTGCGTGGATGAGGTTGGTT CTAAATCACCCATTCAGTAC ATCGATATCGGTAATTATAC AGTTTCCTGTTTACCTTTTA CAATTAATTGCCAGGAACCT 28200

28201 AAATTGGGTAGTCTTGTAGT GCGTTGTTCGTTCTATGAAG ACTTTTTAGAGTATCATGAC GTTCGTGTTGTTTTAGATTT CATCTAAACGAACAAACTAA 28300

28301 AATGTCTGATAATGGACCCC AAAATCAGCGAAATGCACCC CGCATTACGTTTGGTGGACC CTCAGATTCAACTGGCAGTA ACCAGAATGGAGAACGCAGT 28400

28401 GGGGCGCGATCAAAACAACG TCGGCCCCAAGGTTTACCCA ATAATACTGCGTCTTGGTTC ACCGCTCTCACTCAACATGG CAAGGAAGACCTTAAATTCC 28500

28501 CTCGAGGACAAGGCGTTCCA ATTAACACCAATAGCAGTCC AGATGACCAAATTGGCTACT ACCGAAGAGCTACCAGACGA ATTCGTGGTGGTGACGGTAA 28600

28601 AATGAAAGATCTCAGTCCAA GATGGTATTTCTACTACCTA GGAACTGGGCCAGAAGCTGG ACTTCCCTATGGTGCTAACA AAGACGGCATCATATGGGTT 28700

28701 GCAACTGAGGGAGCCTTGAA TACACCAAAAGATCACATTG GCACCCGCAATCCTGCTAAC AATGCTGCAATCGTGCTACA ACTTCCTCAAGGAACAACAT 28800

28801 TGCCAAAAGGCTTCTACGCA GAAGGGAGCAGAGGCGGCAG TCAAGCCTCTGCTCGTTCCT CATCACGTAGTCGCAACAGT TCAAGAAATTCAACTCCAGG 28900

28901 CAGCAGTAGGGGAACTTCTC CTGCTAGAATGGCTGGCAAT GGCGGTGATGCTGCTCTTGC TTTGCTGCTGCTTGACAGAT TGAACCAGCTTGAGAGCAAA 29000

29001 ATGTCTGGTAAAGGCCAACA ACAACAAGGCCAAACTGTCA CTAAGAAATCTGCTGCTGAG GCTTCTAAGAAGCCTCGGCA AAAACGTACTGCCACTAAAG 29100

29101 CATACAATGTAACACAAGCT TTCGGCAGACGTGGTCCAGA ACAAACCCAAGGAAATTTTG GGGACCAGGAACTAATCAGA CAAGGAACTGATTACAAACA 29200

29201 TTGGCCGCAAATTGCACAAT TTGCCCCCAGCGCTTCAGCG TTCTTCGGAATGTCGCGCAT TGGCATGGAAGTCACACCTT CGGGAACGTGGTTGACCTAC 29300

29301 ACAGGTGCCATCAAATTGGA TGACAAAGATCCAAATTTCA AAGATCAAGTCATTTTGCTG AATAAGCATATTGACGCATA CAAAACATTCCCACCAACAG 29400

29401 AGCCTAAAAAGGACAAAAAG AAGAAGGCTGATGAAACTCA AGCCTTACCGCAGAGACAGA AGAAACAGCAAACTGTGACT CTTCTTCCTGCTGCAGATTT 29500

29501 GGATGATTTCTCCAAACAAT TGCAACAATCCATGAGCAGT GCTGACTCAACTCAGGCCTA AACTCATGCAGACCACACAA GGCAGATGGGCTATATAAAC 29600

29601 GTTTTCGCTTTTCCGTTTAC GATATATAGTCTACTCTTGT GCAGAATGAATTCTCGTAAC TACATAGCACAAGTAGATGT AGTTAACTTTAATCTCACAT 29700

29701 AGCAATCTTTAATCAGTGTG TAACATTAGGGAGGACTTGA AAGAGCCACCACATTTTCAC CGAGGCCACGCGGAGTACGA TCGAGTGTACAGTGAACAAT 29800

29801 GCTAGGGAGAGCTGCCTATA TGGAAGAGCCCTAATGTGTA AAATTAATTTTAGTAGTGCT ATCCCCATGTGATTTTAATA GCTTCTTAGGAGAATGACAA 29900

29901 AAAAAAAATCACATGGGGAT AGCAC 29925

```
EMBOSS_001         1 MFVFLVLLPLVSSQCVNLTTRTQLPPAYTNSFTRGVYYPDKVFRSSVLHS     50
                     ||||||||.|||||||||||||||||||||||||||||||||||||||||
EMBOSS_001         1 MFVFLVLLSLVSSQCVNLTTRTQLPPAYTNSFTRGVYYPDKVFRSSVLHS     50

EMBOSS_001        51 TQDLFLPFFSNVTWFHAIHVSGTNGTKRFDNPVLPFNDGVYFASTEKSNI    100
                     ||||||||||||||||||||||||||||||||||||||||||||||||||
EMBOSS_001        51 TQDLFLPFFSNVTWFHAIHVSGTNGTKRFDNPVLPFNDGVYFASTEKSNI    100

EMBOSS_001       101 IRGWIFGTTLDSKTQSLLIVNNATNVVIKVCEFQFCNDPFLGVYYHKNNK    150
                     ||||||||||||||||||||||||||||||||||||||||||||||||||
EMBOSS_001       101 IRGWIFGTTLDSKTQSLLIVNNATNVVIKVCEFQFCNDPFLGVYYHKNNK    150

EMBOSS_001       151 SWMESEFRVYSSANNCTFEYVSQPFLMDLEGKQGNFKNLREFVFKNIDGY    200
                     ||||||||||||||||||||||||||||||||||||||||||||||||||
EMBOSS_001       151 SWMESEFRVYSSANNCTFEYVSQPFLMDLEGKQGNFKNLREFVFKNIDGY    200

EMBOSS_001       201 FKIYSKHTPINLVRDLPQGFSALEPLVDLPIGINITRFQTLLALHRSYLT    250
                     |||||||||||||||||||||||||||||||||||||.||||||||||||
EMBOSS_001       201 FKIYSKHTPINLVRDLPQGFSALEPLVDLPIGINITRLQTLLALHRSYLT    250

EMBOSS_001       251 PGDSSSGWTAGAAAYYVGYLQPRTFLLKYNENGTITDAVDCALDPLSETK    300
                     ||||||||||||||||||||||||||||||||||||||||||||||||||
EMBOSS_001       251 PGDSSSGWTAGAAAYYVGYLQPRTFLLKYNENGTITDAVDCALDPLSETK    300

EMBOSS_001       301 CTLKSFTVEKGIYQTSNFRVQPTESIVRFPNITNLCPFGEVFNATRFASV    350
                     ||||||||||||||||||||||||||||||||||||||||||||||||||
EMBOSS_001       301 CTLKSFTVEKGIYQTSNFRVQPTESIVRFPNITNLCPFGEVFNATRFASV    350

EMBOSS_001       351 YAWNRKRISNCVADYSVLYNSASFSTFKCYGVSPTKLNDLCFTNVYADSF    400
                     ||||||||||||||||||||||||||||||||||||||||||||||||||
EMBOSS_001       351 YAWNRKRISNCVADYSVLYNSASFSTFKCYGVSPTKLNDLCFTNVYADSF    400

EMBOSS_001       401 VIRGDEVRQIAPGQTGKIADYNYKLPDDFTGCVIAWNSNNLDSKVGGNYN    450
                     ||||||||||||||||||||||||||||||||||||||||||||||||||
EMBOSS_001       401 VIRGDEVRQIAPGQTGKIADYNYKLPDDFTGCVIAWNSNNLDSKVGGNYN    450

EMBOSS_001       451 YLYRLFRKSNLKPFERDISTEIYQAGSTPCNGVEGFNCYFPLQSYGFQPT    500
                     ||||||||||||||||||||||||||||||||||||||||||||||||||
EMBOSS_001       451 YLYRLFRKSNLKPFERDISTEIYQAGSTPCNGVEGFNCYFPLQSYGFQPT    500

EMBOSS_001       501 NGVGYQPYRVVVLSFELLHAPATVCGPKKSTNLVKNKCVNFNFNGLTGTG    550
                     ||||||||||||||||||||||||||||||||||||||||||||||||||
EMBOSS_001       501 NGVGYQPYRVVVLSFELLHAPATVCGPKKSTNLVKNKCVNFNFNGLTGTG    550

EMBOSS_001       551 VLTESNKKFLPFQQFGRDIADTTDAVRDPQTLEILDITPCSFGGVSVITP    600
                     ||||||||||||||||||||||||||||||||||||||||||||||||||
EMBOSS_001       551 VLTESNKKFLPFQQFGRDIADTTDAVRDPQTLEILDITPCSFGGVSVITP    600
```

Figure S**1.** Difference of discovered strain 1 spike protein sequence and original strain spike protein sequence

```
EMBOSS_001       601 GTNTSNQVAVLYQDVNCTEVPVAIHADQLTPTWRVYSTGSNVFQTRAGCL    650
                     ..:|||||||||||||||||||||||||||||||||||||||||||||||
EMBOSS_001       601 EVDTSNQVAVLYQDVNCTEVPVAIHADQLTPTWRVYSTGSNVFQTRAGCL    650

EMBOSS_001       651 IGAEHVNNSYECDIPIGAGICASYQTQTNSPRRARSVASQSIIAYTMSLG    700
                     ||||||||||||||||||||||||||||||||||||||||||||||||||
EMBOSS_001       651 IGAEHVNNSYECDIPIGAGICASYQTQTNSPRRARSVASQSIIAYTMSLG    700

EMBOSS_001       701 AENSVAYSNNSIAIPTNFTISVTTEILPVSMTKTSVDCTMYICGDSTECS    750
                     ||||||||||||||||||||||||||||||||||||||||||||||||||
EMBOSS_001       701 AENSVAYSNNSIAIPTNFTISVTTEILPVSMTKTSVDCTMYICGDSTECS    750

EMBOSS_001       751 NLLLQYGSFCTQLNRALTGIAVEQDKNTQEVFAQVKQIYKTPPIKDFGGF    800
                     |||||||||||||||.||||||||||||||||||||||||||||||||||
EMBOSS_001       751 NLLLQYGSFCTQLNRGLTGIAVEQDKNTQEVFAQVKQIYKTPPIKDFGGF    800

EMBOSS_001       801 NFSQILPDPSKPSKRSFIEDLLFNKVTLADAGFIKQYGDCLGDIAARDLI    850
                     ||||||||||||||||||||||||||||||||||||||||||||||||.|
EMBOSS_001       801 NFSQILPDPSKPSKRSFIEDLLFNKVTLADAGFIKQYGDCLGDIAARDHI    850

EMBOSS_001       851 CAQKFNGLTVLPPLLTDEMIAQYTSALLAGTITSGWTFGAGAALQIPFAM    900
                     ||||||||||||||||||||||||||||||||||||||||||||||||||
EMBOSS_001       851 CAQKFNGLTVLPPLLTDEMIAQYTSALLAGTITSGWTFGAGAALQIPFAM    900

EMBOSS_001       901 QMAYRFNGIGVTQNVLYENQKLIANQFNSAIGKIQDSLSSTASALGKLQD    950
                     ||||||||||||||||||||||||||||||||||||||||||||||||||
EMBOSS_001       901 QMAYRFNGIGVTQNVLYENQKLIANQFNSAIGKIQDSLSSTASALGKLQD    950

EMBOSS_001       951 VVNQNAQALNTLVKQLSSNFGAISSVLNDILSRLDKVEAEVQIDRLITGR   1000
                     ||||||||||||||||||||||||||||||||||||||||||||||||||
EMBOSS_001       951 VVNQNAQALNTLVKQLSSNFGAISSVLNDILSRLDKVEAEVQIDRLITGR   1000

EMBOSS_001      1001 LQSLQTYVTQQLIRAAEIRASANLAATKMSECVLGQSKRVDFCGKGYHLM   1050
                     ||||||||||||||||||||||||||||||..:|||||||||||||||||
EMBOSS_001      1001 LQSLQTYVTQQLIRAAEIRASANLAATKMSLRLLGQSKRVDFCGKGYHLM   1050

EMBOSS_001      1051 SFPQSAPHGVVFLHVTYVPAQEKNFTTAPAICHDGKAHFPREGVFVSNGT   1100
                     ||||||||||||||||||||||||||||||...|||||||||||||||||
EMBOSS_001      1051 SFPQSAPHGVVFLHVTYVPAQEKNFTTAPAKSDDGKAHFPREGVFVSNGT   1100

EMBOSS_001      1101 HWFVTQRNFYEPQIITTDNTFVSGNCDVVIGIVNNTVYDPLQPELDSFKE   1150
                     ||||||||||||||||||||||||||||||||||||||||||||||||||
EMBOSS_001      1101 HWFVTQRNFYEPQIITTDNTFVSGNCDVVIGIVNNTVYDPLQPELDSFKE   1150

EMBOSS_001      1151 ELDKYFKNHTSPDVDLGDISGINASVVNIQKEIDRLNEVAKNLNESLIDL   1200
                     |||||||||||.||||||||||||||||||||||||||||||||||||||
EMBOSS_001      1151 ELDKYFKNHTSADVDLGDISGINASVVNIQKEIDRLNEVAKNLNESLIDL   1200

EMBOSS_001      1201 QELGKYEQYIKWPWYIWLGFIAGLIAIVMVTIMLCCMTSCCSCLKGCCSC   1250
                     ||||||||||||||||||||||||||||||||||||||||||||||||||
EMBOSS_001      1201 QELGKYEQYIKWPWYIWLGFIAGLIAIVMVTIMLCCMTSCCSCLKGCCSC   1250

EMBOSS_001      1251 GSCCKFDEDDSEPVLKGVKLHYT   1273
                     |||||||||||||||||||||||
EMBOSS_001      1251 GSCCKFDEDDSEPVLKGVKLHYT   1273
```

Figure S1. Difference of discovered strain 1 spike protein sequence and original strain spike protein sequence

```
EMBOSS_001         1 MFVFLVLLPLVSSQCVNLTTRTQLPPAYTNSFTRGVYYPDKVFRSSVLHS     50
                     |||||||||||||||||||||||||.:.||||||||||||||||||||||
EMBOSS_001         1 MFVFLVLLPLVSSQCVNLTTRTQLPKSDTNSFTRGVYYPDKVFRSSVLHS     50

EMBOSS_001        51 TQDLFLPFFSNVTWFHAIHVSGTNGTKRFDNPVLPFNDGVYFASTEKSNI    100
                     ||||||||||||||||||||||||||||||||||||||||||||||||||
EMBOSS_001        51 TQDLFLPFFSNVTWFHAIHVSGTNGTKRFDNPVLPFNDGVYFASTEKSNI    100

EMBOSS_001       101 IRGWIFGTTLDSKTQSLLIVNNATNVVIKVCEFQFCNDPFLGVYYHKNNK    150
                     ||||||||||||||||||||||||||||||||||||||||||||||||||
EMBOSS_001       101 IRGWIFGTTLDSKTQSLLIVNNATNVVIKVCEFQFCNDPFLGVYYHKNNK    150

EMBOSS_001       151 SWMESEFRVYSSANNCTFEYVSQPFLMDLEGKQGNFKNLREFVFKNIDGY    200
                     ||||||||||||||||||||||||||||||||||||||||||||||||||
EMBOSS_001       151 SWMESEFRVYSSANNCTFEYVSQPFLMDLEGKQGNFKNLREFVFKNIDGY    200

EMBOSS_001       201 FKIYSKHTPINLVRDLPQGFSALEPLVDLPIGINITRFQTLLALHRSYLT    250
                     ||||||||||||||||||||||||||||||||||||||||||||||||||
EMBOSS_001       201 FKIYSKHTPINLVRDLPQGFSALEPLVDLPIGINITRFQTLLALHRSYLT    250

EMBOSS_001       251 PGDSSSGWTAGAAAYYVGYLQPRTFLLKYNENGTITDAVDCALDPLSETK    300
                     ||||||||||||||||||||||||||||||||||||||||||||||||||
EMBOSS_001       251 PGDSSSGWTAGAAAYYVGYLQPRTFLLKYNENGTITDAVDCALDPLSETK    300

EMBOSS_001       301 CTLKSFTVEKGIYQTSNFRVQPTESIVRFPNITNLCPFGEVFNATRFASV    350
                     ||||||||||||||||||||||||||||||||||||||||||||||||||
EMBOSS_001       301 CTLKSFTVEKGIYQTSNFRVQPTESIVRFPNITNLCPFGEVFNATRFASV    350

EMBOSS_001       351 YAWNRKRISNCVADYSVLYNSASFSTFKCYGVSPTKLNDLCFTNVYADSF    400
                     ||||||||||||||||||||||||||||||||||||||||||||||||||
EMBOSS_001       351 YAWNRKRISNCVADYSVLYNSASFSTFKCYGVSPTKLNDLCFTNVYADSF    400

EMBOSS_001       401 VIRGDEVRQIAPGQTGKIADYNYKLPDDFTGCVIAWNSNNLDSKVGGNYN    450
                     ||||||||||||||||||||||||||||||||||||||||||||||||||
EMBOSS_001       401 VIRGDEVRQIAPGQTGKIADYNYKLPDDFTGCVIAWNSNNLDSKVGGNYN    450

EMBOSS_001       451 YLYRLFRKSNLKPFERDISTEIYQAGSTPCNGVEGFNCYFPLQSYGFQPT    500
                     ||||||||||||||||||||||||||||||||||||||||||||||||||
EMBOSS_001       451 YLYRLFRKSNLKPFERDISTEIYQAGSTPCNGVEGFNCYFPLQSYGFQPT    500

EMBOSS_001       501 NGVGYQPYRVVVLSFELLHAPATVCGPKKSTNLVKNKCVNFNFNGLTGTG    550
                     ||||||||||||||||||||||||||||||||||||||||||||||||||
EMBOSS_001       501 NGVGYQPYRVVVLSFELLHAPATVCGPKKSTNLVKNKCVNFNFNGLTGTG    550

EMBOSS_001       551 VLTESNKKFLPFQQFGRDIADTTDAVRDPQTLEILDITPCSFGGVSVITP    600
                     ||||||||||||||||||||||.|||||||||||||||||||||||||||
EMBOSS_001       551 VLTESNKKFLPFQQFGRDIADTIDAVRDPQTLEILDITPCSFGGVSVITP    600

EMBOSS_001       601 GTNTSNQVAVLYQDVNCTEVPVAIHADQLTPTWRVYSTGSNVFQTRAGCL    650
                     ||||||||||||||||||||||||||||||||||||||||||||||||||
EMBOSS_001       601 GTNTSNQVAVLYQDVNCTEVPVAIHADQLTPTWRVYSTGSNVFQTRAGCL    650

EMBOSS_001       651 IGAEHVNNSYECDIPIGAGICASYQTQTNSPRRARSVASQSIIAYTMSLG    700
                     ||||||||||||||||||||||||||||||||||||||||||||||||||
EMBOSS_001       651 IGAEHVNNSYECDIPIGAGICASYQTQTNSPRRARSVASQSIIAYTMSLG    700
```

Figure S**2.** Difference of discovered strain 2 spike protein sequence and original strain spike protein sequence

```
EMBOSS_001       701 AENSVAYSNNSIAIPTNFTISVTTEILPVSMTKTSVDCTMYICGDSTECS    750
                     ||||||||||||||||||||||||||||||||||||||||||||||||||
EMBOSS_001       701 AENSVAYSNNSIAIPTNFTISVTTEILPVSMTKTSVDCTMYICGDSTECS    750

EMBOSS_001       751 NLLLQYGSFCTQLNRALTGIAVEQDKNTQEVFAQVKQIYKTPPIKDFGGF    800
                     ||||||||||||||||||||||||||||||||||||||||||||||||||
EMBOSS_001       751 NLLLQYGSFCTQLNRALTGIAVEQDKNTQEVFAQVKQIYKTPPIKDFGGF    800

EMBOSS_001       801 NFSQILPDPSKPSKRSFIEDLLFNKVTLADAGFIKQYGDCLGDIAARDLI    850
                     ||||||||||||||||||||||||||||||||||||||||||||||||||
EMBOSS_001       801 NFSQILPDPSKPSKRSFIEDLLFNKVTLADAGFIKQYGDCLGDIAARDLI    850

EMBOSS_001       851 CAQKFNGLTVLPPLLTDEMIAQYTSALLAGTITSGWTFGAGAALQIPFAM    900
                     ||||||||||||||||||||||||||||||||||||||||||||||||||
EMBOSS_001       851 CAQKFNGLTVLPPLLTDEMIAQYTSALLAGTITSGWTFGAGAALQIPFAM    900

EMBOSS_001       901 QMAYRFNGIGVTQNVLYENQKLIANQFNSAIGKIQDSLSSTASALGKLQD    950
                     ||||||||||||||||||||||||||||||||||||||||||||||||||
EMBOSS_001       901 QMAYRFNGIGVTQNVLYENQKLIANQFNSAIGKIQDSLSSTASALGKLQD    950

EMBOSS_001       951 VVNQNAQALNTLVKQLSSNFGAISSVLNDILSRLDKVEAEVQIDRLITGR   1000
                     ||||||||||||||||||||||||||||||||||||||||||||||||||
EMBOSS_001       951 VVNQNAQALNTLVKQLSSNFGAISSVLNDILSRLDKVEAEVQIDRLITGR   1000

EMBOSS_001      1001 LQSLQTYVTQQLIRAAEIRASANLAATKMSECVLGQSKRVDFCGKGYHLM   1050
                     ||||||||||||||||||||||||||||||||||||||||||||||||||
EMBOSS_001      1001 LQSLQTYVTQQLIRAAEIRASANLAATKMSECVLGQSKRVDFCGKGYHLM   1050

EMBOSS_001      1051 SFPQSAPHGVVFLHVTYVPAQEKNFTTAPAICHDGKAHFPREGVFVSNGT   1100
                     ||||||||||||||||||||||||||||||||||||||||||||||||||
EMBOSS_001      1051 SFPQSAPHGVVFLHVTYVPAQEKNFTTAPAICHDGKAHFPREGVFVSNGT   1100

EMBOSS_001      1101 HWFVTQRNFYEPQIITTDNTFVSGNCDVVIGIVNNTVYDPLQPELDSFKE   1150
                     ||||||||||||||||||||||||||||||||||||||||||||||||||
EMBOSS_001      1101 HWFVTQRNFYEPQIITTDNTFVSGNCDVVIGIVNNTVYDPLQPELDSFKE   1150

EMBOSS_001      1151 ELDKYFKNHTSPDVDLGDISGINASVVNIQKEIDRLNEVAKNLNESLIDL   1200
                     ||||||||||||||||||||||||||||||||||||||||||||||||||
EMBOSS_001      1151 ELDKYFKNHTSPDVDLGDISGINASVVNIQKEIDRLNEVAKNLNESLIDL   1200

EMBOSS_001      1201 QELGKYEQYIKWPWYIWLGFIAGLIAIVMVTIMLCCMTSCCSCLKGCCSC   1250
                     ||||||||||||||||||||||||||||||||||||||||||||||||||
EMBOSS_001      1201 QELGKYEQYIKWPWYIWLGFIAGLIAIVMVTIMLCCMTSCCSCLKGCCSC   1250

EMBOSS_001      1251 GSCCKFDEDDSEPVLKGVKLHYT   1273
                     |||||||||||||||||||||||
EMBOSS_001      1251 GSCCKFDEDDSEPVLKGVKLHYT   1273
```

Figure S2. Difference of discovered strain 2 spike protein sequence and original strain spike protein sequence

```
EMBOSS_001         1 MFLLTTKRTMFVFLVLLPLVSSQCVNLTTRTQLPPAYTNSFTRGVYYPDK     50
                     ||||||.||||||||||||||||||||.:|||||||||||||||||||.|
EMBOSS_001         1 MFLLTTIRTMFVFLVLLPLVSSQCVNLKSRTQLPPAYTNSFTRGVYYPGK     50

EMBOSS_001        51 VFRSSVLHSTQDLFLPFFSNVTWFHAIHVSGTNGTKRFDNPVLPFNDGVY    100
                     |||||||||||||||||||||||||||||||.||||||||||||||||||
EMBOSS_001        51 VFRSSVLHSTQDLFLPFFSNVTWFHAIHVSGTIGTKRFDNPVLPFNDGVY    100

EMBOSS_001       101 FASTEKSNIIRGWIFGTTLDSKTQSLLIVNNATNVVIKVCEFQFCNDPFL    150
                     |||||||.|||||||||||||||||||||||:||||||||||||||||||
EMBOSS_001       101 FASTEKSIIIRGWIFGTTLDSKTQSLLIVNNSTNVVIKVCEFQFCNDPFL    150

EMBOSS_001       151 GVYYHKNNKSWMESEFRVYSSANNCTFEYVSQPFLMDLEGKQGNFKNLRE    200
                     |||||||||||||||||||||||||||| |||||||||||||||||||||
EMBOSS_001       151 GVYYHKNNKSWMESEFRVYSSANNCTFE-VSQPFLMDLEGKQGNFKNLRE    199

EMBOSS_001       201 FVFKNIDGYFKIYSKHTPINLVRDLPQGFSALEPLVDLPIGINITRFQTL    250
                     ||||||||||||||||||||||||||||||||||||||||||||||||.:
EMBOSS_001       200 FVFKNIDGYFKIYSKHTPINLVRDLPQGFSALEPLVDLPIGINITRFQII    249

EMBOSS_001       251 LALHRSYLTPGDSSSGWTAGAAAYYVGYLQPRTFLLKYNENGTITDAVDC    300
                     |:||||||||||||||||||||||||||||||:|||||||||||||||||
EMBOSS_001       250 LSLHRSYLTPGDSSSGWTAGAAAYYVGYLQPRSFLLKYNENGTITDAVDC    299

EMBOSS_001       301 ALDPLSETKCTLKSFTVEKGIYQTSNFRVQPTESIVRFPNITNLCPFGEV    350
                     ||||||.||||||||||||||||||||||||||||||||||..|||||||
EMBOSS_001       300 ALDPLSGTKCTLKSFTVEKGIYQTSNFRVQPTESIVRFPNIKYLCPFGEV    349

EMBOSS_001       351 FNATRFASVYAWNRKRISNCVADYSVLYNSASFSTFKCYGVSPTKLNDLC    400
                     ||||||||||||||||||||||||||||||..||||||||||||||||||
EMBOSS_001       350 FNATRFASVYAWNRKRISNCVADYSVLYNSVPFSTFKCYGVSPTKLNDLC    399

EMBOSS_001       401 FTNVYADSFVIRGDEVRQIAPGQTGKIADYNYKLPDDFTGCVIAWNSNNL    450
                     ||||||||||||||||||||||||||||||||||||||||||||||||||
EMBOSS_001       400 FTNVYADSFVIRGDEVRQIAPGQTGKIADYNYKLPDDFTGCVIAWNSNNL    449

EMBOSS_001       451 DSKVGGNYNYLYRLFRKSNLKPFERDISTEIYQAGSTPCNGVEGFNCYFP    500
                     |||||||||||||||||||||||||||||||||||||||||||||||||.
EMBOSS_001       450 DSKVGGNYNYLYRLFRKSNLKPFERDISTEIYQAGSTPCNGVEGFNCYFL    499

EMBOSS_001       501 LQSYGFQPTNGVGYQPYRVVVLSFELLHAPATVCGPKKSTNLVKNKCVNF    550
                     ||.|||||||||||||||||||||||||||||||..||||||||||||||
EMBOSS_001       500 LQLYGFQPTNGVGYQPYRVVVLSFELLHAPATVCASKKSTNLVKNKCVNF    549

EMBOSS_001       551 NFNGLTGTGVLTESNKKFLPFQQFGRDIADT-------TDAVRDPQTLEI    593
                     ||||||||||||||||.|..|    .::|:|       ::..|..:.|.:
EMBOSS_001       550 NFNGLTGTGVLTESNKSFCLF----NNLAETLLTLLMLSEVHRHLRFLTL    595

EMBOSS_001       594 LDITPCSFGGVSVITP------------GTNTSNQVAVLYQGVNCTEVPV    631
                     ..:...    |||:..            ..:|:.:..:|:..:|...:.|
EMBOSS_001       596 HHVLLV----VSVLRQEQILLTRLLFFIRVSTAQKSLLLFMQINLLLLGV    641

EMBOSS_001       632 AIHADQLTPTWRVYSTGSNVFQTRAGCLIGAEHVNNSYECDIPIGAGICA    681
                     .| ...|.....|.:.|.|:..|         |::.:|    |:
EMBOSS_001       642 FI-LQVLKFLKHVQAVGLNMSTT---------HMSVTY----PL------    671

EMBOSS_001       682 SYQTQTNSPRRARSVASQSIIAYTMSLGAENSVAYSNNSIAIPTNFTISV    731
                      .|......|.:..:....::....||   :|:.:....|.:.|..|:..
EMBOSS_001       672 -VQVYALVIRLSLILLGGHVVLVNPSL---HSLCHLVQKIQLLTLITLLP    717
```

Figure S**3.** Difference of MR discovered strain 1 spike protein sequence and the MR assembled contig spike protein sequence

```
EMBOSS_001        732 TTEILPVSMTK---------TSVDCT---------MYICGDSTECSNLLL   763
                      ..:||.:.:.:         ..|.||         :|.|..:....|.:|
EMBOSS_001        718 YPQILLLVLPQKFYQCLPRHQIVQCTFGMIQLNAAIYCCNMAVFVHNTVL   767

EMBOSS_001        764 QYGSFCTQLNRALTGIAVEQDKNTQEVFAQVK----------QIYKTPP-   802
                           ...||:..|........|......|:|          ||::...
EMBOSS_001        768 -----LELLNKTKTPKKFVHKSNKFTKHHQLKILVVLIFHKFQIHQNQAR   812

EMBOSS_001        803 ---IKDFGGFNFSQILPDPSKPSKRSFIEDLLFNKVTLADAGFIKQYGDC   849
                         :|.:......|:|     .|..:.:..|:   :.|.:..|:.:
EMBOSS_001        813 GHLLKIYFSTKHLQLL-----ASSNNMVIALV---ILLLEPSFVHK----   850

EMBOSS_001        850 LGDIAARDLICAQKFNGLTVLPPLLTDEMIAQYTSALLAGTITS------   893
                          :...|:.....:.:.:|..||...::     :||.|.:..
EMBOSS_001        851 ----SLTALLFCHLCSQIRLLNTLLHCRVL-----SLLVGPLVQVLHYKY   891

EMBOSS_001        894 ----GWTFGAGA-ALQIPFAMQ----------MAYRFNGIGVTQNVLYEN   928
                          .|..|... .|...|:|:          :..:|....:.|....||
EMBOSS_001        892 HVLCKWLIGLMVLELHRMFSMRTKNLPTNLIVLLAKFKTHFLPQQANLEN   941

EMBOSS_001        929 QKLIANQFNSAIGKIQDSLSSTASALGKLQDVVNQNAQALNTLVKQLSSN   978
                      .|:    :::.:.|:...|::.|..|.:.|
EMBOSS_001        942 FKM----WSTKMHKLTRLLNNLAPILVQFQ--------------------   967

EMBOSS_001        979 FGAISSVLNDILSRLDKVEAEVQIDRLITGRLQSLQTY-VTQQLIRAAEI  1027
                         :..:...:|::|          ||....:.|...: |.:.::...|:
EMBOSS_001        968 ---VFMISFHVLTKL----------RLKCKLIGSQADFNVCRHMLNNLEL  1004

EMBOSS_001       1028 RASANLAATKMSEC---VLGQSKRVDFCGKGYHLMSFPQSAPHGVVF---  1071
                      :.|..|....:..|   .|...|.:.|..:.  ::..|..:.|.:||
EMBOSS_001       1005 QKSELLLILLLLNCQSVYLDNQKELIFVERA--IILCPSLSQHLMVFSCM  1052

EMBOSS_001       1072 ---LH--------VTYVPAQEKNFTTAPAI---CHDGKAHFPREGVFVSN  1107
                         ||        :.:| ..|..|....::   .|.|.    .:|:|:::
EMBOSS_001       1053 LMSLHKKRTSQLLLPFV-MMEHTFLVKVSLFQMAHTGL----HKGIFMNH  1097

EMBOSS_001       1108 -----GTHWF-------VTQRNFYEPQIITTDNTFVSGNCDVVIGIVNN-  1144
                           .||..       |:...|   .|:...||....:.: ::.|:::
EMBOSS_001       1098 KSLLQTTHLCLVTVMLEVSTTQF---MILCNLNTHSRRSIN-ILRIIHHQ  1143

EMBOSS_001       1145 -TVYDPLQPELD-SFKEELDKYFKNHTSPDVDLGDISGINASVVNIQKEI  1192
                       .:...|...|. ::|::|                       ..:::.:|
EMBOSS_001       1144 MLIVTSLALMLQLTYKKKL-----------------------TASMRLQI  1170

EMBOSS_001       1193 DRLNEVAKNLN--ESLIDLQELGK-----------------YEQYIKWPW  1223
                      ..|:.::|||.  .|::.:..|.:                 |:|.:....
EMBOSS_001       1171 MNLSSISKNLESMSSIMAMVHLARFYSWLDCHSNGDNYALLYDQLLLSQG  1220

EMBOSS_001       1224 YIWLGFIAGLIAIV-----MVTIMLCCMTSCCSCLKGCCSCGSCCKFDED  1268
                      .::||.:..:..:.     .:|:.:
EMBOSS_001       1221 LLFLGILLQIRRLASAQRSQITLHI-------------------------  1245

EMBOSS_001       1269 DSEPVLKGVKLHYT   1282

EMBOSS_001       1246 --------------   1245
```

Figure S**3.** Difference of MR discovered strain 1 spike protein sequence and MR assembled contig spike protein sequence

```
EMBOSS_001         1 MFLLTTKRTMFVFLVLLPLVSSQCVNLTTRTQLPPAYTNSFTRGVYYPDK     50
                     ||||||||||||||||||.|||||||||||||||||||||||||||||||
EMBOSS_001         1 MFLLTTKRTMFVFLVLLPLDSSQCVNLTTRTQLPPAYTNSFTRGVYYPDK     50

EMBOSS_001        51 VFRSSVLHSTQDLFLPFFSNVTWFHAIHVSGTNGTKRFDNPVLPFNDGVY    100
                     ||||||||||||||||||||||||||||||||||||||||||||||||||
EMBOSS_001        51 VFRSSVLHSTQDLFLPFFSNVTWFHAIHVSGTNGTKRFDNPVLPFNDGVY    100

EMBOSS_001       101 FASTEKSNIIRGWIFGTTLDSKTQSLLIVNNATNVVIKVCEFQFCNDPFL    150
                     ||||||||||||||||||||||||||||||||||||||||||||||||||
EMBOSS_001       101 FASTEKSNIIRGWIFGTTLDSKTQSLLIVNNATNVVIKVCEFQFCNDPFL    150

EMBOSS_001       151 GVYYHKNNKSWMESEFRVYSSANNCTFEYVSQPFLMDLEGKQGNFKNLRE    200
                     ||||||||||||||||||||||||||||||||||||||||||||||||||
EMBOSS_001       151 GVYYHKNNKSWMESEFRVYSSANNCTFEYVSQPFLMDLEGKQGNFKNLRE    200

EMBOSS_001       201 FVFKNIDGYFKIYSKHTPINLVRDLPQGFSALEPLVDLPIGINITRFQTL    250
                     |||||||||||||||||||||||||||||||||||||||:||||||||||
EMBOSS_001       201 FVFKNIDGYFKIYSKHTPINLVRDLPQGFSALEPLVDLPMGINITRFQTL    250

EMBOSS_001       251 LALHRSYLTPGDSSSGWTAGAAAYYVGYLQPRTFLLKYNENGTITDAVDC    300
                     ||||||||||||||||||||||||||||||||||||||||||||||||||
EMBOSS_001       251 LALHRSYLTPGDSSSGWTAGAAAYYVGYLQPRTFLLKYNENGTITDAVDC    300

EMBOSS_001       301 ALDPLSETKCTLKSFTVEKGIYQTSNFRVQPTESIVRFPNITNLCPFGEV    350
                     ||||||||||||||||||||||||||||||||||||||||||||||||||
EMBOSS_001       301 ALDPLSETKCTLKSFTVEKGIYQTSNFRVQPTESIVRFPNITNLCPFGEV    350

EMBOSS_001       351 FNATRFASVYAWNRKRISNCVADYSVLYNSASFSTFKCYGVSPTKLNDLC    400
                     ||||||||||||||||||||||||||||||||||||||||||||||||||
EMBOSS_001       351 FNATRFASVYAWNRKRISNCVADYSVLYNSASFSTFKCYGVSPTKLNDLC    400

EMBOSS_001       401 FTNVYADSFVIRGDEVRQIAPGQTGKIADYNYKLPDDFTGCVIAWNSNNL    450
                     ||||||||||||||||||||||||||||||||||||||||||||||||||
EMBOSS_001       401 FTNVYADSFVIRGDEVRQIAPGQTGKIADYNYKLPDDFTGCVIAWNSNNL    450

EMBOSS_001       451 DSKVGGNYNYLYRLFRKSNLKPFERDISTEIYQAGSTPCNGVEGFNCYFP    500
                     ||||||||||||||||||||||||||||||||||||||||||||||||||
EMBOSS_001       451 DSKVGGNYNYLYRLFRKSNLKPFERDISTEIYQAGSTPCNGVEGFNCYFP    500

EMBOSS_001       501 LQSYGFQPTNGVGYQPYRVVVLSFELLHAPATVCGPKKSTNLVKNKCVNF    550
                     ||||||||||||||||||||||||||||||||||||||||||||||||||
EMBOSS_001       501 LQSYGFQPTNGVGYQPYRVVVLSFELLHAPATVCGPKKSTNLVKNKCVNF    550

EMBOSS_001       551 NFNGLTGTGVLTESNKKFLPFQQFGRDIADTTDAVRDPQTLEILDITPCS    600
                     ||||||||||||||||||||||||||||||||||||||||||||||||||
EMBOSS_001       551 NFNGLTGTGVLTESNKKFLPFQQFGRDIADTTDAVRDPQTLEILDITPCS    600

EMBOSS_001       601 FGGVSVITPGTNTSNQVAVLYQGVNCTEVPVAIHADQLTPTWRVYSTGSN    650
                     ||||||||||||||||||||||||||||||||||||||||||||||||||
EMBOSS_001       601 FGGVSVITPGTNTSNQVAVLYQGVNCTEVPVAIHADQLTPTWRVYSTGSN    650

EMBOSS_001       651 VFQTRAGCLIGAEHVNNSYECDIPIGAGICASYQTQTNSPRRARSVASQS    700
                     ||||||||||||||||||||||||||||||||||||||||||||||||||
EMBOSS_001       651 VFQTRAGCLIGAEHVNNSYECDIPIGAGICASYQTQTNSPRRARSVASQS    700

EMBOSS_001       701 IIAYTMSLGAENSVAYSNNSIAIPTNFTISVTTEILPVSMTKTSVDCTMY    750
                     ||||||||||||||||||||||||||||||||||||||||||||||||||
EMBOSS_001       701 IIAYTMSLGAENSVAYSNNSIAIPTNFTISVTTEILPVSMTKTSVDCTMY    750
```

Figure S**4.** Difference of MR discovered strain 2 spike protein sequence and MR assembled contig spike protein sequence

```
EMBOSS_001       751 ICGDSTECSNLLLQYGSFCTQLNRALTGIAVEQDKNTQEVFAQVKQIYKT    800
                     ||||||||||||||||||||||||||||||||||||||||||||||||||
EMBOSS_001       751 ICGDSTECSNLLLQYGSFCTQLNRALTGIAVEQDKNTQEVFAQVKQIYKT    800

EMBOSS_001       801 PPIKDFGGFNFSQILPDPSKPSKRSFIEDLLFNKVTLADAGFIKQYGDCL    850
                     ||||||||||||||||||||||||||||||||||||||||||||||||||
EMBOSS_001       801 PPIKDFGGFNFSQILPDPSKPSKRSFIEDLLFNKVTLADAGFIKQYGDCL    850

EMBOSS_001       851 GDIAARDLICAQKFNGLTVLPPLLTDEMIAQYTSALLAGTITSGWTFGAG    900
                     ||||||||||||||||||||||||||||||||||||||||||||||||||
EMBOSS_001       851 GDIAARDLICAQKFNGLTVLPPLLTDEMIAQYTSALLAGTITSGWTFGAG    900

EMBOSS_001       901 AALQIPFAMQMAYRFNGIGVTQNVLYENQKLIANQFNSAIGKIQDSLSST    950
                     ||||||||||||||||||||||||||||||||||||||||||||||||||
EMBOSS_001       901 AALQIPFAMQMAYRFNGIGVTQNVLYENQKLIANQFNSAIGKIQDSLSST    950

EMBOSS_001       951 ASALGKLQDVVNQNAQALNTLVKQLSSNFGAISSVLNDILSRLDKVEAEV   1000
                     ||||||||||||||||||||||||||||||||||||||||||||||||||
EMBOSS_001       951 ASALGKLQDVVNQNAQALNTLVKQLSSNFGAISSVLNDILSRLDKVEAEV   1000

EMBOSS_001      1001 QIDRLITGRLQSLQTYVTQQLIRAAEIRASANLAATKMSECVLGQSKRVD   1050
                     ||||||||||||||||||||||||||||||||||||||||||||||||||
EMBOSS_001      1001 QIDRLITGRLQSLQTYVTQQLIRAAEIRASANLAATKMSECVLGQSKRVD   1050

EMBOSS_001      1051 FCGKGYHLMSFPQSAPHGVVFLHVTYVPAQEKNFTTAPAICHDGKAHFPR   1100
                     ||||||||||||||||||||||||||||||||||||||||||||||||||
EMBOSS_001      1051 FCGKGYHLMSFPQSAPHGVVFLHVTYVPAQEKNFTTAPAICHDGKAHFPR   1100

EMBOSS_001      1101 EGVFVSNGTHWFVTQRNFYEPQIITTDNTFVSGNCDVVIGIVNNTVYDPL   1150
                     ||||||||||||||||||||||||||||||||||||||||||||||||||
EMBOSS_001      1101 EGVFVSNGTHWFVTQRNFYEPQIITTDNTFVSGNCDVVIGIVNNTVYDPL   1150

EMBOSS_001      1151 QPELDSFKEELDKYFKNHTSPDVDLGDISGINASVVNIQKEIDRLNEVAK   1200
                     ||||||||||||||||||||||||||||||||||||||||||||||||||
EMBOSS_001      1151 QPELDSFKEELDKYFKNHTSPDVDLGDISGINASVVNIQKEIDRLNEVAK   1200

EMBOSS_001      1201 NLNESLIDLQELGKYEQYIKWPWYIWLGFIAGLIAIVMVTIMLCCMTSCC   1250
                     ||||||||||||||||||||||||||||||||||||||||||||||||||
EMBOSS_001      1201 NLNESLIDLQELGKYEQYIKWPWYIWLGFIAGLIAIVMVTIMLCCMTSCC   1250

EMBOSS_001      1251 SCLKGCCSCGSCCKFDEDDSEPVLKGVKLHYT   1282
                     ||||||||||||||||||||||||||||||||
EMBOSS_001      1251 SCLKGCCSCGSCCKFDEDDSEPVLKGVKLHYT   1282
```

Figure S**4.** Difference of MR discovered strain 2 spike protein sequence and MR assembled contig spike protein sequence
